# Identification of Pyrazolidine‐3‐One Derivatives as a Novel Structural Scaffold for ATP Synthase Inhibitors

**DOI:** 10.1002/cmdc.70399

**Published:** 2026-07-27

**Authors:** Lisa Reichert, Aro Delparente, Ida R. Hipfinger, Lukas Reininger, Ana Pinto Castro, Daniel J. Hubin, Roger Schibli, Amy E. Fraley, Linjing Mu

**Affiliations:** ^1^ Department of Chemistry and Applied Biosciences Institute of Pharmaceutical Sciences ETH Zürich Zurich Switzerland

**Keywords:** ATP synthase, in vitro evaluation, molecular docking, pyrazol‐3‐ones, pyrazolidin‐3‐ones

## Abstract

In a recent study, the oxadiazin‐5‐one‐based compound **CJ1‐34** was identified as a partial ATP synthase inhibitor, which was found to bind the F_1_ region of the ATP synthase. These findings were used as a starting point for the design and synthesis of smaller heterocycles, such as pyrazolidine‐3‐ones and pyrazol‐3‐ones, as novel structural scaffolds for potential ATP synthase inhibitors. Among the newly synthesized compounds, pyrazolidin‐3‐one derivatives **9a** and **10a** outperformed the lead compound **CJ1‐34** in vitro by inhibiting ATP hydrolytic activity and decreasing ATP levels in HT‐22 cells. Subsequent dose‐dependent studies identified compound **9a** as the most promising ATP synthase inhibitor. Molecular docking revealed similar binding modes for all compounds in the F1‐binding site and the calculated docking scores aligned with the measured IC_50_ values of the tested compounds. This study identified pyrazolidine‐3‐ones as promising structural scaffolds for ATP synthase inhibition, opening avenues for further biomedical applications in central nervous system diseases such as Alzheimer's and Parkinson's.

## Graphical abstract

Following the discovery of oxadiazin‐5‐ones as partial inhibitors of the ATP synthase, we herein report the synthesis of a library of pyrazolidine‐3‐ones and pyrazol‐3‐ones, designed by reducing the six‐membered N–N heterocycles to five‐membered rings. The newly synthesized compounds were evaluated via molecular docking and in vitro assays. Pyrazolidine‐3‐ones were identified as a novel class of ATP synthase inhibitors, supporting their potential application as imaging probes for neurodegenerative diseases.

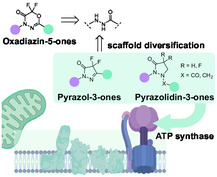

## 1 Introduction

The ATP synthase is an essential component of the mitochondrial electron transport chain and is directly responsible for cellular ATP production. Its dysfunction has been closely associated with various conditions including ischemia, cancer, metabolic disorders, and neurodegenerative diseases [1, 2, 3, 4]. A range of small molecules and oligopeptides have been identified as inhibitors of the ATP synthase. Among them, several have shown promising preclinical efficacy in reducing myocardial infarct size (BTB06584) [5, 6], or exhibiting antitumour activity (Bedaquiline, Gboxin) [5, 6, 7]. Polyphenols such as piceatannol, quercetin, and resveratrol have also been associated with diverse pharmacological effects, including neuroprotection [8, 9, 10].

**FIGURE 1:**
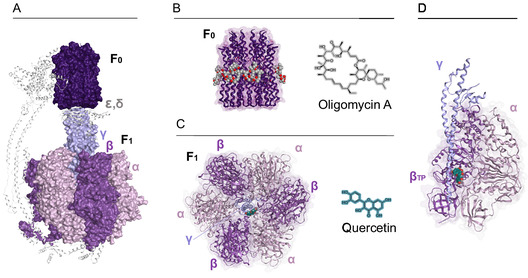
(A) Representation of the human F_0_F_1_ ATP synthase protein (PDB: 8H9T). Subunits: F_0_ dark violet, F_1_ γ subunit lilac, F_1_ α subunits pink, F_1_ β subunits purple [11]. (B) Representation of the F_0_ domain c‐ring subunit of yeast ATP synthase in complex with the natural product Oligomycin A (grey) (PDB: 4F4S) [6]. (C) Representation of the bovine F_1_ subunit of ATP synthase (view from the bottom facing away from the membrane) in complex with the small molecule quercetin (turquoise) (PDB: 2JJ2) [7]. (D) Magnified view of the binding interaction between small molecule quercetin (turquoise) and the F_1_ subunits γ, β_TP_, and α (PBD: 2JJ2) [7].

The ATP synthase consists of two domains F_0_ and F_1_. F_0_ is embedded in the inner mitochondrial membrane, while the F_1_ domain is located in the mitochondrial matrix and consists of six alternating α and β subunits. Furthermore, F_1_ contains γ, δ, and ε subunits that perform the rotary motion in the F_1_ region and make up the central stalk between the two domains [5]. Inhibition of the ATP synthase can be achieved by binding the F_0_ unit, for example, with the natural product Oligomycin A (Figure 1B) [6]. Alternatively, targeting the F_1_ domain has been achieved with the natural products quercetin, resveratrol, and piceatannol, all of which have been structurally validated by crystallization of the F_1_ unit in complex with the small molecules [7, 12]. They share a hydrophobic binding pocket at the bottom of the F_1_ unit facing away from F_0_ domain (Figure 1C). Closer inspection shows that quercetin interacts with the C‐terminal tip of the γ‐subunit, the β_TP_, and an α subunit (Figure 1D) [7].

For central nervous system (CNS) applications, candidate compounds must possess physicochemical properties that enable efficient blood‐barrier penetration. However, most reported ATP synthase inhibitors have unfavorable properties due to their size or polarity, vastly limiting the number of suitable candidates. Starting from piceatannol and quercetin, we recently developed a novel class of six membered N–N heterocyclic oxadiazin‐5‐one based scaffolds through iterative design and in vitro screening. Amongst these compounds, **CJ1‐34** demonstrated improved physiochemical properties for a CNS drug compared with the original polyphenols, as well as increased ATP synthase modulation and specificity. The compound is presumed to bind the same active site as resveratrol, piceatannol, and quercetin, a hypothesis which is supported by molecular docking studies [8].

As part of our ongoing efforts to further develop and optimize ATP synthase inhibitors, we aim to expand our library by reducing the N–N‐heterocycles to five or four‐membered rings. Five‐membered pyrazolidine‐3‐ones, pyrazol‐3‐ones and four‐membered aza‐β‐lactams are of considerable interest due to their broad relevance in medicinal chemistry and synthetic methodology [9, 10, 13]. The therapeutic potential of the pyrazol‐3‐one scaffold is exemplified by edaravone, an FDA‐approved drug for the treatment of amyotrophic lateral sclerosis, originally developed as a free‐radical scavenger for ischemic stroke. The synthesis of both 4,4‐difluoro substituted and unsubstituted pyrazolidine‐3‐ones was accomplished by modifying previously reported synthetic method for **CJ1‐34**. During our attempts to synthesize four‐membered N–N heterocycles by a zinc‐mediated condensation of *N*‐acyl diazene with either ethyl 2‐bromo‐2,2‐difluoroacetate or 2‐bromo‐2,2‐difluoroacetyl chloride, the desired products were not formed. Instead, we discovered the formation of a pyrazol‐3‐one scaffold by converting *N*‐acyl diazene with 2‐bromo‐2,2‐difluoroacetyl chloride. Although the exact reaction mechanism remains unclear, the influence of several reaction parameters was examined; a detailed mechanistic investigation, however, is beyond the scope of the present study. Preliminary in vitro evaluation of their effects on ATP synthase activity suggests that pyrazolidine‐3‐ones represent promising scaffolds for the development of ATP synthase inhibitors.

## 2 Results and Discussion

### 2.1 Synthesis of Pyrazol‐3‐Ones

**SCHEME 1:**
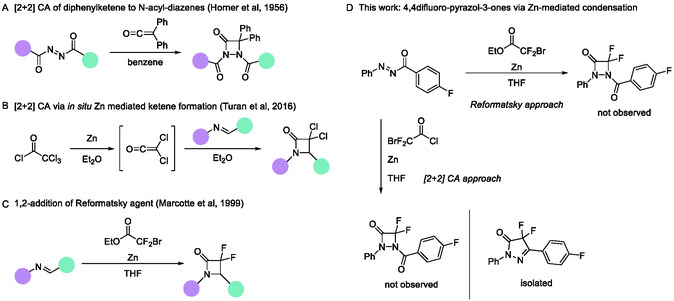
Selected approaches towards the synthesis of *gem*‐difunctionalized β‐lactams. (A) [2 + 2] cycloaddition of diphenylketene to (E)‐diazene‐1,2‐diylbis(phenylmethanone) as reported by Horner et al. [16]. (B) Zn‐mediated dehalogenation of 2,2,2‐trichloroacetylchloride as method to generate 2,2‐dichloroketene in situ as reported by Turan et al. [17]. (C) Zn‐mediated Reformatsky 1,2‐addition of ethyl 2‐bromo‐2,2‐difluoroacetate to imines as reported by Marcotte et al. [18]. (D) Application of selected synthesis strategies toward *gem*‐difluoro aza‐β‐lactams. Formation of a 4,4‐difluoro pyrazol‐3‐one scaffold was observed.

*Gem*‐functionalized β‐lactams and aza‐β‐lactams represent a promising scaffold for drug discovery. Such structures have been reportedly achieved by [2 + 2] cycloaddition of ketenes to diazenes [14, 15], *N*‐acyl‐diazenes (Scheme 1A) [19, 20], and imines [17, 21]. Due to their increased electrophilicity, 2,2‐dihalogenketenes are considered highly unstable and readily polymerize even at very low temperatures. 2,2‐Dihalogenketenes have reportedly been prepared in situ by zinc‐mediated dehalogenation [22]. Turan et al. reported the [2 + 2] cycloaddition of imines and 2,2‐dichloroketene, which was generated from 2,2,2‐trichloro acetylchloride, to produce *gem*‐dichloro‐β lactams (Scheme 1B) [17].

The synthesis of *gem*‐difluoro aza‐β‐lactams remains a yet unresolved challenge, while several methods have been reported to afford the closely related *gem*‐difluoro β‐lactams [23]. An effective strategy involves the 1,2‐addition of *gem*‐difluoromethylene‐organometallic reagents to imine derivates by a Reformatsky reaction as reported by Marcotte et al. (Scheme 1C) [18].

In our attempt to synthesize these type of compounds as bioisosteres for the previously discovered **CJ1‐34**, we investigated the reactivity of a model *N*‐acyl‐diazene compound, (E)‐((4‐fluorophenyl)(phenyldiazenyl)methanone), under reaction conditions for [2 + 2] cycloaddition of in situ generated difluoroketene as well as 1,2‐addition with ethyl 2‐bromo‐2,2‐difluoroacetate under Reformatsky reaction conditions. Although neither of these conditions afforded the desired *gem*‐difluoro aza‐β‐lactam, we observed the formation of a 4,4‐difluoro‐pyrazol‐3‐one scaffold (Scheme 1D). While such scaffolds have previously been accessed via condensation of hydrazine with alkyl‐2,2‐difluoro‐3‐oxo‐propanoates under acidic conditions [24] or from 2,4‐dihydro‐3H‐pyrazol‐3‐ones via mechanochemical reaction with Selectfluor [25], the observed reaction has not yet been reported. To explore the nature of the reaction and to find optimized conditions for this conversion, we screened several solvents, temperatures, concentrations, and reagent equivalents (Table 1). Conversion to **B** was quantified by ^19^F‐NMR with internal standard (Figures S1–S4).

**TABLE 1:** Exploration of reaction conditions and reaction optimization.

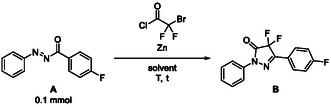							
Entry	Eq. acid chloride	Eq. Zn	Solvent	Concentration, mM	Temperature	Time	Yield^a^
1	1.5	0	THF	0.83	−15 °C	1 h	0%
2	1.5	3.0	THF	0.83	−15 °C	1 h	13.2%
3	1.5	3.0	THF	0.83	0 °C	1 h	8.5%
4	1.5	3.0	THF	0.83	rt	1 h	11.4%
5	1.5	3.0	THF	0.83	50 °C	1 h	14.1%
6	1.5	3.0	CH_2_Cl_2_	0.83	−15 °C	1 h	0%
7	1.5	3.0	CHCl_3_	0.83	50 °C	1 h	0%
8	1.5	3.0	toluene	0.83	50 °C	1 h	0%
9	1.5	3.0	THF	1.66	50 °C	1 h	18.4%
10	1.5	3.0	THF	0.42	50 °C	1 h	15.4%
11	3.0	3.0	THF	1.66	50 °C	1 h	24.1%
12	1.5	5.0	THF	1.66	50 °C	1 h	13.6%

a Yield determined by ^19^F‐NMR integration using CFCl_3_ as quantified internal standard.

As summarized in Table 1, evaluation of the reaction parameters indicates that the choice of solvent and the presence of zinc are both critical. The absence of Zn results in no product formation, indicating its essential role in the reaction mechanism. Furthermore, THF is the only solvent that is effective in this reaction, as no conversion to the desired product is detected in the remaining tested solvents. Notably, applying the optimized conditions to the corresponding hydrazine instead of the diazine results in no observable conversion.

Based on these observations, we hypothesize that the underlying mechanism has similarity to a Reformatsky‐type reaction. Starting from the insertion of zinc into the carbon–halogen bond of the α‐bromine, the respective alkylzinc reagent is formed. This structure could be stabilized by coordination with THF, explaining the solvent's importance. However, the relatively low yield precludes a definitive mechanistic assignment. Nevertheless, the unexpected formation of the five‐membered product of 4,4‐difluoro‐pyrazol‐3‐one has promoted further exploration of this approach to access additional pyrazol‐3‐one scaffold derivatives.

### 2.2 Scaffold Scope of 4,4‐Difluoro‐Pyrazol‐3‐Ones

**FIGURE 2:**
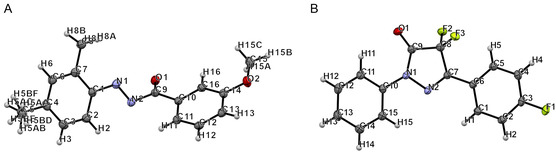
Determined X‐ray crystal structures of **4d** (A) and **5b** (B).

**SCHEME 2:**
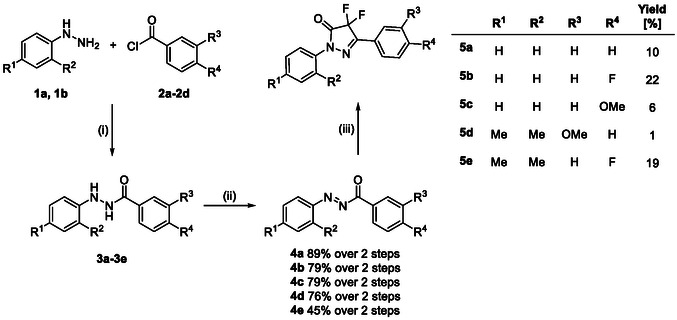
General synthetic route to 4,4‐difluoro‐2,4‐dihydro‐pyrazol‐3‐ones from commercially available hydrazines. (i) 2,6‐lutidine, −18 °C, 15 h; (ii) Mn(IV)O_2_ (3.0 eq), CH_2_Cl_2_, r.t., 3 h; and (iii) 2,2‐difluoro‐2‐bromo‐acetylchloride (3.0 eq), Zn (3.0 eq), THF, 50 °C, 1 h.

We applied the optimized conditions to synthesize a small library of 4,4‐difluoro‐pyrazol‐3‐one scaffolds to investigate the influence of different substituents on the reaction, and to evaluate their potential as ATP synthase inhibitors (Scheme 2). The *N*‐acyl‐diazene derivatives (**4a**–**4e**) were prepared by reacting commercially available hydrazines (**1a**–**1b**) with substituted benzoyl chlorides (**2a**–**2d**), followed by oxidation with manganese(IV) oxide (MnO_2_). This two‐step sequence afforded intermediates **4a**–**4e** in 45–89 % yield over all steps. The molecular structure of **4d** was confirmed by analyzing its X‐ray crystal structure (Figure 2A). The corresponding crystallographic data are provided in the Supporting Information (Tables S15–S21). Subsequent transformations under the optimized conditions (Table 1, entry 11) produced the corresponding 4,4‐difluoro‐pyrazol‐3‐ones (**5a**–**5e**). The yields were found to be sensitive to the electronic and steric properties of the substituents (R3, R4) on the aromatic ring. Substrates **5b** and **5e** (R4 = F) showed a significant increase in conversion, most likely due to the increased electrophilicity of the N‐acyl carbonyl group.

### 2.3 X‐Ray Crystal Structures of 4d and 5b

**4d** and **5b** were crystallized by vapor diffusion from diethyl ether and hexane. X‐ray crystallography confirmed the chemical structures of compounds **4d** and **5b** (Figure 2). The 2,4‐dihydro‐pyrazol‐3‐one moiety in compound **5b** is characterized by the angles C9‐N1‐N2 (112.70°), N1‐C9‐C8 (104.29°), C7‐C8‐C9 (102.83°), N2‐C7‐C8 (110.64°), and C7‐N2‐N1 (109.57°) as well as the double bond N2‐C7 (1.2888 Å) and single‐bonds N1‐N2 (1.4171 Å), C7‐C8 (1.4994 Å), C8‐C9 (1.5385 Å), and N1‐C9 (1.3624 Å). Detailed crystallographic data can be found in the supplementary information of this article (Tables S1–S7 and S15–S21).

### 2.4 Synthesis of Pyrazolidin‐3‐Ones

**SCHEME 3:**
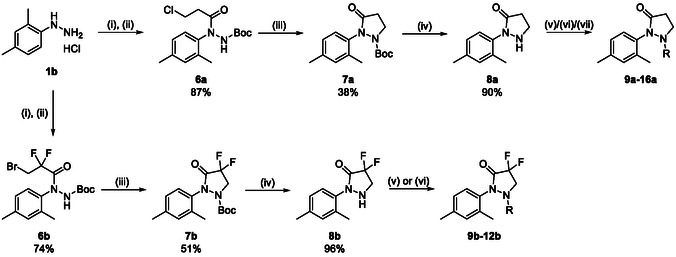
Synthetic route for pyrazolidine‐3‐ones. (i) Boc_2_O (1.0 eq), TEA (2.0 eq), THF, 0 °C‐r.t., 2 h; (ii) 3‐chloropropanoyl chloride or 3‐bromo‐2,2‐difluoropropanoyl chloride (1.1 eq), NaHCO_3_ (1.0 eq), toluene, 80 °C, 3 h; (iii) KO^*t*^Bu (1.2 eq), THF, 0 °C‐r.t., 2 h; (iv) 25 % TFA in CH_2_Cl_2_, r.t., 1 h; (v) acyl chloride (1.2–1.5 eq), TEA (3.0 eq), DMAP (5 mol%), CH_2_Cl_2_, r.t., 2–3 h; (vi) 1‐(bromomethyl)‐3‐methoxybenzene (2.0 eq), K_2_CO_3_ (3.0 eq), DMF, r.t., 16 h; (vii) 3‐fluorobicyclo [1.1.1]pentane‐1‐carboxylic acid (1.5 eq), HATU (2.5 eq), DIPEA (5.0 eq), DMF, r.t., 1 h.

We previously reported the synthetic route toward oxadiazin‐5‐ones from hydrazines. The key step of this process is an intramolecular cyclization via *N*‐alkylation mediated by KO^*t*^Bu [8]. We based this new synthetic approach towards pyrazolidine‐3‐ones on these findings. Scheme 3 depicts the synthetic route towards pyrazolidine‐3‐ones (**9a**‐**16a**) and 4,4‐difluoro‐pyrazolidin‐3‐ones (**9b**‐**12b**). Consecutive acylation of commercially available hydrazine hydrochloride **1b** afforded intermediates **6a** and **6b**, respectively. Distal Boc‐protection is required to direct the subsequent nucleophilic attack of the alkylhalide on the proximal amine. This allows for the desired intramolecular cyclization mediated by KO^*t*^Bu to afford pyrazolidine‐3‐ones **7a** and **7b**, which were then deprotected to yield **8a** and **8b,** respectively. Derivatization of the scaffold was achieved by acylation of **8a** and **8b** with either an acid or an acetyl chloride, or *N*‐alkylation of **8a** and **8b** under basic conditions. Following this route, we synthesized a library of 12 pyrazolidin‐3‐one scaffolds substituted by diverse benzoyl, benzyl and cycloalkyl moieties (Scheme 3). The structure of **9a** was further confirmed by single‐crystal X‐ray analysis. The X‐ray crystal structure and detailed crystallographic data are provided in the Supporting information (Figure S5 and Tables S8–S14).

The ability of compounds to penetrate the blood‐brain‐barrier (BBB) was predicted using the CNS multiparameter optimization (MPO) value (Table 2). This score is based on the interplay of the physiochemical properties of the compound (cLogP, cLogD, molecular weight (MW), topological polar surface area (TPSA), hydrogen bond donors (HBD), and pKa). This system ranks compounds on a scale from 0 to 6, where a higher CNS MPO value aligns with increased BBB permeability [26]. The physiochemical properties of the selected scaffolds were calculated using Percepta (ACD labs, Toronto, Canada). The designed scaffolds have CNS MPO values in a similar range as **CJ1‐34**.

**TABLE 2:** Summary of all synthesized pyrazolidine‐3‐one scaffolds and their evaluation in vitro.

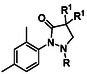						
Entry	R^1^	R	Yield, %	CNS MPO value	ATP hydrolysis^a^ (*p* value, Figure 3B)	ATP content, %^b^ (Figure 3A)
CJ1‐34^c^				5.46	0.0544	−21.0 ± 6.0
9a	H	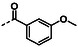	77	5.73	0.0172	−24.53 ± 7.15
10a	H	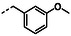	65	4.65	0.0019	−31.17 ± 8.05
11a	H	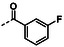	71	5.69	0.0039	−8.35 ± 7.47
12a	H	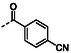	46	5.80	0.0936	+4.57 ± 14.6
13a	H	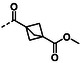	63	6.00	0.8376	−0.77 ± 10.49
14a	H	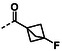	42	6.00	>0.9999	−30.43 ± 7.6
15a	H	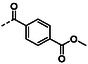	57	5.61	>0.9999	−4.09 ± 10.05
16a	H	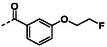	59	5.71	0.0065	−16.23 ± 10.05
9b	F	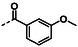	94	5.68	0.0004	−18.81 ± 9.73
10b	F	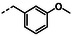	3	4.68	n.d.	n.d.
11b	F	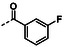	88	5.79	0.0006	−2.17 ± 14.87
12b	F	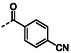	57	5.86	0.0019	−4.74 ± 9.26

a Inhibition of the ATP hydrolytic capacity of ATP synthase with the newly synthesized compound at 100 nM concentration in isolated mitochondria from mouse liver.

b Inhibition of the ATP content after 15 min treatment of HT‐22 cells with the tested compounds.

c Was synthesized and tested previously [8].

### 2.5 In Vitro Evaluation of Pyrazolidine‐3‐Ones

**FIGURE 3:**
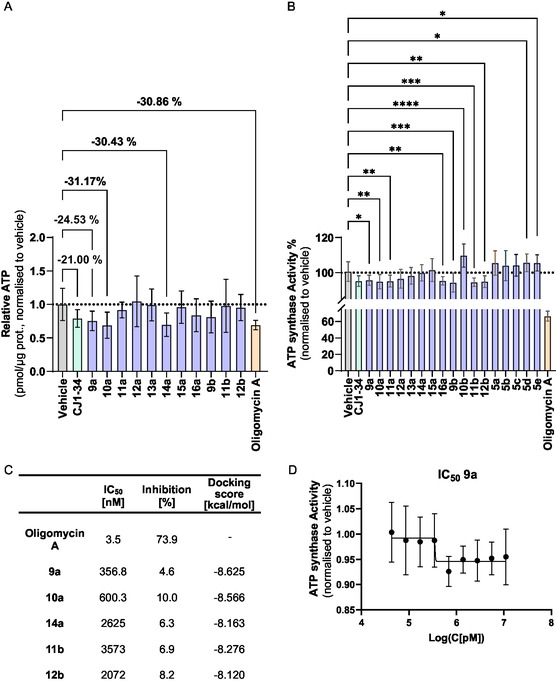
(A) The relative ATP production of neuronal HT22 cells after 15 min incubation with 1 μM tested compounds normalized to a vehicle sample (*n* = 3–4 biological replicates). (B) Normalized ATP hydrolytic activity of each compound was measured at a concentration of 100 nM in isolated liver mitochondria (*n* = 4–5 biological replicates). (C) Half‐maximal inhibitory concentration and percentage inhibition of ATP synthase were measured for a selection of compounds in isolated liver mitochondria; docking scores for the induced fit model are provided for compounds. (D) Dose‐dependent Inhibitory effect of **9a** on ATP synthase activity in isolated liver mitochondria (*n* = 2 biological samples, each in 3 replicates). Standard deviation is shown. Statistical analysis was performed using one‐way ANOVA followed by Dunnett's multiple comparison post‐test. *p < 0.05, **p < 0.01, ***p < 0.001, ****p < 0.0001.

Thomas et al. previously described the dynamic effects of ATP synthase inhibitors on cellular ATP levels. Initial depletion of ATP levels upon incubation with piceatannol was observed after 15 min. This is followed by a transient increase in ATP levels, which is attributed to compensatory mechanisms initiated by the AMP‐activated protein kinase signaling pathway [27]. We applied these findings to evaluate the effect of the compounds on the ATP content in HT‐22 mouse hippocampal neuronal cells after 15 min incubation time. The short‐term decrease in cellular ATP levels can be detected by a luciferase‐catalysed assay that measures ATP‐dependent oxidation of luciferin via bioluminescence [27]. Oligomycin A was used as a positive control to verify the assay. Of the compounds tested Figure 3A), compound **9a** (−24.53 ± 7.15 %), compound **10a** (−31.17 ± 8.05 %), and compound **14a** (−30.43 ± 7.6 %) showed a notable decrease in ATP levels in HT‐22 cells, which are improved compared to that of **CJ1‐34** (−21.0 ± 5.98 %).

While ATP synthase is mainly known for its capability to produce ATP, it also can hydrolyze ATP to ADP and inorganic phosphate (P_i_). In contrast to the ATP synthesis, this process is more specific to the enzyme complex itself [28]. We tested the effects of the newly synthesized compounds on the ATP hydrolytic activity of ATP synthase in isolated mouse liver mitochondria. In this assay, the rate of ATP hydrolysis is linked to the oxidation of NADH to NAD^+^. ADP generated by ATP hydrolysis is rephosphorylated by pyruvate kinase, which catalyzes the conversion of phosphoenolpyruvate to pyruvate. Subsequently, lactate dehydrogenase oxidizes NADH to NAD^+^ through the reduction of pyruvate to lactate. The resulting decrease in NADH can be quantified by absorbance measurements. Oligomycin A was selected as a positive control [8]. As illustrated in Figure 3B, most of the tested pyrazolidine‐3‐one compounds showed a significant inhibition of ATP synthase hydrolytic activity, as evidenced by a reduction in ATP levels within a range similar to that of **CJ1‐34**. In contrast, pyrazol‐3‐ones derivatives **5a–5e** unexpectedly enhanced ATP rather than inhibiting it and were therefore not investigated further. Additional dose–response experiments were performed to assess the ability of pyrazolidine‐3‐one compounds **9a**, **10a**, **14a**, **11b,** and **12b** to inhibit ATP synthase hydrolytic activity in isolated mitochondria. As shown in Figure 3C,D (see also Figure S6), the tested compounds inhibited ATP synthase hydrolytic activity by 5%–10% at the screening concentration, with corresponding IC_50_ values ranging between 357 and 3573 nM. Notably, pyrazolidine‐3‐one derivative **9a** reveals the highest potency among the tested compound (IC_50_ = 357 nM). The reference inhibitor oligomycin A exhibited an IC_50_ value of 3.5 nM, which is in good agreement with our previously reported value of 8.4 nM, indicating the robustness of the assay. Its high ATP synthase inhibition of 74% aligns with the reported range of 70‐80%.

Regarding the structure–activity relationship, the in vitro data indicate that pyrazolidin‐3‐one derivatives are a more favorable scaffold than pyrazol‐3‐one derivatives for ATP synthase inhibition. Comparison of pyrazolidine‐3‐one compounds **9a** and **10a** suggests that the carbonyl linker between the pyrazolidin‐3‐one core and the 3‐methoxyphenyl moiety contributes to increased potency, as reflected by the lower IC_50_ value of **9a** (357 nM) compared with **10a** (600 nM). Replacing the 3‐methoxyphenyl group of **9a** with a fluorinated bicyclic ring (**14a**) markedly reduced inhibitory activity (IC_50_ = 2625 nM). Likewise, introduction of two fluorine substituents into the pyrazolidin‐3‐one scaffold, as in compounds **11b** and **12b**, substantially decreased potency (IC_50_ > 2000 nM).

### 2.6 Molecular Docking

**FIGURE 4:**
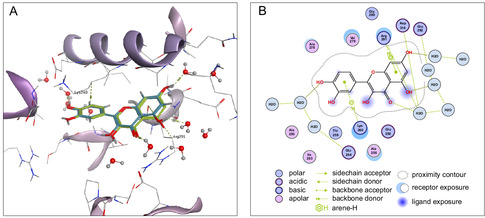
(A) Overlay of the crystal structures of ATP synthase in complex with quercetin crystal structure (green) and induced fit docked quercetin (blue). (B) Ligand Plot of quercetin in the active site.

To assess the interactions of these new ligands with the ATPase F_1_ active site, molecular docking was performed. The three crystal structures of ATPase F_1_ in complex with inhibitors quercetin, resveratrol and piceatannol were compared (Figure 4) and used for re‐docking each of the ligands to evaluate the docking performance. The overlay of the three structures shows that the pocket is spacious and can be filled in multiple modes. This is also reflected in the crystallographic data [7], where the authors describe that piceatannol, for instance, can bind the active site in four different orientations. To validate the docking protocol, the crystallized ligand quercetin was re‐docked using a consensus approach combining MOE Score, GOLDScore, ChemScore, and ASP within the MOE suite. Re‐docking of quercetin reproduced the experimental pose with high accuracy (Figure S7). Additionally, an independent docking workflow was performed using AutoDock Vina through the Pymol DockingPie Plugin, confirming that the correct binding mode is conserved across two different environments. RMSD analysis showed that all docking engines reproduced the crystallographic quercetin pose with deviations below 0.6 Å (Figure S7), demonstrating excellent agreement between the consensus docking in MOE and the AutoDock Vina‐based workflow in PyMOL.

Rigid docking and consensus scoring were first performed to validate the docking protocol and to assess binding mode stability. For the five inhibitors (**9a**, **10a**, **14a**, **11b**, **12b**), CJ1‐34 and quercetin as positive controls and caffeine as a negative control, poses were generated using MOE Score, GOLDScore, ChemScore, and ASP, followed by RMSD clustering (2.0 Å cutoff). Rank by rank analysis showed that compounds **9a** and **10a** consistently scored similar to **CJ1‐34** and better than compounds **14a, 11b**, and **12b** which is reflected in the IC_50_ values (see Table S22). Furthermore, caffeine consistently ranked last, providing an additional validation for the docking approach. The largest RMSD cluster for each ligand, together with the scoring functions supporting that cluster, was used to evaluate pose reproducibility and cross method agreement (Table S23). All largest clusters contained at least two independent scoring functions, demonstrating that the dominant pose family was not an artifact of a single scoring scheme but reflected a consensus across multiple interaction models.

**FIGURE 5:**
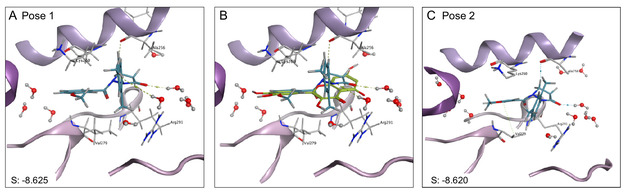
(A) Pose 1 of compound **9a** showing one aromatic ring perpendicular to the pyrazolidine‐3‐one ring stabilized by a π–water interaction, with the carboxylic oxygen is stabilized via a second water interaction. (B) Comparison of the crystalized quercetin structure with compound **9a**, showing clear occupation of the three‐dimensional space in the active site. (C) Pose 2 of compound **9a** displaying similar 3D occupation of the active site, including a π–hydrogen interaction with Val279.

Induced‐fit docking was performed for all ligands, retaining the conserved water molecules present in the ATPase F_1_ active site crystal structure (PDB 2JJ2). Quercetin engages with the water network extensively, supporting the conclusion that these waters play an important role in ligand recognition and stabilization. The induced‐fit binding mode were consistent with the rank order of the experimental IC_50_ values for compounds **9a**, **10a**, **14a**, **11b**, and **12b** (Figure 3). Although the best scoring pose (Figures 5 and S8) was not always the same as the dominant rigid‐dock cluster, it was present in every ligand's rigid‐dock ensemble, demonstrating that the biologically relevant geometry is consistently sampled but not always prioritized when the protein is held rigid. This reflects the fact that different scoring functions weight steric complementarity, hydrogen bonding, electrostatics, and hydrophobic contacts differently; thus, individual scoring functions may favor distinct pose families [29, 30, 31, 32, 33]. Allowing local side‐chain flexibility improves these discrepancies.

Interestingly, the best‐performing compound **9a** adopts a unique binding mode in which one aromatic ring is oriented perpendicular to the other two (Figure 5A). The resulting 3D geometry enables water‐mediated interactions, including a hydrogen bond from the conserved water network to the carboxylic group of the pyrazolidine‐3‐one ring, as well as an additional π–H interaction and a second stabilizing interaction of the pyrazolidine‐3‐one via H‐bond with Ala259. The overlay (Figure 5B) of this induced‐fit pose with quercetin, highlights that **9a** occupies the same interaction space but explores a distinct orientation that leverages structured water molecules for stabilization. The second‐best scoring pose, which exhibits a slight shift toward Val279 (Figure 5C), reproduces the interaction pattern commonly observed for other ligands in the series.

**FIGURE 6:**
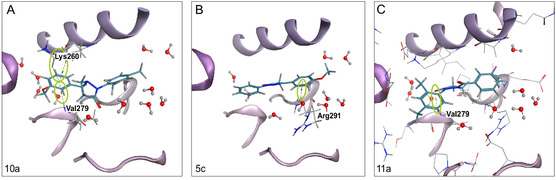
(A) π–Hydrogen bonding interaction between compound **10a** and Lys260 / Val279. (B) π–Hydrogen bonding interaction between Arg291 and compound **5c**. (C) Hydrogen bonding interaction between carboxylic oxygen of compound **11a** and Val279.

Most ligands maintain interactions with Lys260, Arg291, and Val279, primarily through π–hydrogen bonding (Figure 6; Table S24), positioning their aromatic systems in a planar orientation similar to that observed for quercetin, resveratrol, and piceatannol. Compounds **9b**, **10a**, **13a**, and **14a** form π–hydrogen interactions specifically with Lys291 (Figure 6A), anchoring the ligand in a geometry closely resembling the induced‐fit pose of the reference inhibitors. Compounds **5a–d** preferentially engage Arg291 through π–hydrogen bonding (Figure 6B), interacting with the other side of the active site. Additional polar interactions are observed for several ligands: compounds **5b** and **5e** form hydrogen bonds between their carboxylic oxygen and a nearby structured water molecule, whereas compound **5d** displays a C = O Val279 interaction. This Val279 contact is also present for **11a**, **12a**, and **12b** (Figure 6C), indicating that Val279 serves as a secondary anchoring point for multiple members of the series. Together, these interaction patterns highlight a conserved binding motif across the ligand set, with variations in π–hydrogen bonding partners and water mediated contacts impacting the placement of the ligands.

### 2.7 Radiosynthesis and In Vitro Evaluation of [^11^C]9a

**FIGURE 7:**
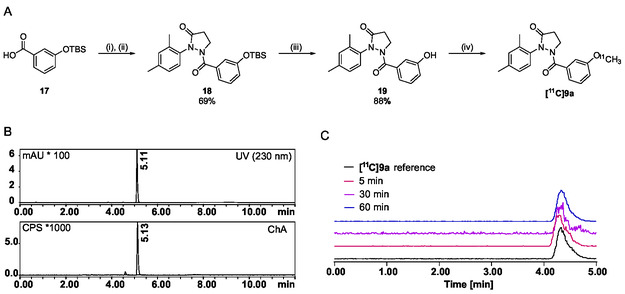
(A) Synthesis of the precursor of **19** and **[** ^**11**^ **C]9a**. (i) oxalyl chloride (1.3 eq), DMF (5 mol%), CH_2_Cl_2_, 0 °C‐r.t., 3 h; (ii) **8a** (0.83 eq), TEA (2.5eq), DMAP (5 mol%), CH_2_Cl_2_, r.t., 2 h; (iii) TBAF (1.5 eq), THF, −78 °C, 30 min; (iv) [^11^C]MeOTf, NaOH (2.0 eq), MeCN, 20 °C, 1 min. (B) Analytical radio‐HPLC chromatograms of purified **[** ^**11**^ **C]9a** co‐injected with non‐radioactive reference compound **9a**. Upper panel: UV‐detection (230 nm); lower panel: Radioactivity detection (GabiStar, Raytest). (C) In vitro stability of **[** ^**11**^ **C]9a** in mouse plasma after incubation at 37 °C for 5, 30, and 60 min.

Encouraged by these results, we established the radiosynthesis of **[** ^**11**^ **C]9a**, illustrated in Figure 7A. 3‐((Tert‐butyldimethylsilyl)oxy)benzoic acid (**17**) was transformed into the respective acyl chloride and reacted with **8a** to afford TBS‐protected intermediate **18**. Deprotection was achieved with TBAF to afford 3‐hydroxybenzoyl precursor **19**. **[** ^**11**^ **C]9a** was obtained via single‐step *O*‐alkylation of **19** using [^11^C]MeOTf under basic conditions. Purification by semi‐preparative HPLC afforded 8.3 ± 1.1 GBq (*n* = 5) of **[** ^**11**^ **C]9a** in high purity (>95 %) in 10 mL formulated solution (5 % EtOH in PBS). The radiosynthetic procedure was completed within 30 min from EOB and resulted in a molar activity in the range of of 333‐374 GBq/μmol (*n* = 3). The chemical identity of the product was confirmed by HPLC co‐injection of the non‐radioactive reference **9a** (Figure 7B).

To assess its in vivo compatibility, the stability and plasma protein binding of **[** ^**11**^ **C]9a** were evaluated in vitro in mouse plasma. Incubation in plasma at 37 °C for up to 60 min and subsequent analysis by ultra‐performance liquid chromatography revealed constant purity with no metabolites detected in the radiochromatograms, indicating stability in blood (Figure 7C). The free fraction of **[** ^**11**^ **C]9a** was determined to be 12.4 ± 0.3 % (*n* = 3) in mouse plasma.

## 3 Conclusion

In this study, we designed, synthesized, and evaluated a series of novel ATP synthase ligands, namely pyrazolidin‐3‐one and pyrazol‐3‐one derivatives, based on the oxadiazin‐5‐one scaffold of **CJ1‐34**. The compounds were assessed using in vitro assays to investigate their effects on ATP synthase function. Pyrazolidin‐3‐one derivatives **9a** and **10a** demonstrated superior inhibitory potency than the lead compound **CJ1‐34**, as evidenced by reduced ATP synthase hydrolytic activity and decreased intracellular ATP levels in HT‐22 cells. Notably, dose–response experiments in isolated bovine mitochondria identified compound **9a** as the most potent inhibitor, with an IC_50_ value of 357 nM. Molecular docking studies showed that the calculated docking scores consistent with the measured binding affinities in vitro and supported the F_1_ region of the ATP synthase as the potential binding site. Furthermore, compound **9a** was successfully radiolabeled with carbon‐11, leading to a promising new candidate for brain imaging. In conclusion, pyrazolidin‐3‐ones were identified as a novel class of ATP synthase inhibitors, warranting further investigation of their potential as imaging agents for neurodegenerative diseases.

## 4 Experimental Section

### 4.1 General Information

Unless otherwise stated, all reactions were carried out under inert atmosphere in commercial anhydrous solvents (Sigma–Aldrich GmbH). Nuclear magnetic resonance (NMR) spectra (^1^H, ^13^C, and if applicable ^19^F) of compounds were recorded on either a Bruker 400 MHz spectrometer or a Bruker 500 MHz spectrometer at r.t. (298 K). Chemical shifts (δ) are reported in parts per million (ppm) relative to trimethylsilane (0 ppm). Values of the coupling constant (*J*) are given in hertz (Hz). Multiplicities in the ^1^H NMR spectra are described as: singlet (s), doublet (d), triplet (t), quartet (q), multiplet (m), doublet of doublet (dd), triplet of doublet (td), and broad peak (br). The chemical shifts of complex multiplets are given as the range of their occurrence. High‐resolution mass spectrometry (HRMS) was performed by the Molecular and Biomolecular Analysis Service (MoBiAS) using a Bruker MaXis with ESI ion source and Qq‐TOF analyzer (ESI−Qq‐TOF‐MS) in positive mode and processed by the software Compass 1.5, HyStar 3.2.44, DataAnalysis 4.1, and BioTools 3.3. HRMS data are reported in m/z. Liquid chromatography mass spectrometry (LC‐MS) and purity were measured on an Agilent InfinityLab LC/MSD XT using an analytical reverse phase column (Waters BEH C18, 1.10x50.0 mm, 1.70 μm) with H_2_O/MeCN (both containing 0.1 % formic acid) as mobile phase. Method: 2 % MeCN from 0 to 1 min, gradient from 2 % to 98 % MeCN from 1 to 8 min, 98 % MeCN from 8 to 9 min, 0.600 mL/min, 1200.0 bar.

### 4.2 Chemistry

#### 4.2.1 General Synthetic Procedure for Pyrazol‐3‐One Derivates (5a‐5e)

Compounds **4a**‐**4e** (1.0 eq) and freshly activated Zn dust (3.0 eq) were suspended in THF (1.66 mM). 2‐bromo‐2,2‐difluoroacetyl chloride (3.0 eq) was added and the reaction mixture was heated to 50 °C. Upon completion (monitored by TLC), the mixture was filtered. The residue was washed with EtOAc, and the filtrate was concentrated under reduced pressure. Purification by flash column chromatography (SiO_2_, EtOAc in hexanes) afforded compounds **5a‐5e**.

#### 4.2.2 4,4‐Difluoro‐2,5‐Diphenyl‐2,4‐Dihydro‐3H‐Pyrazol‐3‐One (5a)

The characterization data of the compound is in accordance with reported spectra [25].

^**1**^ **H NMR** (500 MHz, CDCl_3_) δ 8.02–7.93 (m, 4H), 7.58–7.46 (m, 5H), 7.31 (td, *J* = 7.4, 1.2 Hz, 1H); ^**19**^ **F NMR** (471 MHz, CDCl_3_) δ −115.64. ^**19**^ **F NMR** (471 MHz, CDCl_3_) δ −115.64.

**HRMS (ESI+)**: calculated for for C_15_H_10_F_2_N_2_NaO [M+Na]^+^, 295.0653, found 295.0654 Δ −0.9 ppm

#### 4.2.3 4,4‐difluoro‐5‐(4‐fluorophenyl)‐2‐phenyl‐2,4‐dihydro‐3H‐pyrazol‐3‐one (5b)

^**1**^ **H NMR** (500 MHz, CDCl_3_) δ 8.00 (dd, *J* = 8.6, 5.4 Hz, 2H), 7.96 – 7.91 (m, 2H), 7.51 – 7.45 (m, 2H), 7.33 – 7.28 (m, 1H), 7.25 – 7.20 (m, 2H); ^**19**^ **F NMR** (471 MHz, CDCl_3_) δ −105.30, −115.72; ^**13**^ **C NMR** (126 MHz, CDCl_3_) δ 165.20 (d, *J* = 254.8 Hz), 159.14 (t, *J* = 29.8 Hz), 148.97 (t, *J* = 21.5 Hz), 136.77, 129.35, 129.21 (d, *J* = 8.9 Hz), 126.69, 122.77, 118.92, 116.86 (d, *J* = 22.2 Hz), 109.10 (t, *J* = 258 Hz); **HRMS (ESI+)**: calculated for C_15_H_9_F_3_N_2_NaO [M+Na]^+^, 313.0559, found 313.0562 Δ −0.9 ppm

#### 4.2.4 4,4‐Difluoro‐5‐(4‐Methoxyphenyl)‐2‐Phenyl‐2,4‐Dihydro‐3H‐Pyrazol‐3‐One (5c)

^**1**^ **H NMR** (500 MHz, CDCl_3_) δ 7.94 (dt, *J* = 7.9, 1.1 Hz, 2H), 7.55 (dt, *J* = 7.7, 1.3 Hz, 1H), 7.51 – 7.42 (m, 5H), 7.33 – 7.28 (m, 1H), 7.11 (ddd, *J* = 8.3, 2.6, 1.0 Hz, 1H), 3.90 (s, 3H); ^**19**^ **F NMR** (471 MHz, CDCl_3_) δ −115.45; ^**13**^ **C NMR** (126 MHz, CDCl_3_) δ 160.23, 159.37 (t, *J* = 29.9 Hz), 149.83 (t, *J* = 21.2 Hz), 136.82, 130.48, 129.32, 127.59, 126.65, 119.66, 118.99, 118.70, 111.35, 109.11 (t, *J* = 258.5 Hz), 55.63; **HRMS (ESI+)**: calculated for C_16_H_12_F_2_N_2_NaO_2_ [M+Na]^+^, 325.0759, found 325.0759 Δ 0 ppm

#### 4.2.5 2‐(2,4‐Dimethylphenyl)−4,4‐Difluoro‐5‐(3‐Methoxyphenyl)−2,4‐Dihydro‐3H‐Pyrazol‐3‐One (5d)

^**1**^ **H NMR** (400 MHz, CDCl_3_) δ 7.49 (dq, *J* = 7.6, 1.3 Hz, 1H), 7.45 – 7.38 (m, 2H), 7.27–7.25 (m, 1H), 7.17 – 7.11 (m, 2H), 7.08 (ddd, *J* = 8.2, 2.6, 1.0 Hz, 1H), 3.86 (s, 3H), 2.38 (s, 3H), 2.27 (s, 3H); ^**19**^ **F NMR** (376 MHz, CDCl_3_) δ −116.84; ^**13**^ **C NMR** (101 MHz, CDCl_3_) δ 160.20, 149.73 (t, *J* = 20.9 Hz), 139.88, 135.09, 132.18, 131.81, 130.39, 127.81, 127.71, 126.41, 119.47, 118.69, 110.79, 108.74 (t, *J* = 258.7 Hz), 55.59, 21.31, 18.20.; **HRMS (ESI+)**: calculated for C_18_H_16_F_2_N_2_NaO_2_ [M+Na]^+^, 353.1072, found 353.107 Δ 0.6 ppm

#### 4.2.6 2‐(2,4‐Dimethylphenyl)−4,4‐Difluoro‐5‐(4‐Fluorophenyl)−2,4‐Dihydro‐3H‐Pyrazol‐3‐One (5e)

^**1**^ **H NMR** (400 MHz, CDCl_3_) δ 7.93 (ddt, *J* = 8.1, 4.5, 1.0 Hz, 2H), 7.25 – 7.10 (m, 5H), 2.38 (s, 3H), 2.27 (s, 3H); ^**19**^ **F NMR** (376 MHz, CDCl_3_) δ −105.85, −117.14; ^**13**^ **C NMR** (126 MHz, CDCl_3_) δ 164.99 (d, *J* = 254.5 Hz), 159.95 (t, *J* = 29.2 Hz), 148.86 (t, *J* = 21.3 Hz), 139.88, 135.00, 132.19, 131.75, 128.91 (d, *J* = 9.0 Hz), 127.71, 126.32, 122.98 (t, *J* = 1.9 Hz), 116.74 (d, *J* = 22.4 Hz), 108.72 (t, *J* = 258.7 Hz), 21.29, 18.19.; **HRMS (ESI+)**: calculated for C_17_H_13_F_3_N_2_NaO [M+Na]^+^, 341.0872, found 341.087 Δ 0.6 ppm

#### 4.2.7 General Synthetic Procedure for Pyrazolidine Derivates (9a, 11a‐13a, 15a, 16a, 9b, 11b, 12b, 18)

To a solution of **8a** or **8b** (1.0 eq) in CH_2_Cl_2_ (54‐59 mM) was added TEA (3.0 eq) and DMAP (5 mol%). The respective acyl chloride (1.2 or 1.5 eq) was added dropwise, and the reaction mixture was stirred at r.t. until completion (monitored by TLC) was reached. Sat. aq. NaHCO_3_ was added, the layers were separated, and the aqueous layer was extracted three times with CH_2_Cl_2_. The combined organic extracts were dried over MgSO_4_ or Na_2_SO_4_, filtered, and concentrated under reduced pressure. Purification by flash column chromatography (SiO_2_, EtOAc in hexanes) afforded compounds **9a, 11a‐13a, 15a, 16a, 9b, 11b, 12b, 18**.

#### 4.2.8 2‐(2,4‐Dimethylphenyl)‐1‐(3‐Methoxybenzoyl)pyrazolidin‐3‐One (9a)

^**1**^ **H NMR** (400 MHz, CDCl_3_): δ 7.39 – 7.30 (m, 2H), 7.27 (t, *J* = 1.4 Hz, 1H), 7.25 – 7.23 (m, 1H), 7.09 – 6.98 (m, 3H), 4.28 (t, *J* = 7.8 Hz, 2H), 3.82 (s, 3H), 2.80 (t, *J* = 7.8 Hz, 2H), 2.40 (s, 3H), 2.29 (s, 3H).

^**13**^ **C NMR** (101 MHz, CDCl_3_): δ 171.07, 169.49, 159.87, 138.19, 135.02, 134.13, 133.51, 131.88, 129.81, 127.16, 126.05, 120.93, 118.42, 113.92, 55.56, 50.21, 32.36, 21.18, 18.47.

**HRMS (ESI+)**: calculated for C_19_H_20_N_2_NaO_3_ [M+Na]^+^, 347.1366, found 347.1361 Δ 1.6 ppm

**LCMS (ESI+):** m/z found 325.0 [M+H]^+^, calculated for C_19_H_21_N_2_O_3_; **Purity by LC‐MS** (254 nm): >99%

#### 4.2.9 2‐(2,4‐Dimethylphenyl)‐1‐(3‐Fluorobenzoyl)pyrazolidin‐3‐One (11a)

^**1**^ **H NMR** (500 MHz, CDCl_3_) δ 7.49 (dt, *J* = 7.7, 1.3 Hz, 1H), 7.46 – 7.36 (m, 2H), 7.30 (d, *J* = 8.1 Hz, 1H), 7.23 (tdd, *J* = 8.2, 2.7, 1.1 Hz, 1H), 7.08 (d, *J* = 2.1 Hz, 1H), 7.03 (dd, *J* = 8.0, 2.0 Hz, 1H), 4.27 (t, *J* = 7.8 Hz, 2H), 2.82 (t, *J* = 7.8 Hz, 2H), 2.39 (s, 3H), 2.30 (s, 3H); ^**19**^ **F NMR** (471 MHz, CDCl_3_) δ −111.12; ^**13**^ **C NMR** (126 MHz, CDCl_3_) δ 169.82 (d, *J* = 2.4 Hz), 169.42, 162.69 (d, *J* = 248.9 Hz), 138.42, 135.10, 135.05 (d, *J* = 6.9 Hz), 133.40, 131.99, 130.65 (d, *J* = 7.8 Hz), 127.27, 126.06, 124.52 (d, *J* = 3.2 Hz), 119.48 (d, *J* = 21.3 Hz), 115.94 (d, *J* = 23.2 Hz), 50.07, 32.32, 21.23, 18.50; **HRMS (ESI+)**: calculated for C_18_H_18_FN_2_O_2_ [M+H]^+^, 313.1347, found 313.1339 Δ 2.4 ppm; **LC‐MS (ESI+)** m/z found 312.3 [M+H]^+^, calculated for C_18_H_18_FN_2_O_2_; **Purity by LC‐MS** (254 nm): >99%

#### 4.2.10 4‐(2‐(2,4‐Dimethylphenyl)‐3‐Oxopyrazolidine‐1‐Carbonyl)benzonitrile (12a)

^**1**^ **H NMR** (500 MHz, CDCl_3_) δ 7.80 – 7.71 (m, 4H), 7.27 (s, 1H), 7.09 – 7.05 (m, 1H), 7.03 (t, *J* = 7.8 Hz, 1H), 4.26 (t, *J* = 7.8 Hz, 2H), 2.84 (t, *J* = 7.8 Hz, 2H), 2.36 (s, 3H), 2.30 (s, 3H).

^**13**^ **C NMR** (101 MHz, CDCl_3_) δ 169.34, 169.05, 138.59, 137.09, 135.10, 133.35, 132.62, 132.01, 129.31, 127.30, 125.82, 117.82, 115.81, 49.74, 32.21, 21.20, 18.46.

**HRMS (ESI+)**: calculated for C19H18N3O2 [M+H]^+^, 320.1394, found 320.1390 Δ 1.1 ppm; **LC‐MS (ESI+)** m/z found 320.0 [M+H]^+^, calculated for C19H18N3O2; **Purity by LC‐MS** (254 nm): >96%

#### 4.2.11 Methyl 3‐(2‐(2,4‐Dimethylphenyl)‐3‐Oxopyrazolidine‐1‐Carbonyl)bicyclo [1.1.1] Pentane‐1‐Carboxylate (13a)

^**1**^ **H NMR** (400 MHz, CDCl_3_): δ 7.12 – 7.03 (m, 2H), 6.99 (dd, *J* = 8.1, 2.0 Hz, 1H), 4.28 (t, *J* = 7.9 Hz, 2H), 3.68 (s, 3H), 2.84 (t, *J* = 7.9 Hz, 2H), 2.36 (s, 6H), 2.29 (s, 6H); ^**13**^ **C NMR** (101 MHz, CDCl_3_) δ 169.42, 167.62, 138.23, 134.84, 133.99, 131.97, 127.24, 125.04, 53.59, 52.04, 46.90, 38.77, 38.47, 32.21, 21.21, 21.13, 18.42; **HRMS (ESI+)**: calculated for C_19_H_23_N_2_O_4_ [M+H]^+^, 343.1652, found 343.1646 Δ 1.7 ppm; **LC‐MS (ESI+)** m/z found 342.8 [M+H]^+^, calculated for C_19_H_23_N_2_O_4_; **Purity by LC‐MS** (254 nm): >99%

#### 4.2.12 Methyl 4‐(2‐(2,4‐Dimethylphenyl)‐3‐Oxopyrazolidine‐1‐Carbonyl)benzoate (15a)

^**1**^ **H NMR** (400 MHz, CDCl_3_) δ 8.13 – 8.06 (m, 2H), 7.78 – 7.71 (m, 2H), 7.30 (d, *J* = 8.1 Hz, 1H), 7.07 (d, *J* = 1.9 Hz, 1H), 7.02 (dd, *J* = 8.1, 2.0 Hz, 1H), 4.25 (t, *J* = 7.8 Hz, 2H), 3.94 (s, 3H), 2.81 (t, *J* = 7.8 Hz, 2H), 2.39 (s, 3H), 2.29 (s, 3H); ^**13**^ **C NMR** (101 MHz, CDCl_3_) δ 170.08, 169.41, 166.14, 138.40, 136.94, 135.11, 133.46, 133.45, 131.95, 130.00, 128.77, 127.25, 125.97, 52.63, 49.92, 32.30, 21.19, 18.48.; **HRMS (ESI +)**: calculated for C_20_H_20_N_2_NaO_4_ [M+Na]^+^, 375.1315, found 375.1313 Δ 0.5 ppm; **LC‐MS (ESI+)** m/z found 353.0 [M+H]^+^, calculated for C_20_H_21_N_2_O_4_; **Purity by LC‐MS** (254 nm): >99%

#### 4.2.13 2‐(2,4‐Dimethylphenyl)‐1‐(3‐(2‐Fluoroethoxy)benzoyl)pyrazolidin‐3‐One (16a)

^**1**^ **H NMR** (500 MHz, CDCl_3_) δ 7.31 (t, *J* = 7.9 Hz, 1H), 7.28 – 7.23 (m, 1H), 7.23 – 7.19 (m, 1H), 7.07 – 7.01 (m, 2H), 6.98 (dd, *J* = 8.1, 2.0 Hz, 1H), 4.67 (dt, *J* = 47.7, 4.1, 2H), 4.23 (t, *J* = 7.7 Hz, 2H), 4.18 (dt, *J* = 27.9, 4.1, 2H), 2.75 (t, *J* = 7.8 Hz, 2H), 2.36 (s, 3H), 2.25 (s, 3H).; ^**19**^ **F NMR** (376 MHz, CDCl_3_) δ −72.74.; ^**13**^ **C NMR** (126 MHz, CDCl_3_) δ 170.89, 169.48, 158.70, 135.05, 134.26, 131.90, 129.98, 127.18, 126.02, 114.56, 81.85 (d, *J* = 171.3 Hz), 67.43 (d, *J* = 20.3 Hz), 50.20, 32.35, 18.48; **HRMS (ESI +)**: calculated for C_20_H_22_FN_2_O_3_ [M+H]^+^, 357.1609, found 357.1607 Δ 0.6 ppm; **LC‐MS (ESI+)** m/z found 357.0 [M+H]^+^, calculated for C_20_H_22_FN_2_O_3_; **Purity by LC‐MS** (254 nm): >95%

#### 4.2.14 1‐(3‐((tert‐Butyldimethylsilyl)oxy)benzoyl)‐2‐(2,4‐Dimethylphenyl)pyrazolidin‐3‐One (18)

^**1**^ **H NMR** (400 MHz, CDCl_3_) δ 7.33–7.26 (m, 3H), 7.17–7.16 (m, 1H), 7.07–7.06 (m, 1H), 7.05–7.01 (m, 1H), 7.01–6.97 (m, 1H), 4.26 (t, *J* = 7.8 Hz, 2H), 2.80 (t, *J* = 7.8 Hz, 2H), 2.40 (s, 3H), 2.29 (s, 3H), 0.98 (s, 9H), 0.20 (s, 6H).; ^**13**^ **C NMR** (101 MHz, CDCl_3_) δ 171.00, 169.51, 156.12, 138.21, 134.93, 134.20, 133.48, 131.93, 129.89, 127.21, 126.15, 124.27, 121.68, 120.42, 50.18, 25.74, 21.21, 18.49, 11.66, −4.28.; **HRMS (ESI +)**: calculated for C_24_H_33_N_2_O_3_Si [M+H]^+^, 425.2255, found 425.2244 Δ 2.6 ppm

#### 4.2.15 2‐(2,4‐Dimethylphenyl)−4,4‐Difluoro‐1‐(3‐Methoxybenzoyl)pyrazolidin‐3‐One (9b)

^**1**^ **H NMR** (500 MHz, CDCl_3_) δ 7.41–7.32 (m, 2H), 7.28–7.21 (m, 2H), 7.11 (tdd, *J* = 3.8, 2.6, 1.0 Hz, 2H), 7.05 (dd, *J* = 8.1, 2.0 Hz, 1H), 4.41 (t, *J* = 12.8 Hz, 2H), 3.84 (s, 3H), 2.43 (s, 3H), 2.32 (s, 3H); ^**19**^ **F NMR** (471 MHz, CDCl_3_) δ −114.14; ^**13**^ **C NMR** (126 MHz, CDCl_3_) δ 171.65, 160.17, 158.42 (t, *J* = 29.9 Hz), 139.34, 132.78, 132.17, 132.13, 130.26, 127.37, 125.49, 120.85, 119.35, 114.20 (t, *J* = 257.4 Hz), 114.00, 56.82 (t, *J* = 28.2 Hz), 55.66, 21.25, 18.36; **HRMS (ESI +)**: calculated for C_19_H_18_F_2_N_2_NaO_3_ [M+Na]^+^, 383.1178, found 383.1176 Δ 0.6 ppm; **LC‐MS (ESI+)** m/z found 361.0 [M+H]^+^, calculated for C_19_H_20_F_2_N_2_O_3_; **Purity by LC‐MS** (254 nm): >99%

#### 4.2.16 2‐(2,4‐Dimethylphenyl)−4,4‐Difluoro‐1‐(3‐Fluorobenzoyl)pyrazolidin‐3‐One (11b)

^**1**^ **H NMR** (400 MHz, CDCl_3_) δ 7.52–7.44 (m, 2H), 7.40 (ddd, *J* = 8.8, 2.8, 1.2 Hz, 1H), 7.33 (d, *J* = 8.1 Hz, 1H), 7.32–7.26 (m, 1H), 7.12 (d, *J* = 2.0 Hz, 1H), 7.06 (dd, *J* = 8.1, 2.0 Hz, 1H), 4.40 (t, *J* = 12.7 Hz, 2H), 2.42 (s, 3H), 2.32 (s, 3H); ^**19**^ **F NMR** (376 MHz, CDCl_3_) δ −110.15, −110.16, −110.17, −110.18, −110.19, −110.21, −114.16; ^**13**^ **C NMR** (101 MHz, CDCl_3_) δ 170.44, 164.05, 161.57, 139.51, 135.12, 133.65, 132.22, 131.98, 131.16, 131.08, 127.43, 125.49, 124.55, 120.55, 120.34, 116.12, 115.89, 114.08, 56.92, 56.64, 56.36, 21.25, 18.34; **HRMS (ESI +)**: calculated for C_18_H_15_F_3_N_2_NaO_2_ [M+Na]^+^, 371.0978, found 371.0975 Δ 0.6 ppm; **LC‐MS (ESI+)** m/z found 349.0 [M+H]^+^, calculated for C_18_H_16_F_3_N_2_O_2_; **Purity by LC‐MS** (254 nm): >99%

#### 4.2.17 4‐(2‐(2,4‐Dimethylphenyl)−4,4‐Difluoro‐3‐Oxopyrazolidine‐1‐Carbonyl)benzonitrile (12b)

^**1**^ **H NMR** (500 MHz, CDCl_3_) δ 7.83–7.74 (m, 4H), 7.12 (d, *J* = 2.0 Hz, 1H), 7.06 (dd, *J* = 8.1, 2.0 Hz, 1H), 4.38 (t, *J* = 12.5 Hz, 2H), 2.39 (s, 3H), 2.32 (s, 3H); ^**19**^ **F NMR** (471 MHz, CDCl_3_) δ −114.30; ^**13**^ **C NMR** (126 MHz, CDCl_3_) δ 169.98, 158.27 (t, *J* = 29.6 Hz), 139.74, 135.62, 135.14, 132.99, 132.29, 131.88, 129.37, 127.51, 125.37, 117.52, 116.81, 113.90 (t, *J* = 257.9 Hz), 56.39 (t, *J* = 28.3 Hz), 21.27, 18.32.; **HRMS (ESI +)**: calculated for C_19_H_15_F_2_N_3_NaO_2_ [M+Na]^+^, 378.1025, found 378.1023 Δ 0.5 ppm; **LC‐MS (ESI+)** m/z found [M+]^+^, calculated for; **Purity by LC‐MS** (254 nm): >99%

#### 4.2.18 General Synthetic Procedure for Pyrazolidine Derivates 10a and 10b

To a suspension of K_2_CO_3_ (2.0 eq) and **8a** or **8b** (1.0 eq) in DMF (44‐45 mM) at r.t. was added a solution of 1‐(bromomethyl)‐3‐methoxybenzene (2.0 eq) in DMF (44‐45 mM). The reaction mixture was stirred at r.t. until completion (monitored by TLC) was reached. Sat. aq. NaCl and EtOAc were added, the layers were separated and the organic layer was washed with sat. aq. NaCl three times. The organic extract was dried over MgSO_4_ or Na_2_SO_4_, filtered, and concentrated under reduced pressure. Purification by flash column chromatography (SiO_2_, EtOAc in hexanes) afforded compounds **10a** and **10b**.

#### 4.2.19 2‐(2,4‐Dimethylphenyl)‐1‐(3‐Methoxybenzyl)pyrazolidin‐3‐One (10a)

^**1**^ **H NMR** (400 MHz, CDCl_3_) δ 7.30–7.25 (m, 1H), 7.21 (ddt, *J* = 8.4, 7.0, 1.3 Hz, 1H), 7.11–7.07 (m, 1H), 7.05 (dd, *J* = 8.0, 2.1 Hz, 1H), 6.87–6.83 (m, 1H), 6.80 (dd, *J* = 7.3, 1.1 Hz, 2H), 3.82 (bs, 2H), 3.78 (s, 3H), 3.34 (bs, 2H), 2.73 (bs, 2H), 2.33 (d, *J* = 1.4 Hz, 6H); ^**13**^ **C NMR** (101 MHz, CDCl_3_) δ 170.87, 159.80, 138.19, 138.11, 135.86, 132.30, 132.02, 127.56, 127.29, 121.39, 114.84, 112.99, 59.52, 55.31, 48.42, 30.27, 21.21, 18.42.; **HRMS (ESI+)**: calculated for C_19_H_23_N_2_O_2_ [M+H]^+^, 311.1754, found 311.1752 Δ 0.8 ppm; **LCMS (ESI+):** m/z found 310.8 [M+H]^+^, calculated for C_19_H_23_N_2_O_3_; **Purity by LC‐MS** (254 nm): >99%

#### 4.2.20 2‐(2,4‐Dimethylphenyl)−4,4‐Difluoro‐1‐(3‐Methoxybenzyl)pyrazolidin‐3‐One (10b)

^**1**^ **H NMR** (500 MHz, CDCl_3_) δ 7.21 (t, *J* = 7.9 Hz, 1H), 7.16 (d, *J* = 8.0 Hz, 1H), 7.06–6.99 (m, 2H), 6.86 (dd, *J* = 8.2, 2.5 Hz, 1H), 6.67 (d, *J* = 7.5 Hz, 1H), 6.60 (d, *J* = 2.1 Hz, 1H), 5.08 (s, 2H), 4.46 (t, *J* = 12.7 Hz, 2H), 3.76 (s, 3H), 2.33 (s, 3H), 2.18 (s, 3H); ^**19**^ **F NMR** (471 MHz, CDCl_3_) δ −115.10; ^**13**^ **C NMR** (126 MHz, CDCl_3_) δ 159.89, 159.79 (t, *J* = 30.7 Hz), 142.69, 139.53, 134.89, 132.23, 132.06, 129.65, 127.56, 126.25, 119.18, 116.75, 113.30, 112.31 (t, *J* = 254.9 Hz), 65.21, 55.30, 51.32 (t, *J* = 25.1 Hz), 21.19, 17.87.**HRMS (ESI +)**: calculated for C_19_H_21_F_2_N_2_O_2_ [M+H]^+^, 347.1566, found 347.1566 Δ 0.1 ppm; **LC‐MS (ESI+)** m/z found 347.0 [M+H]^+^, calculated for C_19_H_21_F_2_N_2_O_2_; **Purity by LC‐MS** (254 nm): >99%

#### 4.2.21 2‐(2,4‐Dimethylphenyl)‐1‐(3‐Fluorobicyclo [1.1.1]pentane‐1‐Carbonyl)pyrazolidin‐3‐One (14a)

To a solution of 3‐fluorobicyclo [1.1.1]pentane‐1‐carboxylic acid (25.6 mg, 0.20 mmol, 1.5 eq) and HATU (124.9 mg, 0.33 mmol, 2.5 eq) in DMF (1.0 mL) was added DIPEA (114.0 μL). The reaction mixture was stirred for 30 min at r.t. A solution of **8a** (25.0 mg, 0.13 mmol, 1.0 eq) in DMF (1.0 mL) was added dropwise. Upon completion as monitored by TLC (50 % EtOAc in hexanes) after 1 h, EtOAc (20 mL) and 10 % aq. citric acid (10 mL) and sat. aq. NaCl (10 mL) were added. The layers were separated and the aqueous layer was extracted with EtOAc (3x 15 mL). The combined organic extracts were dried over Na_2_SO_4_, filtered and concentrated under reduced pressure. Purification by flash column chromatography (SiO_2_, 50 % EtOAc in hexanes) afforded the title compound **14a** (16.8 mg, 0.055 mmol, 42 %) as a light‐yellow solid.

^**1**^ **H NMR** (400 MHz, CDCl_3_) δ 7.12–7.04 (m, 1H), 6.99 (dd, *J* = 8.1, 2.0 Hz, 0H), 4.25 (t, *J* = 7.9 Hz, 1H), 2.85 (t, *J* = 7.9 Hz, 1H), 2.40 (d, *J* = 2.4 Hz, 3H), 2.29 (s, 3H); ^**19**^ **F NMR** (376 MHz, CDCl_3_) δ −149.07; ^**13**^ **C NMR** (101 MHz, CDCl_3_) δ 169.51, 166.92 (d, *J* = 32.1 Hz), 138.30, 134.85, 133.99, 132.03, 127.28, 124.99, 75.29 (d, *J* = 328.2 Hz), 56.18 (d, *J* = 21.7 Hz), 47.22, 32.17, 29.10 (d, *J* = 45.3 Hz), 21.23, 18.45; **HRMS (ESI +)**: calculated for C_17_H_19_FN_2_NaO_2_ [M+Na]^+^, 325.1323, found 325.1316 Δ 2.1 ppm; **LC‐MS (ESI+)** m/z found 303.8 [M+H]^+^, calculated for C_17_H_20_FN_2_O_2_; **Purity by LC‐MS** (254 nm): >99%

#### 4.2.22 Synthesis of Precursor 19

To a solution of **18** (75.0 mg, 0.177 mmol, 1.0 eq) in THF (9.5 mL) at ‐78 °C was dropwise added TBAF (1 M in THF, 265 μL, 0.265 mmol, 1.5 eq). The reaction mixture was stirred at ‐78 °C for until completion as monitored by TLC (20 % EtOAc in hexanes) after 30 min. The reaction mixture was allowed to reach r.t. Sat. aq. NaHCO_3_ (20 mL) and EtOAc (20 mL) were added, the layers were separated, and the aqueous layer was extracted with EtOAc (3x 20 mL). The combined organic extracts were dried over Na_2_SO_4_, filtered, and concentrated under reduced pressure. Purification by flash column chromatography (SiO_2_, 40 % EtOAc in hexanes) afforded the title compound **19** (48.1 mg, 0.155 mmol, 88%) as a white solid.

^**1**^ **H NMR** (400 MHz, DMSO) δ 9.79 (s, 1H), 7.34 – 7.25 (m, 2H), 7.12 (dt, *J* = 7.6, 1.2 Hz, 1H), 7.09 – 7.04 (m, 2H), 7.01 (dd, *J* = 8.1, 2.1 Hz, 1H), 6.95 (ddd, *J* = 8.1, 2.5, 1.0 Hz, 1H), 4.24 (t, *J* = 7.7 Hz, 2H), 2.76 (t, *J* = 7.7 Hz, 2H), 2.30 (s, 3H), 2.26 (s, 3H). ^**13**^ **C NMR** (101 MHz, CDCl_3_) δ 170.03, 169.65, 157.42, 136.72, 134.65, 134.55, 134.34, 130.99, 129.73, 126.56, 125.29, 119.03, 118.88, 115.10, 49.97, 31.76, 20.56, 18.15.

**HRMS (ESI +)**: calculated for C_18_H_18_N_2_NaO_3_ [M+Na]^+^, 333.121, found 333.1207Δ 0.7 ppm

#### 4.2.23 Radiosynthesis of 2‐(2,4‐Dimethylphenyl)‐1‐(3‐Methoxybenzoyl)pyrazolidin‐3‐One ([^11^C]9a)

[^11^C]CO_2_ was produced via the ^14^N(p, a)^11^C nuclear reaction by proton bombardment of nitrogen gas containing 0.5% oxygen in a Cyclone 18/9 cyclotron (17‐MeV; IBA, Ottignies‐Louvain‐la‐Neuve, Belgium). [^11^C]CO_2_ was reduced to [^11^C]CH_4_ via a heterogenous nickel‐based catalytic reduction which was subsequently converted to [^11^C]CH_3_I by gas phase iodination. [^11^C]CH_3_I was passed through a pre‐heated column (180 °C) containing silver triflate on graphitized carbon spheres to achieve conversion to [^11^C]CH_3_OTf.

[^11^C]CH_3_OTf was bubbled into a reaction vial containing a solution of precursor **19** (0.8 mg, 2.6 μmol) and aq. NaOH (1 M, 2.5 μL) in MeCN (550.0 μL). The reaction was stirred at 20 °C for 1 min. After dilution with 0.1 % H_3_PO_4_ in water (1.6 mL), the resulting mixture was loaded onto a semi‐preparative HPLC column (ACE 3 C18, mobile phase A: 0.1 % H_3_PO_4_ in water, mobile phase B: 5 % water in MeCN, gradient method: 0.0–5.0 min, 35%–45% B; 5.0–8.0 min, 45%–65 % B; 8.0–12.0 min, 65%–75% B; 12.0–21.0 min, 75%–85%, 21.0–25.0 min, 85%–35% B; flow = 4 mL/min). the collected fraction was diluted with water (10 mL) and passed through a Sep‐Pak C18 Plus Light cartridge (Waters, Massachusetts, USA). The resin was washed with water (5 mL) and the compound was eluted with EtOH (0.5 mL). The radiotracer was formulated with PEG300 (1.0 mL) and PBS (8.5 mL), resulting in a neutral solution (pH = 7.4). Quality control was performed on an analytical Agilent 1100 series HPLC system (Agilent Technologies, California, USA) equipped with UV multiwavelength detector, GabiStar radiodetector (Elysia Raytest, Liège, Belgium), and a reverse phase column (Agilent Zorbax 3.5 μm XDB‐C18; 75x4.6 mm; mobile phase A: 0.1 % H_3_PO_4_ in water, mobile phase B: 5 % water in MeCN, gradient method:0.0‐5.0 min, 10 % B, 5.0–7.0 min, 10‐85 % B, 7.0–10.0 min, 85‐95 % B, 10.0–12.0 min, 95–10 % B; flow = 1 mL/min, UV detection at 230 nm and 254 nm). Molar activities were calculated based on UV intensity of **[** ^**11**^ **C]9a** and a linear calibration curve obtained by linear regression of the UV intensity of standard solutions of non‐radioactive reference compound **9a**.

### 4.3 Molecular Docking

The protein structures of the F1 ATPase respectively inhibited by small molecule ligands quercetin (PDB ID: 2JJ2), resveratrol (PDB ID: 2JIZ), and piceatannol (PDB ID: 2JJ1) [7] were downloaded from the RSCB protein databank.

The protein structures were prepared using MOE v.2024.0601 [34] QuickPrep module with the following settings: Preserve Sequence and Neutralize; Use Protonate 3D for Protonation = True; Allow ASN/GLN/HIS “Flips” in Protonate 3D = True; Delete Water Molecules Farther than 4.5 Å from Ligand or Receptor = True, Tether Receptor: Strength = 10, Buffer = 0.25; Fix: Atoms Farther than 8 Å from Ligands, Hydrogens close to Binding Site will not be fixed; Refine: To RMS Gradient of 0.1 kcal/mol/Å^2^; Retain QuickPrep Minimization Restraints = True.

The ligand molecules were prepared using Protonate3D and the Compute ‐ Wash module with the following settings: Fragments: Disconnect Group I Metals in Simple Salts = True; Keep Only Largest Molecular Fragment = True; Protonation: Dominant; pH = 7; Hydrogens: Add Explicit; Coordinates: Rebuild 3D; Scale to Reasonable Bond Lengths = True.

For the consensus docking, the prepared ligand molecules (quercetin, CJ1‐34, 9a, 10a, 11b, 12b and caffeine) were docked in MOE using GOLD v2022.2.0 with the following settings: Receptor: Receptor and Solvent Atoms; Site: Ligand Atoms (quercetin); Efficiency: Default 100; Early Termination Number: 3, RMS: 1.5; Diverse Poses During Placement = True; Ligand: COOH: rotate; Flip: Ring Corners = True, Planar N = True, Pyramidal N = True; Scoring: GoldScore/ChemScore/ASP; Refinement: Rigid Receptor; Placement: 40 poses/compound, Refinement: 10 poses/compound with GBVI/WSA dG. For MOE Docking the following settings were chosen Receptor: Receptor and Solvent Atoms; Site: Ligand Atoms (quercetin); Placement: Triangle Matcher, Refinement: Rigid Receptor; Placement: 40 poses/compound, Refinement: 10 poses/compound with GBVI/WSA dG.

AutoDock Vina [35] docking was performed within PyMOL v3.1.7.2 (Schrodinger, LLC [36]) using the DockingPie 1.2 [37] plugin. The protein structure was exported from MOE and loaded in PyMOL. Ligands were converted and protonated via Openbabel [38] (smiles to sdf) and loaded in PyMOL. Receptor and Ligand atoms were prepared within DockingPie, and Grid Setting were chosen around quercetin (Grid Dimension: All: 4, X:4, Y:4, Z:4, Grid Center Coordinates: Z: −7.26, Y: 25.49, Z9.46; Docking: AllvsAll, Poses: 10, Exhaustivness: 40, Energy Range 3).

Rank by rank scoring was carried out by assigning rank positions (best scoring pose) to each ligand for every scoring function and computing the mean aggregated rank. This average rank reflects how consistently a ligand is prioritized across scoring functions.

To assess the different poses the MOE SVL script for clustering was used. Each ligand (10 poses per method) was clustered by conformation (heavy atoms) using a hierarchical modeling method with average linkage with a cutplane of 2 Å. The clusters were then analyzed by number of poses as well as number of different docking methods / cluster.

For the induced fit docking of all prepared ligands, the MOE suite internal function was used with the following settings: Receptor: Receptor and Solvent Atoms; Site: Ligand Atoms (quercetin); Placement: Triangle Matcher, Refinement: Rigid Receptor; Placement: 30 poses/compound, and Refinement: five poses/compound with GBVI/WSA dG [39], which is a scoring function that estimated free binding energies based on protein–ligand hydrogen bond energy, protein‐ligand van der Waals energy, ligand internal van der Waals energy, and ligand torsional strain energy.

### 4.4 *In Vitro* Evaluation

#### 4.4.1 ATP Hydrolysis in Isolated Mitochondria

This assay procedure was adapted from Serdiuk et al. [40]. A detailed description of the mouse liver mitochondria isolation and assay procedure can be found in the supplementary information. In brief, an aliquot of isolated liver mitochondria was diluted with assay buffer (25 mM HEPES, 25 mM KCl, 2 mM MgCl_2_ hexahydrate, pH 7.5‐8.0 adjusted using KOH) to a concentration of 5 mg/mL, frozen in liquid nitrogen for 3 min, and thawed in a thermomixer at 37 °C. The freezing and thawing cycle was repeated four times in total. The mitochondrial suspension was further diluted with assay buffer to a concentration of 1.32 mg/mL and kept on ice. Reaction mix (2.84 mM KCl, 1.42 mM MgCl_2_, 1.42 U/mL PK, 1.95 U/mL LDH, 0.85 μM FCCP, 0.26 μM antimycin A, 1.13 mM PEP, 638.12 μM NADH) in nanopure water was freshly prepared on ice. In a 96‐well plate, reaction mix (85 μL) and mitochondria suspension (25 μL) were added to each well. Compounds were diluted with DMSO to the desired concentration. A 1 μL of diluted compound was added in triplicates achieving a final concentration of 100 nM in the well. The reaction was started by adding 2 μL ATP to each well. immediately, the plate was placed into a preheated microplate reader at 37 °C and the absorbance was monitored at 340 nm for 30 min.

#### 4.4.2 ATP Production in HT‐22 Cells

This assay procedure was adapted from Serdiuk et al. [40]. Detailed description of cell‐line maintenance, assay procedure can be found in the Supplementary Information. In brief, HT‐22 cells were counted suspended in DMEM and transferred into a 96‐well culture plate. The plated cells were incubated for 24 h at 37 °C and 5 % CO_2_. Compound solutions were prepared from stock solutions in DMSO by diluting with DMEM with a final concentration of 1 μM (<0.1 % DMSO) in the cell plate. A vehicle sample (<0.1 % DMSO in DMEM) was used as reference and oligomycin A (5 μM in DMEM, <0.1 % DMSO) was used as a positive control. Each experiment was conducted in triplicates. Compound and vehicle‐treated cells were incubated for 15 min. In a second well plate, ATP standards for a calibration curve (0, 1, 2, 3, 5, 10, and 15 μM in PBS) were prepared, 25 μL of Cell titer‐Glo buffer was added to each ATP standard well, and the plate was shaken at 130 rpm for 15 min. The cell plate with the incubated compounds was washed with 100 μL PBS, followed by adding 50 μL of PBS and 25 μL of cell‐titer Glo buffer for each well. The plate was then shaken at 130 rpm for 15 min to lyse the cells. For protein content determination via BCA protein determination assay, 10 μL aliquots from each well were pipetted from all ATP standard samples and all test samples into a clear 96‐wellplate. To the remaining sample volume, 2xcell titer‐Glo reagents (25 μL per well) were added and the plates were incubated in the dark for 30 min under light shaking. Subsequently, luminescence of ATP standard samples and test samples was measured at 530/25 nm using a microplate reader.

## 5 Statistical Analysis

Data analysis was performed using Prism Software (Graphpad, version 10.4.1). One‐way ANOVA was conducted followed by Dunnett's multiple comparisons test to determine statistical significance between vehicle control and test compounds in all in vitro evaluations. *p* < 0.05 was considered significant.

## 6 Plasma Stability Evaluation

**[** ^**11**^ **C]9a** in formulation (10.0 μL, ∼8 MBq) was added to mouse plasma (300 μL) and incubated at 37 °C under light shaking. For each timepoint (5, 30, 60 min), 100 μL of the mixture was added to 100 μL ice‐cold MeCN. The samples were centrifuged (r.t., 5 min), and the supernatant was added to 100 μL ice‐cold MeCN to ensure complete protein precipitation. Centrifugation was repeated, and the supernatant was diluted with water (300 μL), passed through a syringe filter (Nalgene, 0.22 μM, PES membrane). A reference sample was prepared by adding **[** ^**11**^ **C]9a** in formulation (10.0 μL, ∼8 MBq) to PBS (300 μL) and incubating (37 °C, light shaking) for 60 min. All samples were analyzed via UPLC equipped with a a reverse phase column (Waters Acquity, C18, 2.1x10.0 mm, mobile phase A: 0,1 % TFA in water, mobile phase B: 5% water in MeCN, gradient method: 0–0.5 min, 35 % B, 0,5–5 min 35%–95% B; flow = 1 mL/min).

## 7 Free Fraction Determination

**[** ^**11**^ **C]9a** in formulation (7.0 μL, ∼3 MBq) was added to mouse plasma (150 μL) and incubated at 37 °C for 10 min under light shaking. The mixture was subsequently transferred to a centrifugal filter unit (MerckMillipore, UFC501096) with 300 μL ice‐cold PBS and briefly vortexed. The sample was centrifuged (15000 g, 4 °C, 10 min). The filter was subsequently washed by adding 100 μL ice‐cold PBS and centrifugation (15000 g, 4 °C, 10 min). This washing step was repeated 3 times in total, and the radioactivity of the collected filtrate was measured by an automatic γ‐counter (Wizard, PerkinElmer). The filteres were inverted and centrifuged (1000 g, 4 °C, 2 min) to obtain the plasma protein fraction (A_protein_). Both filters and plasma protein fraction were measured by the γ‐counter. The free fraction was calculated according to $1-\frac{A_{\text{protein}}}{A_{\text{total}}}$. The experiment was carried out in triplicate.

## Funding

This work was supported by the Swiss National Fundation (Grant 205321_192409/1) and the Novartis Stiftung für Medizinisch‐Biologische Forschung (Grant #22A047).

## Conflicts of Interest

The authors declare no conflicts of interest.

## Supporting information

The experimental procedures and characterization data (^1^H‐ and ^19^F‐NMR spectra and LC‐MS) of all compounds and further data on the crystal structures for **4d**, **5b** and **9a** can be found in the supporting information. Deposition Numbers CCDC‐2564047 (**4d**), CCDC‐2521080 (**5b**) and CCDC‐2564048 (**9a**) contain the supplementary crystallographic data for this paper, including structure factors and refinement instructions, and can be obtained free of charge from The Cambridge Crystallographic Data Centre, 12 Union Road, Cambridge CB2 1EZ, UK via https://www.ccdc.cam.ac.uk/getstructures. The authors have cited additional references within the Supporting Information.

## Data Availability

The data that support the findings of this study are available in the supplementary material of this article.
