# Supplementary material for: Identification of Pyrazolidine‐3‐One Derivatives as a Novel Structural Scaffold for ATP Synthase Inhibitors
Source: ChemMedChem. 2026 Jul 27;21(14):e70399. doi: 10.1002/cmdc.70399 (PMC13407218; doi:10.1002/cmdc.70399)
Supplement: Supplementary file 1 — The experimental procedures and characterization data (1H‐ and 19F‐NMR spectra and LC‐MS) of all compounds and further data on the crystal structures for 4d, 5b and 9a can be found in the supporting information. Deposition Numbers CCDC‐2564047 (4d), CCDC‐2521080 (5b) and CCDC‐2564048 (9a) contain the supplementary crystallographic data for this paper, including structure factors and refinement instructions, and can be obtained free of charge from The Cambridge Crystallographic Data Centre, 12 Union Road, Cambridge CB2 1EZ, UK via https://www.ccdc.cam.ac.uk/getstructures. The authors have cited additional references within the Supporting Information. [file CMDC-21-e70399-s001.pdf]

Supporting Information

Identification of Pyrazolidine-3-one Derivatives as a novel Structural Scaffold for ATP Synthase Inhibitors

Lisa Reichert[a], Aro Delparente[a], Ida R. Hipfinger[a], Lukas Reininger[a], Ana Pinto Castro[a], Daniel J. Hubin[a], Roger Schibli[a], Amy E. Fraley[a], Linjing Mu[a]*

[a] L. Reichert, A. Delparente, I. R. Hipfinger, Lukas Reininger, Ana Pinto Castro, D. J. Hubin, Prof. Dr. R. Schibli, Prof. Dr. A. E. Fraley, Dr. L. Mu
Institute of Pharmaceutical Sciences
Department of Chemistry and Applied Biosciences
ETH Zürich
CH-8093 Zurich, Switzerland
E-mail: linjing.mu@pharma.ethz.ch

General Information

Unless otherwise stated, all reactions were carried out under inert atmosphere in commercial anhydrous solvents (Sigma Aldrich GmbH). Reagents were purchased from Sigma Aldrich GmbH (Buchs, Switzerland), ABCR GmbH (Karlsruhe, Germany), Fluorocham Ltd. (Hadfield, UK), Tokyo Chemical Industry Co. Ltd (Tokyo, Japan), Apollo Scientific Ltd (Cheshire, UK), Acros Organics (Geel, Belgium), Thermo Fisher Scientific (Hampton, USA), BLD Pharmatech Ltd (shanghai, China). Solvents for flash column chromatography, thin layer chromatography (TLC) and liquid extractions were purchased as commercial grade. TLC was conducted using silica gel 60 plates (Merck) under UV light (254 nm) and/or by staining with potassium permanganate ($KMnO_4$). Flash column chromatography was performed using silica gel (60 Å pore size, 230-400 mesh particle size, Sigma Aldrich GmbH). Nuclear magnetic resonance (NMR) spectra (^{1}H, ^{13}C, and if applicable ^{19}F) of compounds were recorded on either a Bruker 400 MHz spectrometer or a Bruker 500 MHz spectrometer at r.t. (298 K). NMR spectra were processed and analyzed using MestreNova (Version 14.2.3, Mestrelab Research S. L.). Chemical shifts (δ) are reported in parts per million (ppm) relative to trimethylsilane (0 ppm). Values of the coupling constant (*J*) are given in hertz (Hz). Multiplicities in the ^{1}H NMR spectra are described as: singlet (s), doublet (d), triplet (t), quartet (q), multiplet (m), doublet of doublet (dd), triplet of doublet (td), and broad peak (br). The chemical shifts of complex multiplets are given as the range of their occurrence. High resolution mass spectrometry (HRMS) was performed by the Molecular and Biomolecular Analysis Service (MoBiAS) using a Bruker MaXis with ESI ion source and Qq-TOF analyser (ESI−Qq-TOF-MS) in positive mode and processed by the software Compass 1.5, HyStar 3.2.44, DataAnalysis 4.1 and BioTools 3.3. HRMS data are reported in m/z. Liquid chromatography mass spectrometry (LC-MS) and purity was measured on an Agilent InfinityLab LC/MSD XT using an analytical reverse phase column (Waters BEH C18, 1.10x50.0 mm, 1.70 µm) with H_2O/MeCN (both containing 0.1 % formic acid) as mobile phase. Method: 2 % MeCN from 0-1 min, gradient from 2 % to 98 % MeCN from 1-8 min, 98 % MeCN from 8-9 min, 0.600 mL/min, 1200.0 bar.

Screening of reaction conditions – General procedure.

Diazene **4b** (22.8 mg, 0.1 mmol, 1.0 eq) and freshly activated zinc dust were suspended in the respective solvent. The reaction mixture was warmed to the respective temperature and 2-bromo-2,2-difluoroacetyl chloride was added dropwise. The reaction mixture was stirred for 1 h. Subsequently, the reaction mixture was transferred into an NMR tube and diluted with $CDCl_3$. The noted amount of $CFCl_3$ was added and the samples were measured immediately.

Activated Zinc dust

This procedure was adapted from Yamamura *et al.*[1] Zinc dust (1.5 g, 22.94 mmol) was suspended in 2 % aq. HCl (10 mL) and stirred vigorously for 30 min. The mixture was filtered and the residue was washed with water (3x 20 mL), then EtOH (5 mL), acetone (10 mL) and diethylether (5 mL) in this order. The residue was subsequently dried under vacuum at 85 °C for 15 min. The product was used within one day of activation.

(E)-phenyl(phenyldiazenyl)methanone (4a)

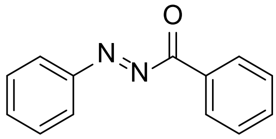

To a solution of phenylhydrazine (1.0 mL, 10.15 mmol, 1.0 eq) in 2,6-lutidine (20.0 mL) at -18 °C was added benzoyl chloride (1.30 mL, 11.17 mmol, 1.1 eq). The reaction mixture was stirred at -18 °C for 1 h and subsequently allowed to reach r.t. until completion as monitored by TLC (30 % EtOAc in hexanes) was reached after 15h. 1 M HCl (250 mL) and CH_2Cl_2 (100 mL) were added, the layers were separated and the aqueous layer was extracted with CH_2Cl_2 (3x 50 mL). the combined organic extracts were dried over Na_2SO_4, filtered and concentrated under reduced pressure. The residue was taken up in CH_2Cl_2 (15.2 mL) and Mn(IV)O_2 (activated, 3.115 g, 30.46 mmol, 3.0 eq) was added in portions. The reaction mixture was stirred at r.t. until completion as monitored by TLC (10 % EtOAc in hexanes) after 3 h. The reaction mixture was filtered over a pad of celite and the residue was washed with copious amounts of CH_2Cl_2. The filtrate was concentrated under reduced pressure. Purification by flash column chromatography (SiO_2, 5 % EtOAc in hexane) afforded the title compound **4a** (1.899 g, 9.03 mmol, 89 %) as a dark red oil. The characterization data of the compound is in accordance with reported spectra.[2]

^{1}H NMR (500 MHz, $CDCl_3$) δ 8.10 – 8.04 (m, 2H), 8.03 – 7.98 (m, 2H), 7.67 (ddt, *J* = 7.9, 7.1, 1.3 Hz, 1H), 7.64 – 7.50 (m, 5H).

HRMS (ESI+): calculated for $C_{13}H_{10}N_2NaO$ $[M+Na]^+$, 233.0685, found 233.0682 Δ 1.4 ppm

(E)-(4-fluorophenyl)(phenyldiazenyl)methanone (4b)

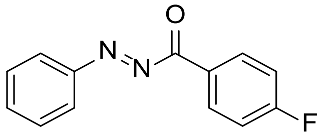

To a solution of phenylhydrazine (1.0 mL, 10.15 mmol, 1.0 eq) in 2,6-lutidine (20.0 mL) at -18 °C was added 4-fluorobenzoyl chloride (1.32 mL, 11.17 mmol, 1.1 eq). The reaction mixture was stirred at -18 °C for 1 h and subsequently allowed to reach r.t. until completion as monitored by TLC (30 % EtOAc in hexanes) after 15 h. 1 M HCl (250 mL) and CH_2Cl_2 (100 mL) were added, the layers were separated and the aqueous layer was extracted with CH_2Cl_2 (3x 50 mL). the combined organic extracts were dried over Na_2SO_4, filtered and concentrated under reduced pressure. The residue was taken up in CH_2Cl_2 (15.2 mL) and Mn(IV)O_2 (activated, 3.115 g, 30.46 mmol, 3.0 eq) was added in portions. The reaction mixture was stirred at r.t. until completion as monitored by TLC (10 % EtOAc in hexanes) after 3 h. The reaction mixture was filtered over a pad of celite and the residue was washed with copious amounts of CH_2Cl_2. The filtrate was concentrated under reduced pressure. Purification by flash column chromatography (SiO_2, 5 % EtOAc in hexane) afforded the title compound **4b** (1.832 g, 8.03 mmol, 79 %) as a dark red solid. The characterization data of the compound is in accordance with reported spectra.[3]

^{1}H NMR (500 MHz, $CDCl_3$) δ 8.15 – 8.09 (m, 2H), 8.00 (dt, *J* = 7.0, 1.5 Hz, 2H), 7.64 – 7.55 (m, 3H), 7.21 (t, *J* = 8.6 Hz, 2H).

^{19}F NMR (471 MHz, $CDCl_3$) δ -101.96.

HRMS (ESI+): calculated for $C_{13}H_9FN_2NaO$ $[M+Na]^+$, 251.0591, found 251.0589 Δ 0.8 ppm

(E)-(4-methoxyphenyl)(phenyldiazenyl)methanone (4c)

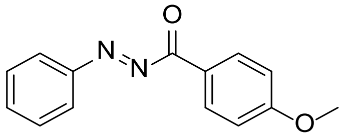

To a solution of phenylhydrazine (1.0 mL, 10.15 mmol, 1.0 eq) in 2,6-lutidine (20.0 mL) at -18 °C was added 4-methoxybenzoyl chloride (1.51 mL, 11.17 mmol, 1.1 eq). The reaction mixture was stirred at -18 °C for 1 h and subsequently allowed to reach r.t. until completion as monitored by TLC (30 % EtOAc in hexanes) after 15 h. 1 M HCl (250 mL) and CH_2Cl_2 (100 mL) were added, the layers were separated and the aqueous layer was extracted with CH_2Cl_2 (3x 50 mL). the combined organic extracts were dried over Na_2SO_4, filtered and concentrated under reduced pressure. The residue was taken up

in CH_2Cl_2 (15.2 mL) and Mn(IV)O_2 (activated, 3.115 g, 30.46 mmol, 3.0 eq) was added in portions. The reaction mixture was stirred at r.t. until completion as monitored by TLC (10 % EtOAc in hexanes) after 3 h. The reaction mixture was filtered over a pad of celite and the residue was washed with copious amounts of CH_2Cl_2. The filtrate was concentrated under reduced pressure. Purification by flash column chromatography (SiO_2, 5 % EtOAc in hexane) afforded the title compound **4c** (1.832 g, 8.03 mmol, 79 %) as a dark red solid.
^{1}H NMR (500 MHz, $CDCl_3$) δ 8.03 – 7.98 (m, 2H), 7.64 – 7.55 (m, 5H), 7.44 – 7.39 (m, 1H), 7.21 (ddd, *J* = 8.3, 2.7, 1.1 Hz, 1H), 3.88 (s, 3H).
HRMS (ESI+): calculated for $C_{14}H_{12}N_2NaO_2$ $[M+Na]^+$, 263.0791, found 263.0786 Δ 1.8 ppm

(E)-((2,4-dimethylphenyl)diazenyl)(3-methoxyphenyl)methanone (4d)

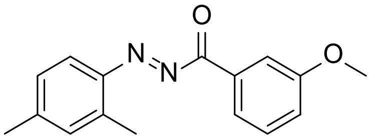

To a suspension of 2,4-dimethylpheyl hydrazine hydrochloride (1.0 g, 5.79 mmol, 1.0 eq.) in 2,6-lutidine (20 mL) at -18 °C was added 3-methoxybenzoyl chloride (0.90 mL, 6.37 mmol, 1.1 eq.). The reaction mixture was stirred at -18 °C for 1 h and subsequently allowed to reach r.t. until completion as monitored by TLC (30 % EtOAc in hexanes) after 15 h. 1 M HCl (250 mL) and CH_2Cl_2 (100 mL) were added, the layers were separated and the aqueous layer was extracted with CH_2Cl_2 (3x 50 mL). the combined organic extracts were dried over Na_2SO_4, filtered and concentrated under reduced pressure. The residue was taken up in CH_2Cl_2 (8.7 mL) and Mn(IV)O_2 (activated, 1.51 g, 17.38 mmol, 3.0 eq.) was added in portions. The reaction mixture was stirred at r.t. until completion as monitored by TLC (10 % EtOAc in hexanes) after 3 h. The reaction mixture was filtered over a pad of celite and the residue was washed with copious amounts of CH_2Cl_2. The filtrate was concentrated under reduced pressure. Purification by flash column chromatography (SiO_2, 5 % EtOAc in hexane) afforded the title compound **4e** (0.702 g, 2.62 mmol, 45 %) as a dark red solid.
^{1}H NMR (400 MHz, $CDCl_3$) δ 7.63 – 7.57 (m, 3H), 7.40 (t, *J* = 7.9 Hz, 1H), 7.21 – 7.18 (m, 2H), 7.10 – 7.08 (m, 1H), 3.87 (s, 3H), 2.68 (s, 3H), 2.41 (s, 3H).
^{13}C NMR (101 MHz, $CDCl_3$) δ 182.53, 160.00, 148.71, 144.56, 140.86, 132.64, 132.41, 129.90, 127.47, 123.45, 121.17, 115.37, 114.42, 55.65, 21.74, 17.58.
HRMS (ESI+): calculated for $C_{16}H_{16}N_2NaO_2$ $[M+Na]^+$, 291.1104, found 291.11 Δ 1.3 ppm

(E)-((2,4-dimethylphenyl)diazenyl)(4-fluorophenyl)methanone (4e)

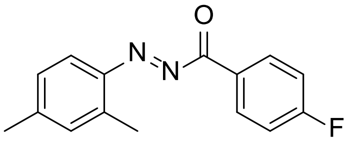

To a suspension of 2,4-dimethylpheyl hydrazine hydrochloride (1.0 g, 5.79 mmol, 1.0 eq.) in 2,6-lutidine (20 mL) at -18 °C was added 4-fluorobenzoyl chloride (0.754 mL, 6.37 mmol, 1.1 eq.). The reaction mixture was stirred at -18 °C for 1 h and subsequently allowed to reach r.t. until completion as monitored by TLC (30 % EtOAc in hexanes) after 15 h. 1 M HCl (250 mL) and CH_2Cl_2 (100 mL) were added, the layers were separated and the aqueous layer was extracted with CH_2Cl_2 (3x 50 mL). the combined organic extracts were dried over Na_2SO_4, filtered and concentrated under reduced pressure. The residue was taken up in CH_2Cl_2 (8.7 mL) and Mn(IV)O_2 (activated, 1.51 g, 17.38 mmol, 3.0 eq.) was added in portions. The reaction mixture was stirred at r.t. until completion as monitored by TLC (10 % EtOAc in hexanes) after 3 h. The reaction mixture was filtered over a pad of celite and the residue was washed with copious amounts of CH_2Cl_2. The filtrate was concentrated under reduced pressure. Purification by flash column chromatography (SiO_2, 5 % EtOAc in hexane) afforded the title compound **4d** (1.133 g, 4.42 mmol, 76 %) as a dark red solid.
^{1}H NMR (400 MHz, $CDCl_3$) δ 8.15 – 8.06 (m, 2H), 7.59 (d, *J* = 8.3 Hz, 1H), 7.24 – 7.15 (m, 3H), 7.12 – 7.06 (m, 1H), 2.67 (s, 3H), 2.41 (s, 3H).
^{19}F NMR (376 MHz, $CDCl_3$) δ -102.56 – -102.65 (m, 1F).
^{13}C NMR (101 MHz, $CDCl_3$) δ 181.09, 166.57 (d, *J* = 256.8 Hz), 148.70, 144.83, 141.09, 133.45 (d, *J* = 9.3 Hz), 132.48, 127.92 (d, *J* = 3.0 Hz), 127.50, 116.25 (d, *J* = 22.1 Hz), 115.30, 21.76, 17.58.
HRMS (ESI+): calculated for $C_{15}H_{14}FN_2O$ $[M+H]^+$, 257.1085, found 257.1082 Δ 1.2 ppm

Synthesis of pyrazolidin-3-ones

tert-butyl 2-(3-chloropropanoyl)-2-(2,4-dimethylphenyl)hydrazine-1-carboxylate (6a)

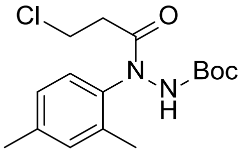

To a suspension of tert-butyl 2-(2,4-dimethylphenyl)hydrazine-1-carboxylate (684 mg, 2.9 mmol, 1.0 eq) and $NaHCO_3$ (243 mg, 2.9 mmol, 1.0 eq) in toluene (17.0 mL) was dropwise added a solution of 3-chloropropionylchloride (0.28 mL, 2.9 mmol, 1.0 eq) in toluene (2.0 mL). The reaction mixture was heated to 80 °C under reflux. Upon completion after 2 h as monitored by TLC (30 % EtOAc in hexanes), the reaction mixture was allowed to reach r.t.. Water (20 mL) was added, the layers were separated and the aqueous layer was extracted with EtOAc (3x 20 mL). The combined organic extracts were dried over Na_2SO_4, filtered and concentrated under reduced pressure. Purification by flash column chromatography (SiO_2, using a gradient of 10 to 30 % EtOAc in hexanes) afforded the title compound **6a** (946.1 mg, 2.518 mmol, 87 %) as a colorless solid.
^{1}H NMR (500 MHz, $CDCl_3$) Rotameric ratio maj:min = 0.78:0.22; δ 7.39 (d, *J* = 8.0 Hz, 1H), 7.13 – 6.99 (m, 2H), 6.83 (s, 1H), 3.87 (s, 1H, min. rot.), 3.82 (m, 1H), 3.68 (m, 1H), 3.03 (bs, 1H, maj. rot), 2.64 (m, 1H), 2.42 (m, 1H), 2.35 (s, 3H, maj. rot.), 2.31 (s, 3H, min. rot.), 2.24 (s, 3H, maj. rot.), 2.21 (s, 3H, min. rot.), 1.47 (s, 9H).
13C NMR (126 MHz, CDCl3) δ 169.90, 155.57, 139.64, 137.39, 135.58, 132.11, 129.28, 128.04, 82.07, 39.38, 36.79, 28.35, 28.30, 21.26, 17.81.
HRMS (ESI +): calculated for $C_{16}H_{23}ClN_2NaO_3$ $[M+Na]^+$, 349.1289, found 349.1291 Δ 0.4 ppm

tert-butyl 2-(2,4-dimethylphenyl)-3-oxopyrazolidine-1-carboxylate (7a)

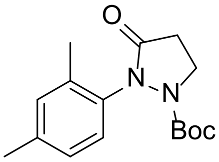

To a suspension of ^{t}BuOK (206.0 mg, 1.84 mmol, 1.2 eq) in THF (10.0 mL) at 0 °C was dropwise added a solution of **6a** (500 mg, 1.53 mmol, 1.0eq) in THF (7.0 mL). The reaction mixture was stirred at 0 °C for 30 min and subsequently allowed to reach r.t.. Upon completion observed by TLC (40 % EtOAc in hexanes) after 1.5 h, the reaction mixture was quenched by addition of sat. aq. NH_4Cl (20 mL). The layers were separated and the aqueous layer was extracted with EtOAc (3x 15 mL). The combined organic extracts were dried over Na_2SO_4, filtered and concentrated under reduced pressure. Purification by flash column chromatography (SiO_2, 30 % EtOAc in hexanes) afforded the title compound **7a** (170.6 mg, 0.59 mmol, 38 %) as a colorless solid.
^{1}H NMR (500 MHz, $CDCl_3$) δ 7.16 (d, *J* = 8.0 Hz, 1H), 7.05 – 6.96 (m, 2H), 4.22 (t, *J* = 7.8 Hz, 2H), 2.76 (t, *J* = 7.8 Hz, 2H), 2.30 (s, 3H), 2.27 (s, 3H), 1.29 (s, 9H).
^{13}C NMR (126 MHz, $CDCl_3$) δ 170.17, 156.57, 137.47, 134.90, 134.36, 131.65, 126.94, 125.14, 82.89, 47.45, 32.02, 28.05, 21.18, 18.60.
HRMS (ESI +): calculated for $C_{16}H_{22}N_2NaO_3$ $[M+Na]^+$, 313.1523, found 313.1519Δ 1.1 ppm

2-(2,4-dimethylphenyl)pyrazolidin-3-one (8a)

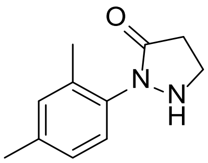

To a solution of **7a** (100.0 mg, 0.34 mmol, 1.0 eq) in CH_2Cl_2 (2.5 mL) was added TFA (0.86 mL. 11.21 mmol, 32.5 eq). The reaction mixture was stirred at r.t.. Upon completion as monitored by TLC (50 % EtOAc in hexanes) after 1 h, sat. aq. NaCl (10 mL) was added. The layers were separated, and the aqueous layer was extracted with EtOAc (4x 10 mL). The combined organic extracts were dried over Na_2SO_4, filtered and concentrated under reduced pressure. Purification by flash column chromatography (SiO_2, 1-2 % MeOH in $CHCl_3$) afforded the title compound **8a** (88.8 mg, 0.47 mmol, 90 %) as an orange solid.

^{1}H NMR (400 MHz, $CDCl_3$) δ 7.15 (d, *J* = 8.0 Hz, 1H), 7.08 – 7.05 (m, 1H), 7.02 (m, 1H), 3.56 (t, *J* = 7.7 Hz, 2H), 3.36 (bs, 1H), 2.74 (t, *J* = 7.7 Hz, 2H), 2.31 (s, 3H), 2.25 (s, 3H).
^{13}C NMR (101 MHz,$CDCl_3$): δ 171.52, 139.76, 136.01, 132.15, 131.95, 127.73, 127.14, 43.76, 32.40, 21.26, 17.91.
HRMS (ESI+): calculated for $C_{11}H_{15}N_2O$ $[M+H]^+$, 191.1179, found 191.1179 Δ 0 ppm

tert-butyl 2-(3-bromo-2,2-difluoropropanoyl)-2-(2,4-dimethylphenyl)hydrazine-1-carboxylate (6b)

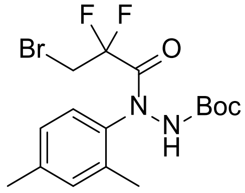

Oxalyl chloride (154 µL, 1.77 mmol, 1.56 eq) was added dropwise to a solution of 3-bromo-2,2-difluoropropanoic acid (278.0 mg, 1.47 mmol, 1.3 eq.) in CH_2Cl_2 (5.4 mL) at 0 °C. A drop of DMF (ca. 5 µL) was added and the reaction mixture was allowed to reach r.t.. After 3 h, volatiles were removed under reduced pressure without heating. The residue was taken up in toluene (5.7 mL) and dropwise added to a suspension of tert-butyl 2-(2,4-dimethylphenyl)hydrazine-1-carboxylate (267.4 mg, 1.13 mmol, 1.0 eq) and $NaHCO_3$ (95.0 mg, 1.13 mmol, 1.0 eq) in toluene (2.0 mL). The reaction mixture was heated to 80 °C and stirred until completion was monitored by TLC (20 % EtOAc in hexane) after 3 h. The reaction mixture was allowed to reach r.t.. Water (10 mL) and EtOAc (10 mL) were added, the layers were separated and the aqueous layer was extracted with EtOAc (3x 10 mL). The combined organic extracts were dried over Na_2SO_4, filtered and concentrated under reduced pressure. Purification by flash column chromatography (SiO_2, 10 % EtOAc in hexane) afforded the title compound **6b** (342.4 mg, 0.841 mmol, 74 %) as a colorless solid.
^{1}H NMR (500 MHz, $CDCl_3$) Rotameric ratio maj:min = 0.54:0.46; δ 7.42 (d, *J* = 7.8 Hz, 1H), 7.31 (bs, 1H), 7.21 (s, 1H), 7.10 – 7.00 (m, 2H), 3.93 (t, *J* = 14.8 Hz, 1H), 3.89 – 3.70 (m, 1H), 2.34 (s, 3H, maj. rot.), 2.32 (s, 3H, min. rot.), 2.25 (s, 3H, maj. rot.), 2.23 (s, 3H, min. rot.), 1.48 (s, 9H, maj. rot.), 1.46 (s, 9H, min. rot.).
^{19}F NMR (471 MHz, $CDCl_3$) δ -97.08, -97.67, -101.78, -102.89, -103.47.
^{13}C NMR (126 MHz, $CDCl_3$) δ 162.23 (t, J=29.1 Hz, 1C), 154.82, 153.95, 139.84, 139.11, 136.96, 136.72, 135.67, 134.61, 131.81, 131.64, 129.15, 127.77, 114.26 (, J=259.62 Hz, 1C, min. rot.), 114.19 (t, J=259,62 Hz, 1C, maj. rot.), 112.14, 82.57, 77.41, 77.36, 77.16, 76.91, 30.14 (t, J=29,1 Hz, 1C), 28.32, 28.18, 21.23, 21.17, 17.72, 17.66.
HRMS (ESI+): calculated for $C_{16}H_{21}BrF_2N_2NaO_3$ $[M+Na]^+$, 429.0596, found 429.0595 Δ 0.2 ppm
LC-MS (ESI+) m/z found 430.5 $[M+Na]^+$, calculated for $C_{16}H_{21}BrF_2N_2NaO_3$; **Purity by LC-MS** (254 nm): >99%

tert-butyl 2-(2,4-dimethylphenyl)-4,4-difluoro-3-oxopyrazolidine-1-carboxylate (7b)

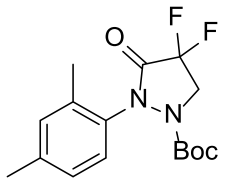

To a suspension of ^{t}BuOK (78.0 mg, 0.70 mmol, 1.2 eq) in THF (5 mL) at 0 °C was dropwise added a solution of **6b** (236.2 mg, 0.58 mmol, 1.0 eq.) in THF (1.7 mL). The reaction mixture was stirred at 0 °C for 30 min and subsequently allowed to reach r.t.. Upon completion as monitored by TLC (20 % EtOAc in hexane) after 1 h, the reaction mixture was concentrated under reduced pressure. Sat. aq. NaCl (20 mL) and EtOAc (20 mL) were added, the layers were separated and the aqueous layer was extracted with EtOAc (3x 10 mL). the combined organic extracts were dried over Na_2SO_4, filtered and concentrated under reduced pressure. Purification by flash column chromatography (SiO_2, 10 % EtOAc in hexane) afforded the title compound **7b** (97.2 mg, 0.30 mmol, 51 %) as a light yellow solid.
^{1}H NMR (400 MHz, $CDCl_3$) δ 7.20 (d, *J* = 8.1 Hz, 1H), 7.10 – 7.00 (m, 2H), 4.40 (t, *J* = 12.9 Hz, 2H), 2.33 (s, 3H), 2.28 (s, 3H), 1.33 (s, 9H).
^{19}F NMR (376 MHz, $CDCl_3$) δ -115.47.
^{13}C NMR (101 MHz, $CDCl_3$) δ 158.60 (t, *J* = 30.0 Hz), 155.90, 138.69, 134.24, 133.29, 131.90, 127.18, 124.70, 114.64 (t, *J* = 256.8 Hz), 84.68, 54.76 (t, *J* = 27.6 Hz), 27.89, 21.21, 18.41.

HRMS (ESI+): calculated for $C_{16}H_{20}F_2N_2NaO_3$ $[M+Na]^+$, 349.1334, found 349.1333 Δ 0.4 ppm

2-(2,4-dimethylphenyl)-4,4-difluoropyrazolidin-3-one (8b)

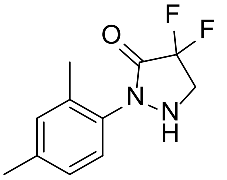

TFA (168 µL, 2.184 mmol, 32.4 eq) was dropwise added to a solution of **7b** (22.0 mg, 0.067 mmol, 1.0 eq) in CH_2Cl_2 (0.5 mL) at r.t.. Upon completion as monitored by TLC (20 % EtOAc in hexanes) after 1 h, sat. aq. NaCl (10 mL) and EtOAc (10 mL) were added. The layers were separated and the aqueous layer was extracted with EtOAc (3x 10 mL). The combined organic extracts were dried over Na_2SO_4, filtered and concentrated under reduced pressure. Purification by flash column chromatography (SiO_2, 20 % EtOAc in hexanes) afforded the title compound **8b** (14.2 mg, 0.065 mmol, 96 %) as an orange solid.
^{1}H NMR (400 MHz, $CDCl_3$) δ 7.18 (d, *J* = 8.0 Hz, 1H), 7.09 (d, *J* = 2.0 Hz, 1H), 7.06 (dd, *J* = 8.0, 2.0 Hz, 1H), 5.16 (s, 1H), 3.77 (t, *J* = 13.7 Hz, 2H), 2.33 (s, 3H), 2.24 (s, 3H).
^{19}F NMR (376 MHz, $CDCl_3$) δ -114.56.
^{13}C NMR (126 MHz, $CDCl_3$) δ 159.81 (t, *J* = 30.5 Hz), 134.95, 132.16, 126.30, 116.70 (t, *J* = 254.9 Hz), 51.51 (t, *J* = 25.2 Hz), 21.26, 17.98.
HRMS (ESI+): calculated for C11H13F2N2O $[M+H]^+$, 227.099, found 227.0988 Δ 1.0 ppm

2-(2,4-dimethylphenyl)-4,4-difluoro-1-(3-fluorobenzoyl)pyrazolidin-3-one (11b)

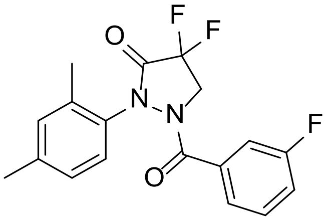

To a solution of **8b** (15.0 mg, 0.066 mmol, 1.0 eq) in CH_2Cl_2 (1.2 mL) was added TEA (27.7 µL, 0.199 mmol, 3.0 eq) and DMAP (0.4 mg, 0.002 mmol, 5 mol%). 3-methoxybenzoyl chloride (12.1 µL, 0.099 mmol, 1.5 eq) was added dropwise and the reaction mixture was stirred at r.t.. Upon completion as monitored by TLC (50 % EtOAc in hexanes) after 1 h, the reaction was quenched by addition of sat. aq. $NaHCO_3$ (5 mL). The layers were separated and the aqueous layer was extracted with CH_2Cl_2 (3x 10 mL). The combined organic extracts were dried over $MgSO_4$, filtered and concentrated under reduced pressure. Purification by flash column chromatography (SiO_2, 20 % EtOAc in hexanes) afforded the title compound **11b** (20.4 mg,0.059 mmol, 88 %) as a colorless solid.
^{1}H NMR (400 MHz, $CDCl_3$) δ 7.52 – 7.44 (m, 2H), 7.40 (ddd, *J* = 8.8, 2.8, 1.2 Hz, 1H), 7.33 (d, *J* = 8.1 Hz, 1H), 7.32 – 7.26 (m, 1H), 7.12 (d, *J* = 2.0 Hz, 1H), 7.06 (dd, *J* = 8.1, 2.0 Hz, 1H), 4.40 (t, *J* = 12.7 Hz, 2H), 2.42 (s, 3H), 2.32 (s, 3H).
^{19}F NMR (376 MHz, $CDCl_3$) δ -110.15, -110.16, -110.17, -110.18, -110.19, -110.21, -114.16.
^{13}C NMR (101 MHz, $CDCl_3$) δ 170.44, 164.05, 161.57, 139.51, 135.12, 133.65, 132.22, 131.98, 131.16, 131.08, 127.43, 125.49, 124.55, 120.55, 120.34, 116.12, 115.89, 114.08, 56.92, 56.64, 56.36, 21.25, 18.34.
HRMS (ESI +): calculated for $C_{18}H_{15}F_3N_2NaO_2$ $[M+Na]^+$, 371.0978, found 371.0975 Δ 0.6 ppm
LC-MS (ESI+) m/z found 349.0 $[M+H]^+$, calculated for $C_{18}H_{16}F_3N_2O_2$; **Purity by LC-MS** (254 nm): >99%

tert-butyl 2-(2,4-dimethylphenyl)hydrazine-1-carboxylate

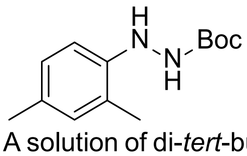

A solution of di-*tert*-butyl decarbonate (6.320 g, 2.90 mmol, 1.0 eq) in THF (20 mL) was added dropwise to a suspension of 2,4-dimethylpheyl hydrazine hydrochloride (5.00 g, 2.90 mmol, 1.0 eq) in THF (200 mL) at 0 °C. The reaction mixture was allowed to reach r.t. until completion as monitored by TLC

(20 % EtOAc in hexane) after 1.5 h. The reaction mixture was concentrated under reduced pressure. EtOAc (100 mL) and sat. aq. $NaHCO_3$ (100 mL) were added, the layers were separated and the aqueous layer was extracted with EtOAc (3x 50 mL). the combined organic extracts were dried over Na_2SO_4, filtered and concentrated under reduced pressure. The crude product was used without any further purification. The characterization data of the compound is in accordance with reported spectra[4].
^{1}H NMR (400 MHz, $CDCl_3$) δ 6.94 (dd, *J* = 8.2, 2.0 Hz, 1H), 6.88 (d, *J* = 2.0 Hz, 1H), 6.78 (d, *J* = 8.1 Hz, 1H), 6.31 (s, 1H), 5.56 (s, 1H), 2.24 (s, 3H), 2.19 (s, 3H), 1.52 – 1.36 (m, 9H).

4-fluoro-N'-phenylbenzohydrazide

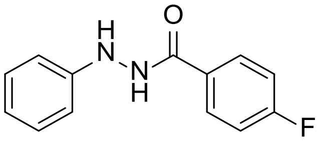

4-fluorobenzoylchloride (0.75 mL, 6.37 mmol, 1.1 eq) was added dropwise to a solution of phenylhydrazine (1 mL, 5.79 mmol, 1.0 eq.) in 2,6-lutidine (20 mL) at -18 °C. The reaction mixture was stirred at -18 °C until completion as monitored by TLC (20 % EtOAc in hexane) after 2 h. 1 M HCl (250 mL) and CH_2Cl_2 (100 mL) were added, the layers were separated and the aqueous layer was extracted with CH_2Cl_2 (3x 100 mL). The combined organic extracts were dried over Na_2SO_4, filtered and concentrated under reduced pressure. Purification by flash column chromatography (SiO_2, 15 % EtOAc in hexane) afforded the title compound as a colorless crystalline solid.
^{1}H NMR (500 MHz, $CDCl_3$) δ 7.92 (s, 1H), 7.88 – 7.82 (m, 2H), 7.27-7.24 (m, 2H), 7.15 (t, *J* = 8.5 Hz, 2H), 6.94 (dd, *J* = 5.5, 4.0 Hz, 3H), 5.88 (s, 1H).
^{19}F NMR (471 MHz, $CDCl_3$) δ -106.93.

methyl 3-(chlorocarbonyl)bicyclo[1.1.1]pentane-1-carboxylate

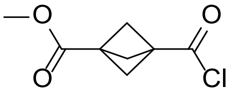

To a solution of 3-(methoxycarbonyl)bicyclo[1.1.1]pentane-1-carboxylic acid (470.0 mg, 2.76 mmol, 1.0 eq) in Et_2O (10.6 mL) was added oxalylchloride (481.9 µL, 5.52 mmol, 2.0 eq) and then DMF (21.4 µ, 0.28 mmol, 10 mol%). The reaction mixture was stirred at r.t. for 30 min. The volatiles were removed under reduced pressure to afford methyl 3-(chlorocarbonyl)bicyclo[1.1.1]pentane-1-carboxylate as an off-white solid, which was immediately used for the next step without any further purification.

3-fluorobicyclo[1.1.1]pentane-1-carboxylic acid

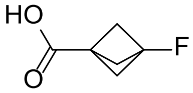

Bicyclo[1.1.1]pentane-1,3-dicarboxylic acid (250 mg, 1.60 mmol, 1.0 eq), SelectFluor (1.418 g, 4.00 mmol, 2.5 eq) and $AgNO_3$ (20.4 mg, 0.12 mmol, 7.5 mol%) were flushed with Argon. Degassed H_2O (8.0 mL) was added and the reaction mixture was stirred under reflux at 65 °C. After 16 h, the reaction mixture was allowed to reach r.t.. Et_2O (20 mL) was added, the layers were separated and the aqueous layer was extracted with Et_2O (3x 15 mL). The combined organic extracts were dried over Na_2SO_4, filtered and concentrated under reduced pressure. The residue was suspended in pentane (5.0 mL), sonicated and filtered. The filtrated was concentrated under reduced pressure to afford the title compound as a colorless crystalline solid (147.1 mg, 1.13 mmol, 71 %). The characterization data of the compound is in accordance with reported spectra.[5]
^{1}H NMR (400 MHz, $CDCl_3$) δ 2.40 (d, *J* = 2.4 Hz, 1H).

3-((tert-butyldimethylsilyl)oxy)benzoic acid

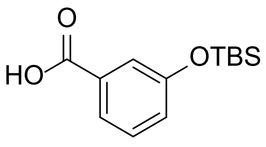

This procedure was adapted from Strong *et* al.[6].To a solution of 3-hydroxybenzoic acid (100.0 mg, 0.724 mmol, 1.0 eq) in DMF (3.6 mL) at r.t. was added 1*H*-imidazole (295.7 mg, 4.344 mmol, 6.0 eq) and *tert*-butyldimethylsilylchloride (436.4 mg, 2.896 mmol, 4.0 eq) in portions. The reaction mixture was stirred at r.t. until completion as monitored by TLC (10 % EtOAc in hexanes) after 15 h. Water (10 mL) and hexane (10 mL) were added, the layers were separated and the aqueous layer was extracted with hexane (3x 10 mL). The combined organic extracts were dried over $MgSO_4$, filtered and concentrated under reduced pressure. THF (0.39 mL), acetic acid (1.2 mL) and water (3.9 mL) were added to the residue in this order. The mixture was stirred at r.t. until completion as monitored by TLC (10 % EtOAc in hexanes) after 3 h. The reaction mixture was concentrated under reduced pressure. Purification by flash column chromatography (SiO_2, 10% EtOAc in hexanes) afforded the title compound (156.6 mg, 0.620 mmol, 86 %) as a colorless solid. The characterization data of the compound is in accordance with reported spectra.[6]
^{1}H NMR (400 MHz, $CDCl_3$) δ 7.71 (dt, *J* = 7.7, 1.3 Hz, 1H), 7.56 (dd, *J* = 2.6, 1.6 Hz, 1H), 7.33 (t, *J* = 7.9 Hz, 1H), 7.09 (ddd, *J* = 8.1, 2.6, 1.1 Hz, 1H), 1.00 (s, 9H), 0.23 (s, 6H).

Spectra of compounds

Characterization of (6a)

1H-NMR

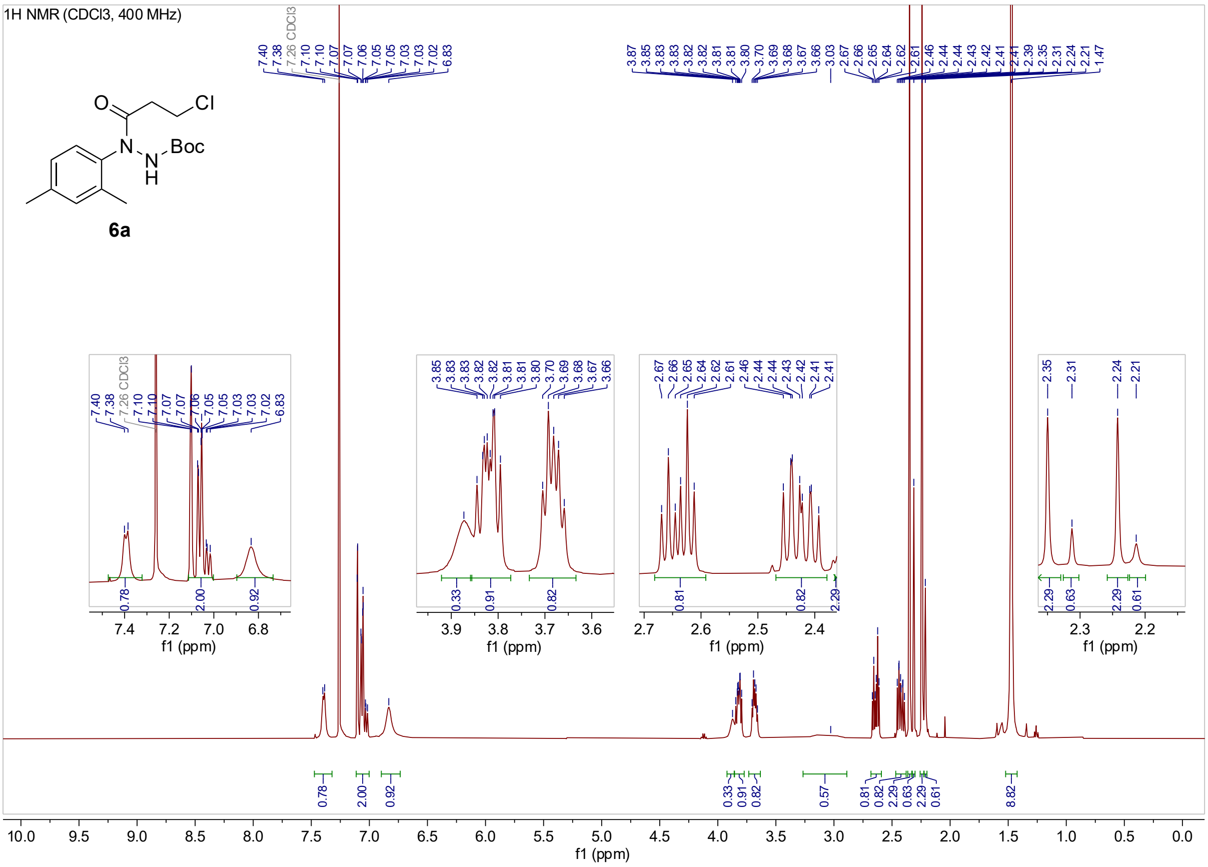

^{1}H NMR spectrum of (**6a**)

13C-NMR

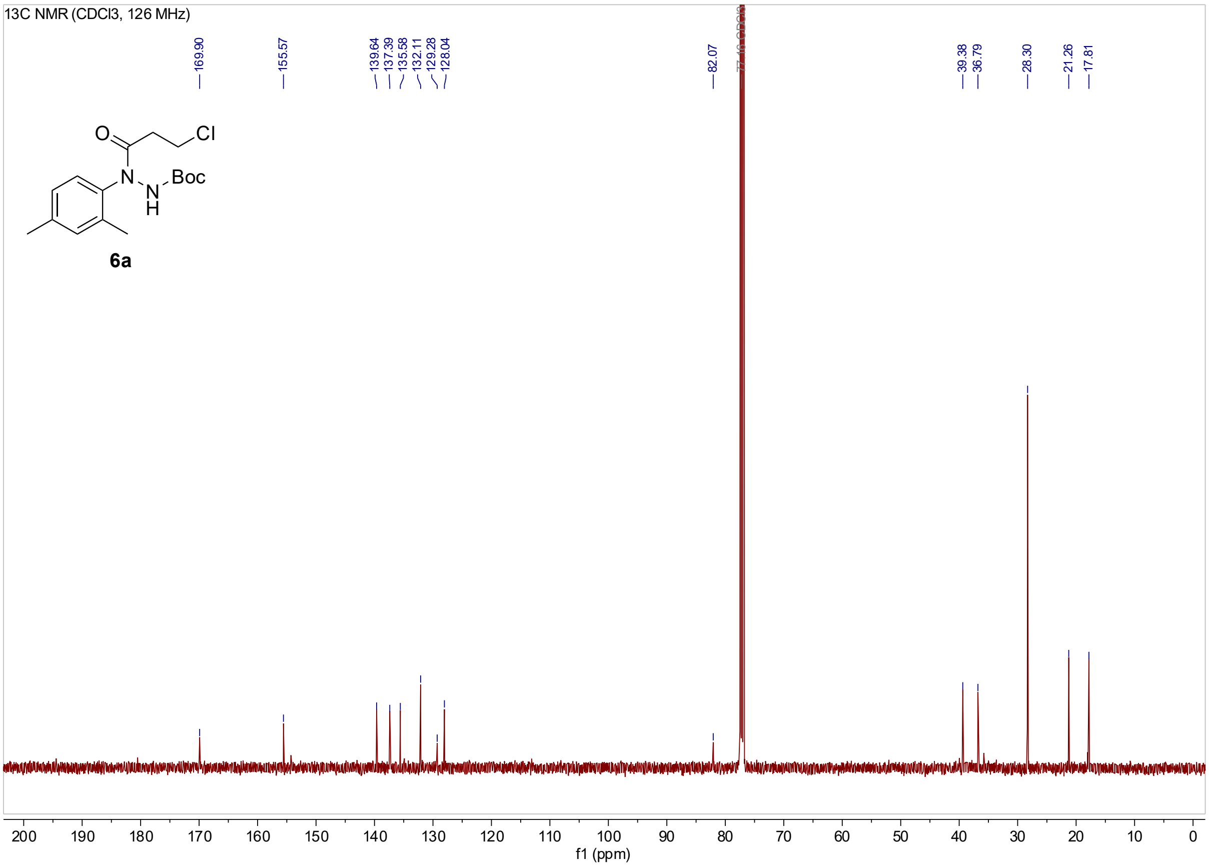

^{13}C NMR spectrum of (**6a**)

HRMS

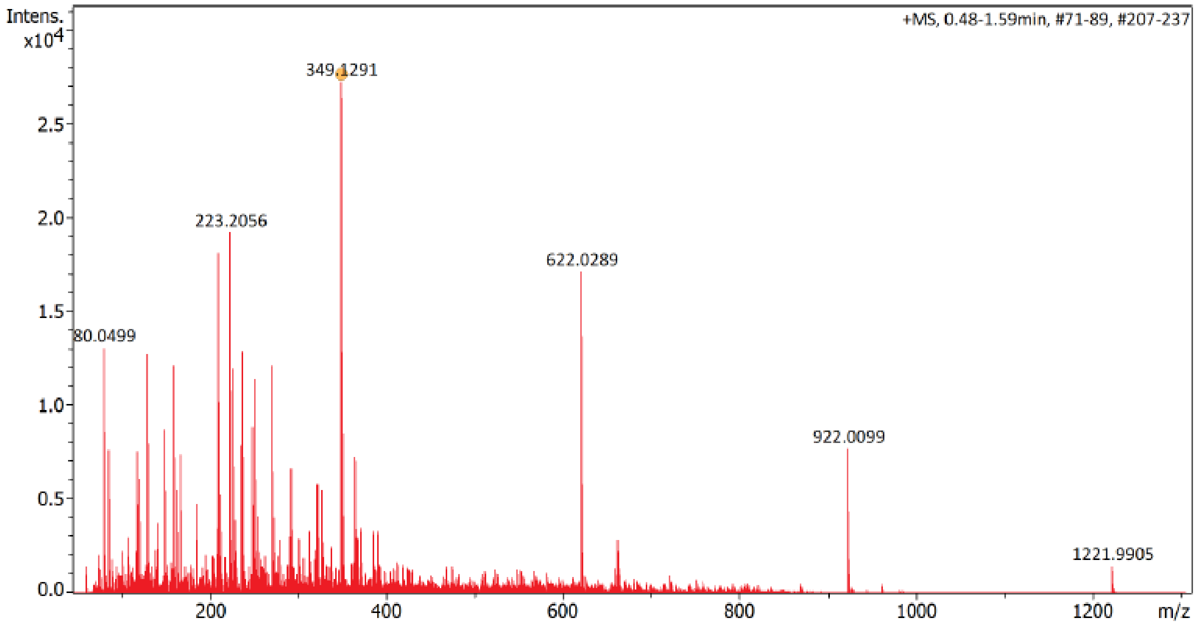

HRMS(ESI+) spectrum of (**6a**)

Characterization of (7a)

1H-NMR

1H NMR (CDCl3, 500 MHz)

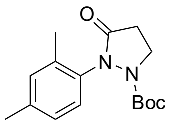

7a

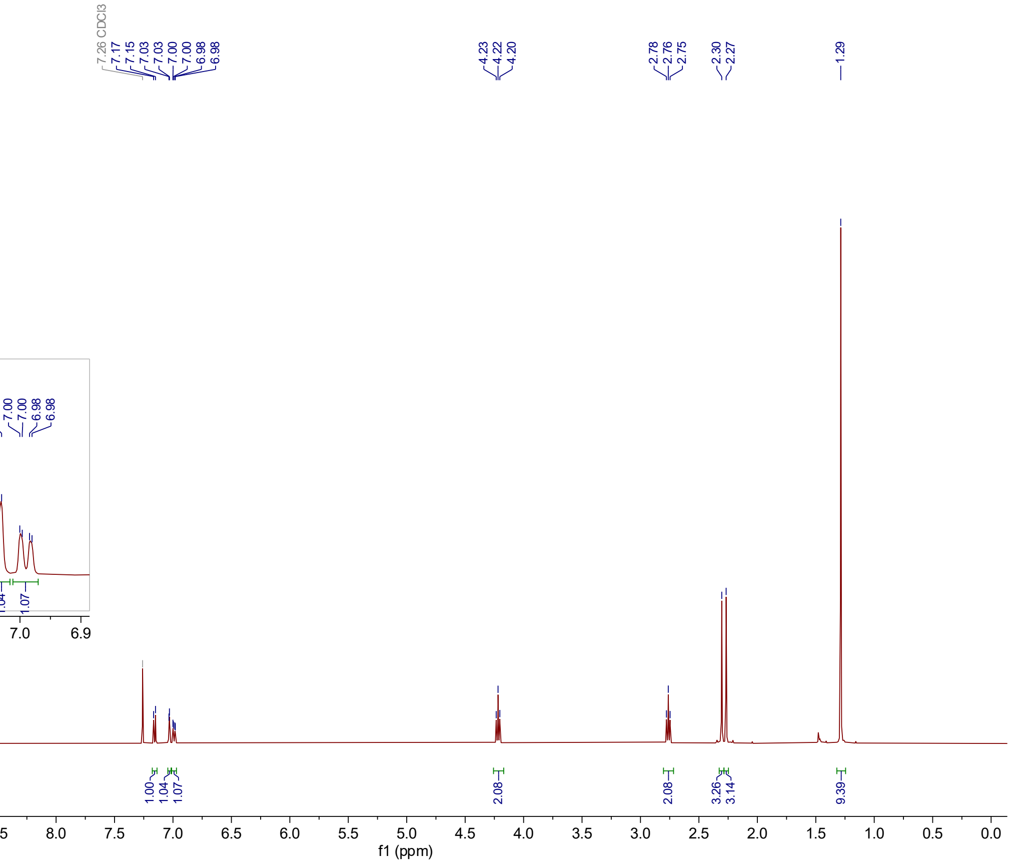

^{1}H NMR spectrum of (**7a**)

13C-NMR

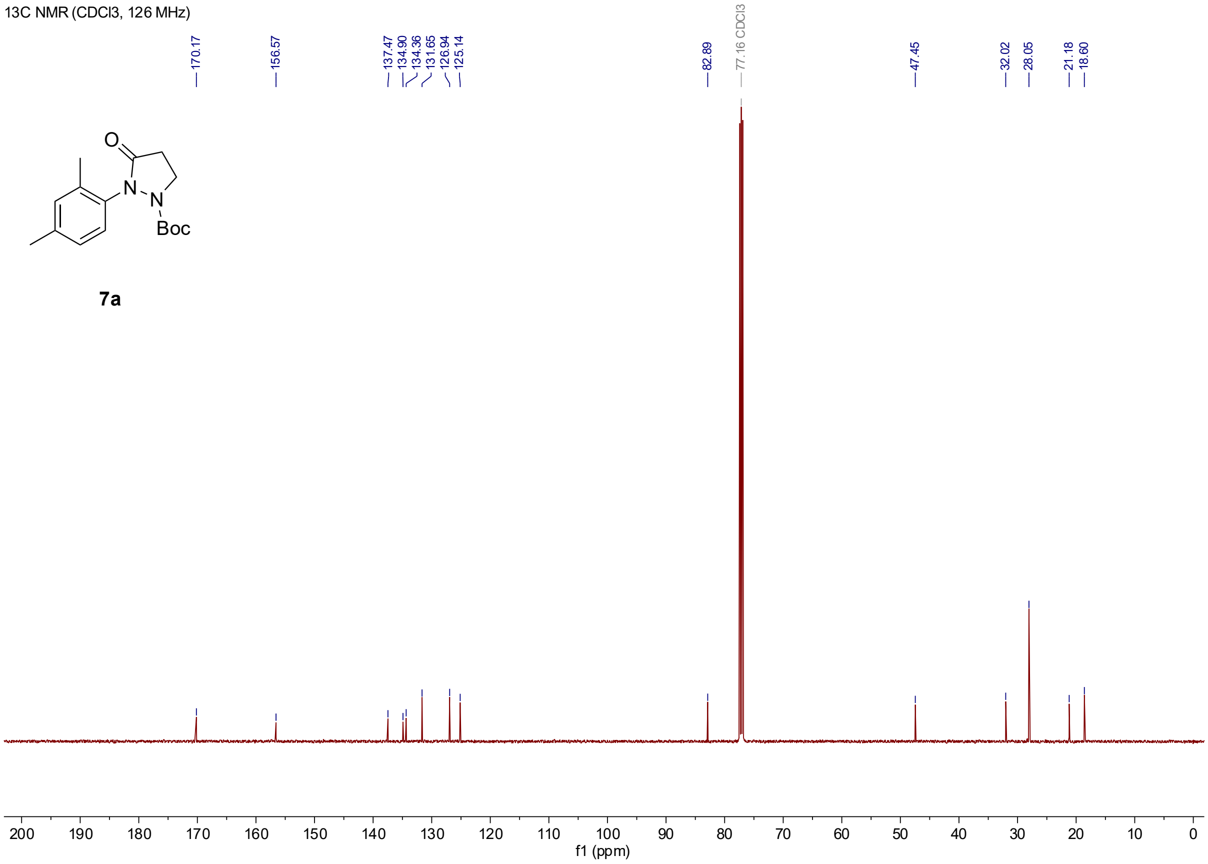

^{13}C NMR spectrum of (**7a**)

HRMS

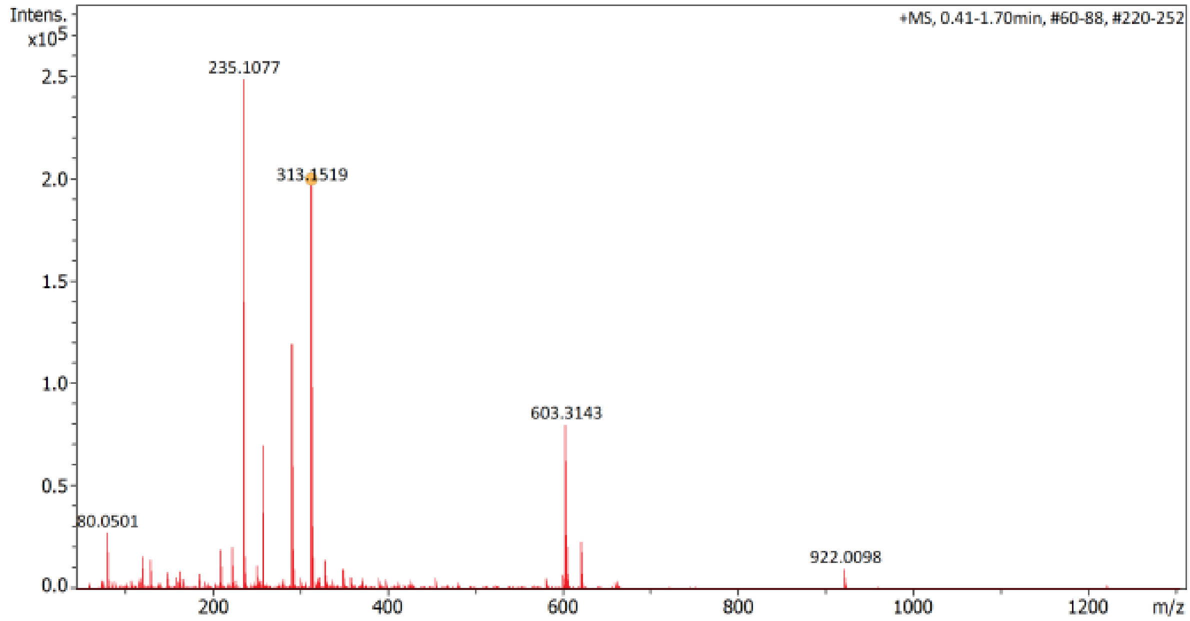

HRMS(ESI+) spectrum of (**7a**)

Characterization of (8a)

1H-NMR

1H NMR (CDCl3, 400MHz)

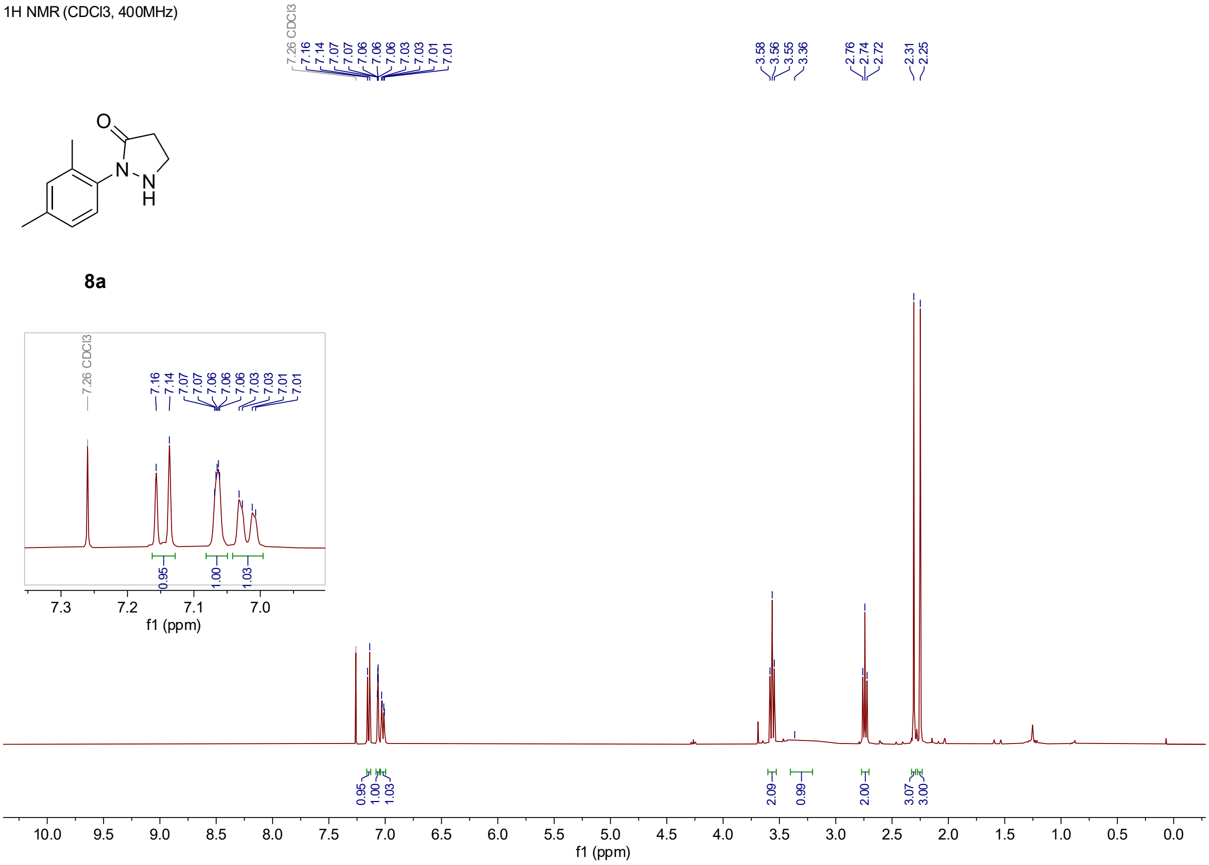

^{1}H NMR spectrum of (**8a**)

13C-NMR

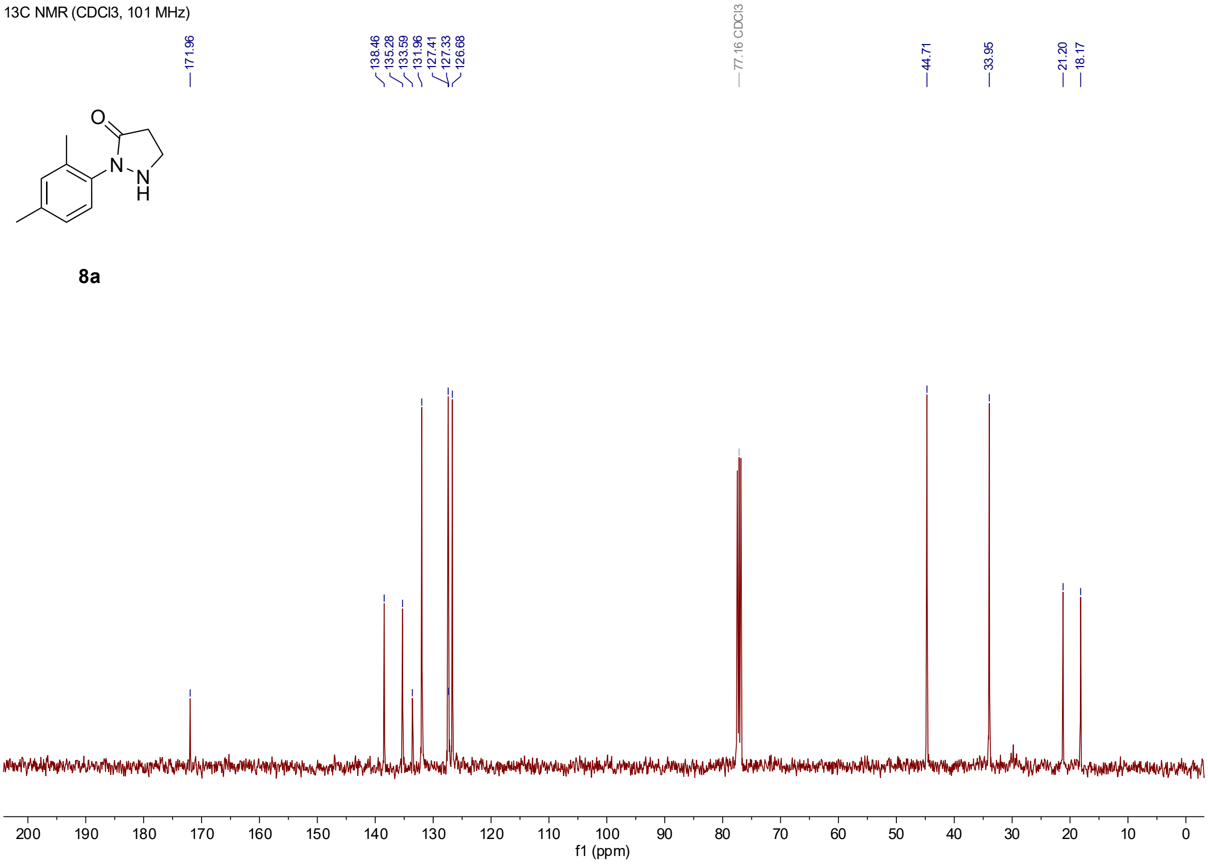

8a

^{13}C NMR spectrum of (**8a**)

HRMS

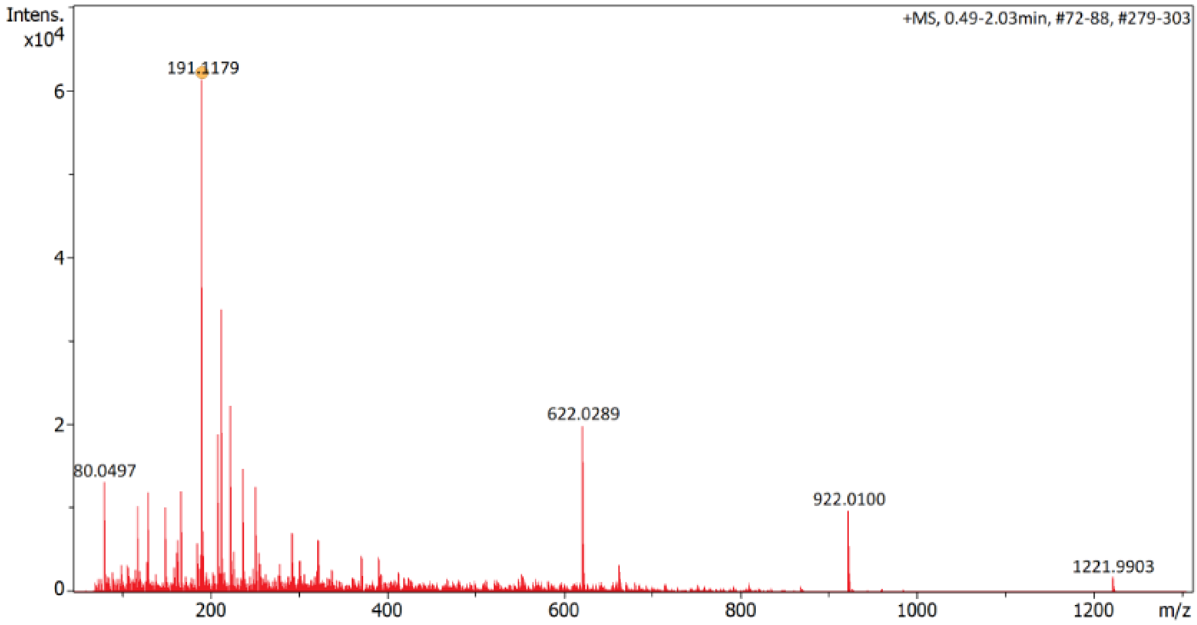

HRMS(ESI+) spectrum of (**8a**)

Characterization of (9a)

^{1}H NMR spectrum of (9a)

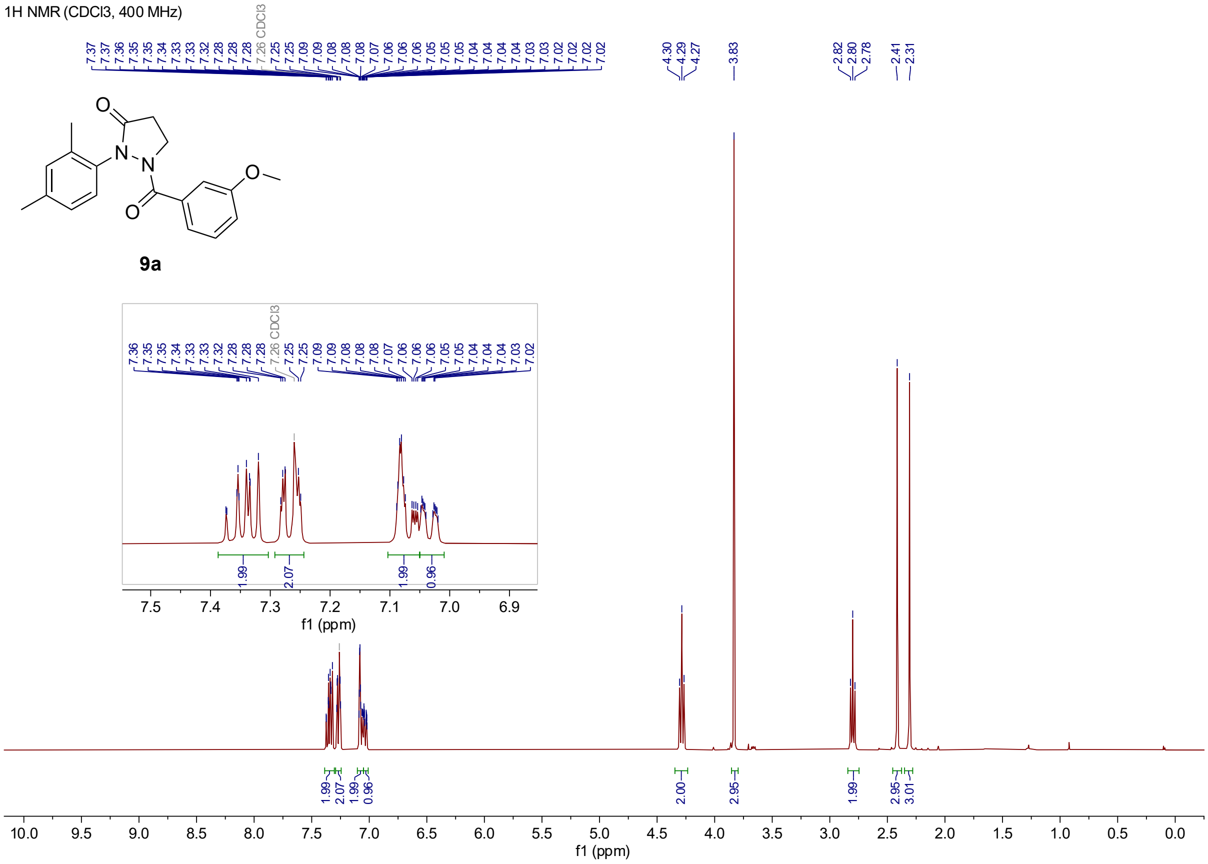

^{1}H NMR spectrum of (**9a**)

^{13}C NMR spectrum of (9a)

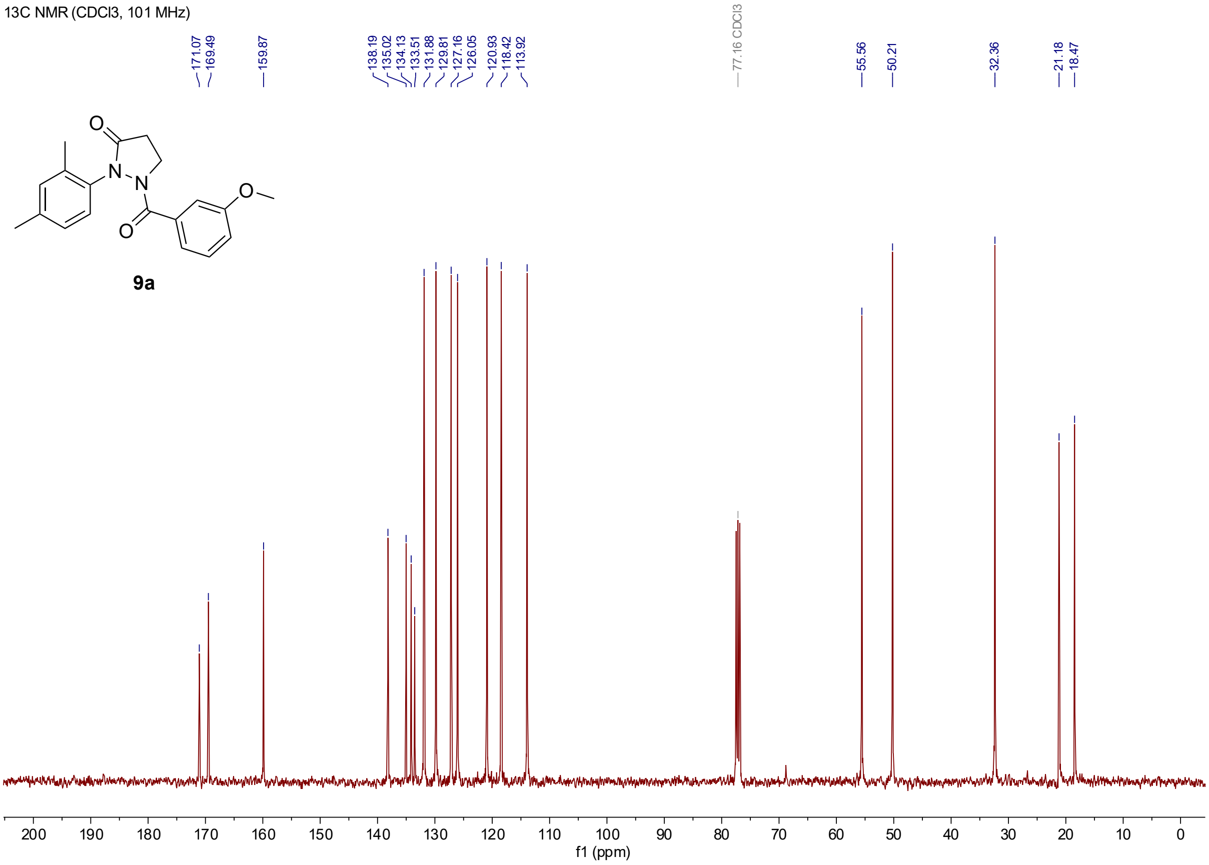

^{13}C NMR spectrum of (**9a**)

HRMS(ESI+) spectrum of (9a)

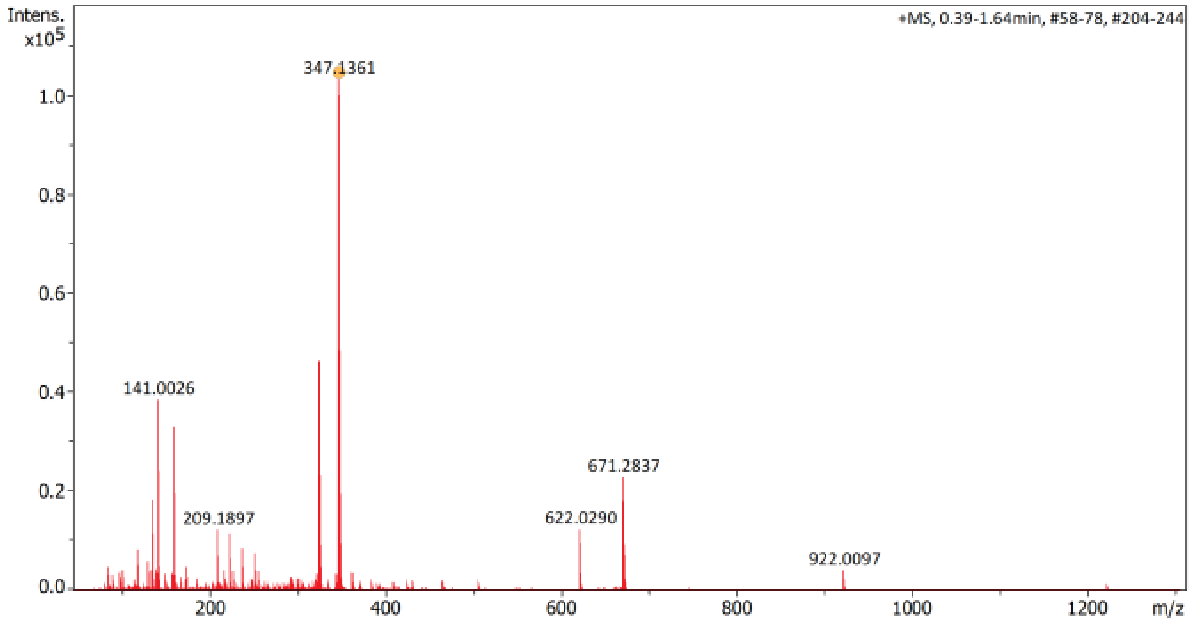

HRMS(ESI+) spectrum of (**9a**)

LC-MS HPLC chromatogram at 254.4 nm of (9a)

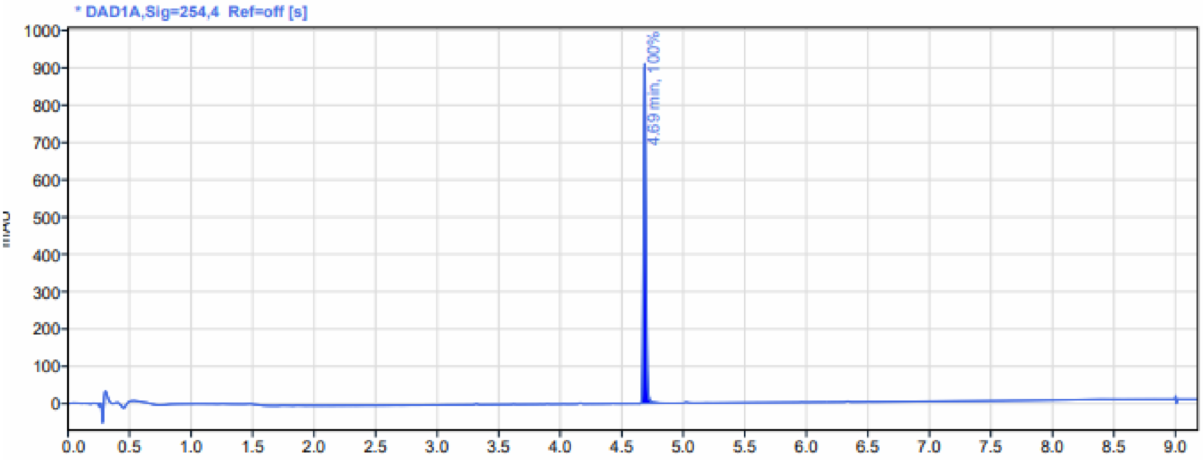

LC-MS HPLC chromatogram at 254.4 nm of (**9a**)

Characterization of (10a)

^{1}H NMR spectrum of (10a)

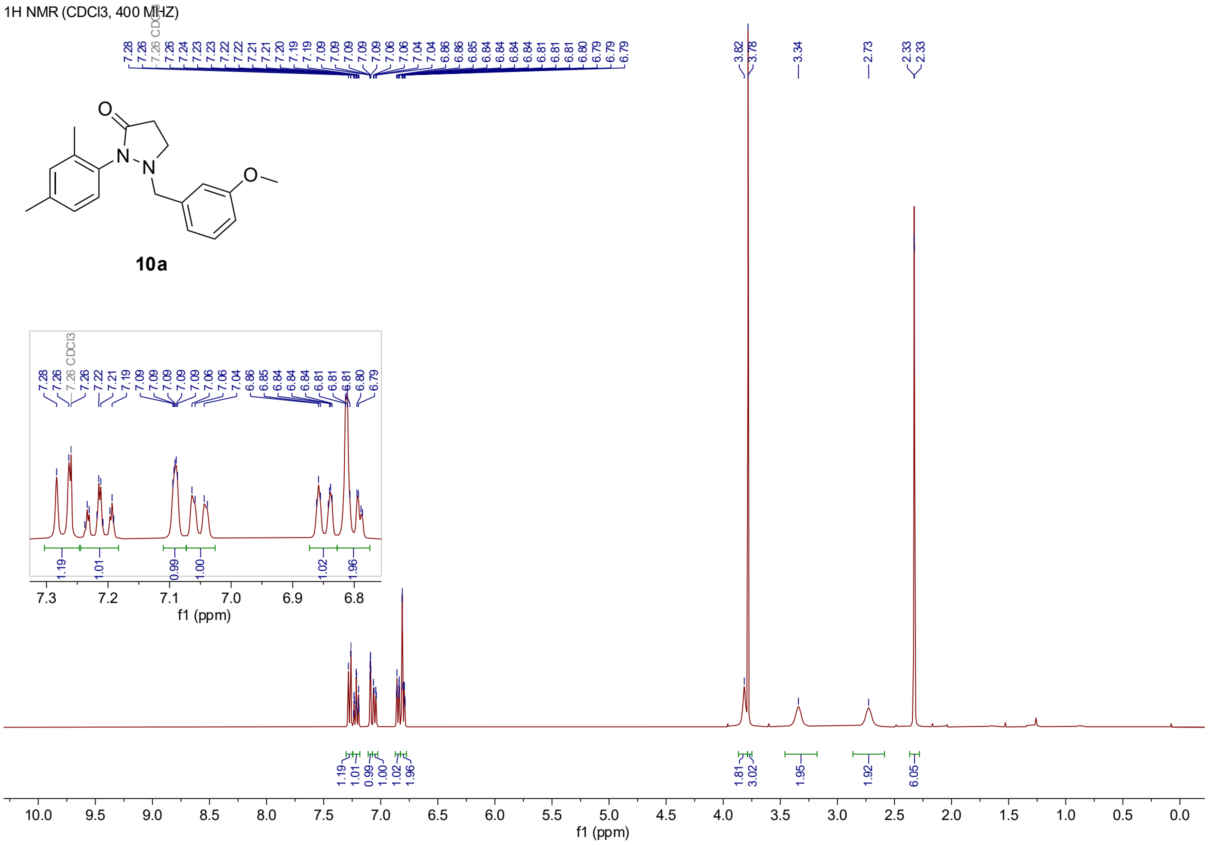

^{1}H NMR spectrum of (**10a**)

13C-NMR

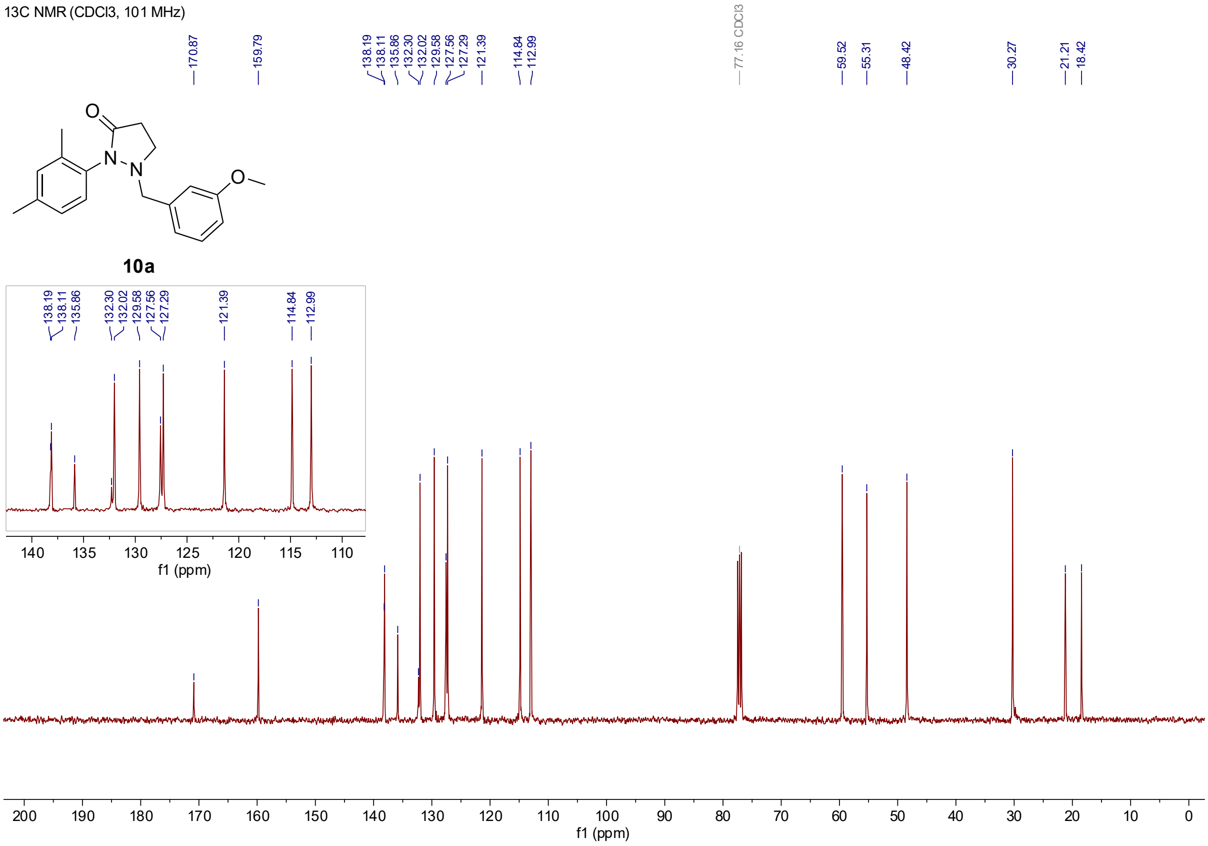

^{13}C NMR spectrum of (**10a**)

HRMS

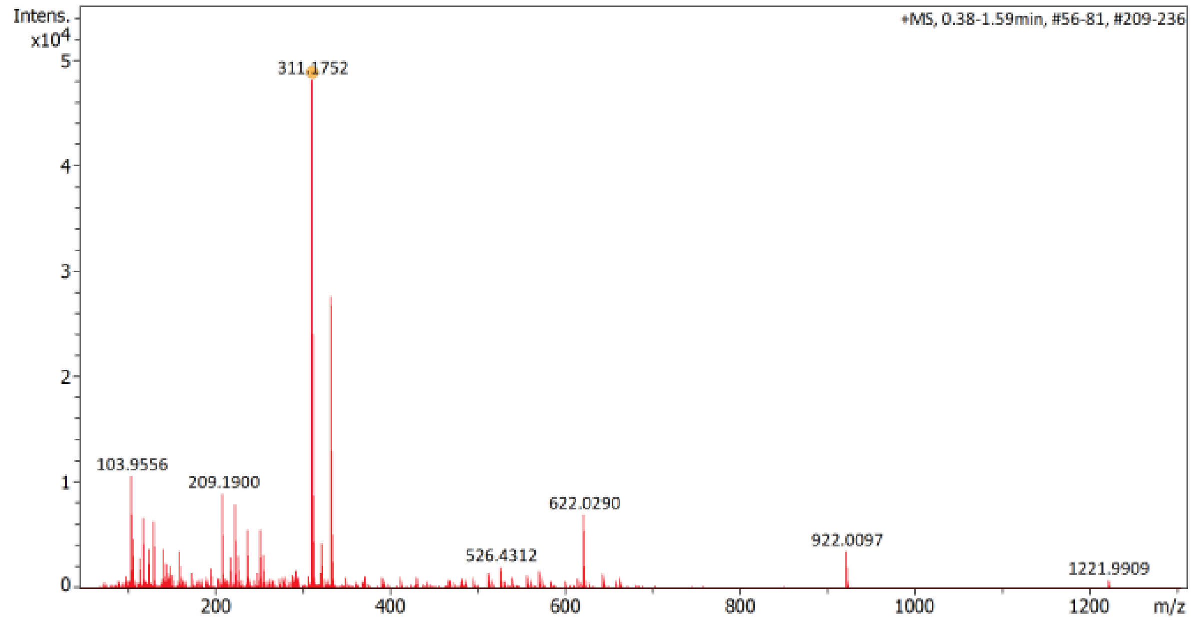

HRMS(ESI+) spectrum of (**10a**)

LC-MS

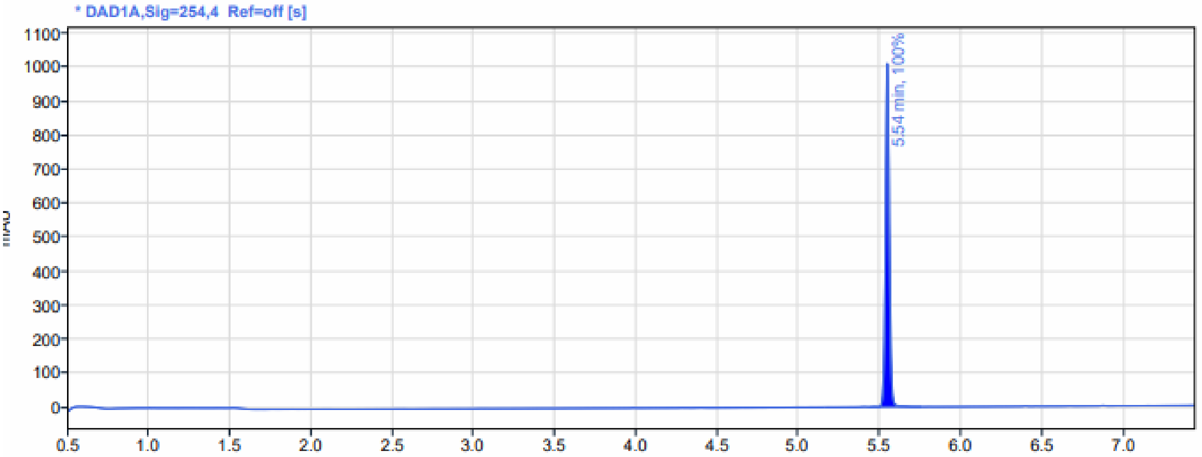

LC-MS HPLC chromatogram at 254.4 nm of (**10a**)

Characterization of (11a)

1H-NMR

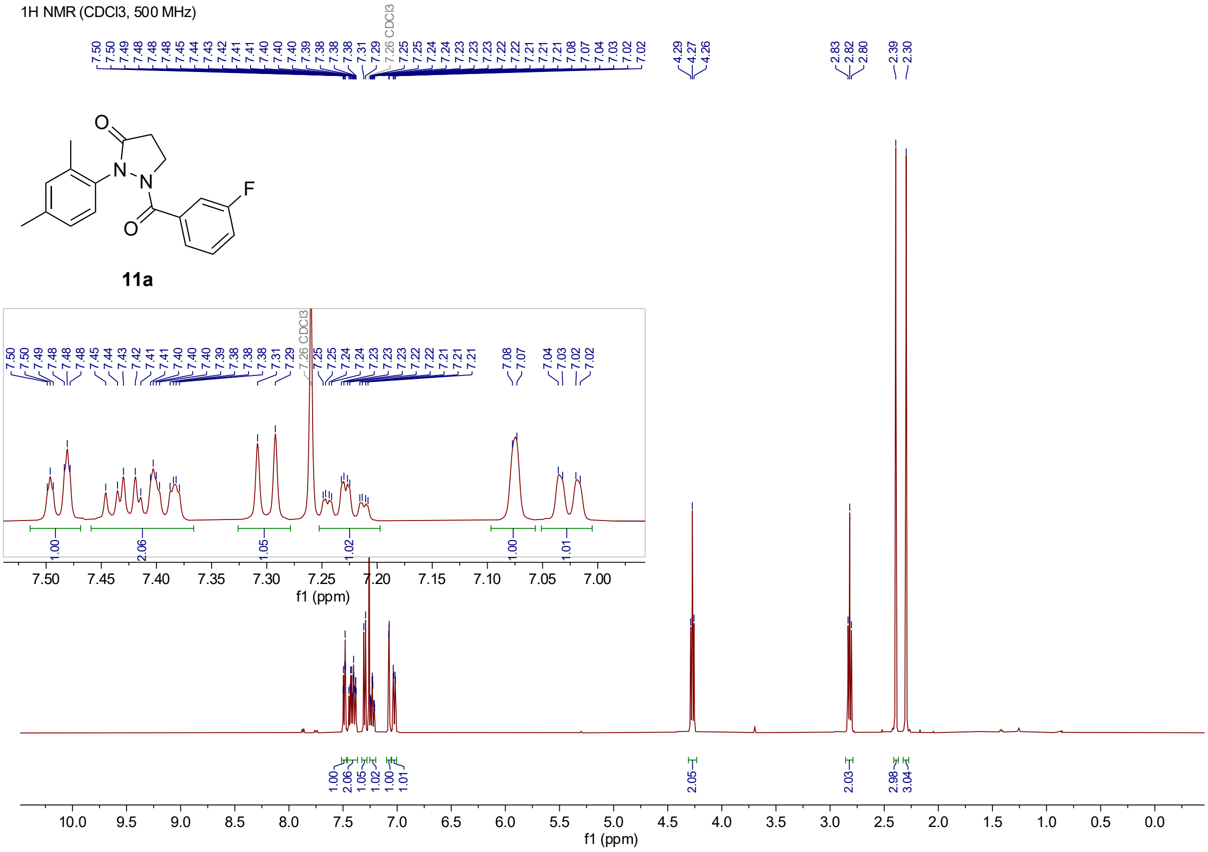

^{1}H NMR spectrum of (**11a**)

19F-NMR

19F NMR (CDCl3, 471 MHz)

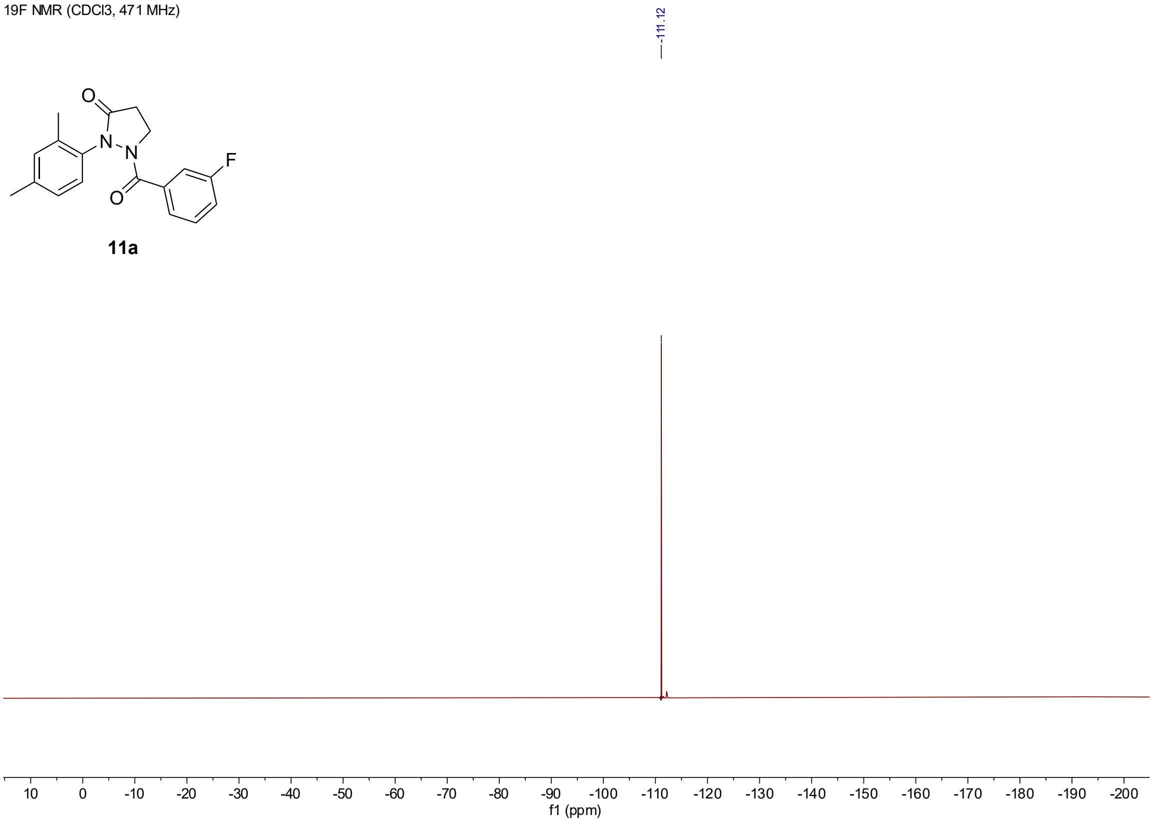

^{19}F NMR spectrum of (**11a**)

13C-NMR

13C NMR (CDCl3, 126 MHz)

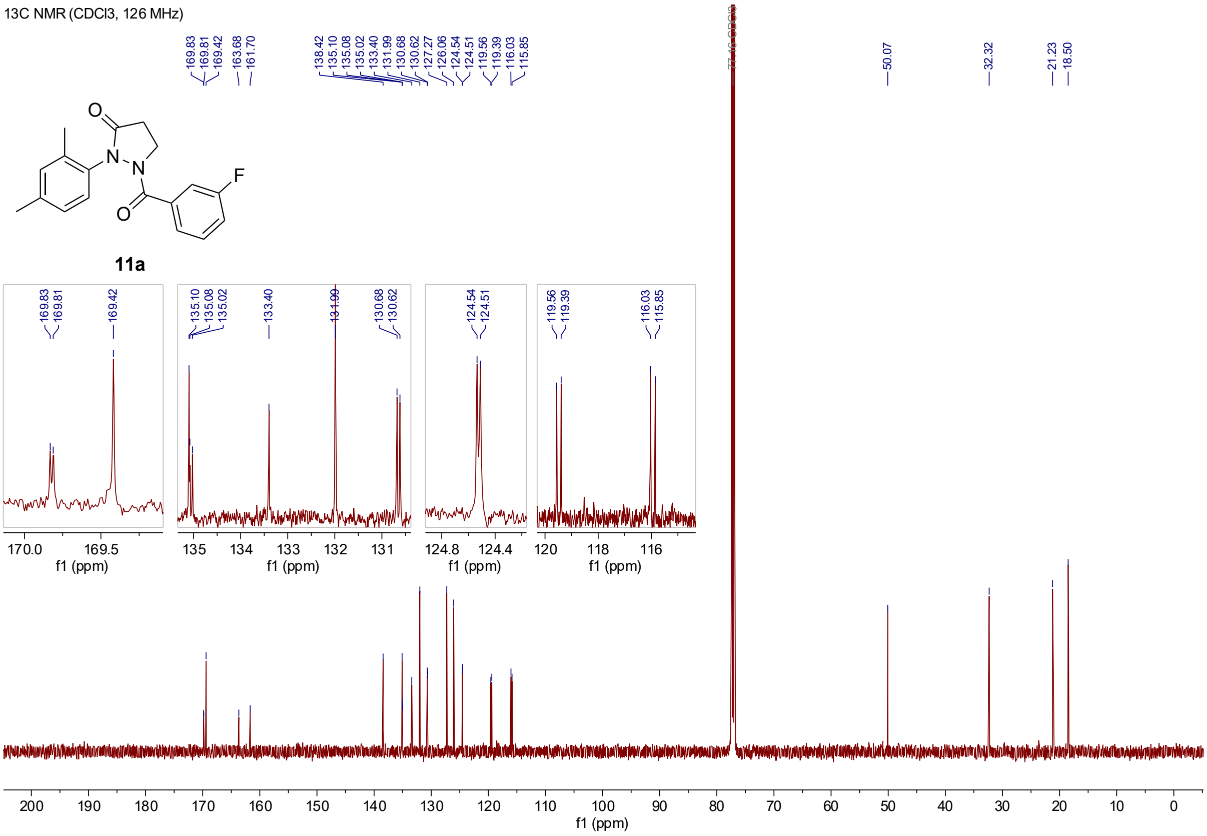

^{13}C NMR spectrum of (**11a**)

HRMS

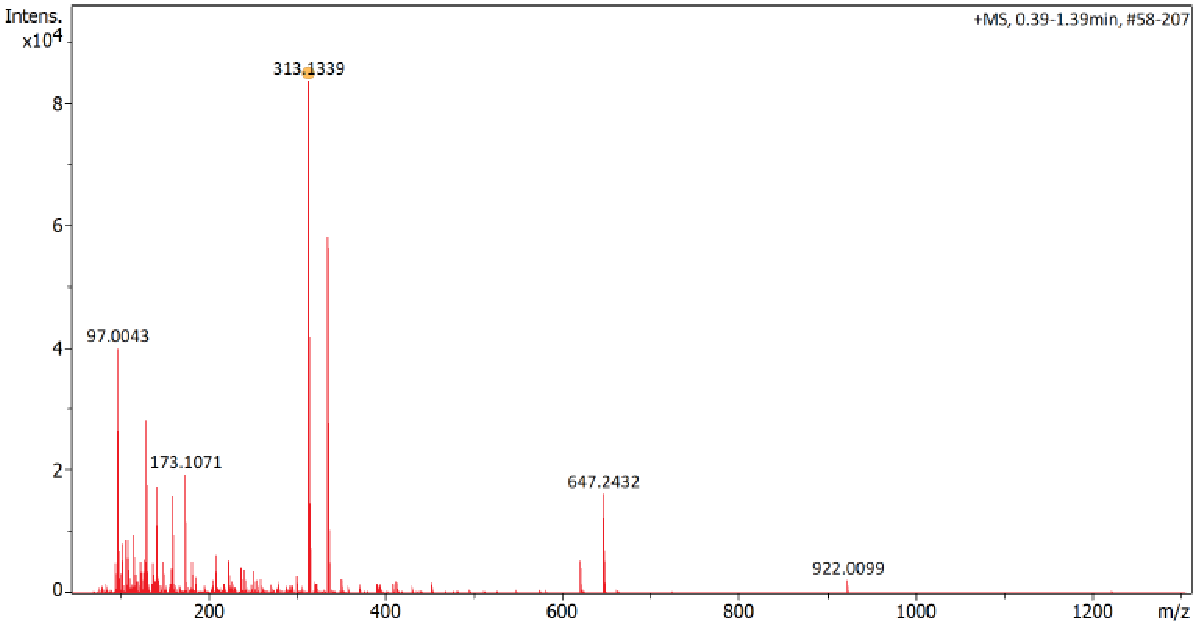

HRMS(ESI+) spectrum of (**11a**)

LC-MS

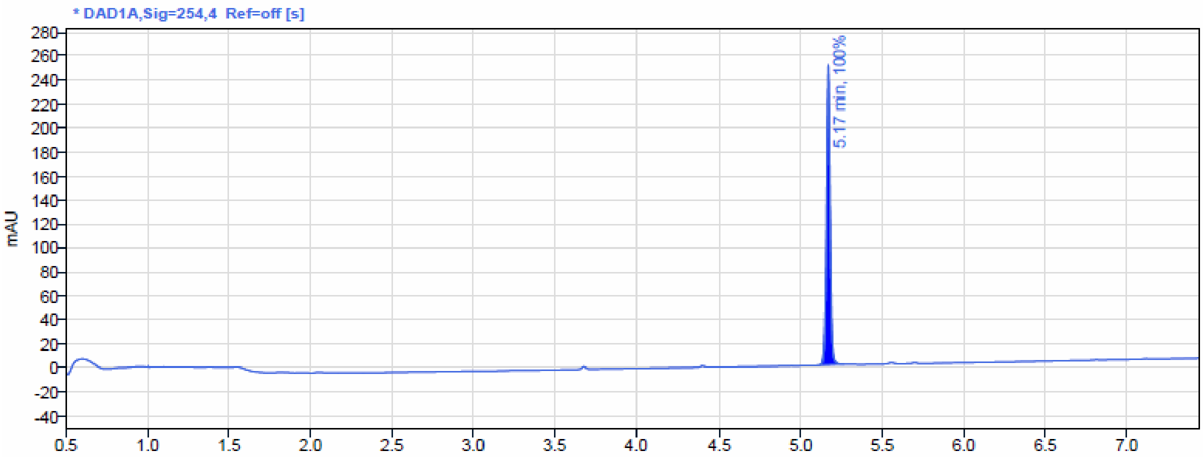

LC-MS HPLC chromatogram at 254.4 nm of (**11a**)

Characterization of (12a)

1H-NMR

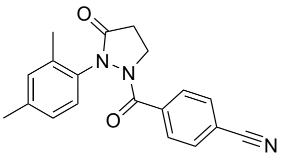

12a

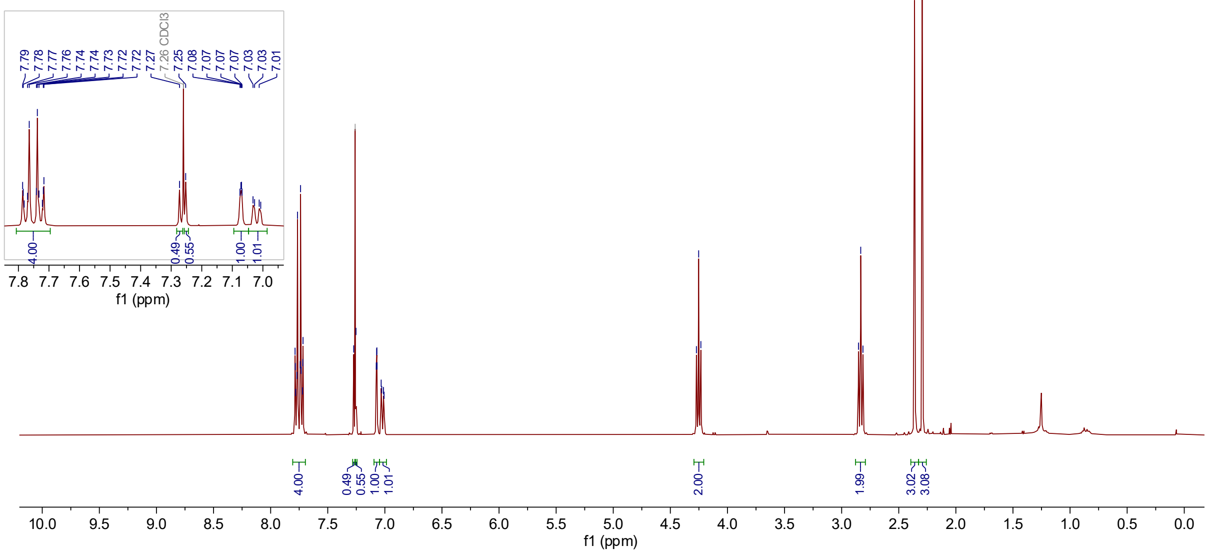

^{1}H NMR spectrum of (**12a**)

13C-NMR

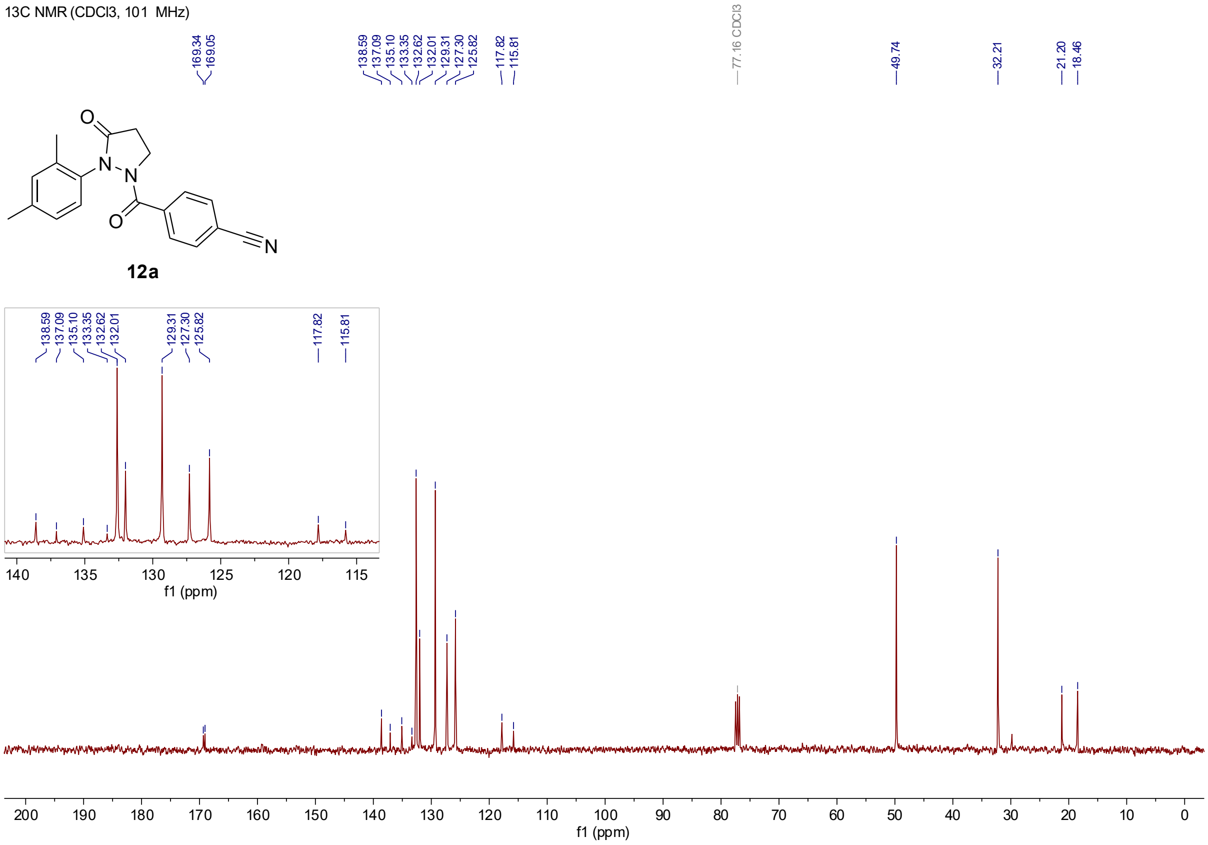

^{13}C NMR spectrum of (**12a**)

HRMS

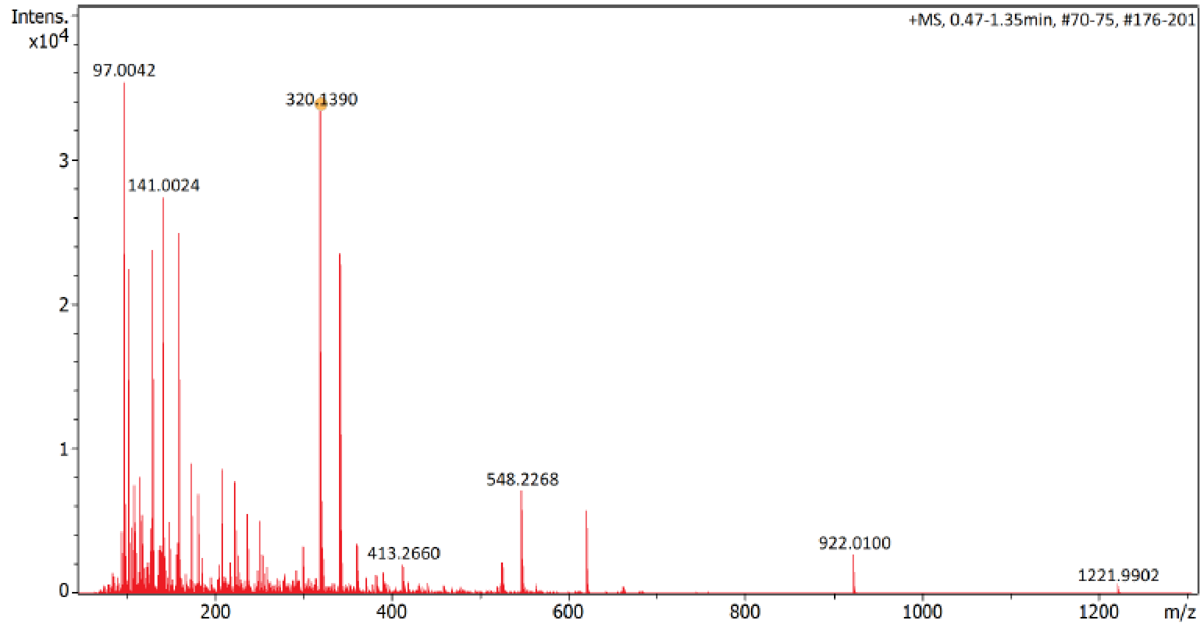

HRMS(ESI+) spectrum of (**12a**)

LC-MS

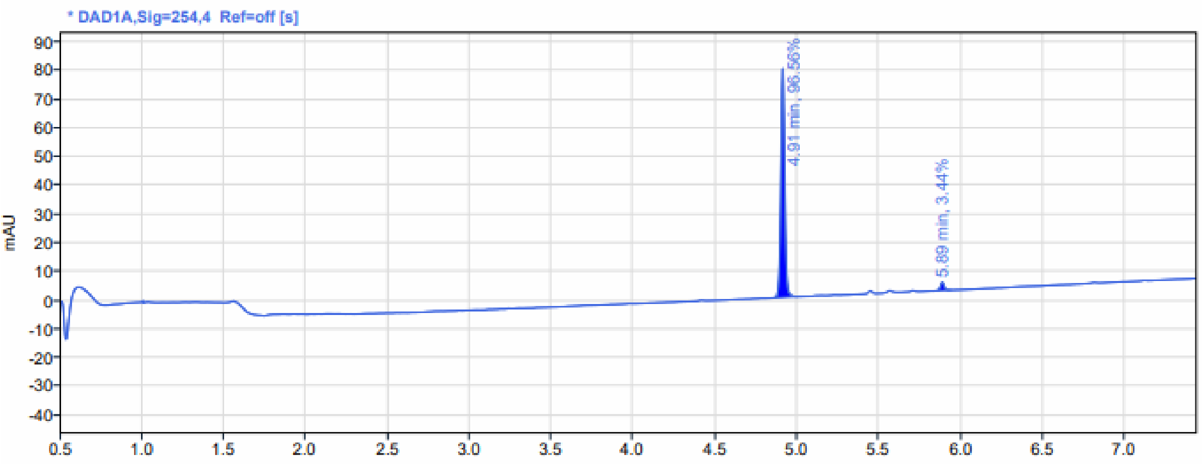

LC-MS HPLC chromatogram at 254.4 nm of (**12a**)

Characterization of (13a)

1H-NMR

1H NMR (CDCl3, 400 MHz)

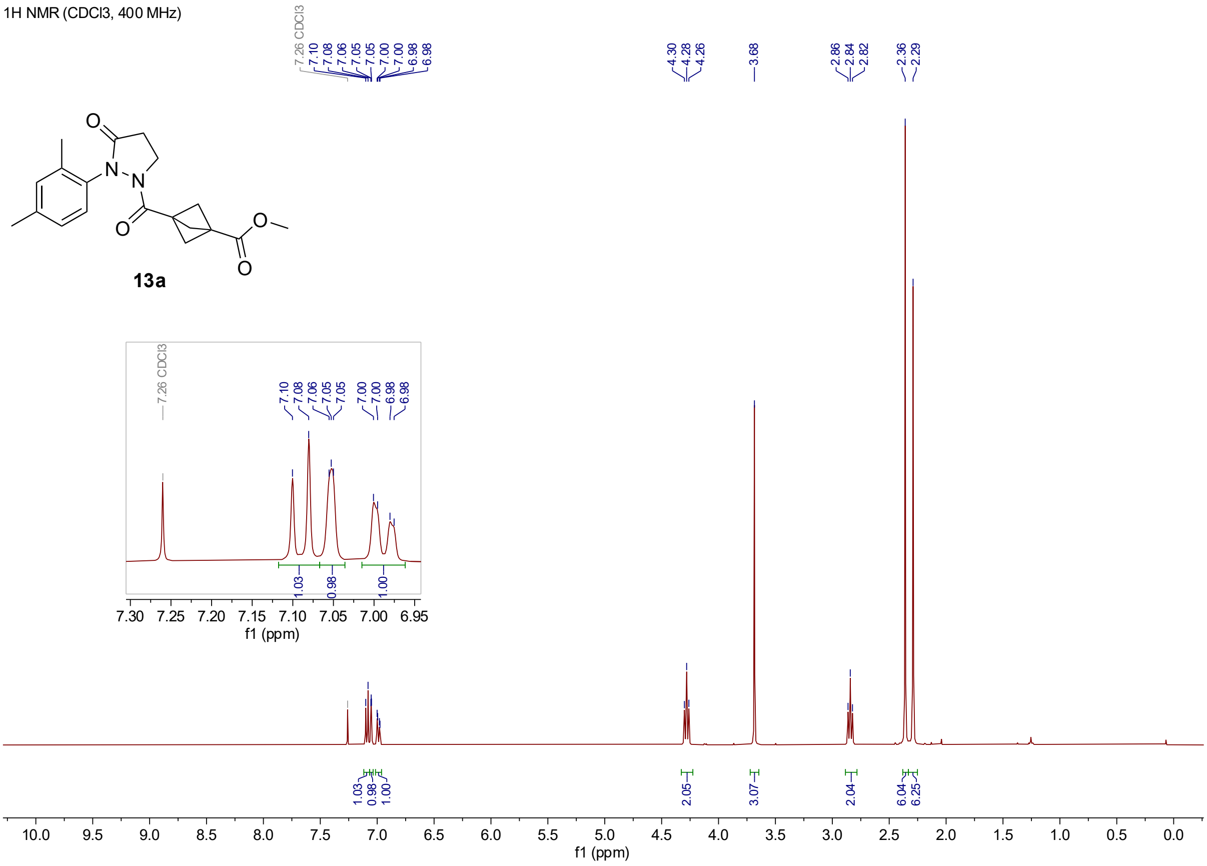

^{1}H NMR spectrum of (**13a**)

13C-NMR

13C (CDCl3, 101 MHz)

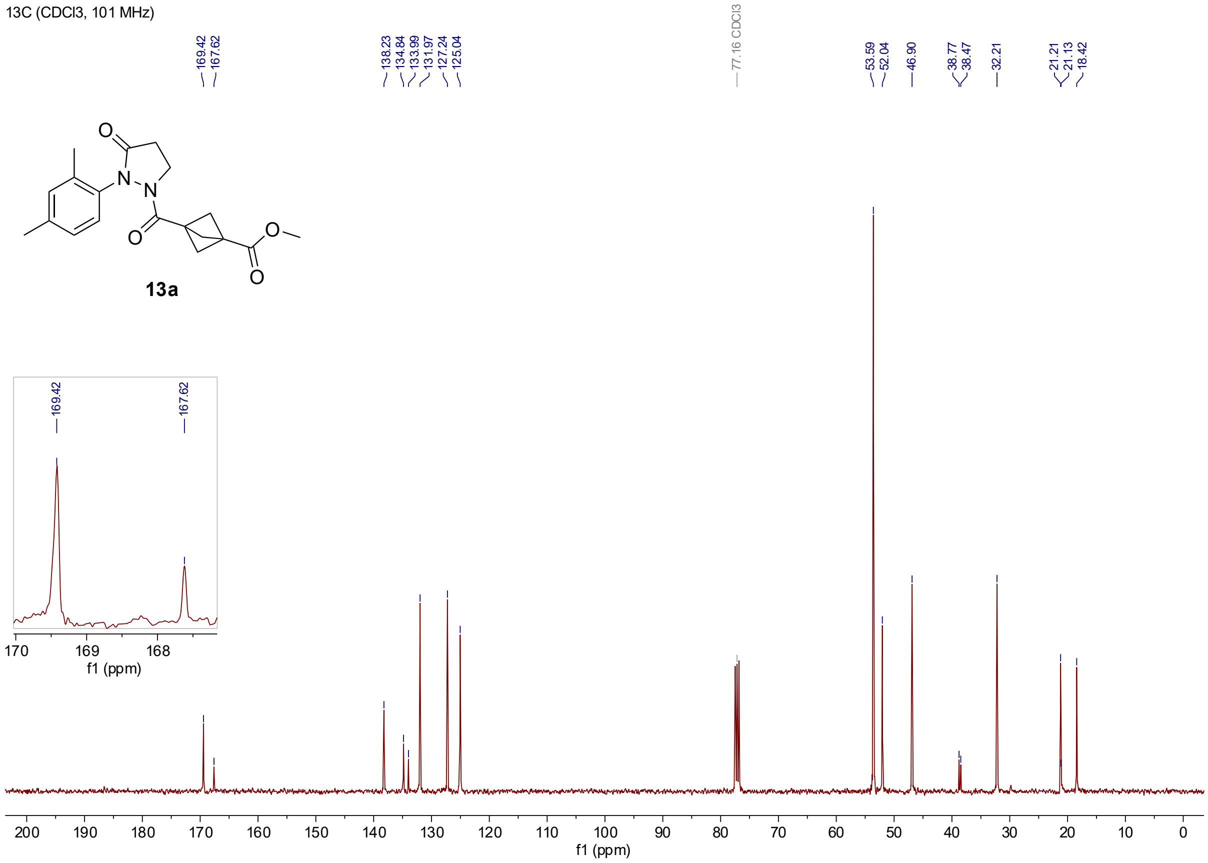

^{13}C NMR spectrum of (**13a**)

HRMS

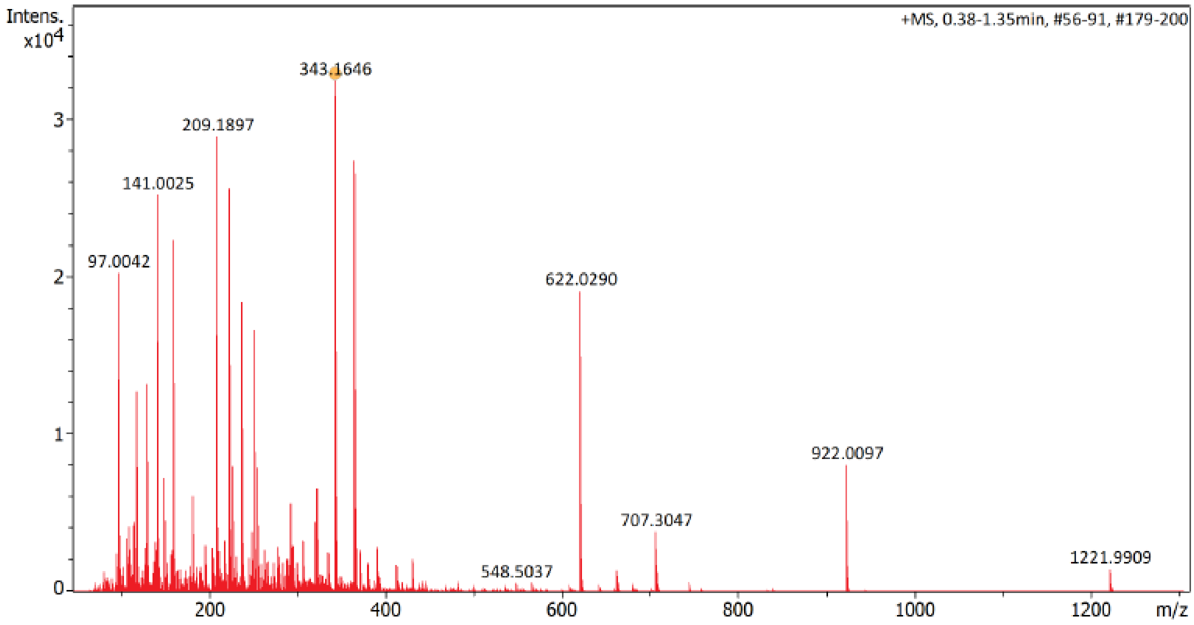

HRMS(ESI+) spectrum of (**13a**)

LC-MS

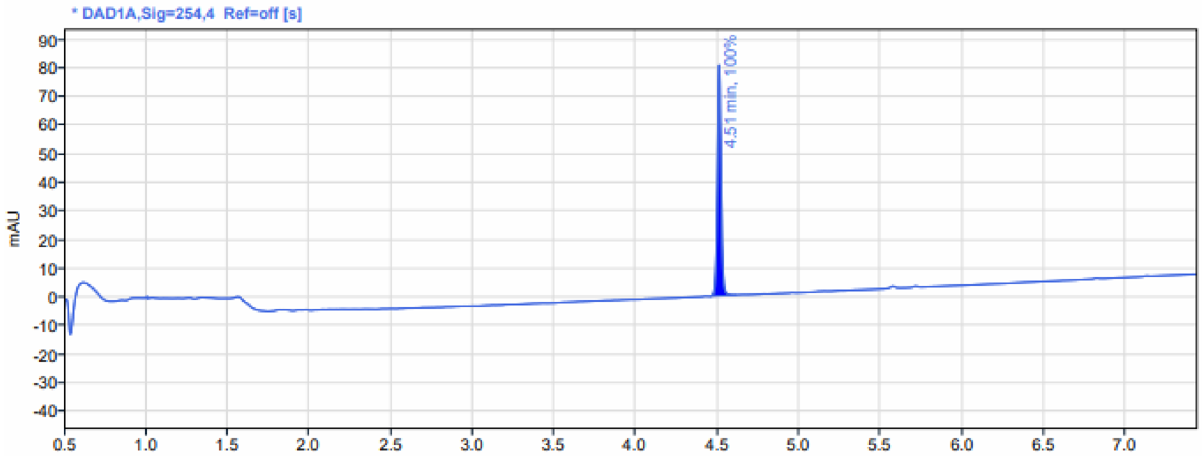

LC-MS HPLC chromatogram at 254.4 nm of (**13a**)

Characterization of (14a)

1H-NMR

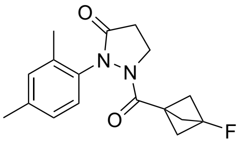

14a

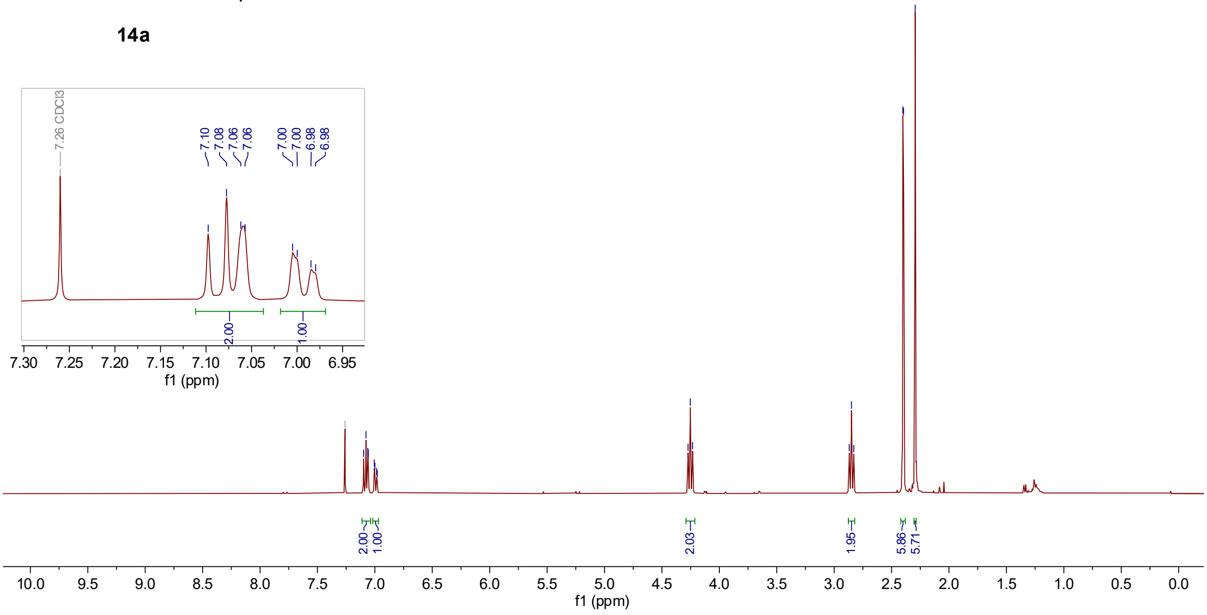

^{1}H NMR spectrum of (**14a**)

19F-NMR

19F NMR (CDCl3, 376 MHz)

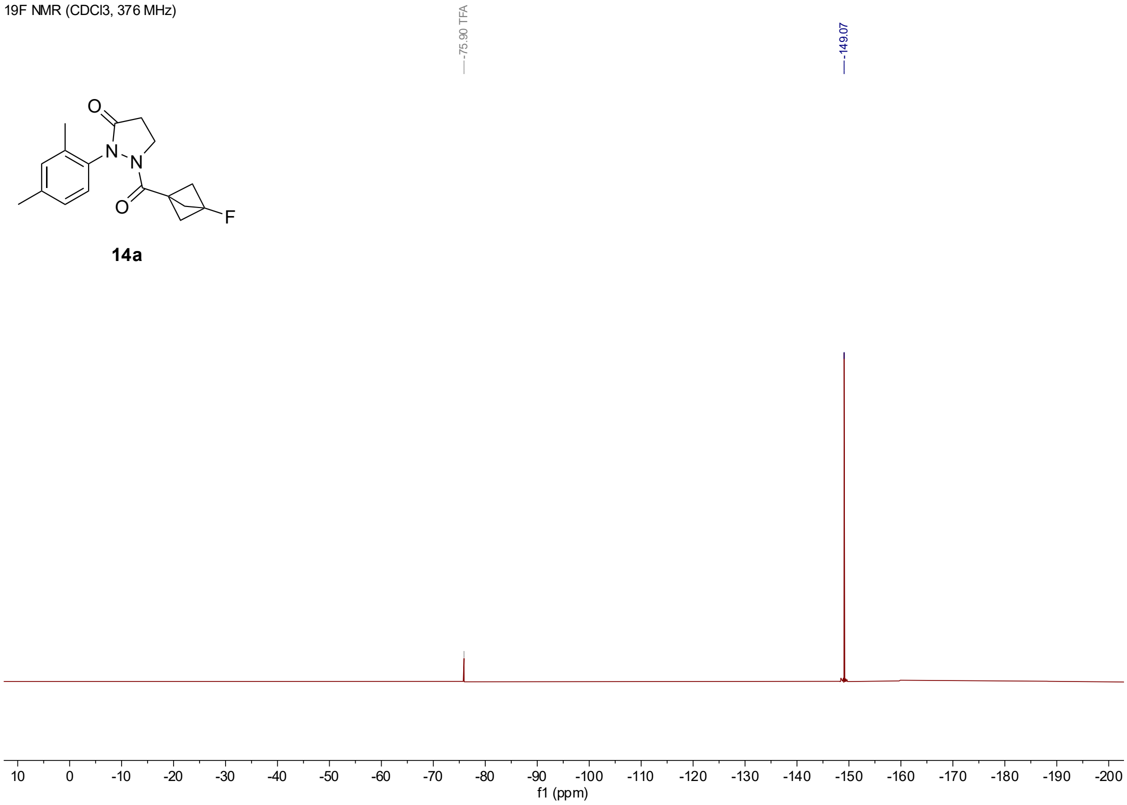

^{19}F NMR spectrum of (**14a**)

13C-NMR

13C NMR (CDCl3, 101 MHz)

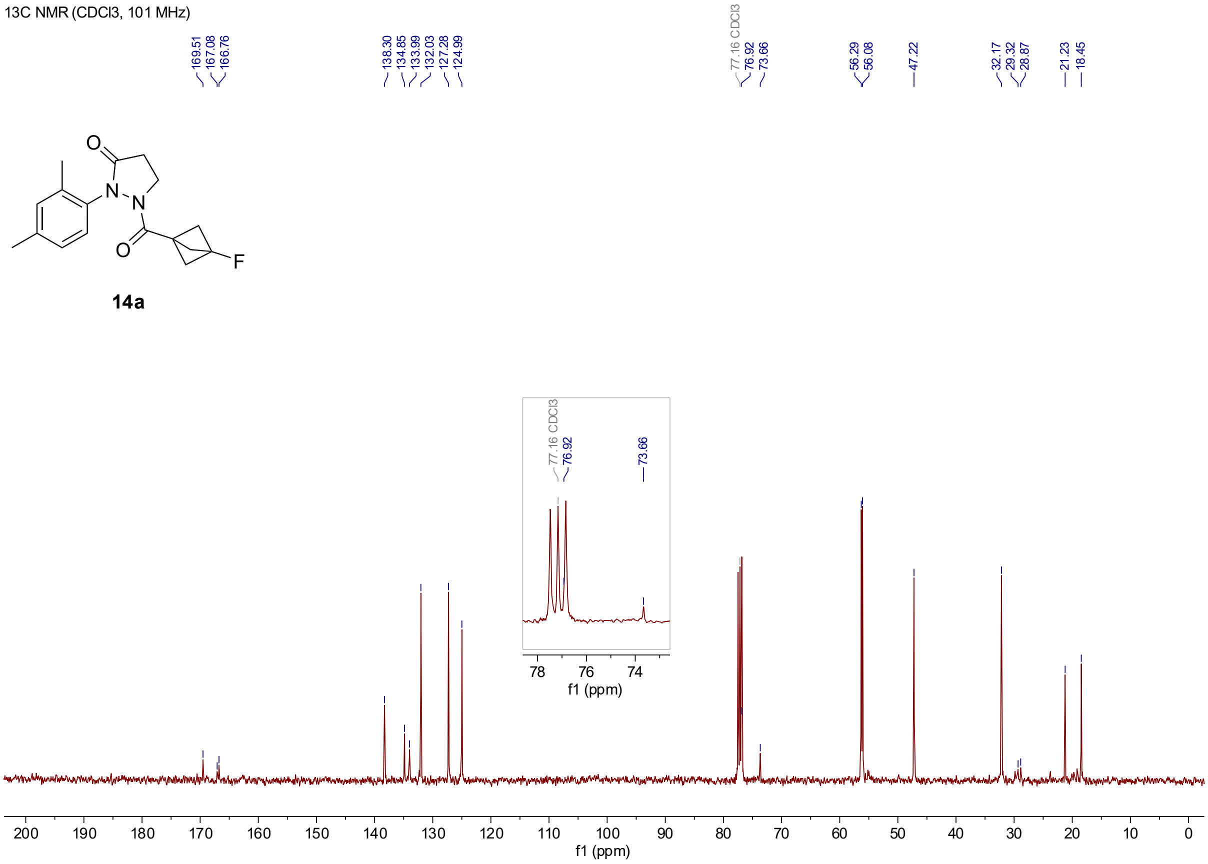

^{13}C NMR spectrum of (**14a**)

HRMS

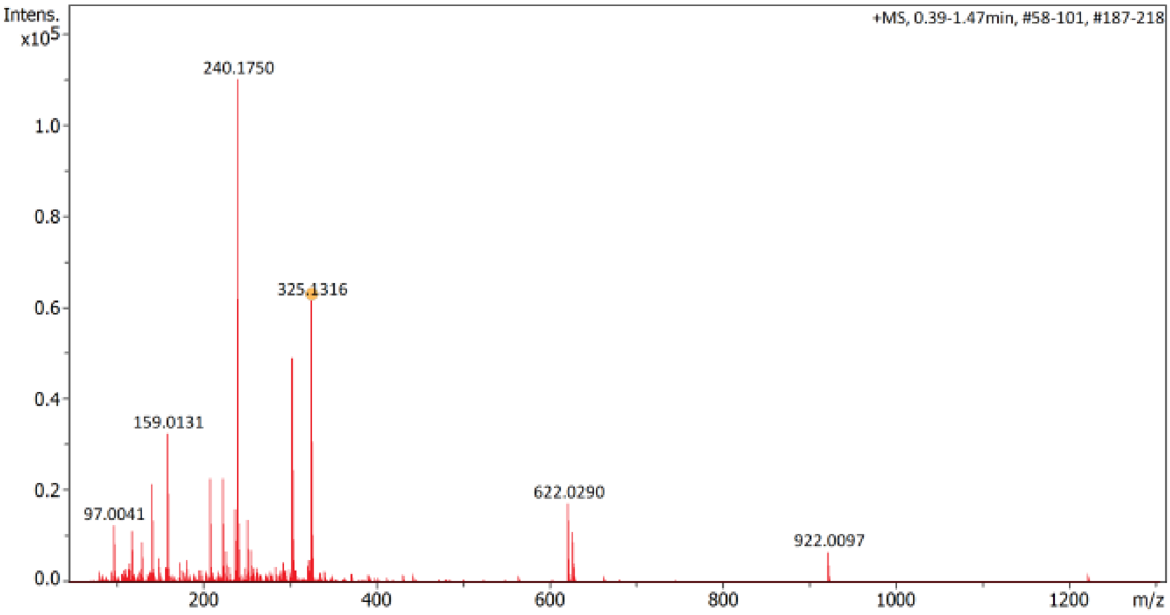

HRMS(ESI+) spectrum of (**14a**)

LC-MS

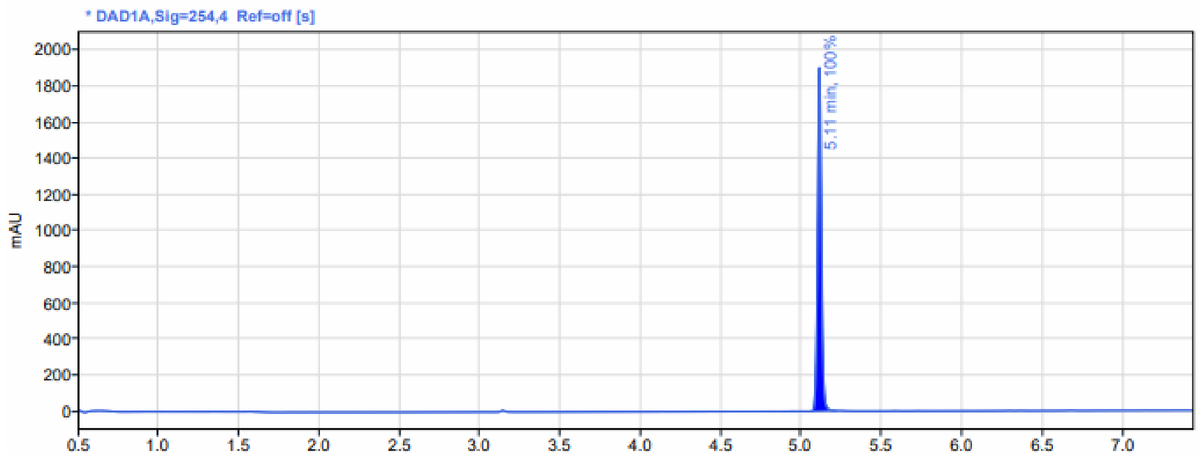

LC-MS HPLC chromatogram at 254.4 nm of (**14a**)

Characterization of (15a)

1H-NMR

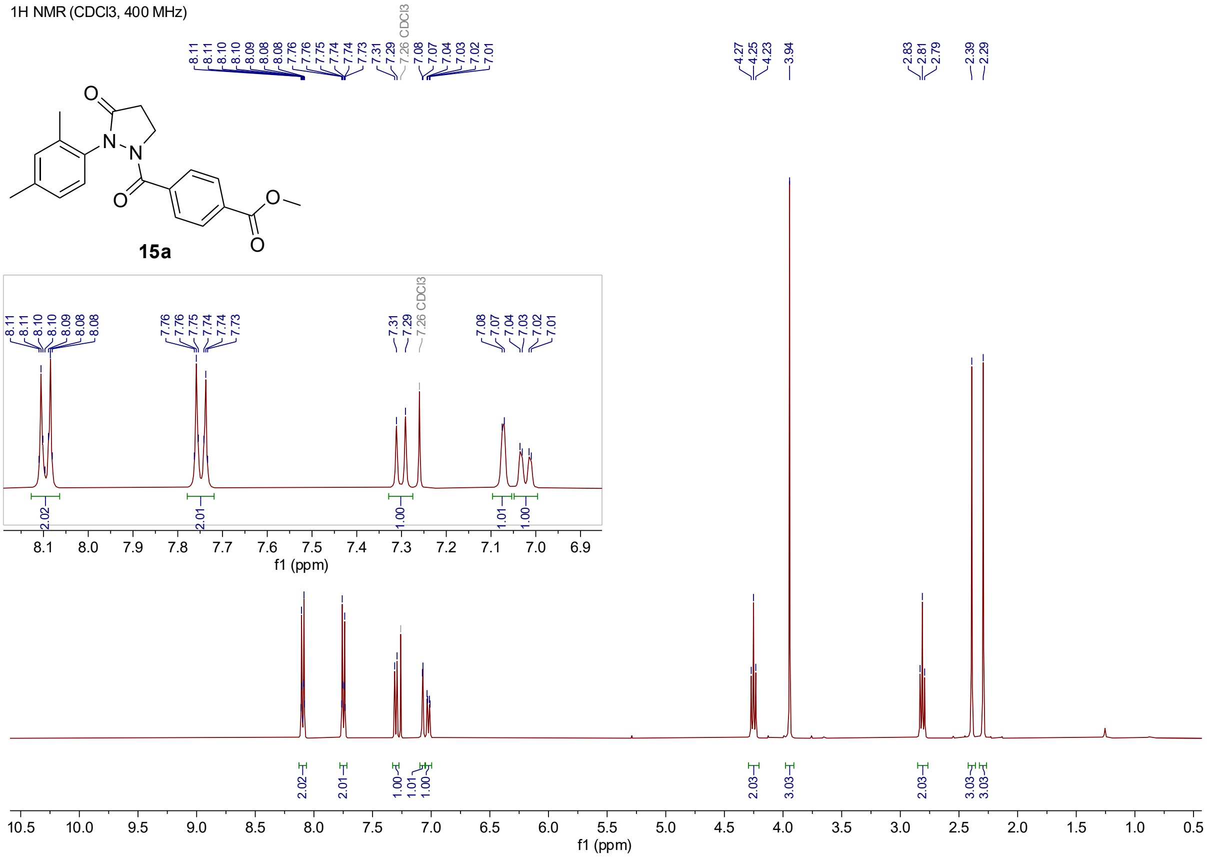

^{1}H NMR spectrum of (**15a**)

13C-NMR

13C NMR (CDCl3, 101 MHz)

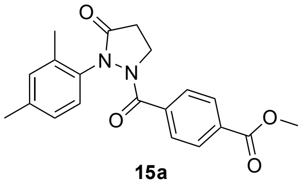

15a

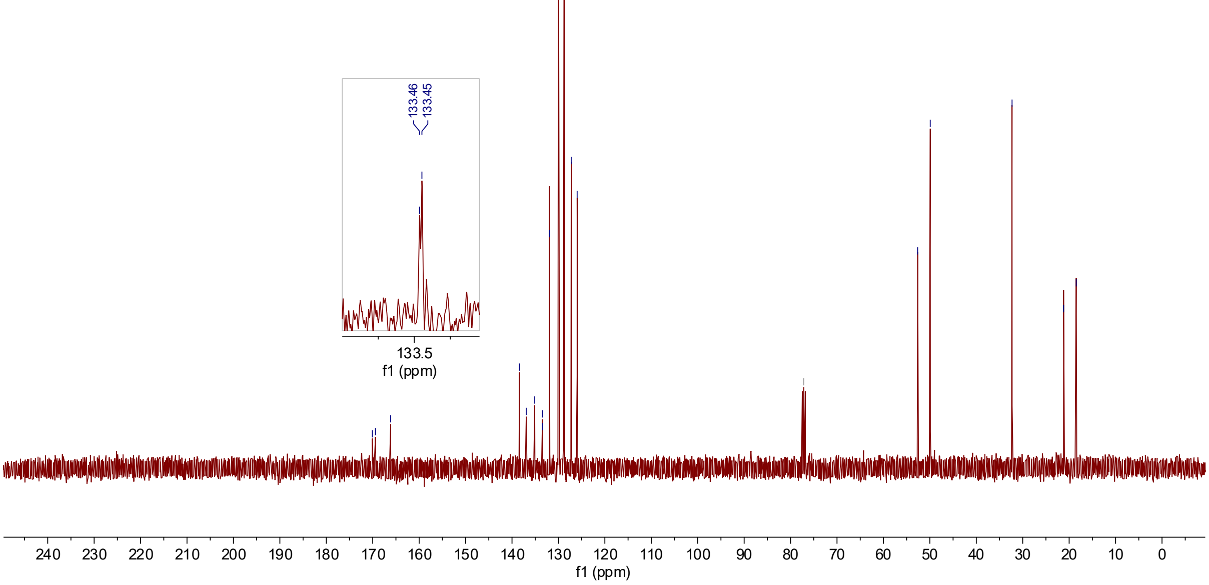

^{13}C NMR spectrum of (**15a**)

HRMS

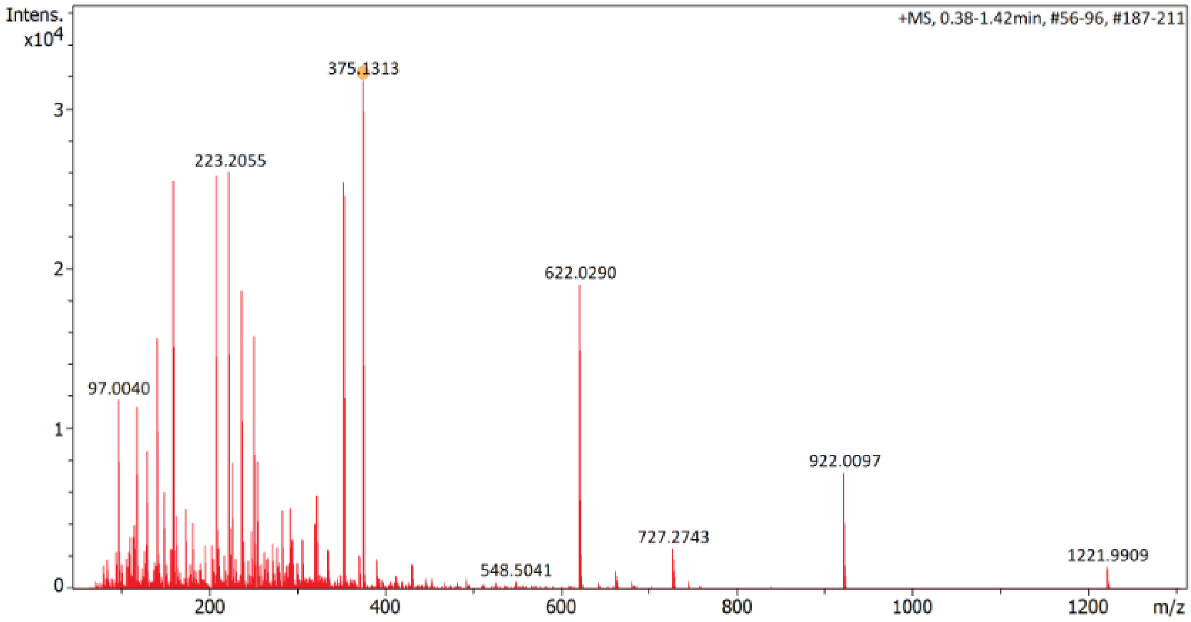

HRMS(ESI+) spectrum of (**15a**)

LC-MS

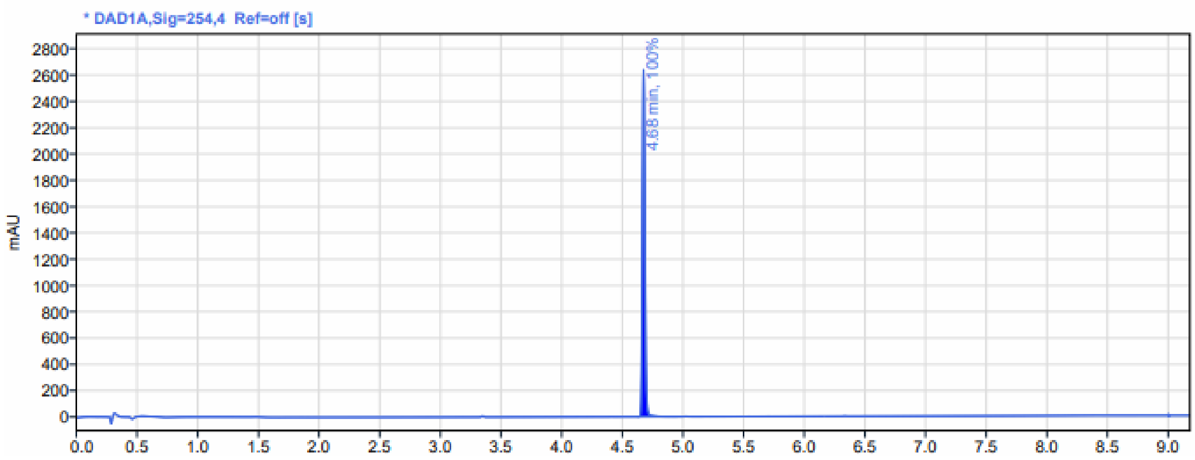

LC-MS HPLC chromatogram at 254.4 nm of (**15a**)

Characterization of (16a)

1H-NMR

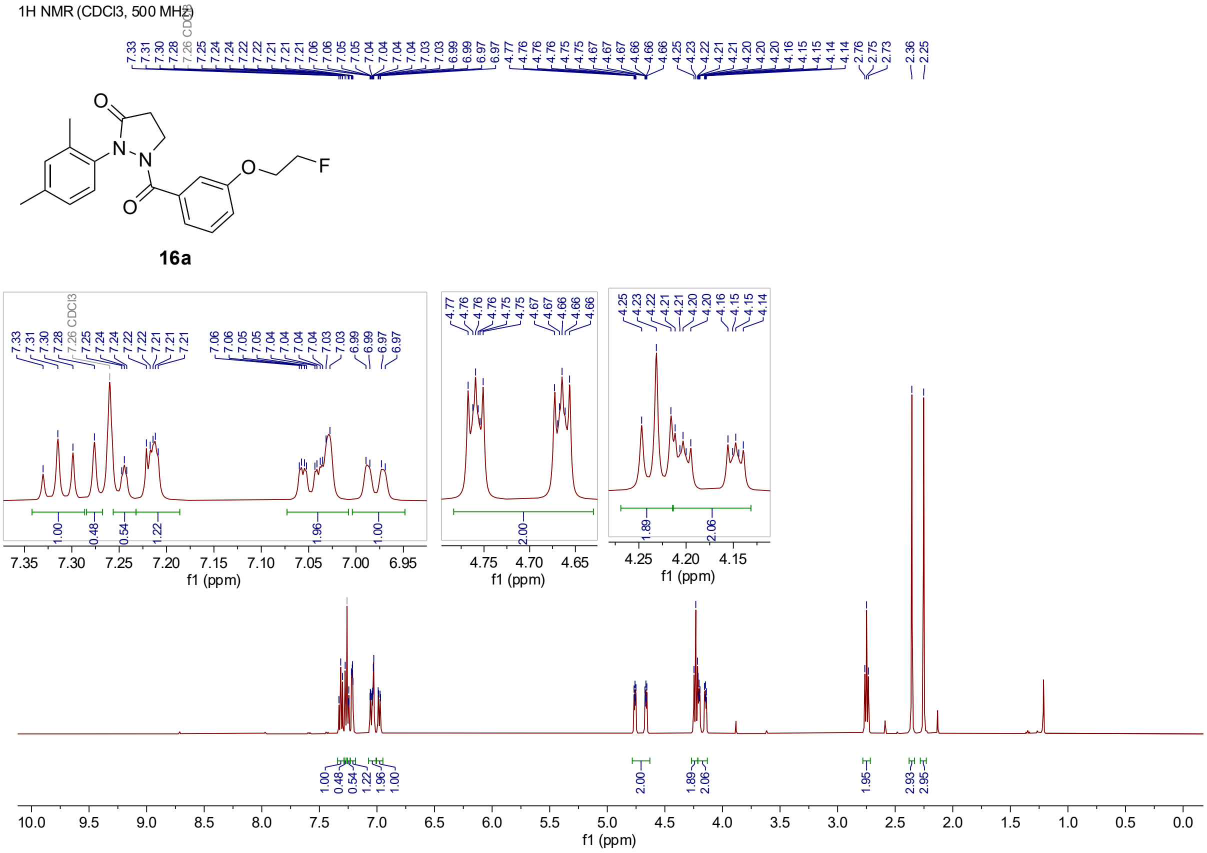

^{1}H NMR spectrum of (**16a**)

19F-NMR

19F NMR (CDCl3, 376 MHz)

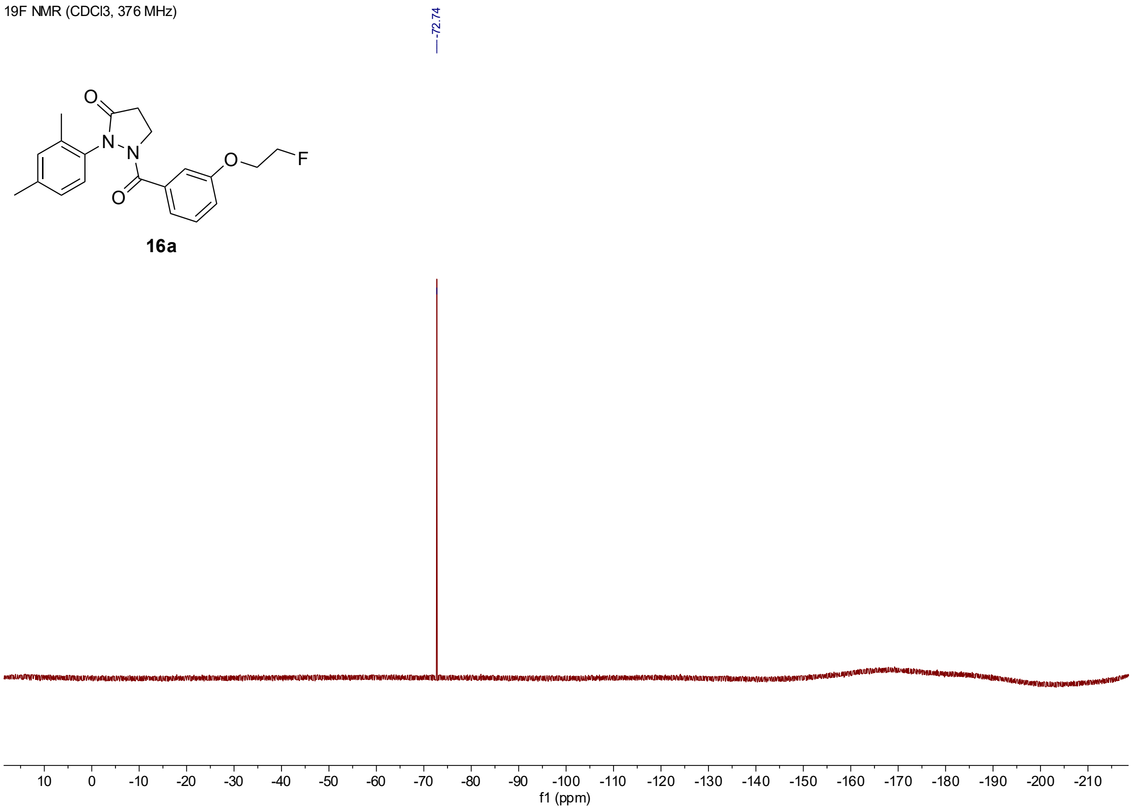

^{19}F NMR spectrum of (**16a**)

13C-NMR

13C NMR (CDCl3, 126 MHz)

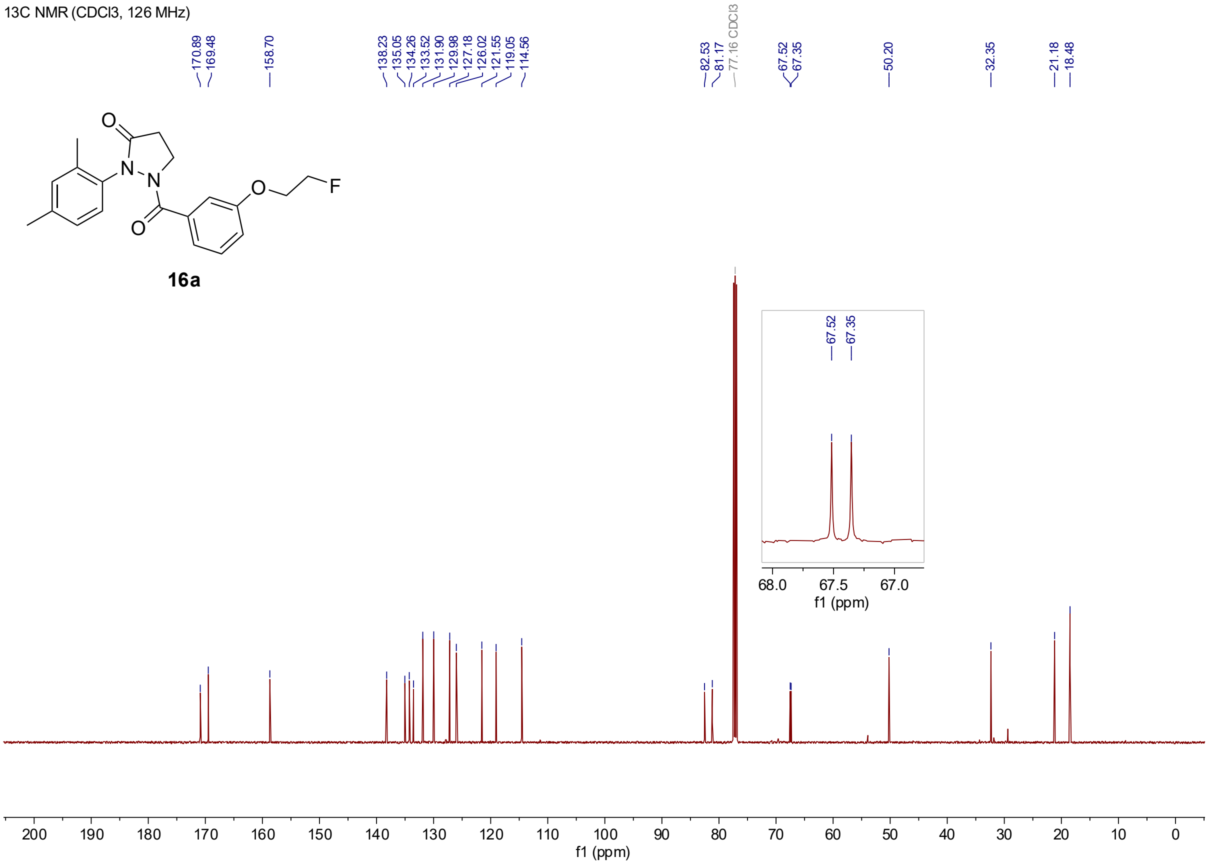

^{13}C NMR spectrum of (**16a**)

HRMS

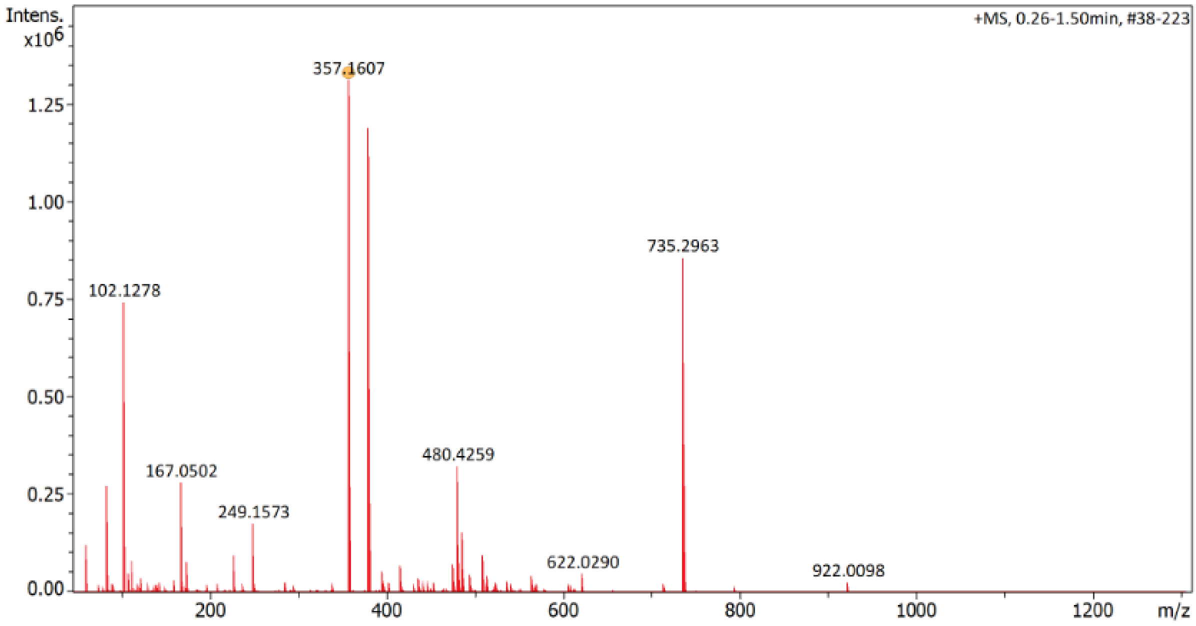

HRMS(ESI+) spectrum of (**16a**)

LC-MS

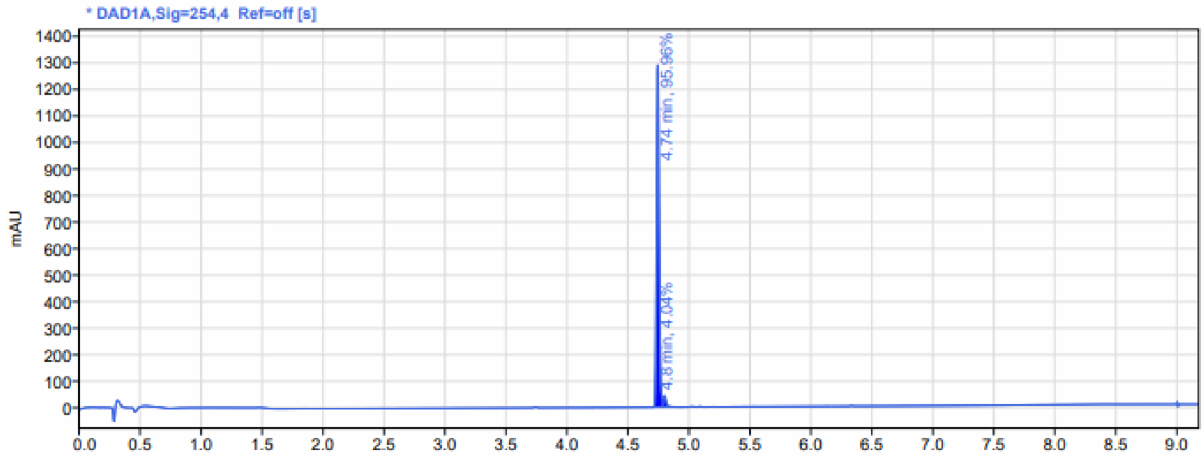

LC-MS HPLC chromatogram at 254.4 nm of (**16a**)

Characterization of (18)

1H-NMR

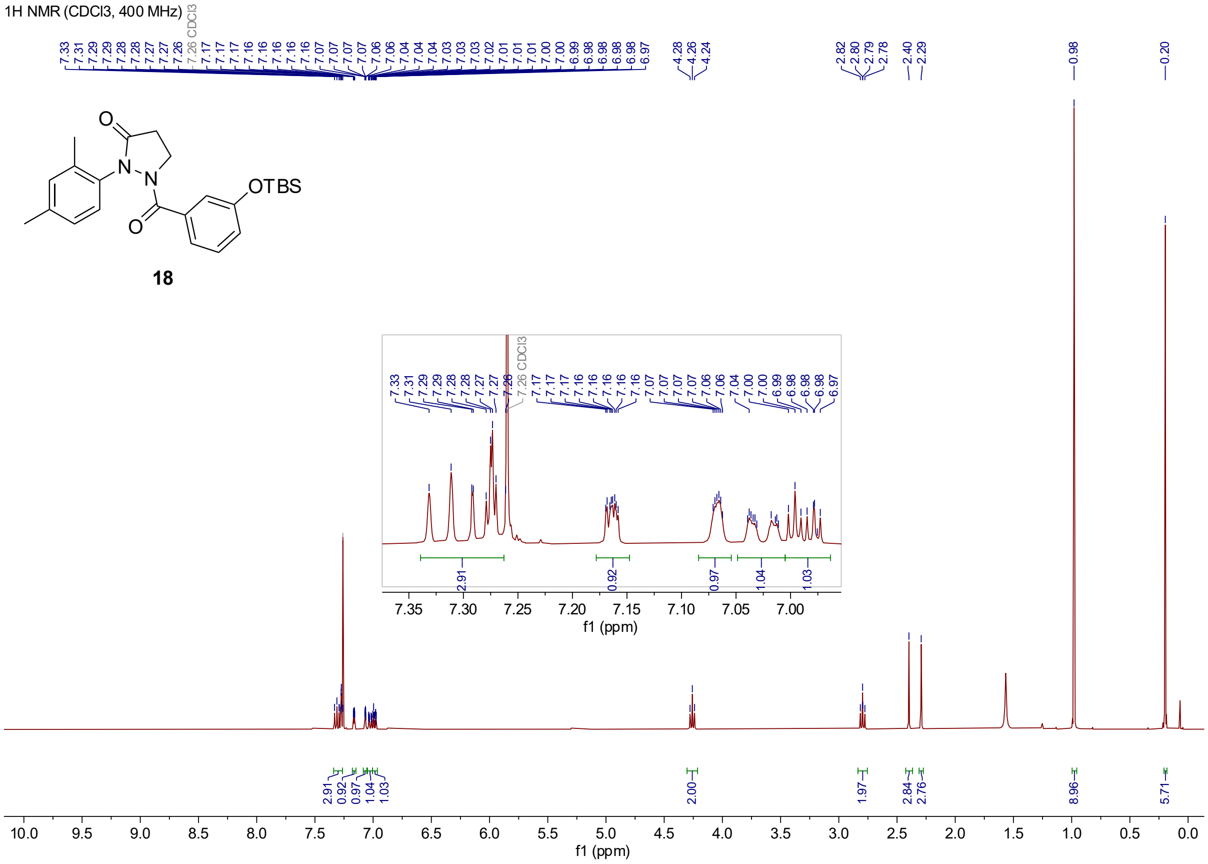

18

^{1}H NMR spectrum of (**18**)

13C-NMR

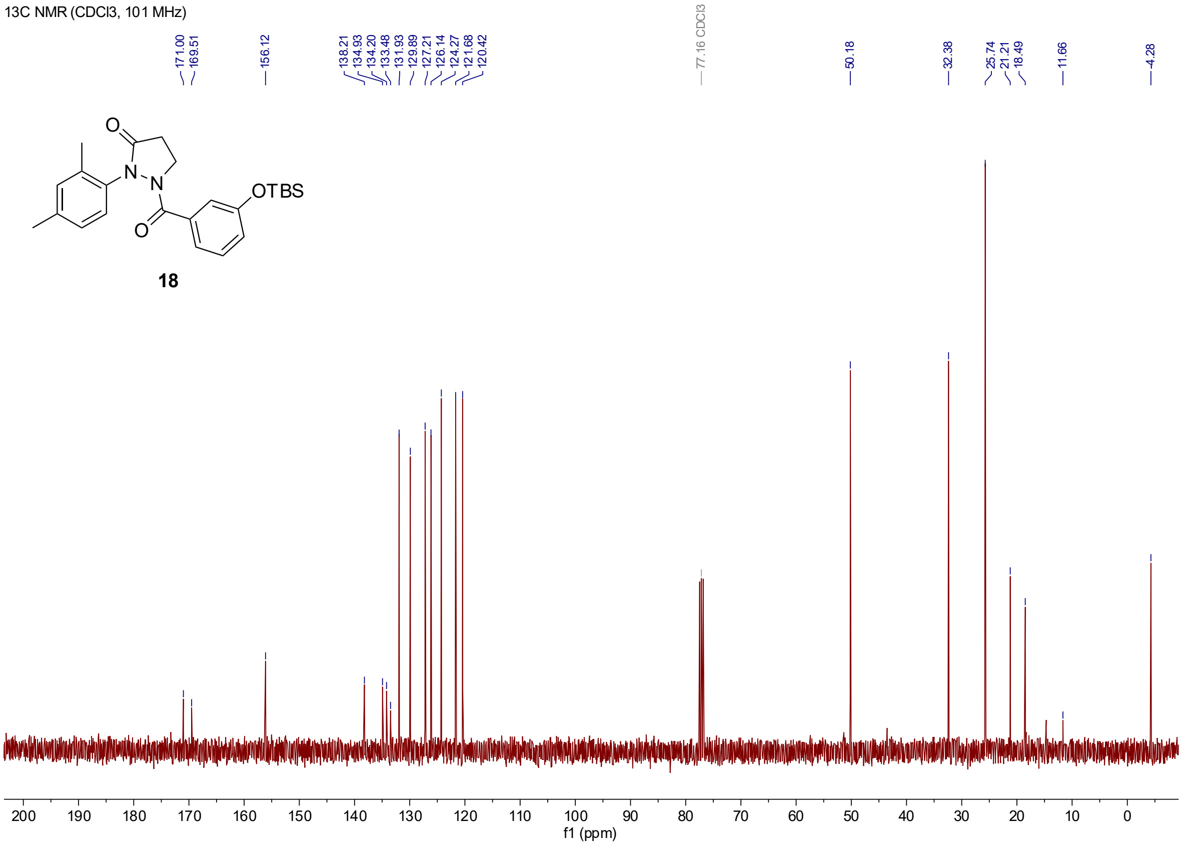

^{13}C NMR spectrum of (**18**)

HRMS

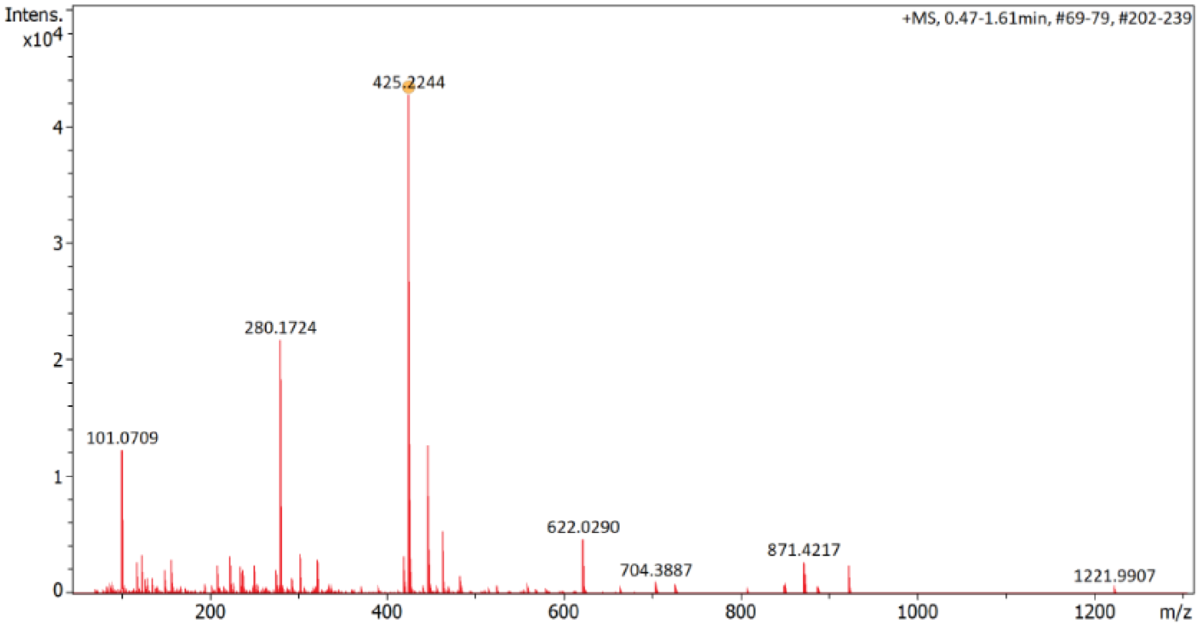

HRMS(ESI+) spectrum of (**18**)

Characterization of (19)

1H-NMR

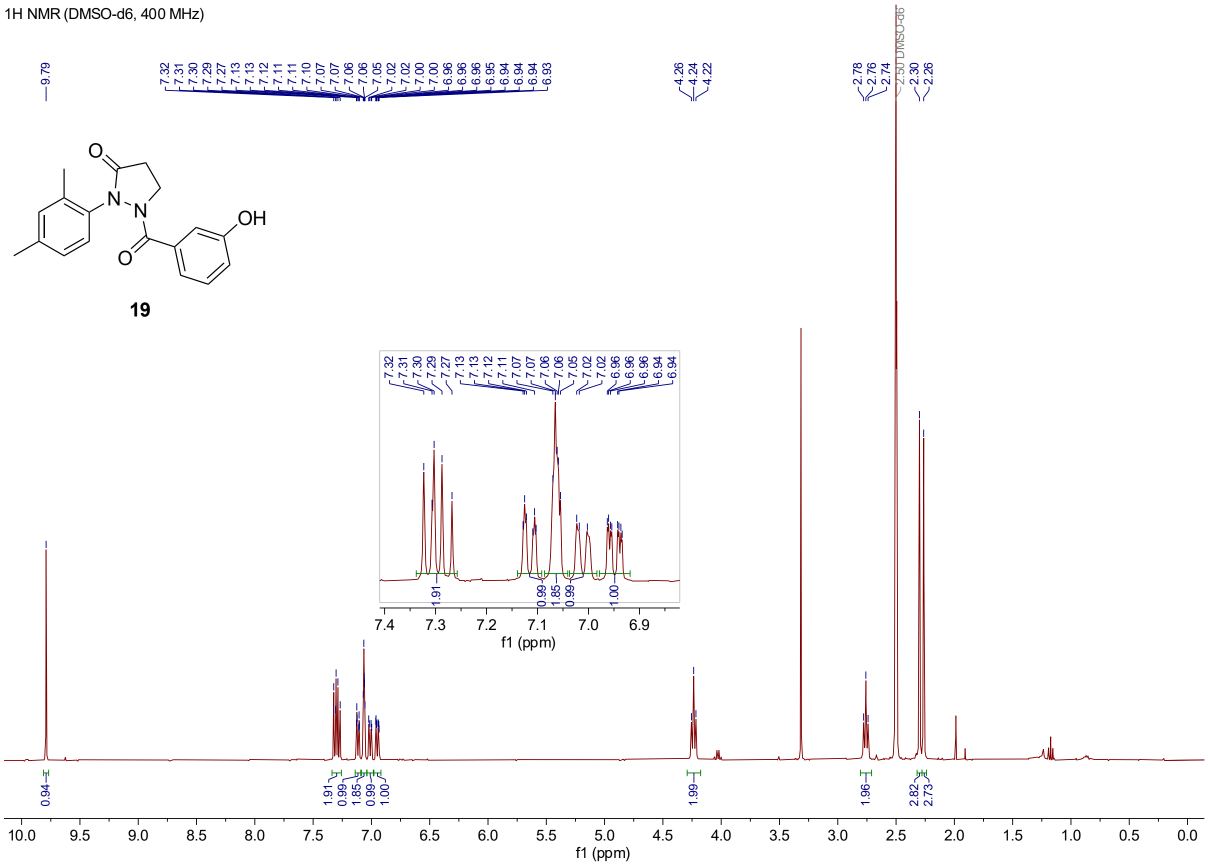

19

^{1}H NMR spectrum of (**19**)

13C-NMR

13C NMR (DMSO-d6, 101 MHz)

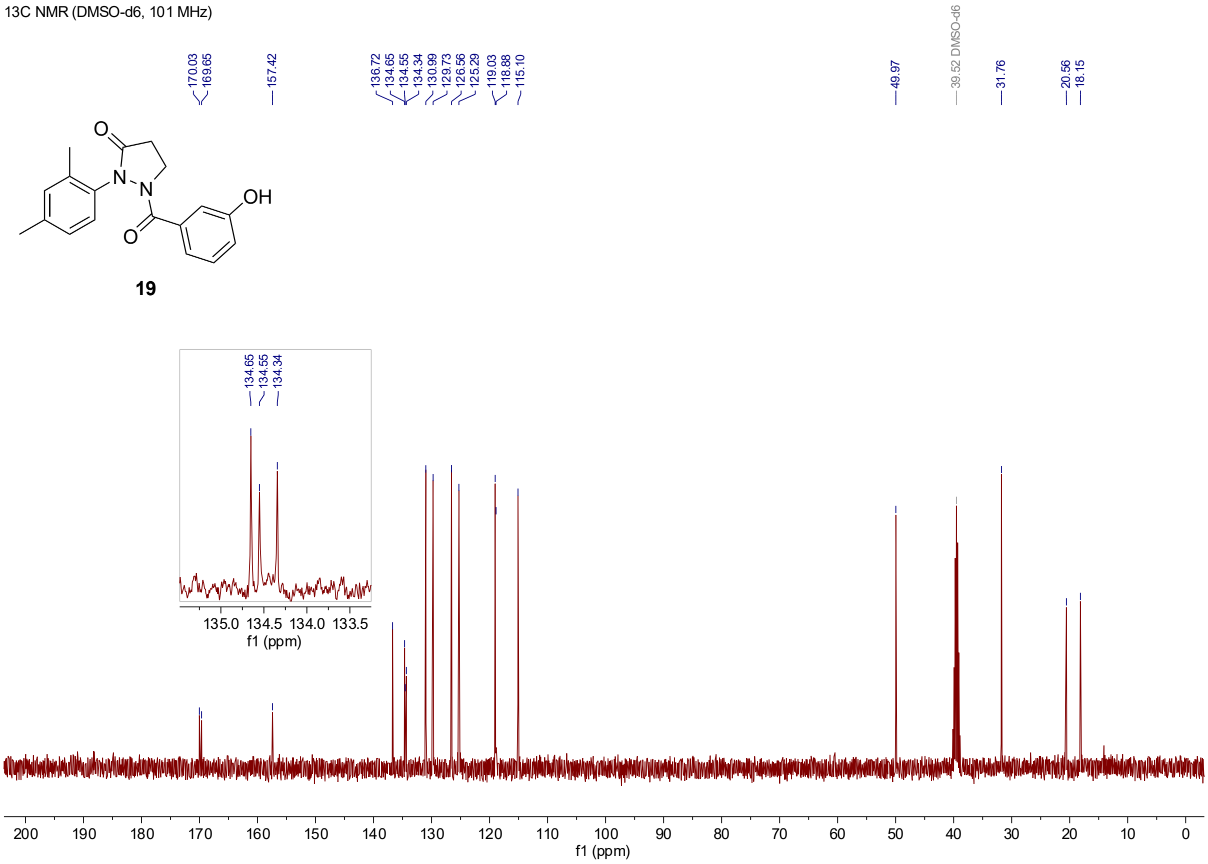

19

^{13}C NMR spectrum of (**19**)

HRMS

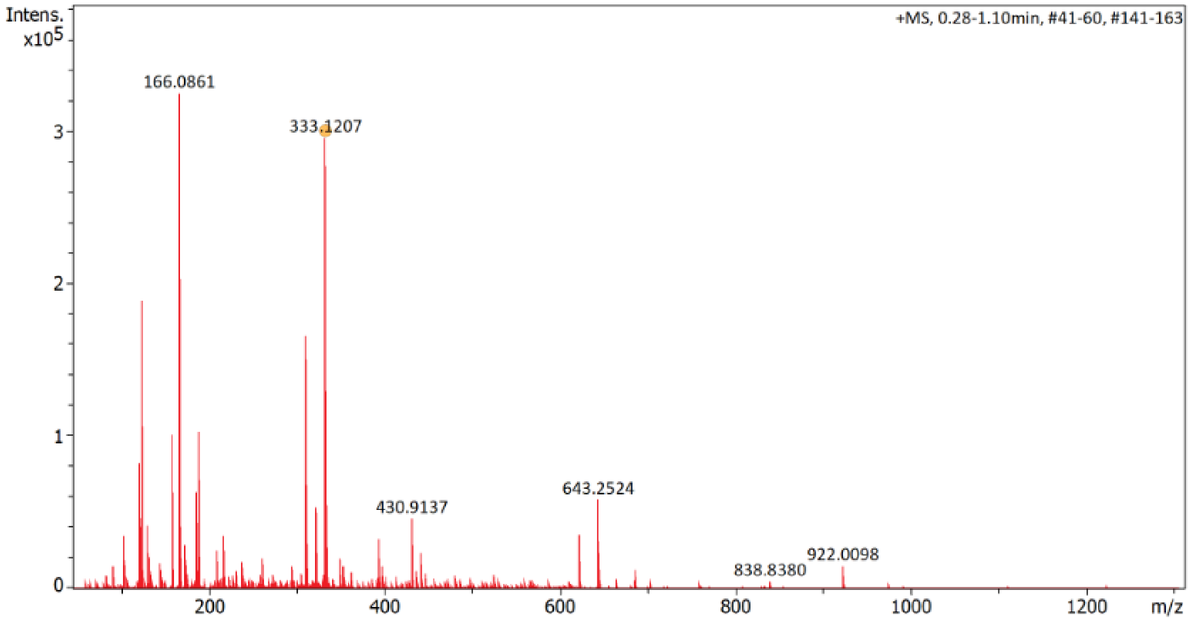

HRMS(ESI+) spectrum of (**19**)

Characterization of (6b)

1H-NMR

1H NMR (CDCl3, 400 MHz)

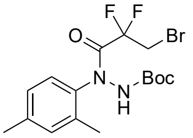

6b

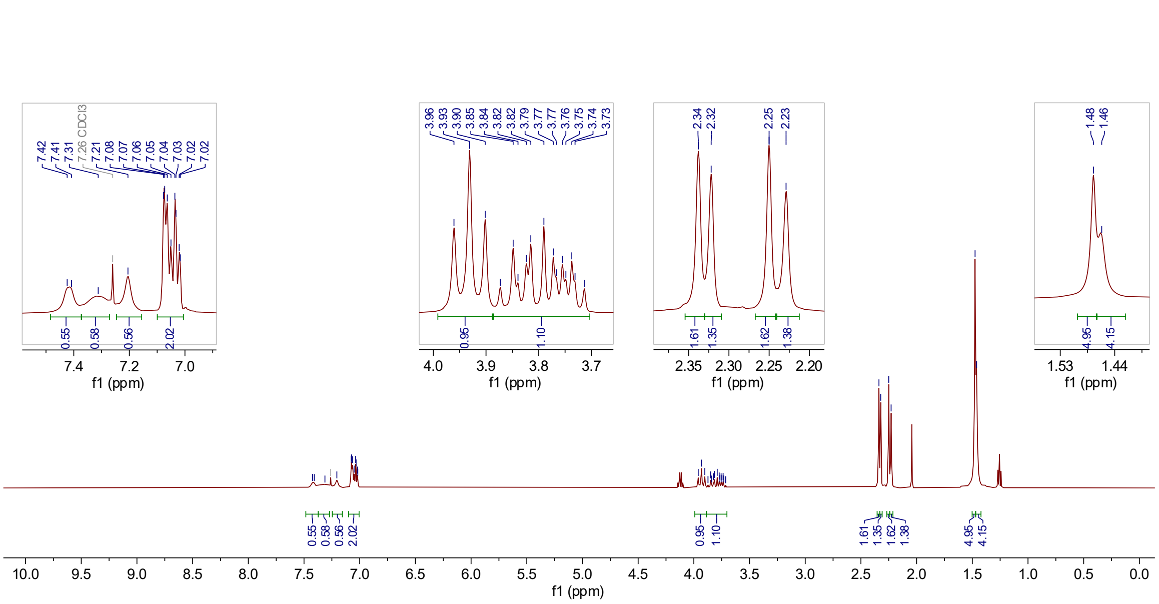

^{1}H NMR spectrum of (**6b**)

19F-NMR

19F NMR (CDCl3, 471 MHz)

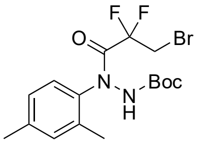

6b

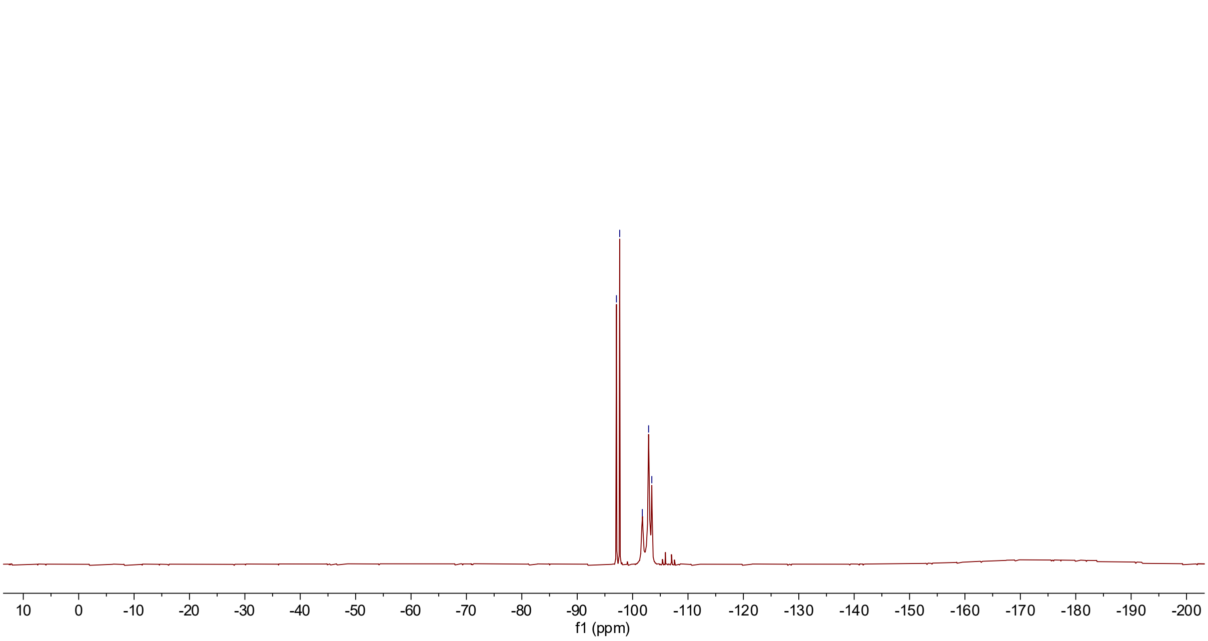

^{19}F NMR spectrum of (**6b**)

13C-NMR

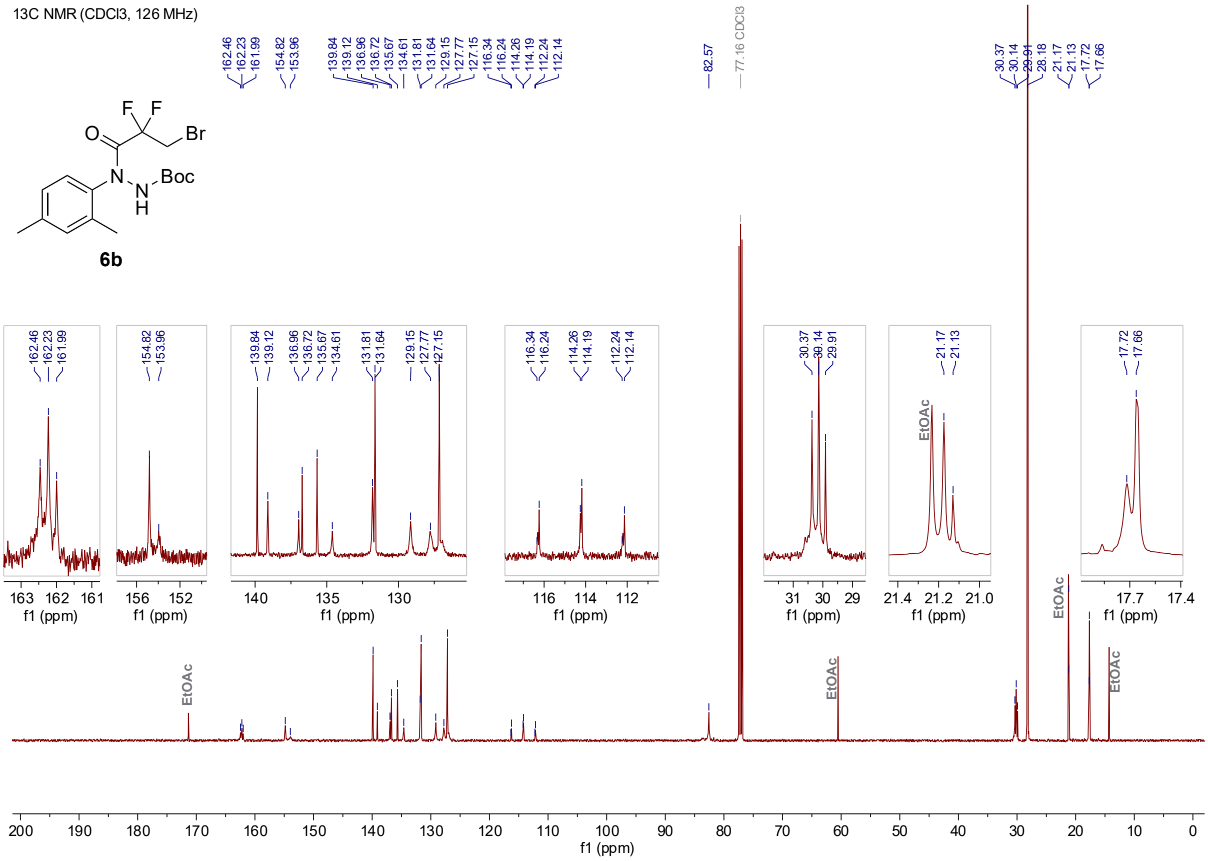

^{13}C NMR spectrum of (**6b**)

HRMS

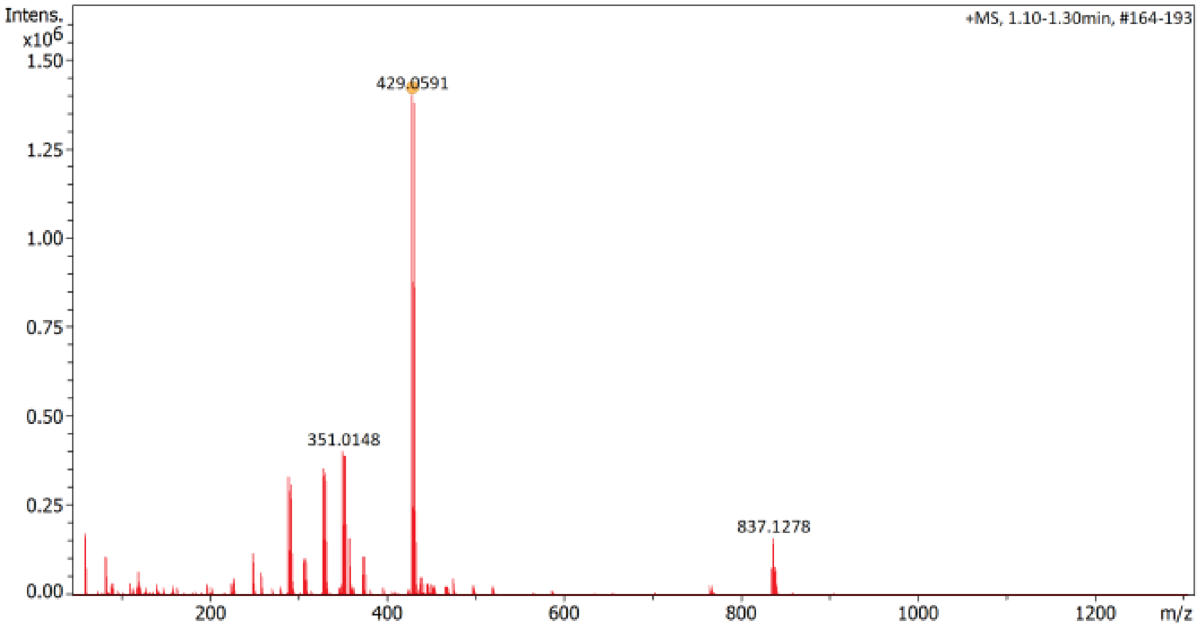

HRMS(ESI+) spectrum of (**6b**)

LC-MS

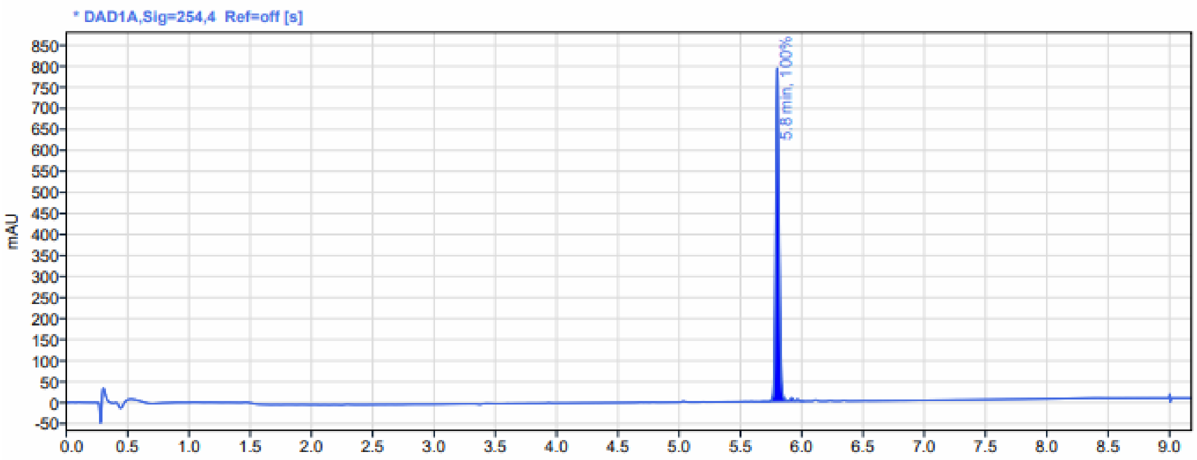

LC-MS HPLC chromatogram at 254.4 nm of (**6b**)

Characterization of (7b)

1H-NMR

1H NMR (CDCl3, 400 MHz)

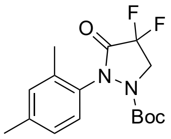

7b

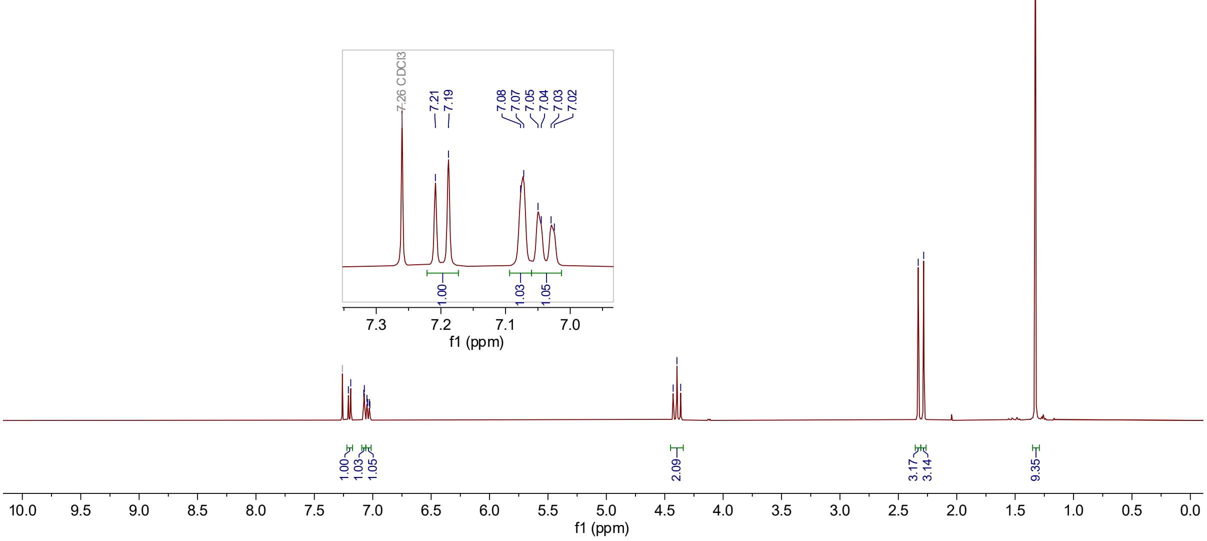

^{1}H NMR spectrum of (**7b**)

19F-NMR

19F NMR (CDCl3, 376 MHz)

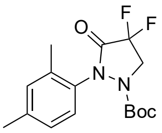

7b

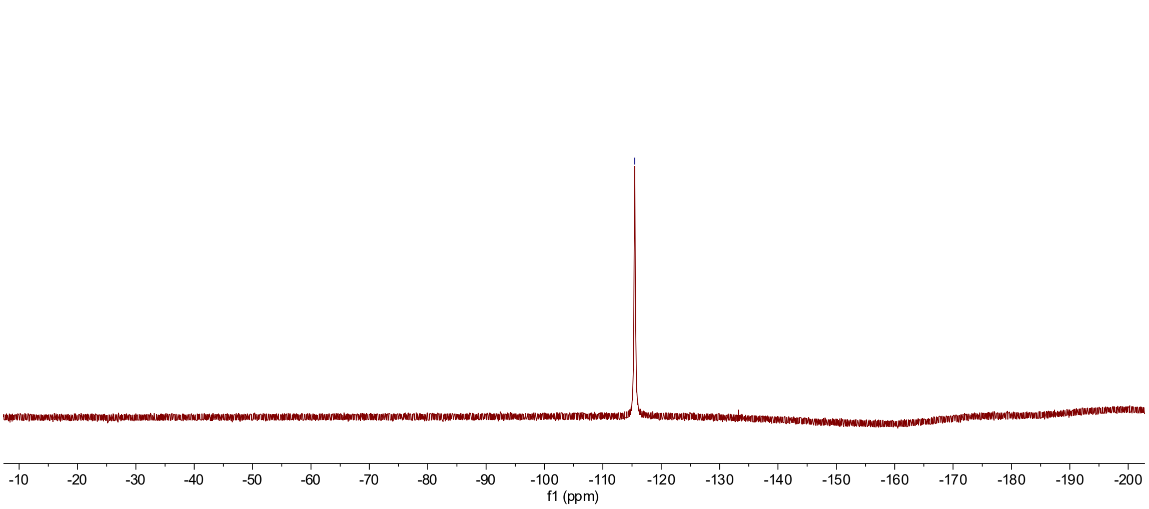

^{19}F NMR spectrum of (**7b**)

13C-NMR

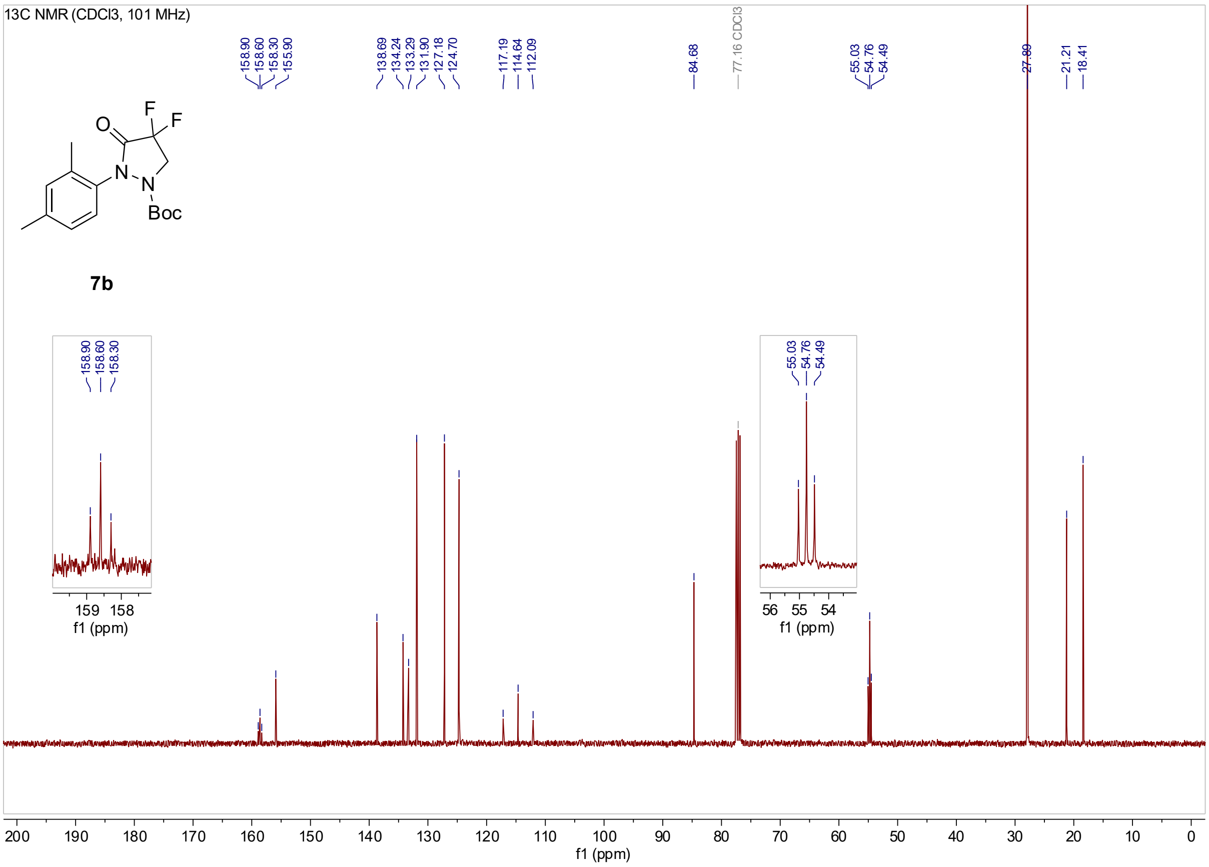

^{13}C NMR spectrum of (**7b**)

HRMS

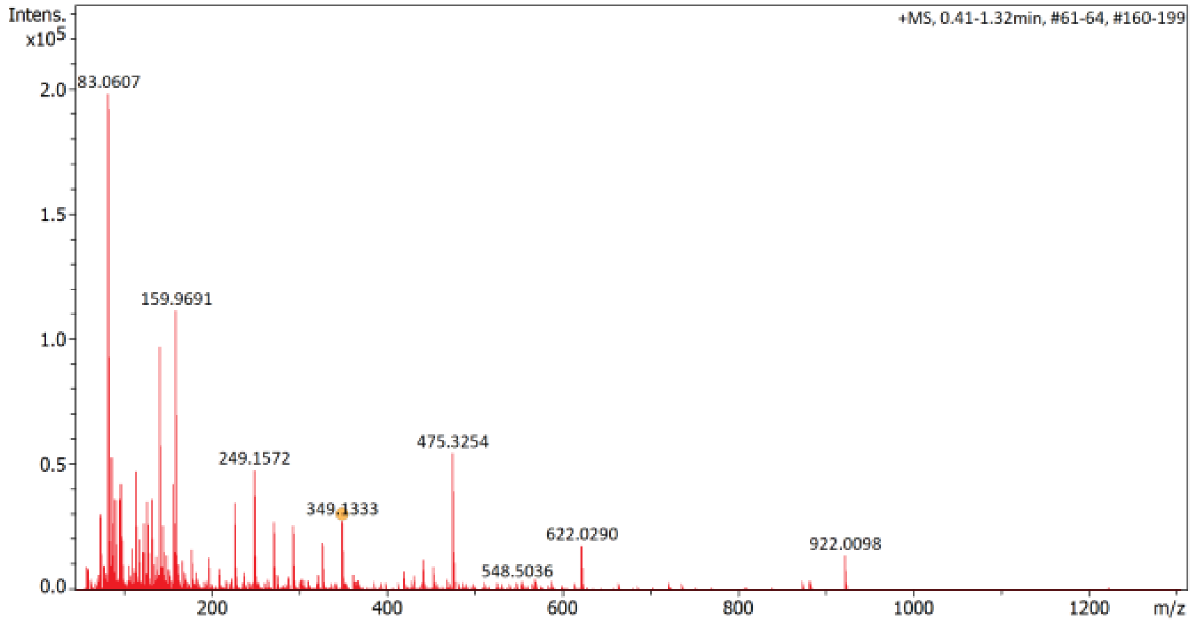

HRMS(ESI+) spectrum of (**7b**)

Characterization of (8b)

1H-NMR

1H NMR (CDCl3, 400 MHz)

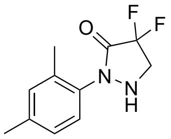

8b

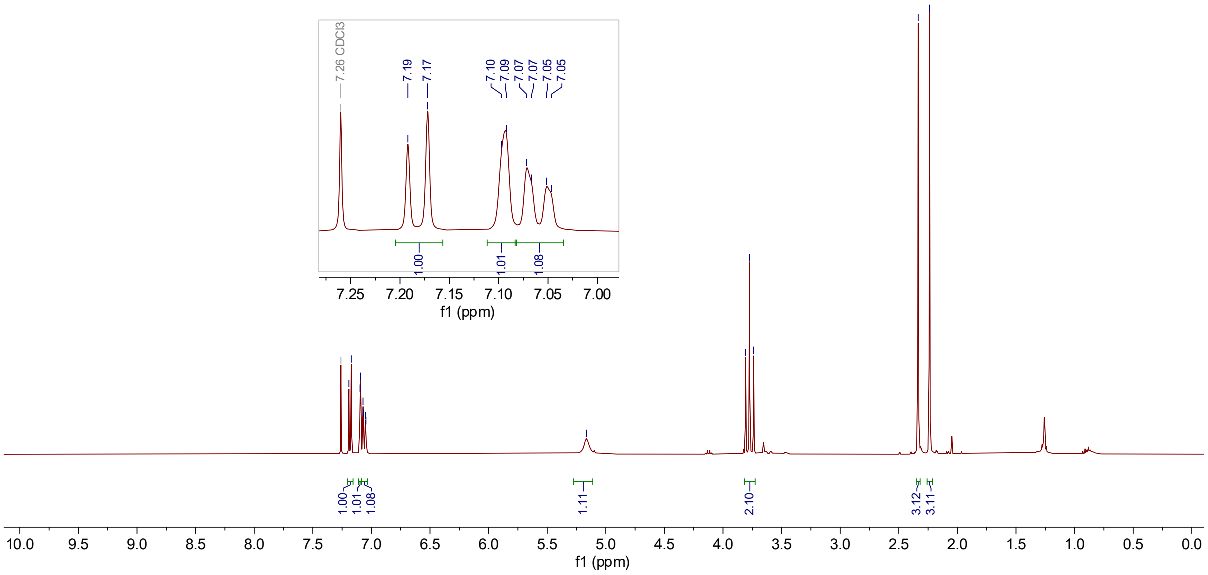

^{1}H NMR spectrum of (**8b**)

19F-NMR

19F NMR (CDCl3, 376 MHz)

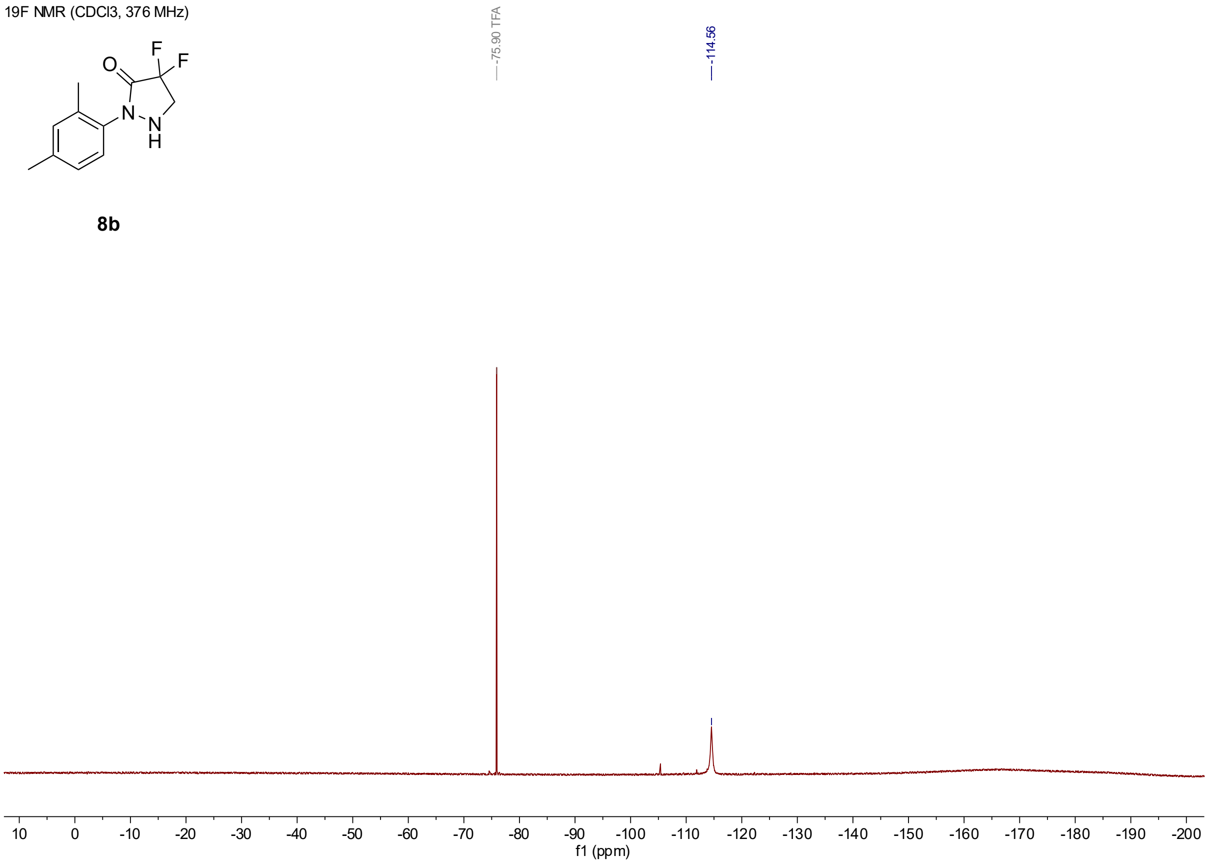

^{19}F NMR spectrum of (**8b**)

13C-NMR

13C NMR (CDCl3, 126 MHz)

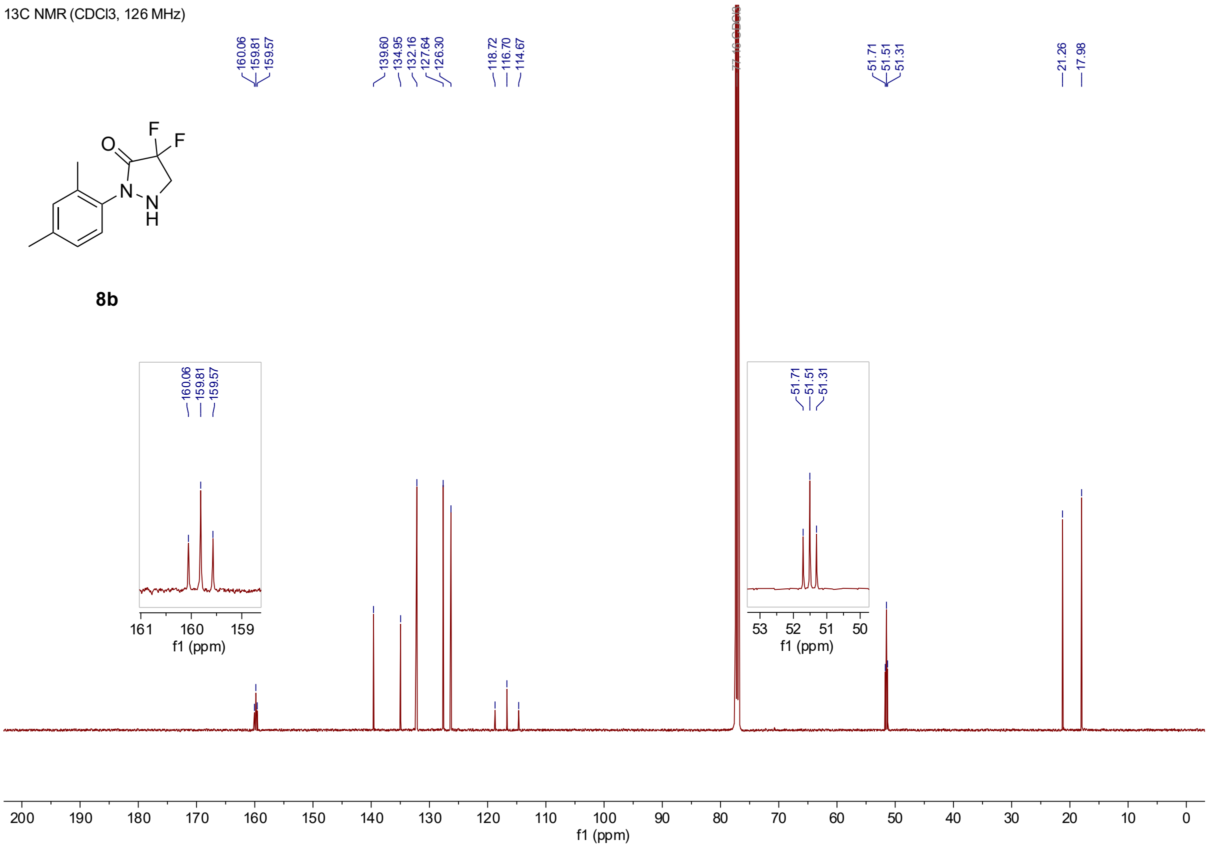

^{13}C NMR spectrum of (**8b**)

HRMS

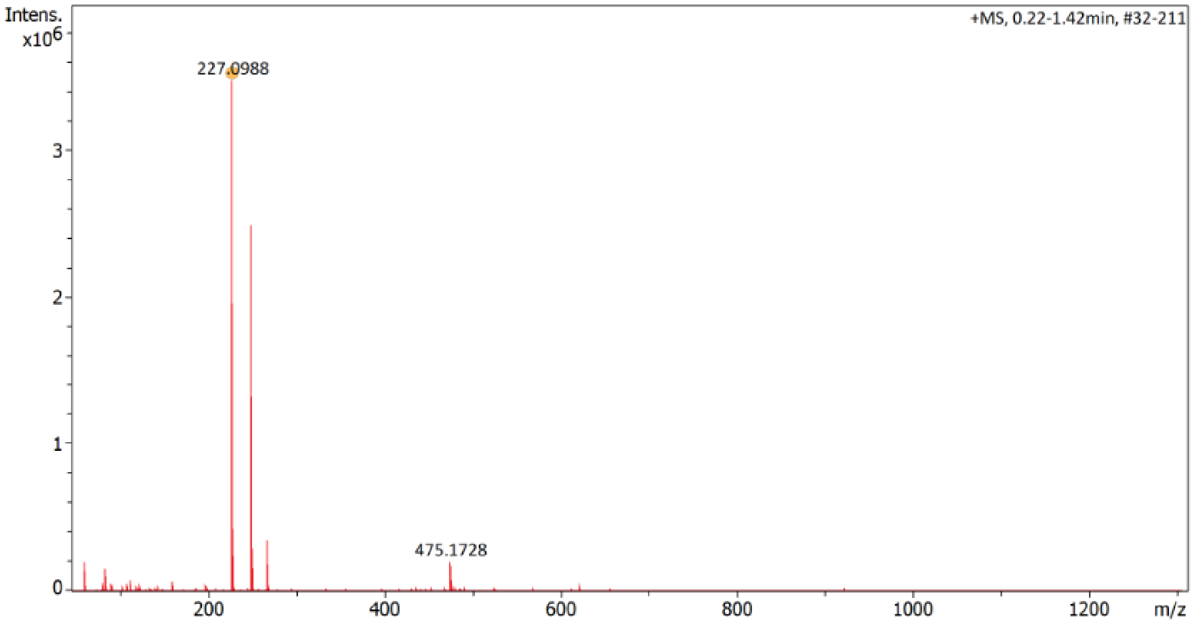

HRMS(ESI+) spectrum of (**8b**)

Characterization of (9b)

1H-NMR

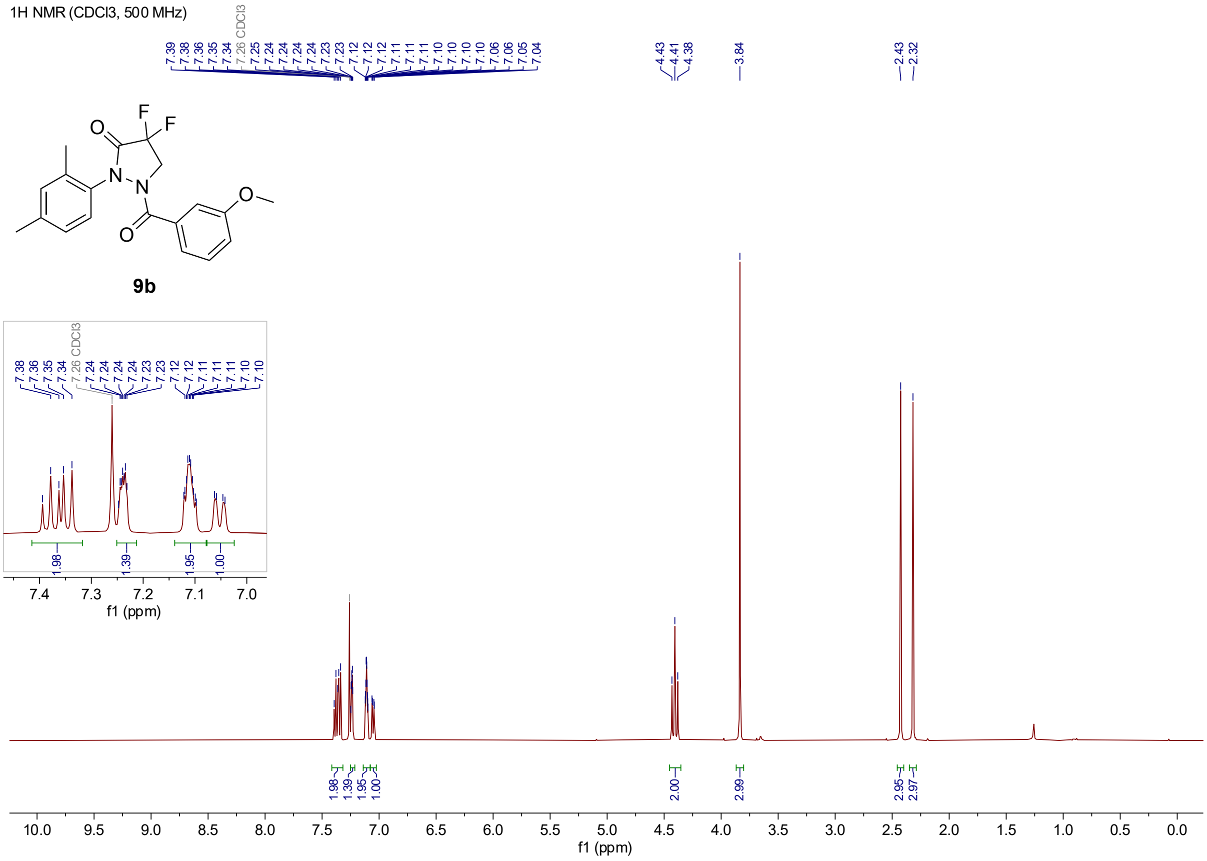

^{1}H NMR spectrum of (**9b**)

19F-NMR

19F NMR (CDCl3, 471 MHz)

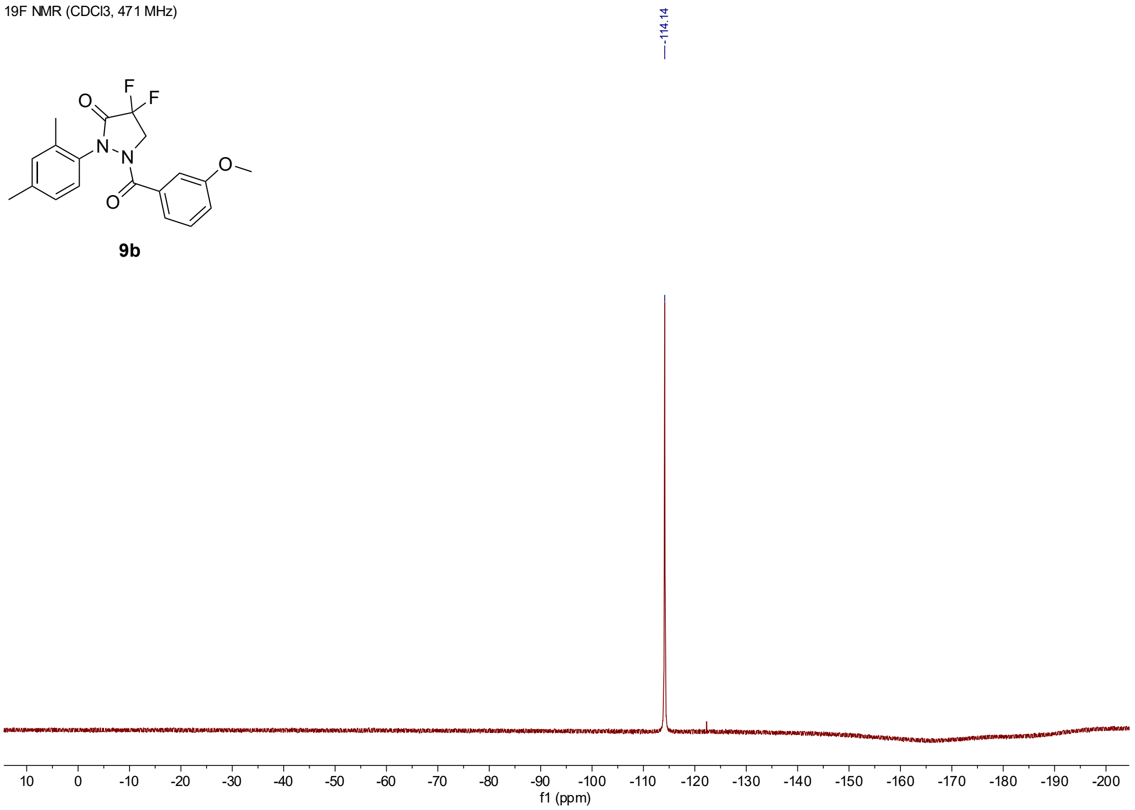

^{19}F NMR spectrum of (**9b**)

13C-NMR

13C NMR (CDCl3, 126 MHz)

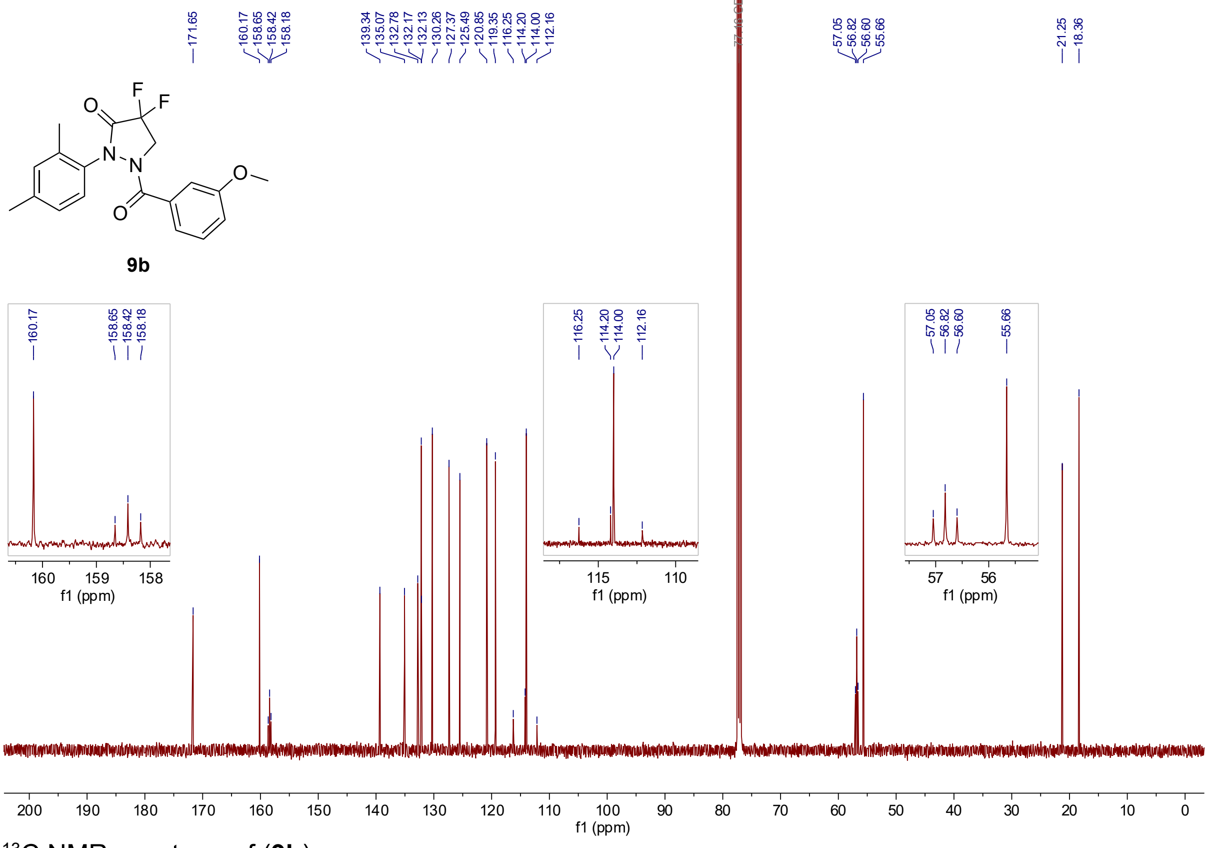

^{13}C NMR spectrum of (**9b**)

HRMS

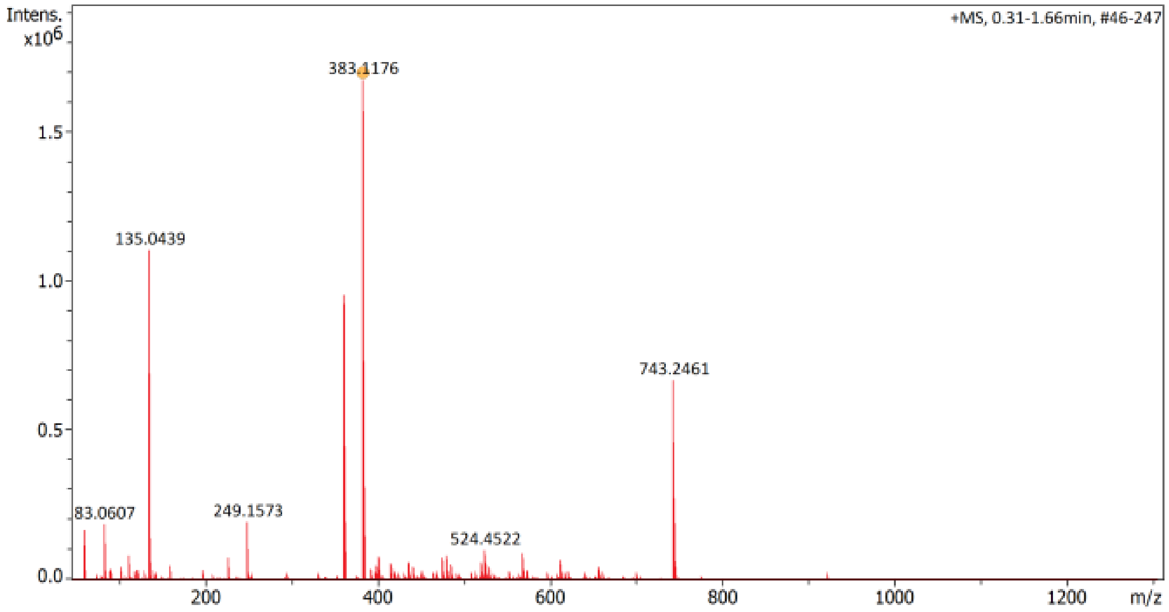

HRMS(ESI+) spectrum of (**9b**)

LC-MS

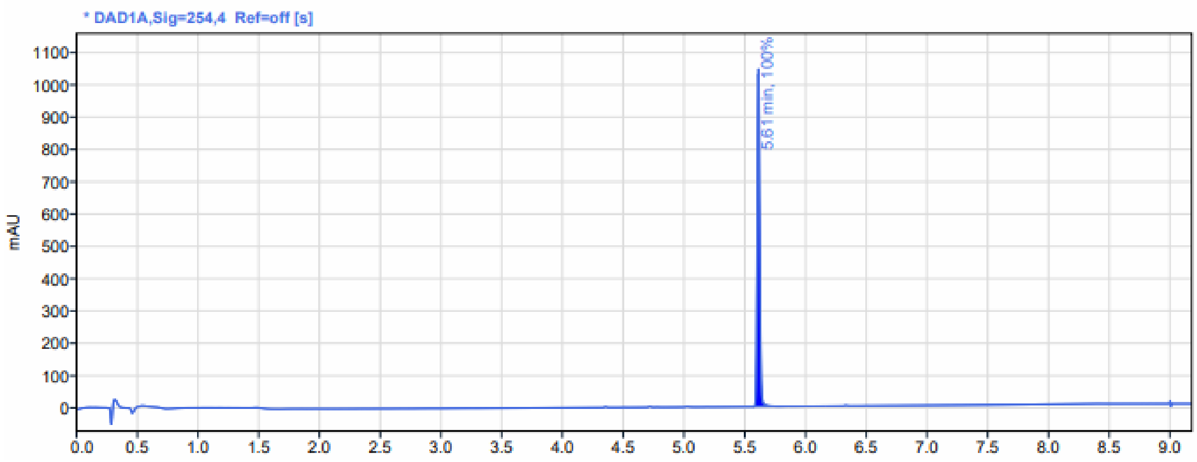

LC-MS HPLC chromatogram at 254.4 nm of (**9b**)

Characterization of (10b)

1H-NMR

1H NMR(CDCl3, 500 MHz)

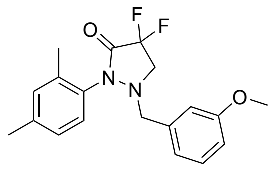

10b

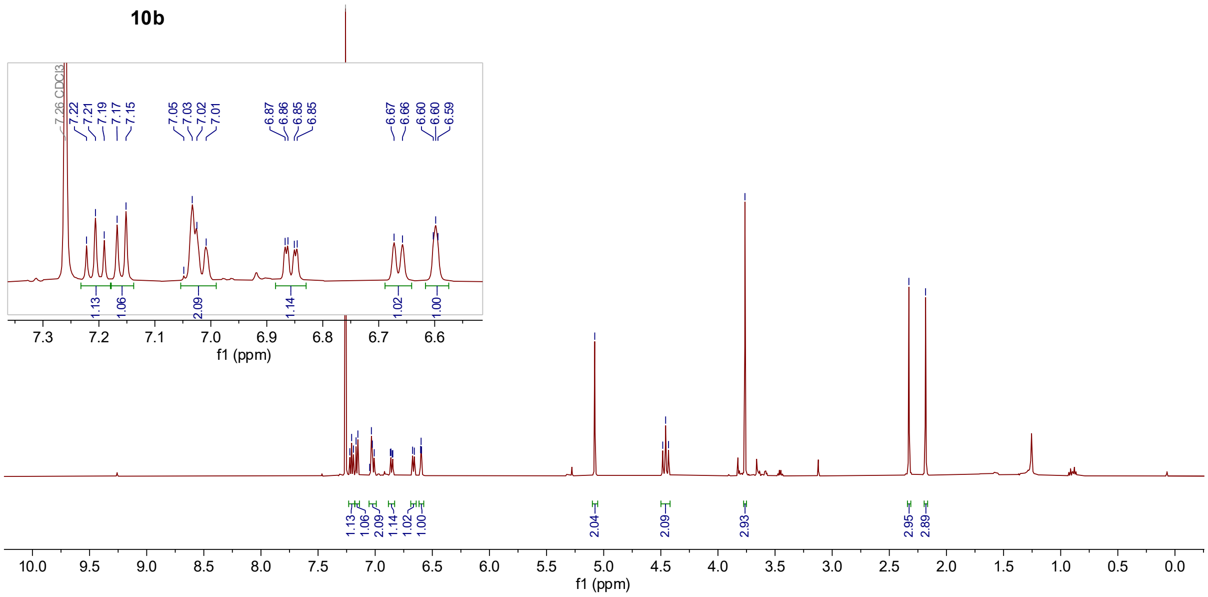

^{1}H NMR spectrum of (**10b**)

19F-NMR

19F NMR (CDCl3, 471 MHz)

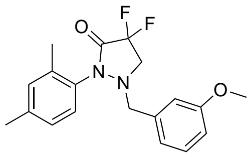

10b

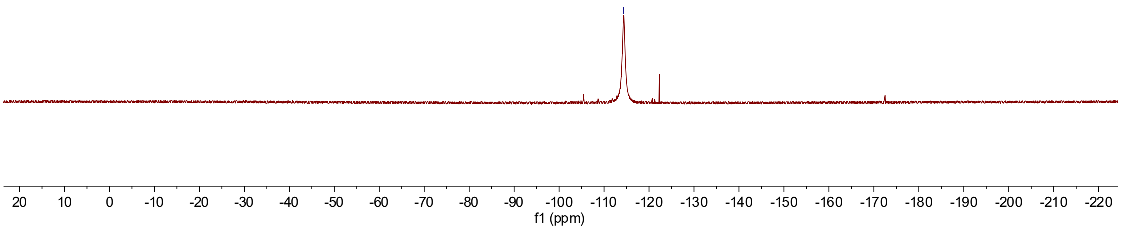

^{19}F NMR spectrum of (**10b**)

13C-NMR

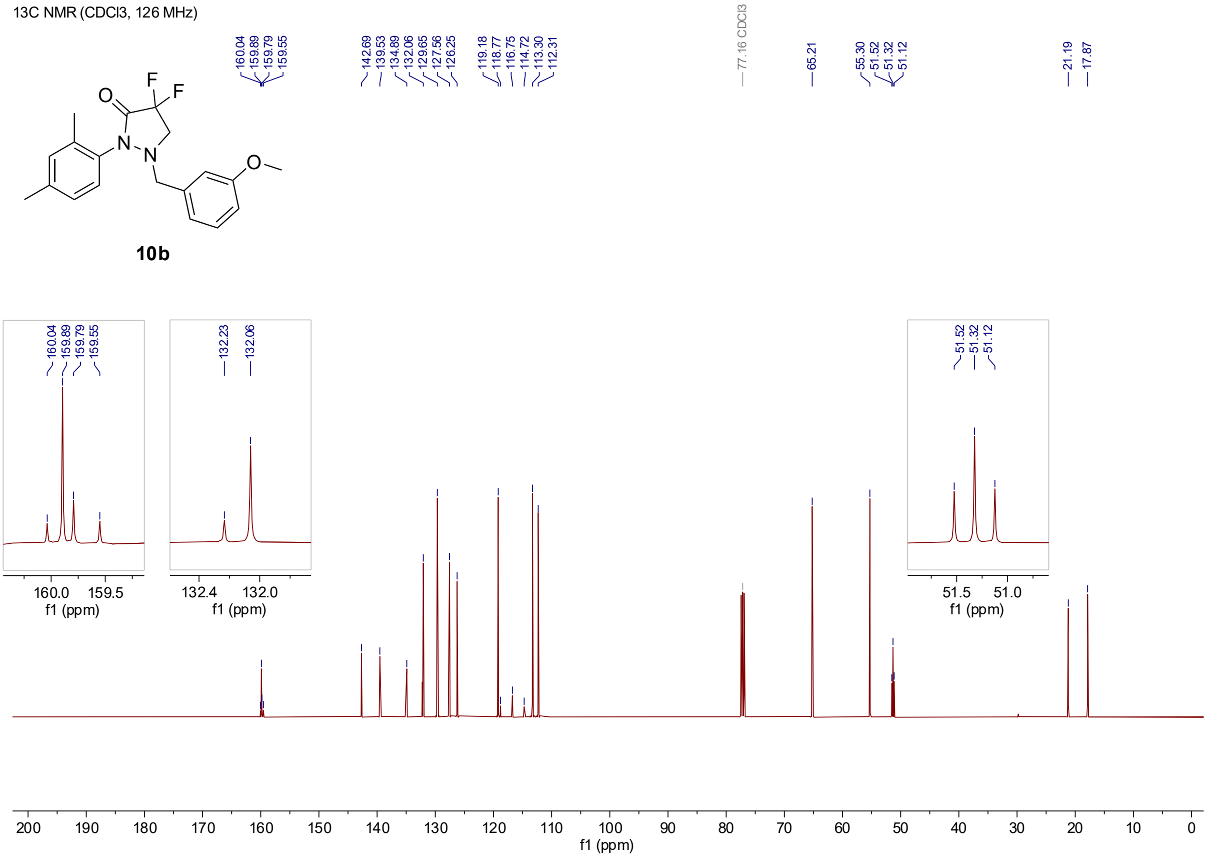

^{13}C NMR spectrum of (**10b**)

HRMS

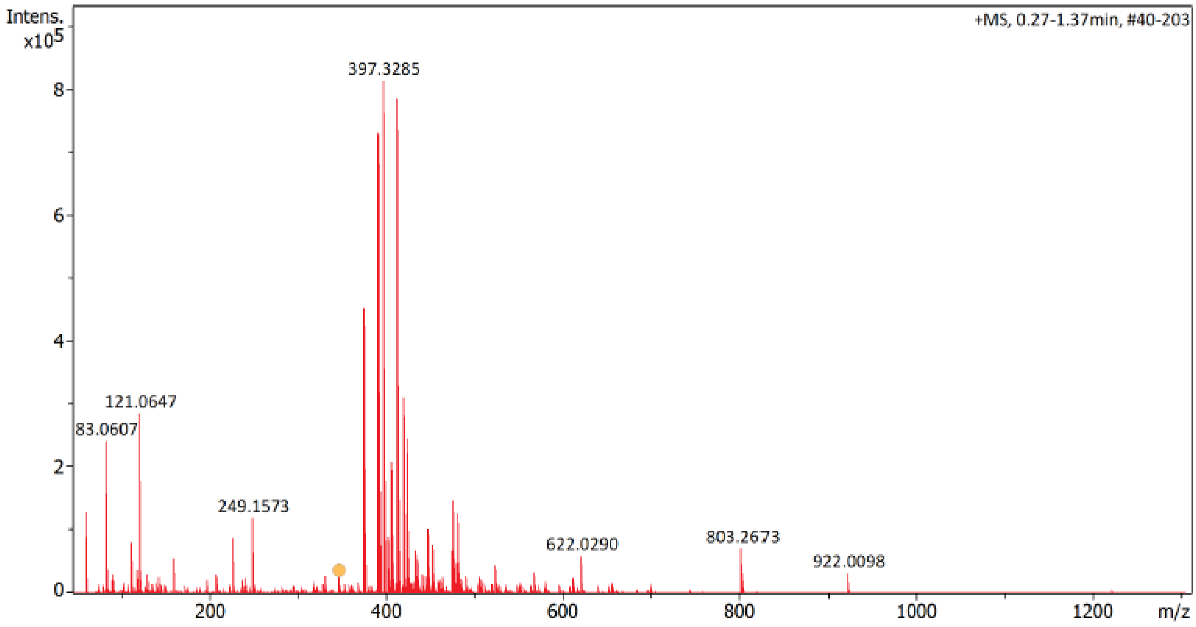

HRMS(ESI+) spectrum of (**10b**)

LC-MS

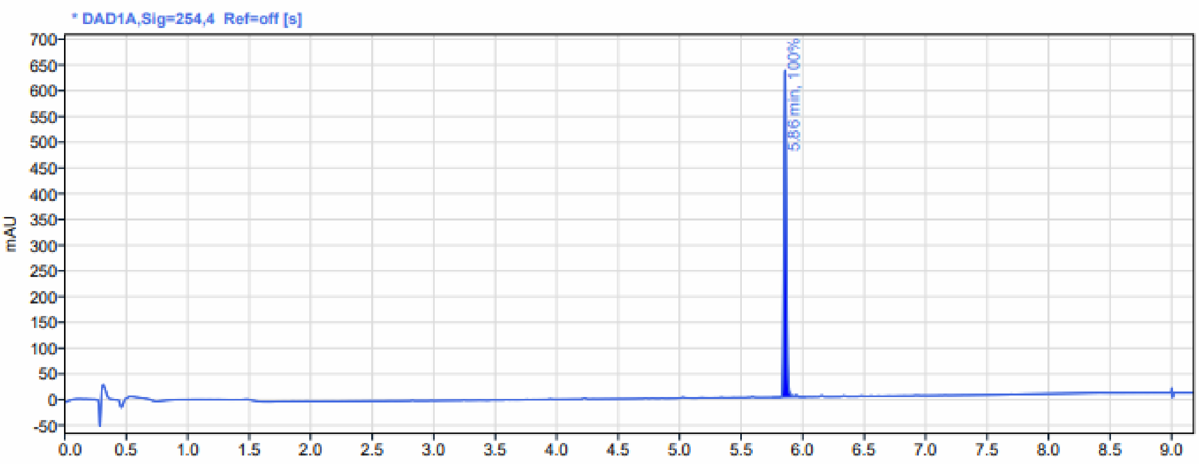

LC-MS HPLC chromatogram at 254.4 nm of (**10b**)

Characterization of (11b)

1H-NMR

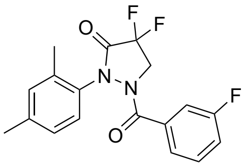

11b

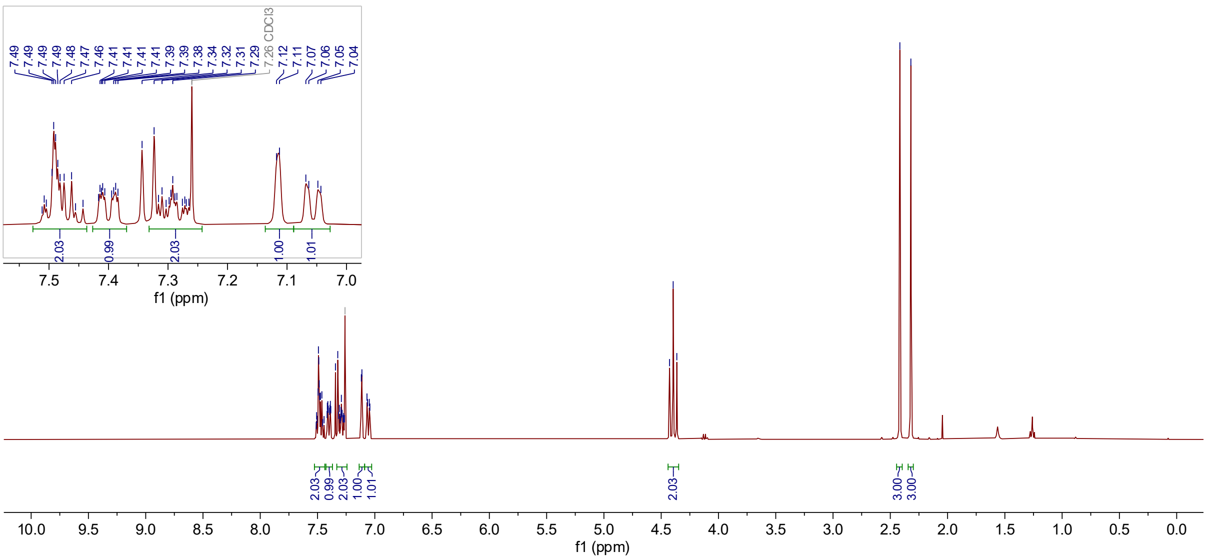

^{1}H NMR spectrum of (**11b**)

19F-NMR

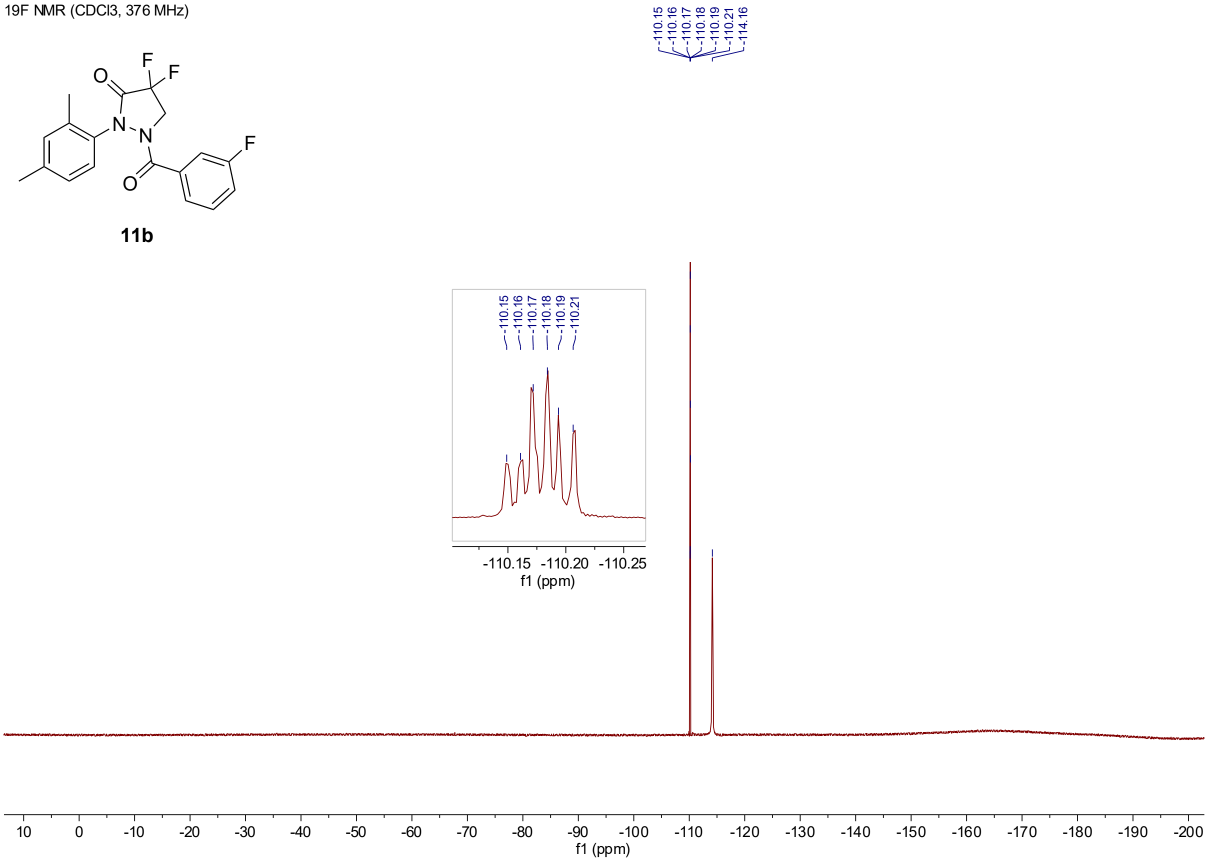

^{19}F NMR spectrum of (**11b**)

13C-NMR

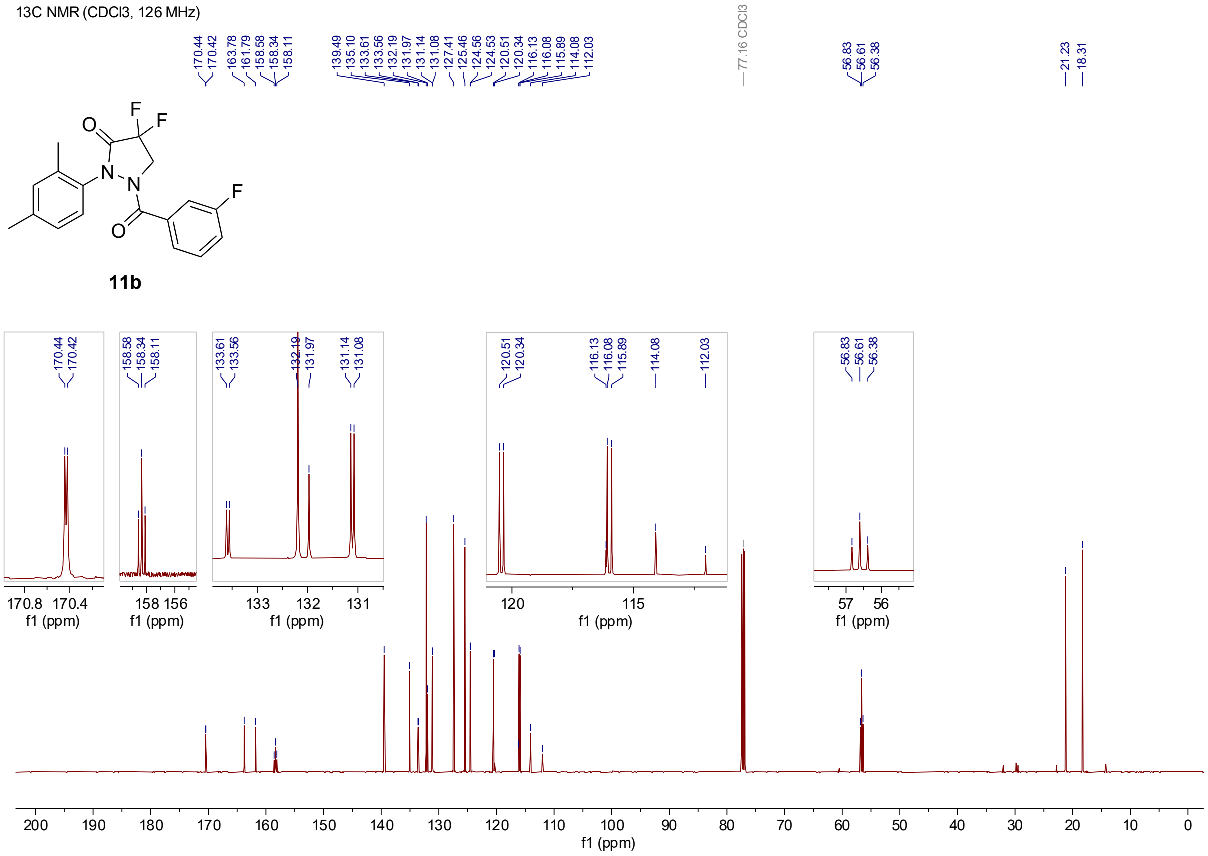

^{13}C NMR spectrum of (**11b**)

HRMS

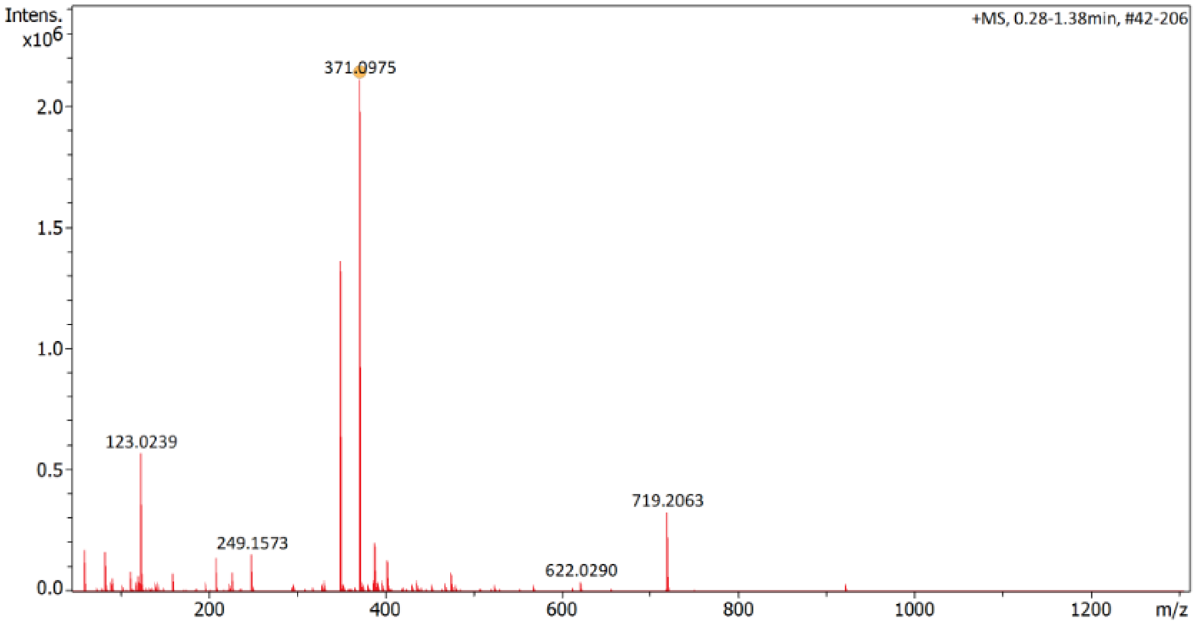

HRMS(ESI+) spectrum of (**11b**)

LC-MS

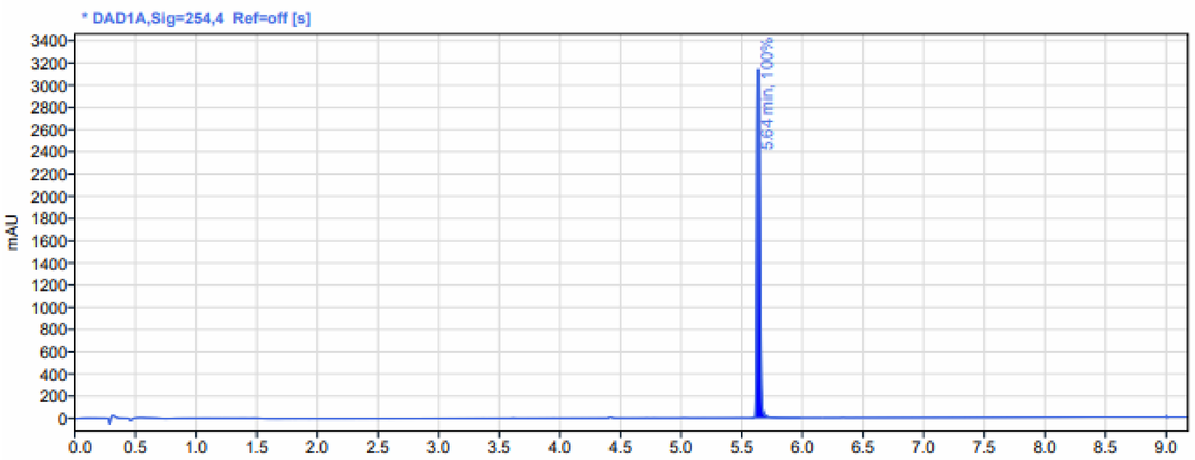

LC-MS HPLC chromatogram at 254.4 nm of (**11b**)

Characterization of (12b)

1H-NMR

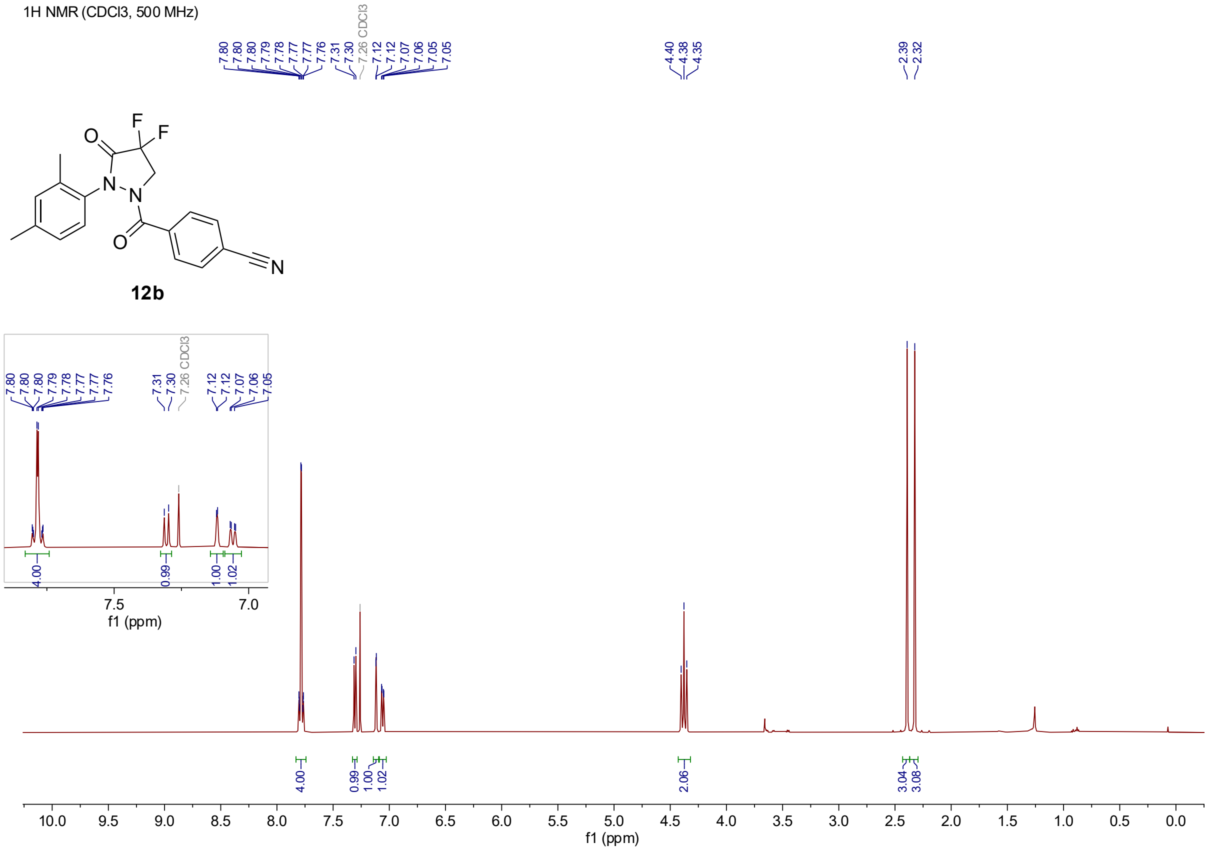

^{1}H NMR spectrum of (**12b**)

19F-NMR

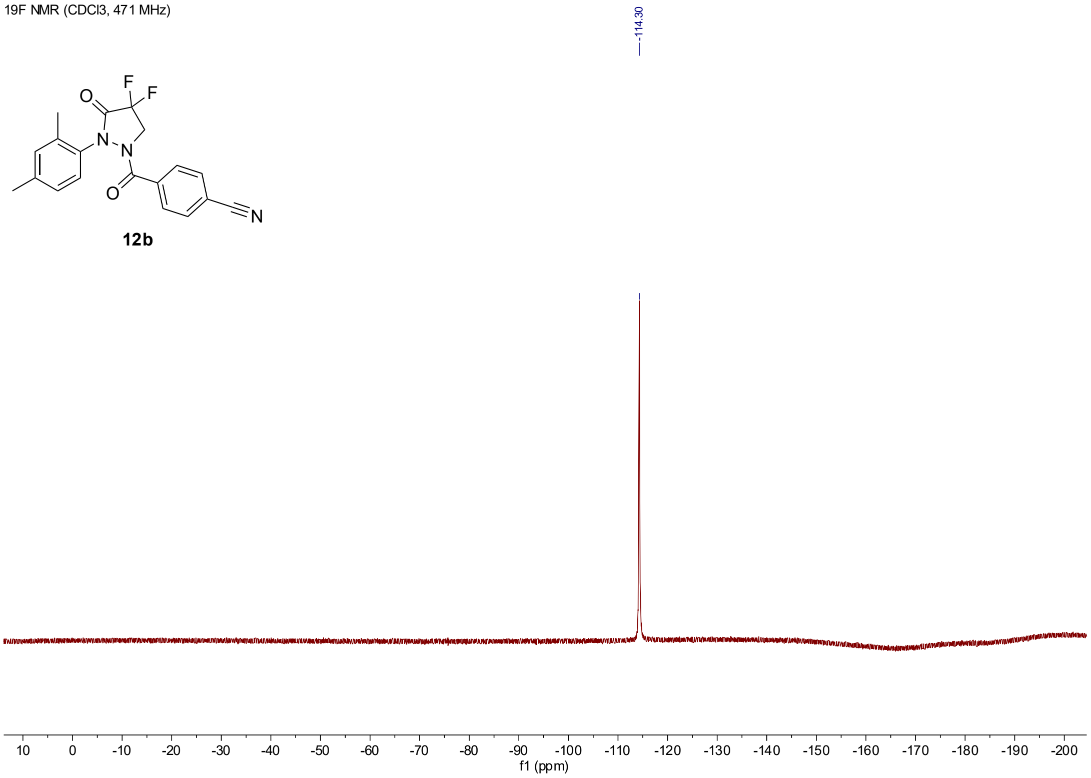

^{19}F NMR spectrum of (**12b**)

13C-NMR

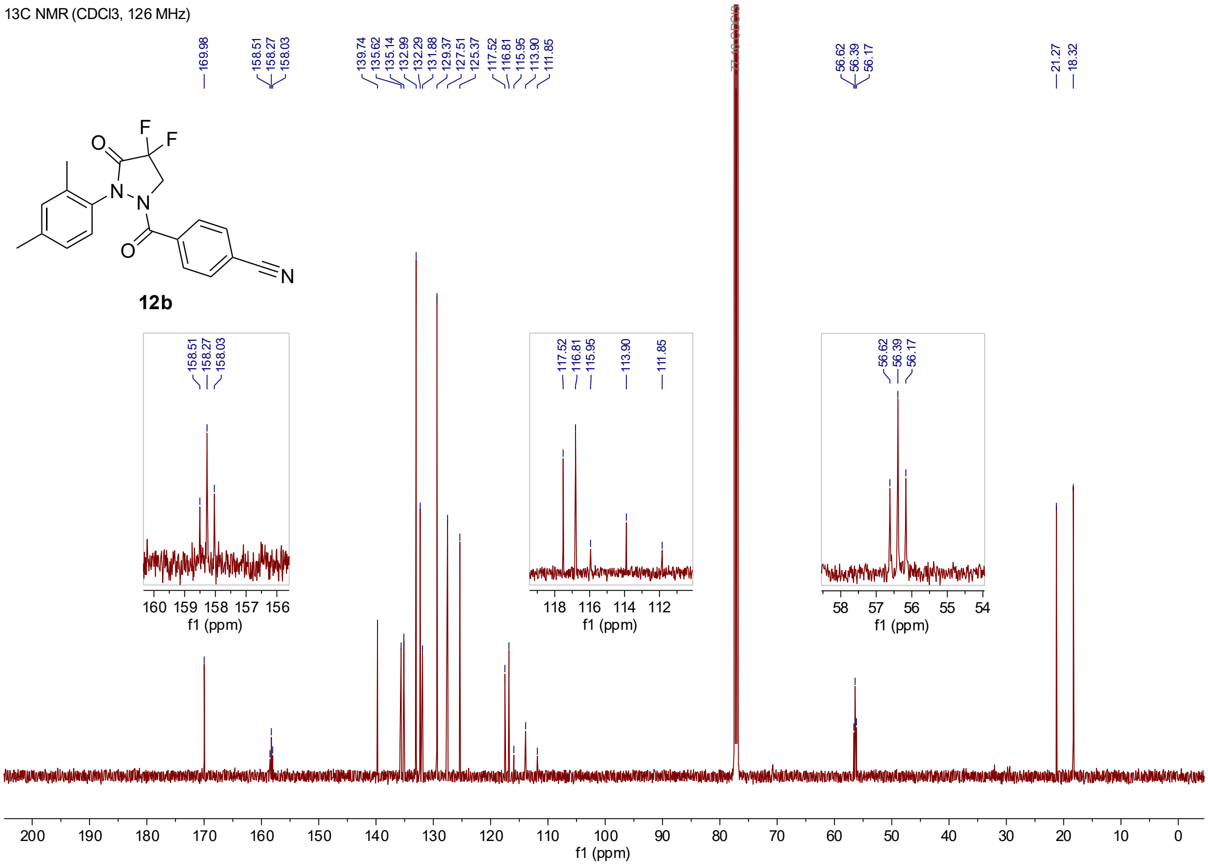

^{13}C NMR spectrum of (**12b**)

HRMS

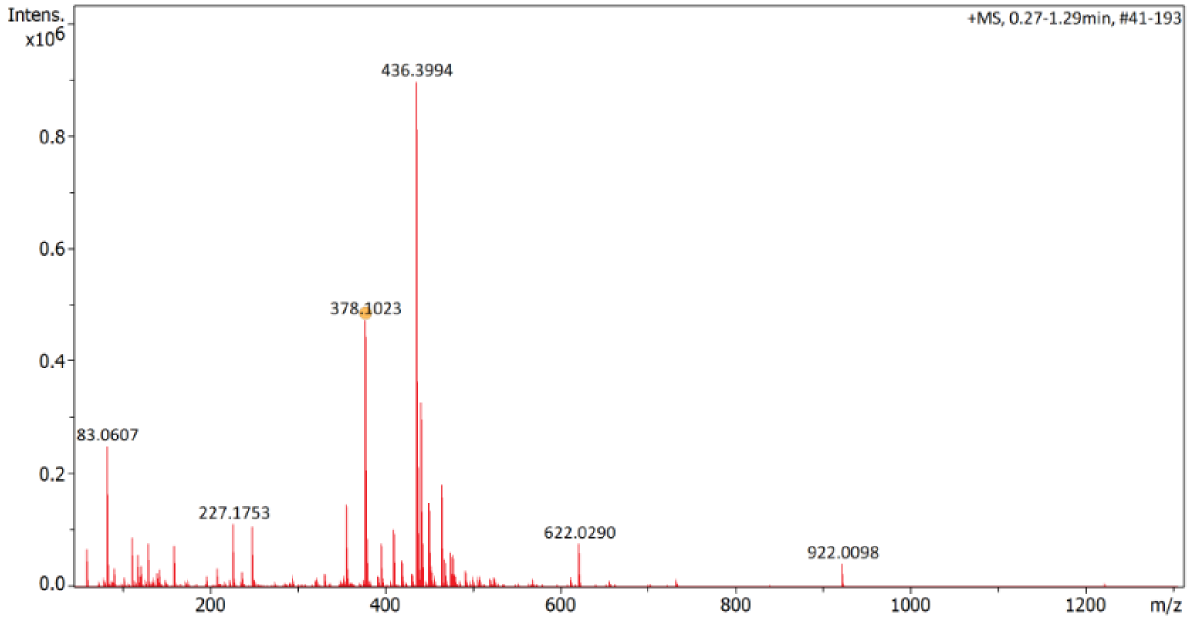

HRMS(ESI+) spectrum of (**12b**)

LC-MS

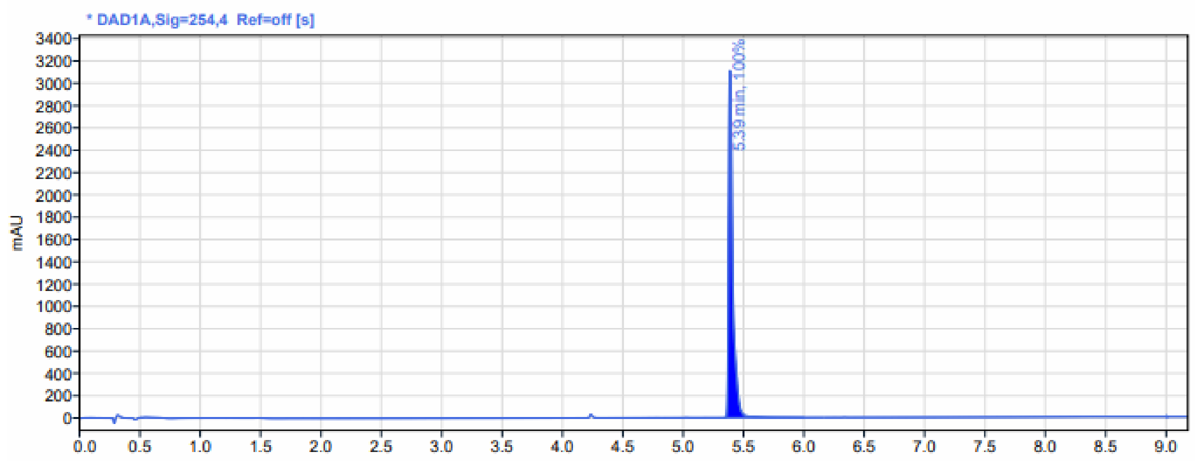

LC-MS HPLC chromatogram at 254.4 nm of (**12b**)

4,4-Difluoro-pyrazol-3-ones

Characterization of (4a)

1H-NMR

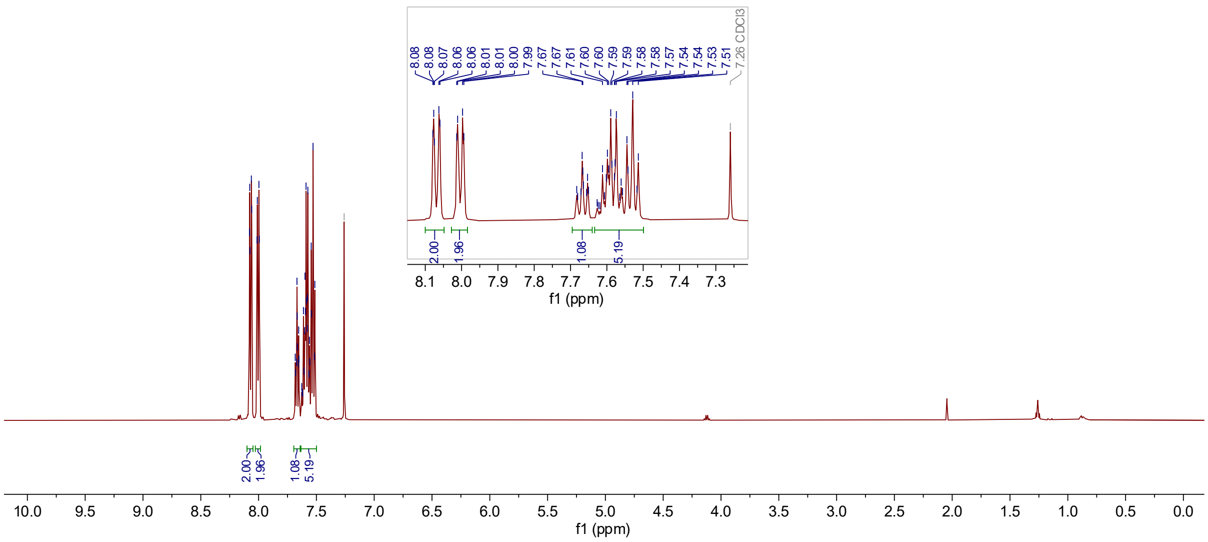

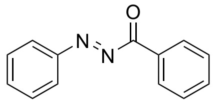

4a

^{1}H NMR spectrum of (**4a**)

HRMS

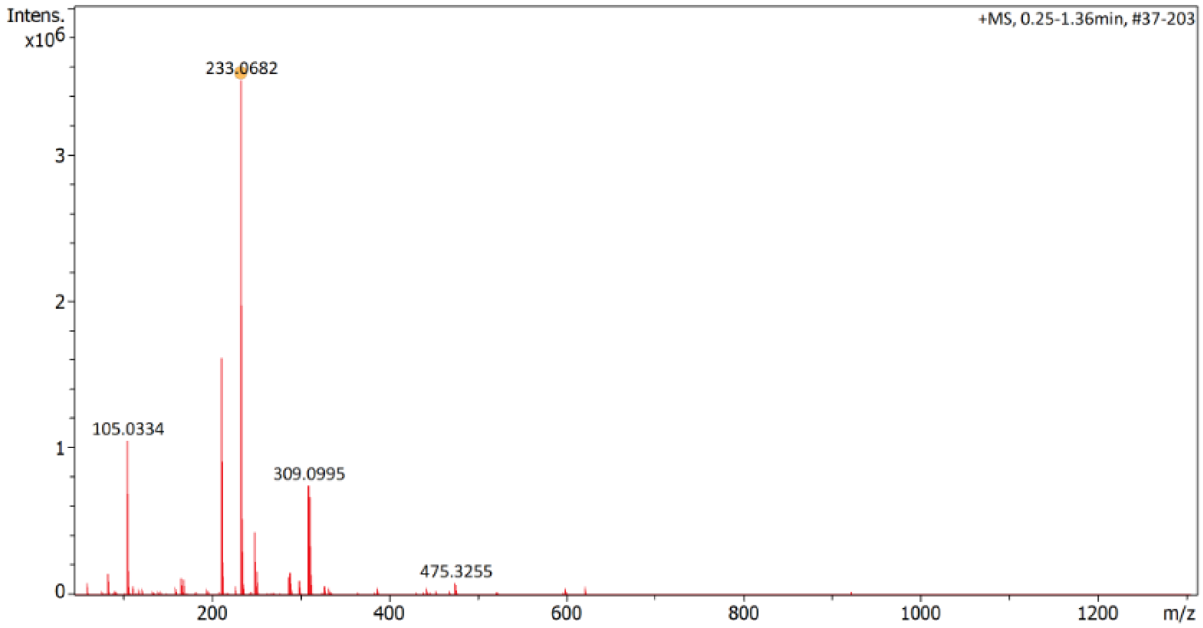

HRMS(ESI+) spectrum of (**4a**)

Characterization of (4b)

1H-NMR

1H NMR (CDCl3, 500 MHz)

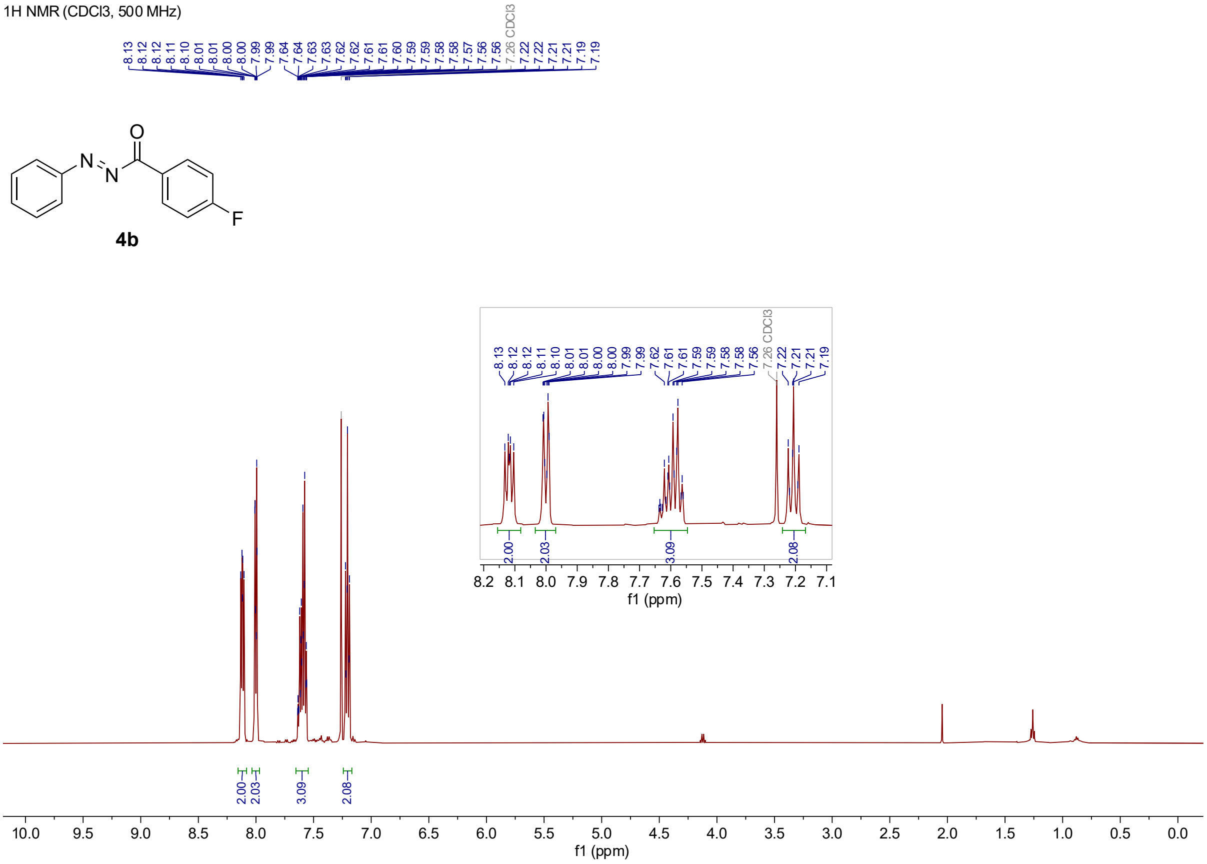

^{1}H NMR spectrum of (**4b**)

19F-NMR

19F NMR (CDCl3, 471 MHz)

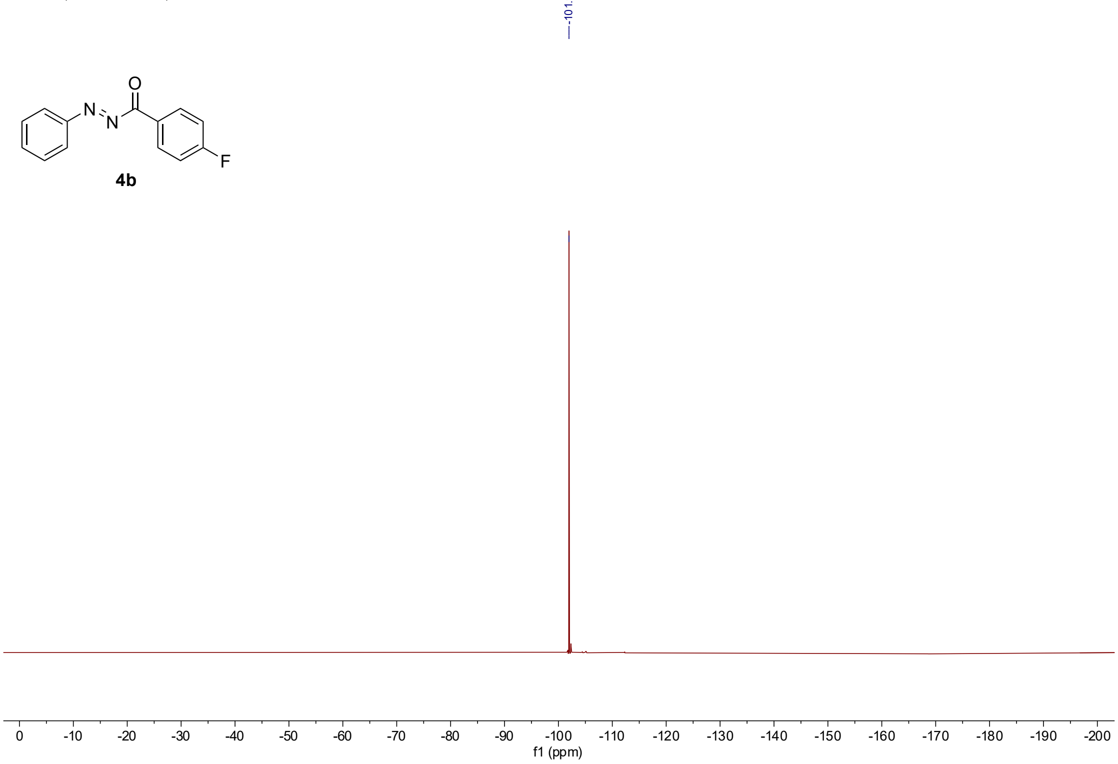

^{19}F NMR spectrum of (**4b**)

HRMS

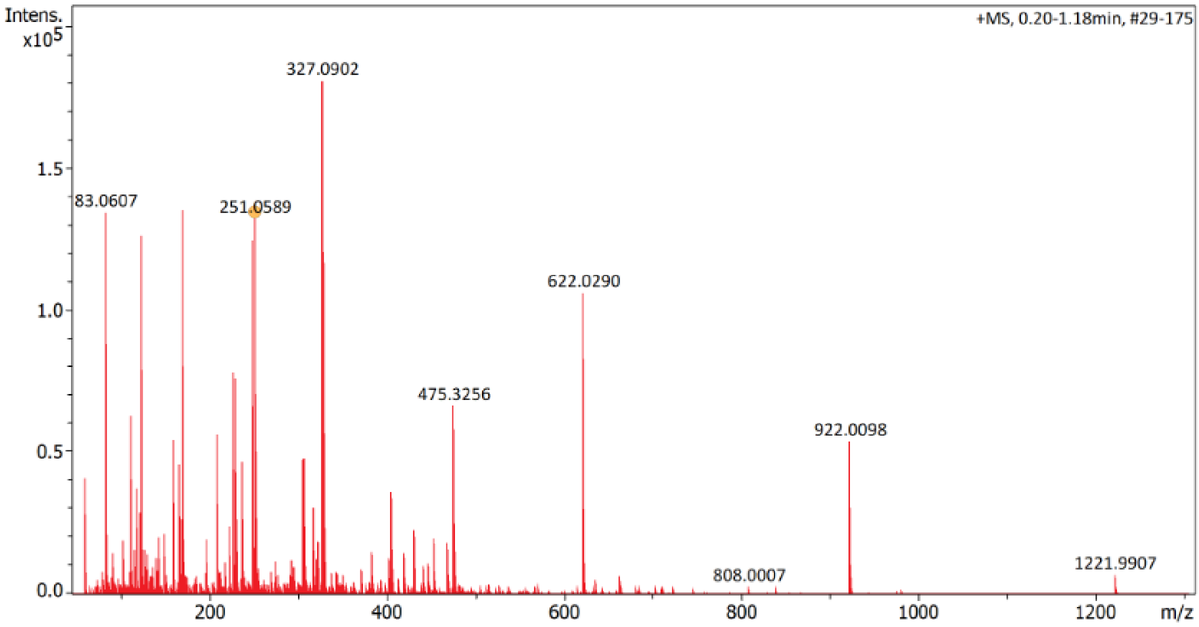

HRMS(ESI+) spectrum of (**4b**)

Characterization of (4c)

1H-NMR

1H NMR (CDCl3, 500 MHz)

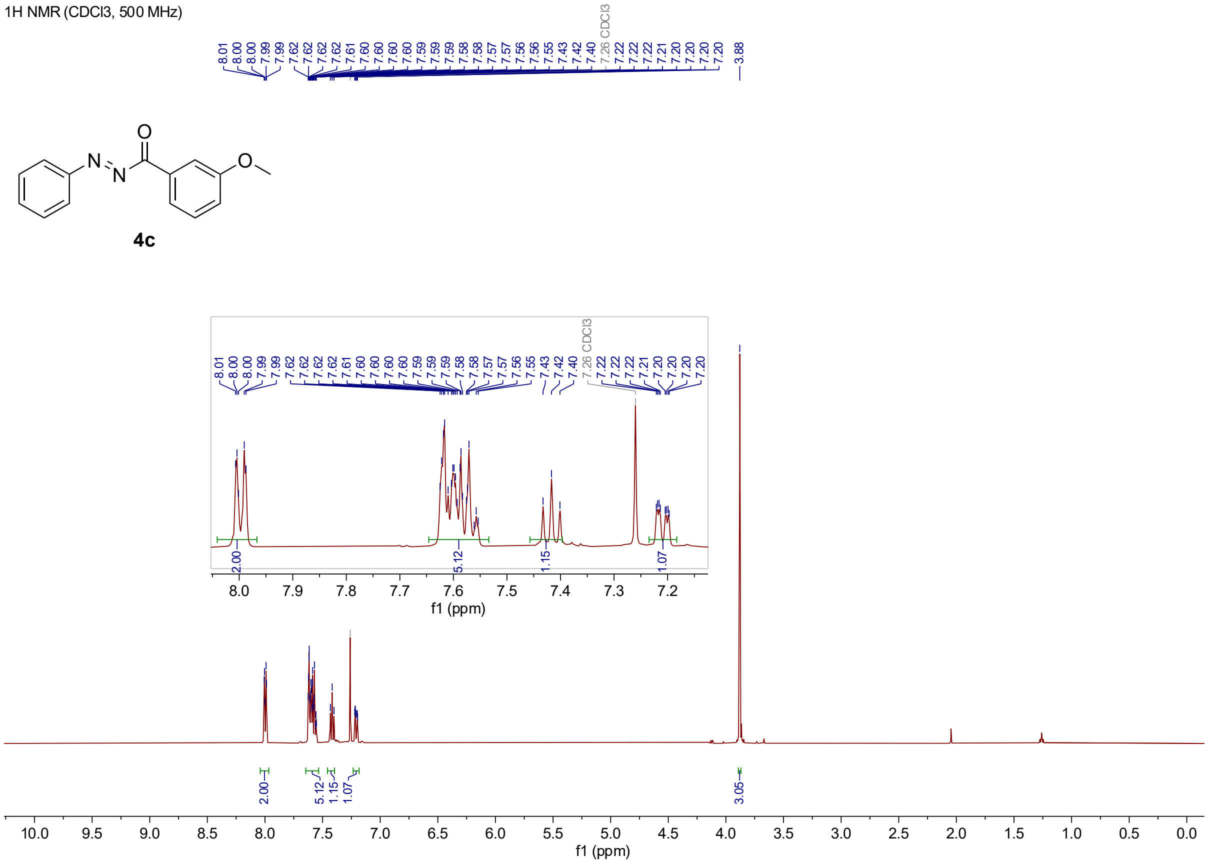

^{1}H NMR spectrum of (**4c**)

HRMS

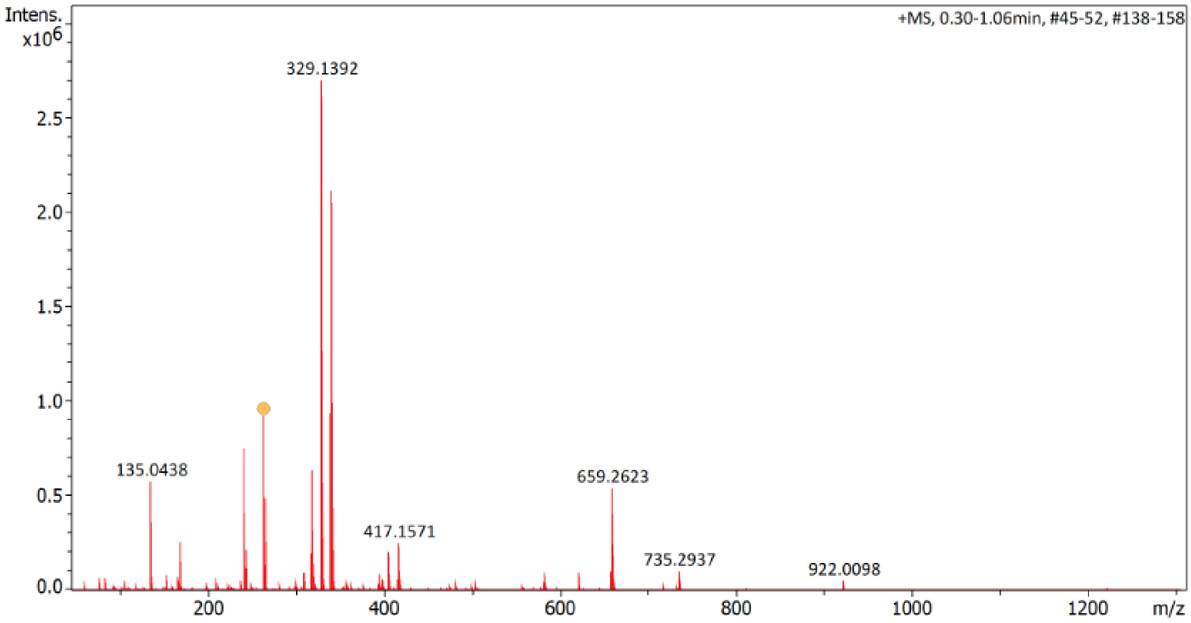

HRMS(ESI+) spectrum of (**4c**)

Characterization of (4d)

1H-NMR

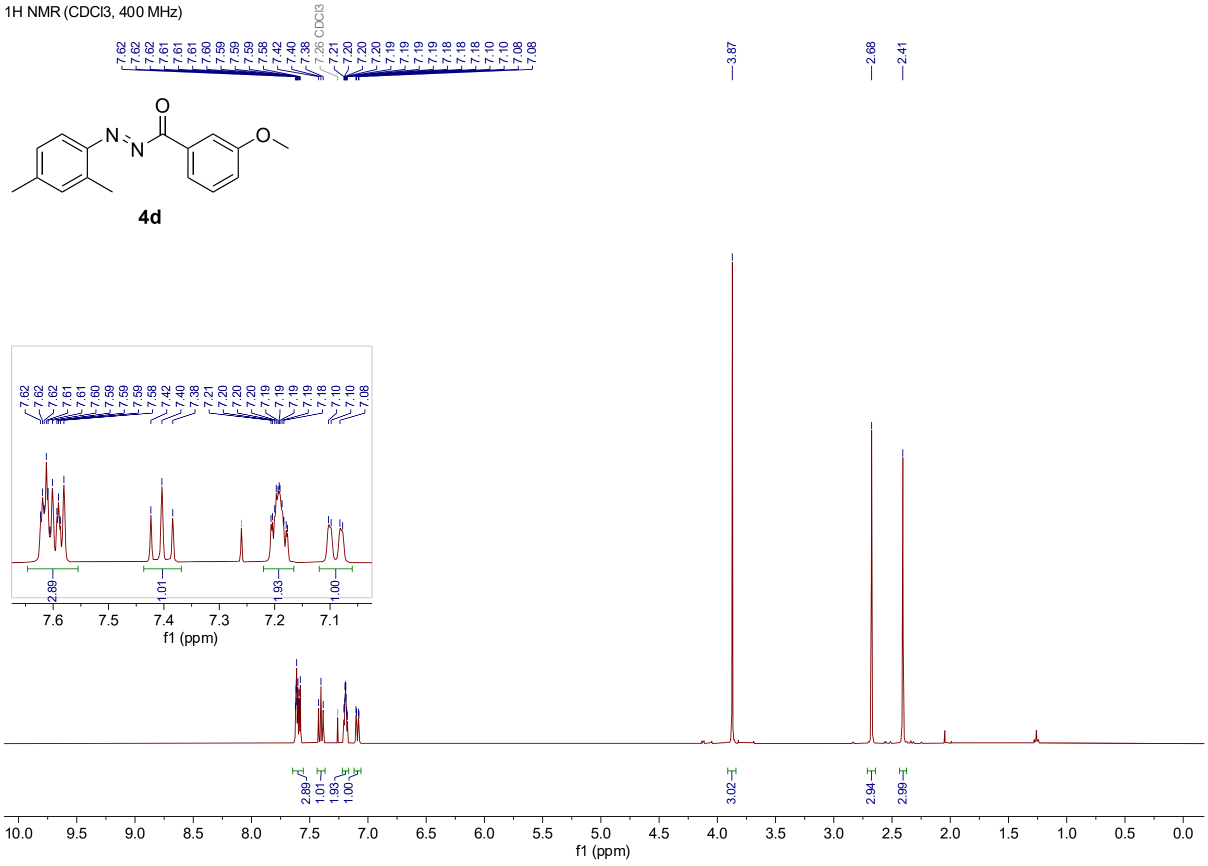

^{1}H NMR spectrum of (**4d**)

13C-NMR

13C NMR (CDCl3, 101 MHz)

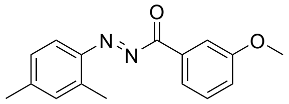

4d

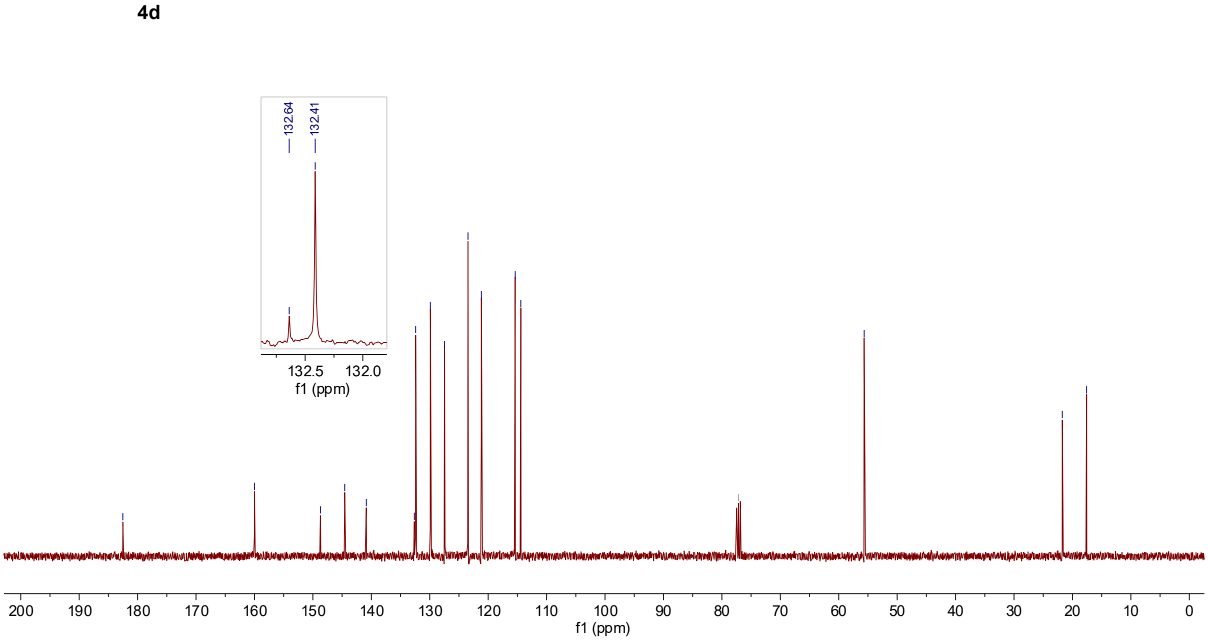

^{13}C NMR spectrum of (**4d**)

HRMS

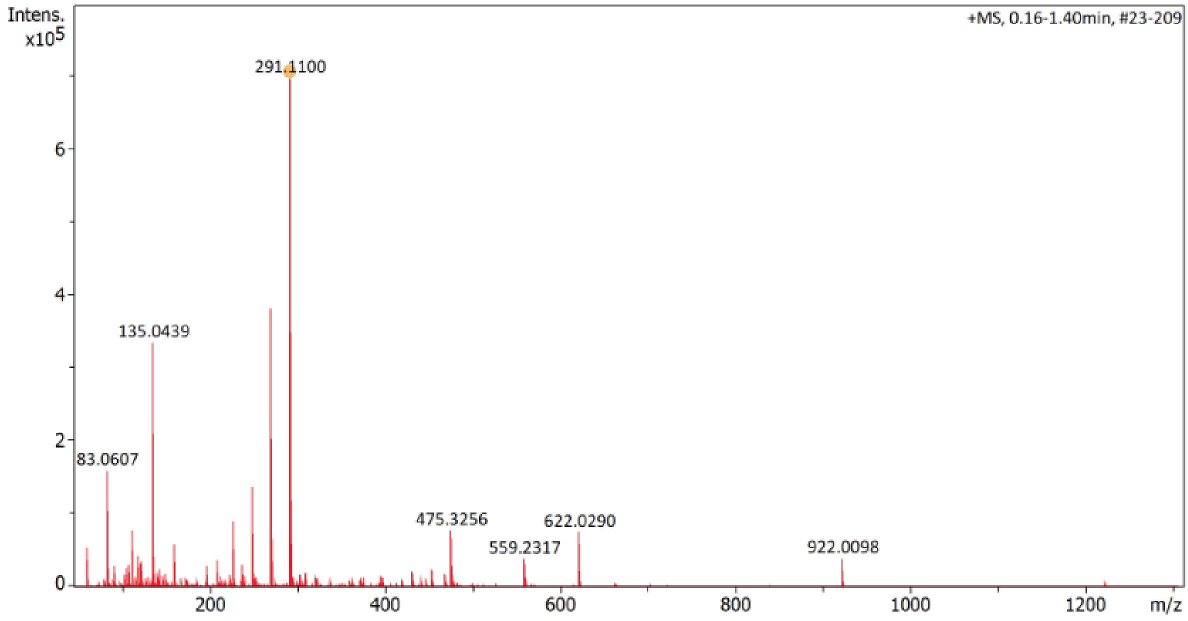

HRMS(ESI+) spectrum of (**4d**)

Characterization of (4e)

1H-NMR

1H NMR (CDCl3, 400 MHz)

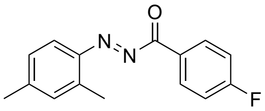

4e

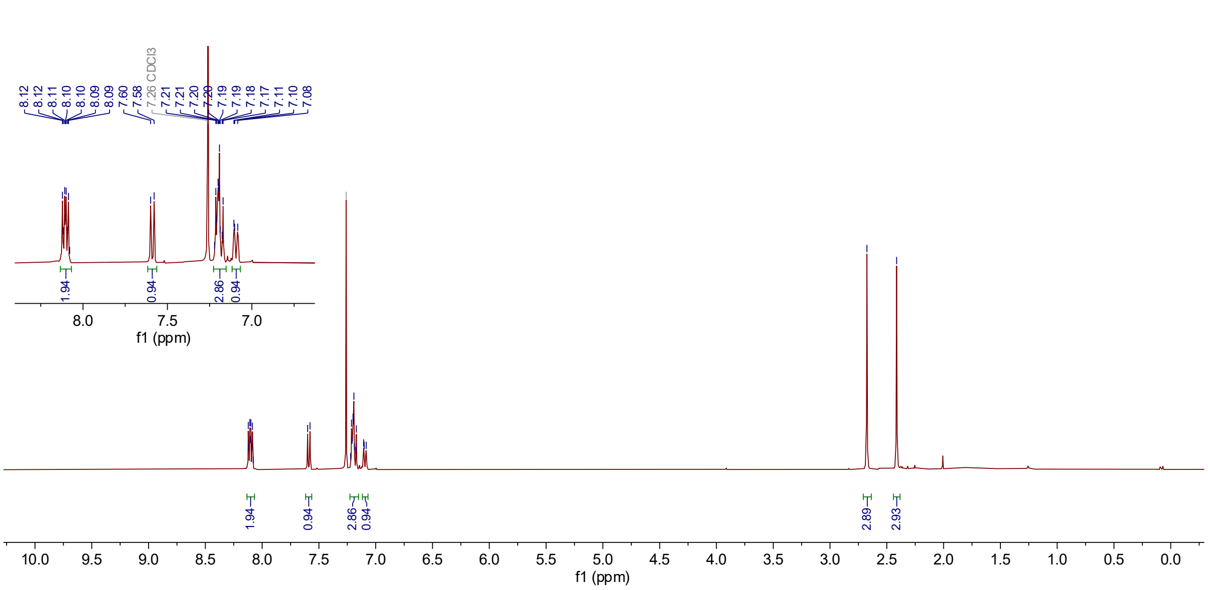

^{1}H NMR spectrum of (**4e**)

19F-NMR

19F NMR (CDCl3, 376 MHz)

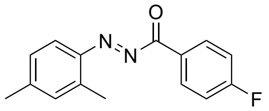

4e

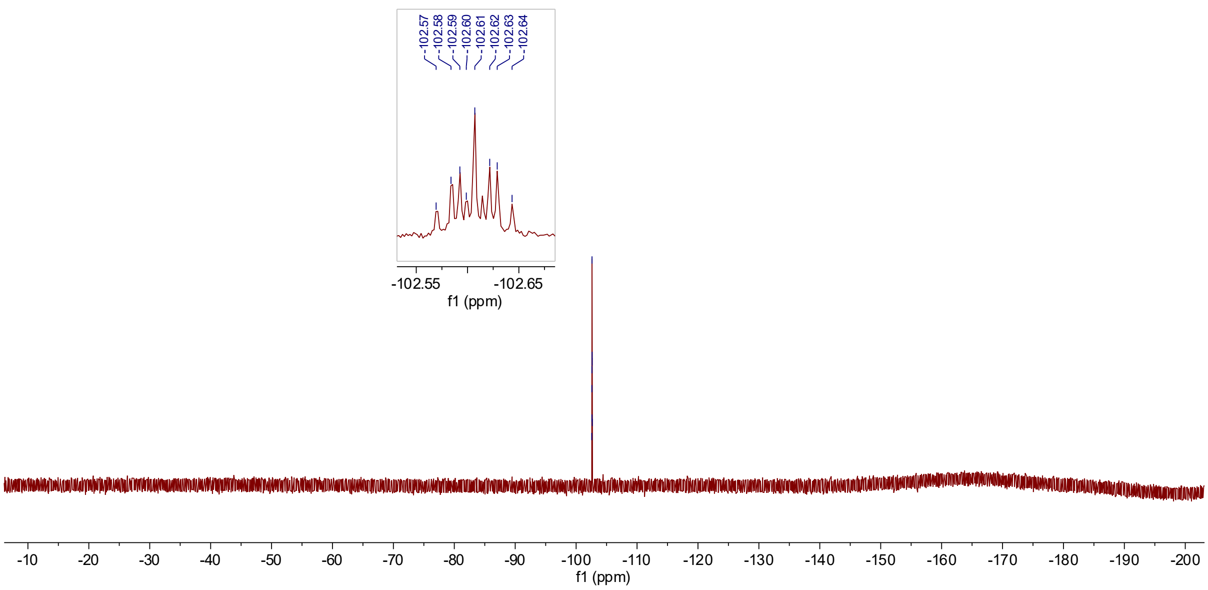

^{19}F NMR spectrum of (**4e**)

13C-NMR

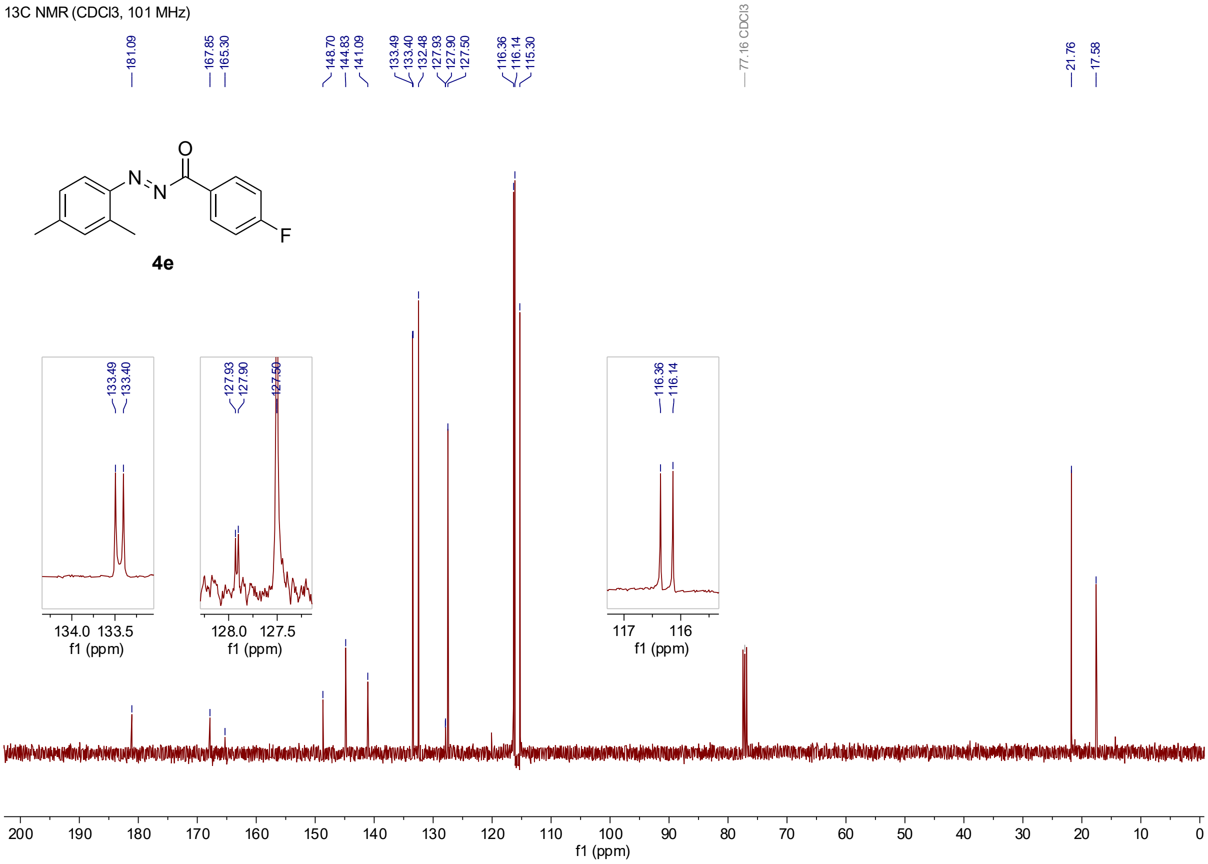

^{13}C NMR spectrum of (**4e**)

HRMS

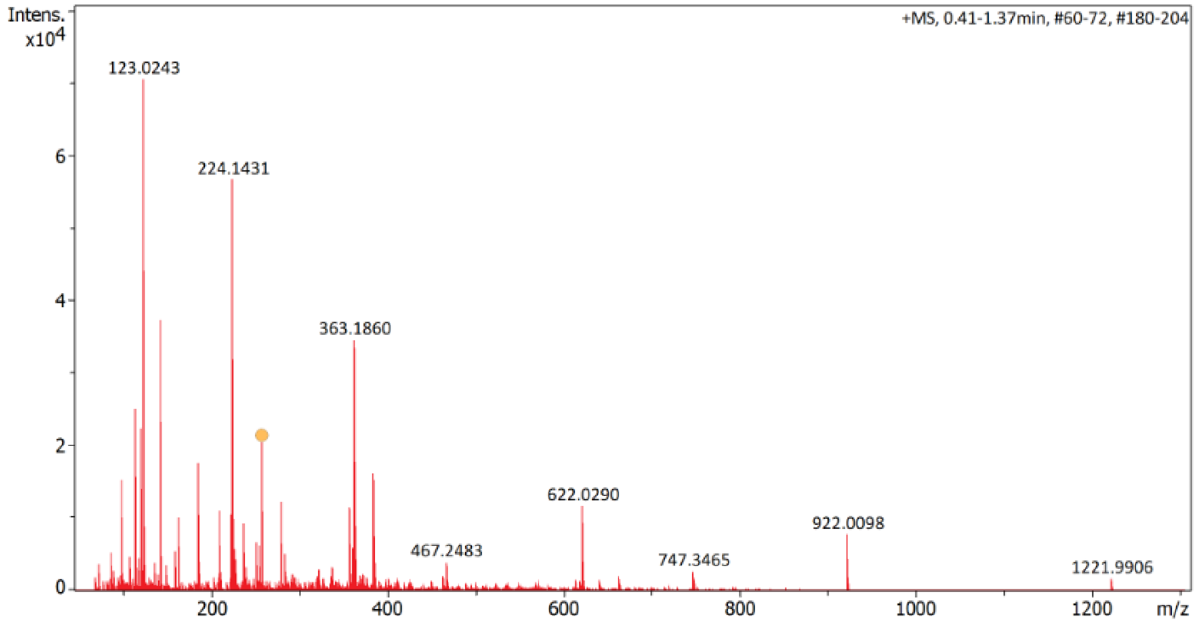

HRMS(ESI+) spectrum of (**4e**)

Characterization of (5a)

1H-NMR

1H NMR (CDCl3, 500 MHz)

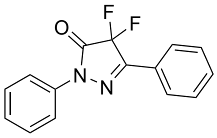

5a

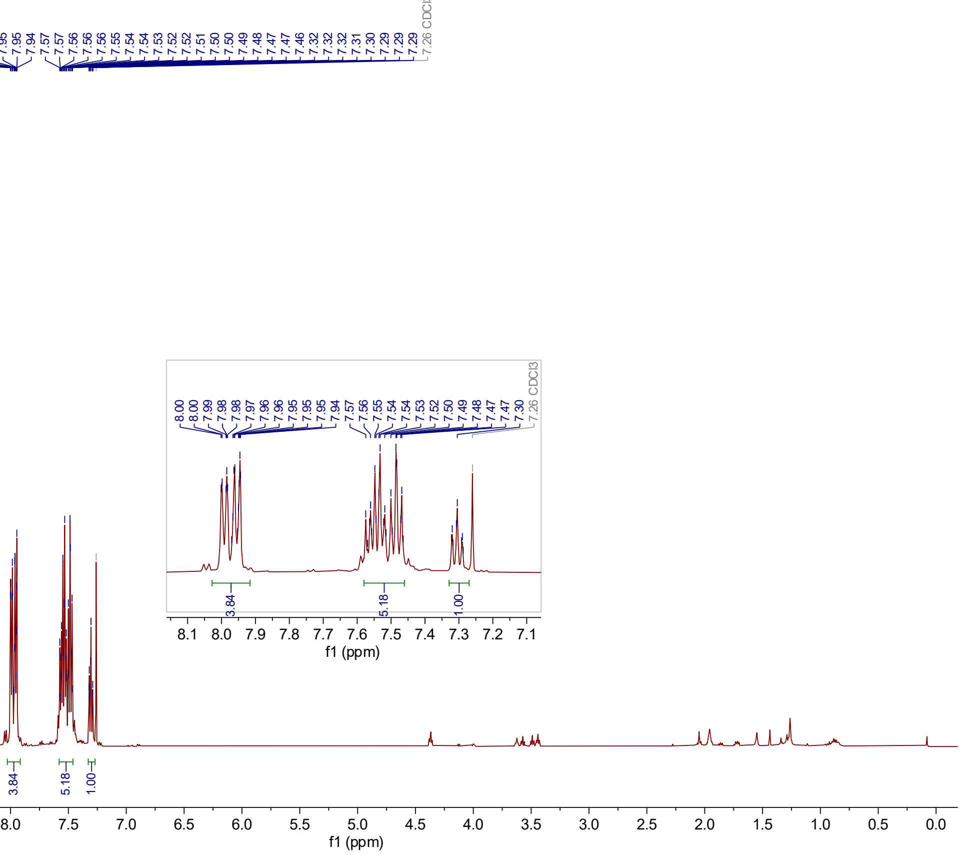

^{1}H NMR spectrum of (**5a**)

19F NMR

19F NMR (CDCl3, 471 MHz)

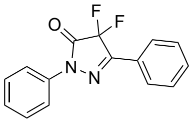

5a

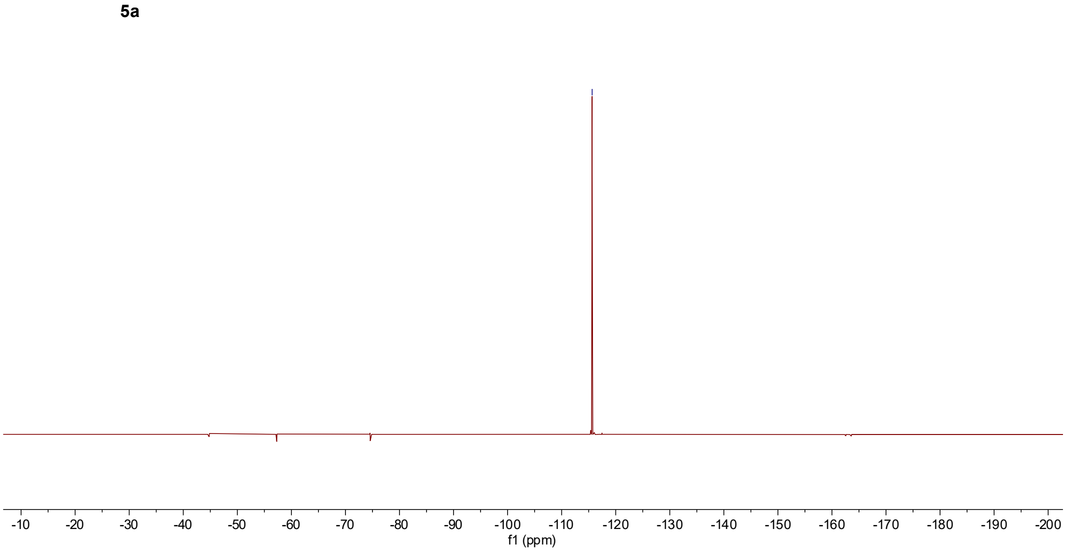

^{19}F NMR spectrum of (**5a**)

LC-MS

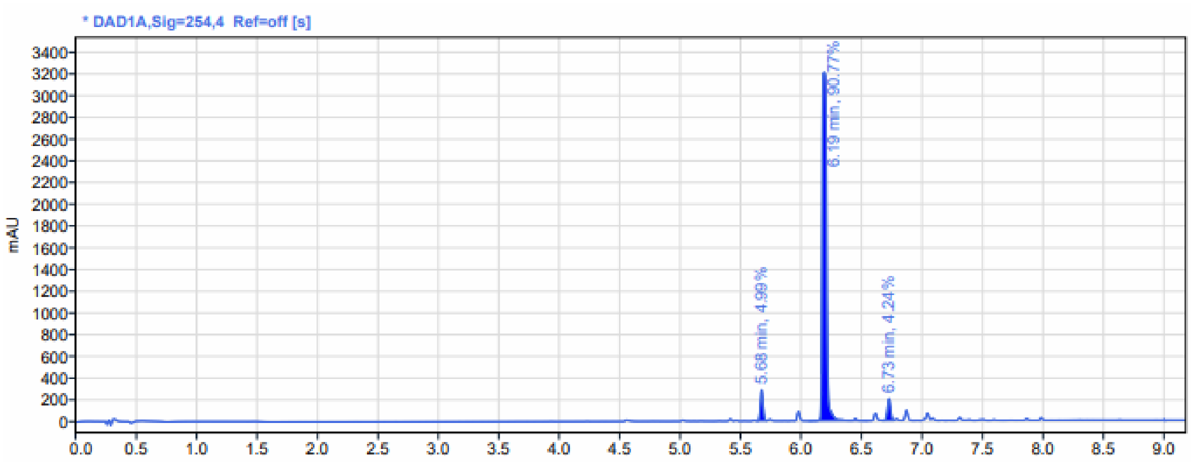

LC-MS HPLC chromatogram at 254.4 nm of (**5a**)

Characterization of (5b)

1H-NMR

1H NMR (CDCl3, 500 MHz)

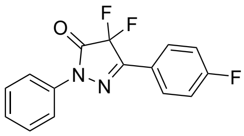

5b

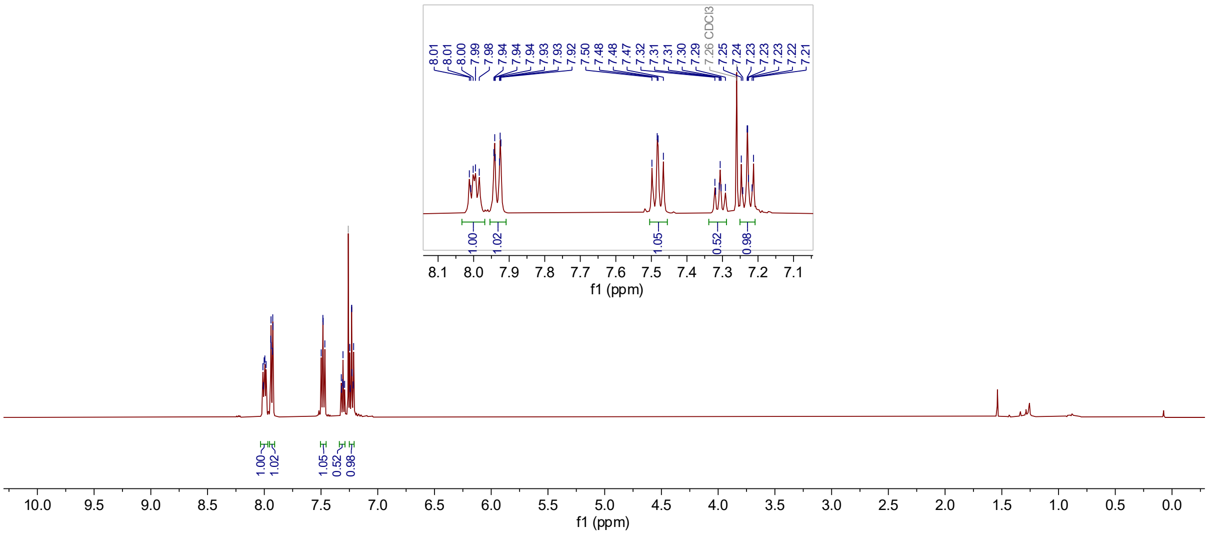

^{1}H NMR spectrum of (**5b**)

19F-NMR

19F NMR (CDCl3, 471 MHz)

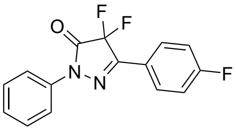

5b

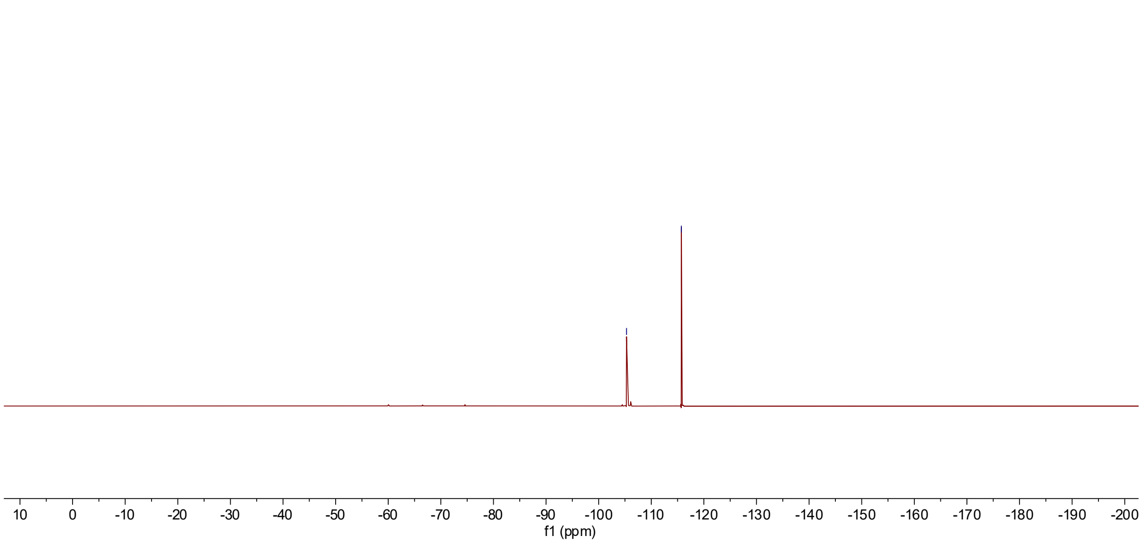

^{19}F NMR spectrum of (**5b**)

13C-NMR

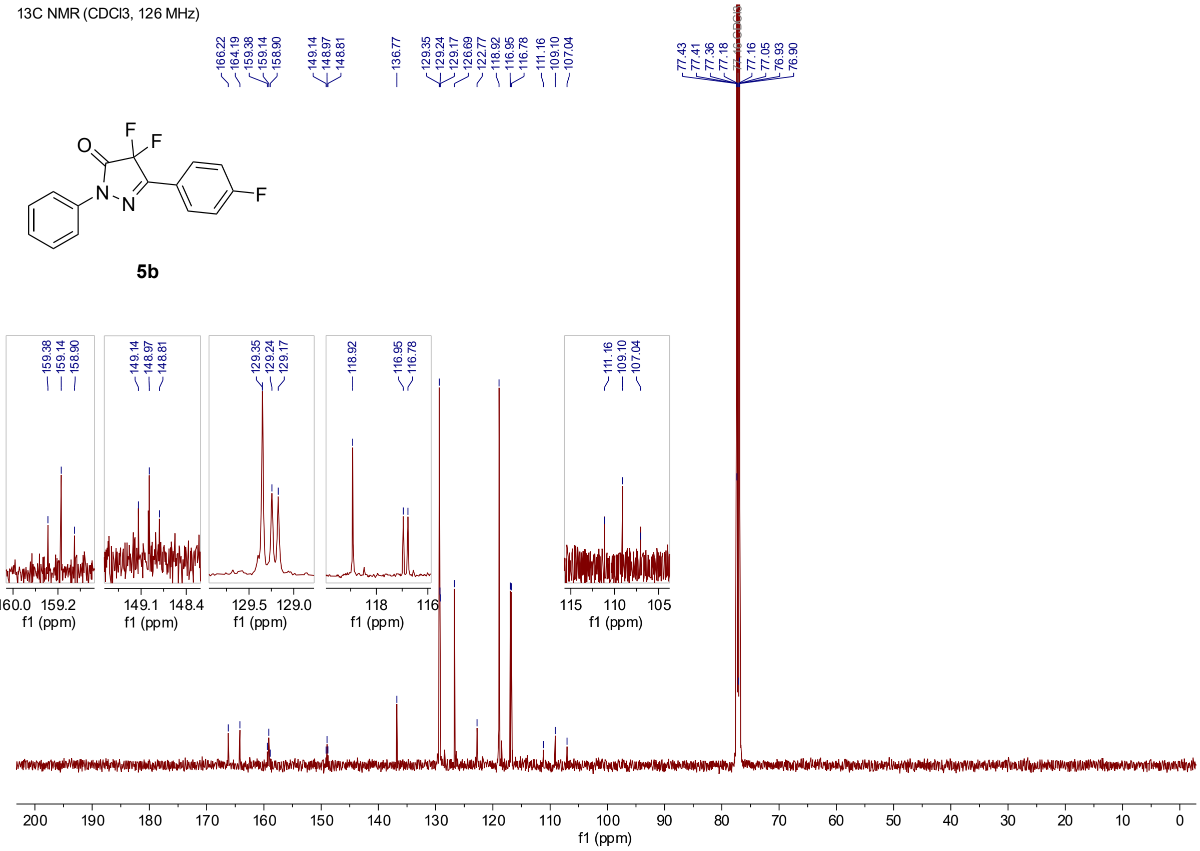

^{13}C NMR spectrum of (**5b**)

HRMS

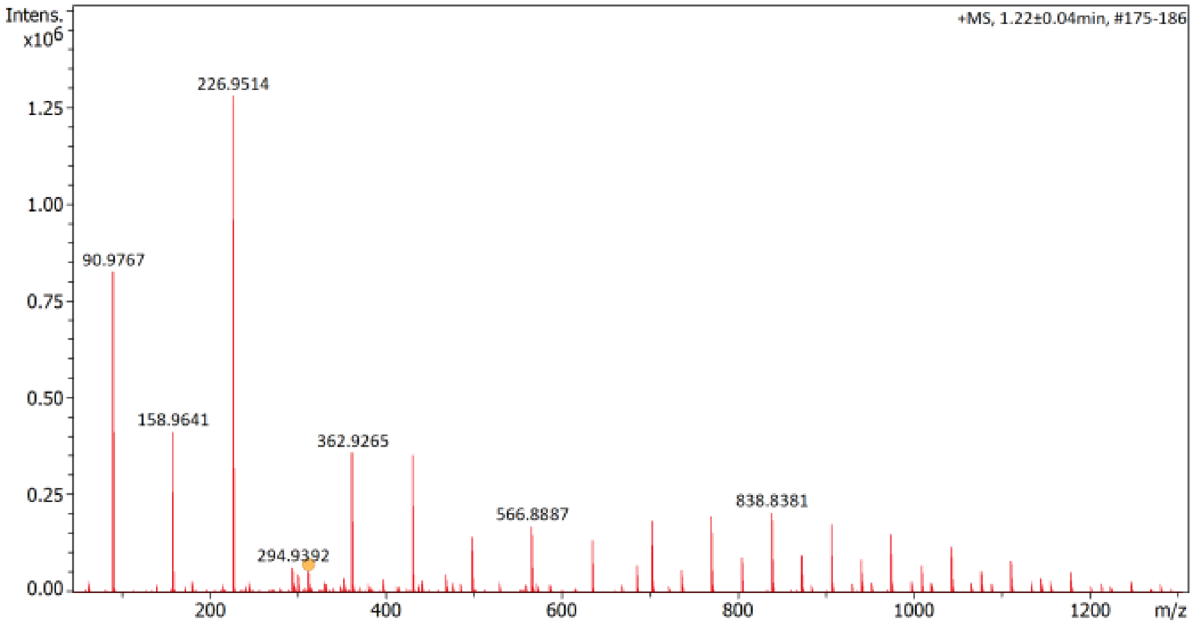

HRMS(ESI+) spectrum of (**5b**)

LC-MS

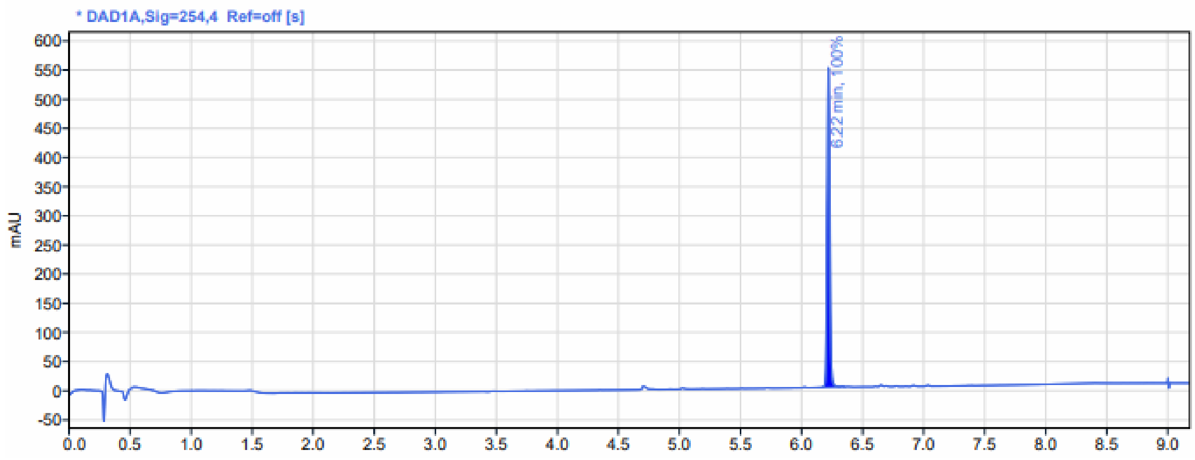

LC-MS HPLC chromatogram at 254.4 nm of (**5b**)

Characterization of (5c)

1H-NMR

1H NMR (CDCl3, 500 MHz)

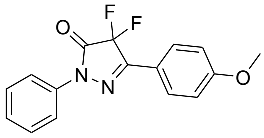

5c

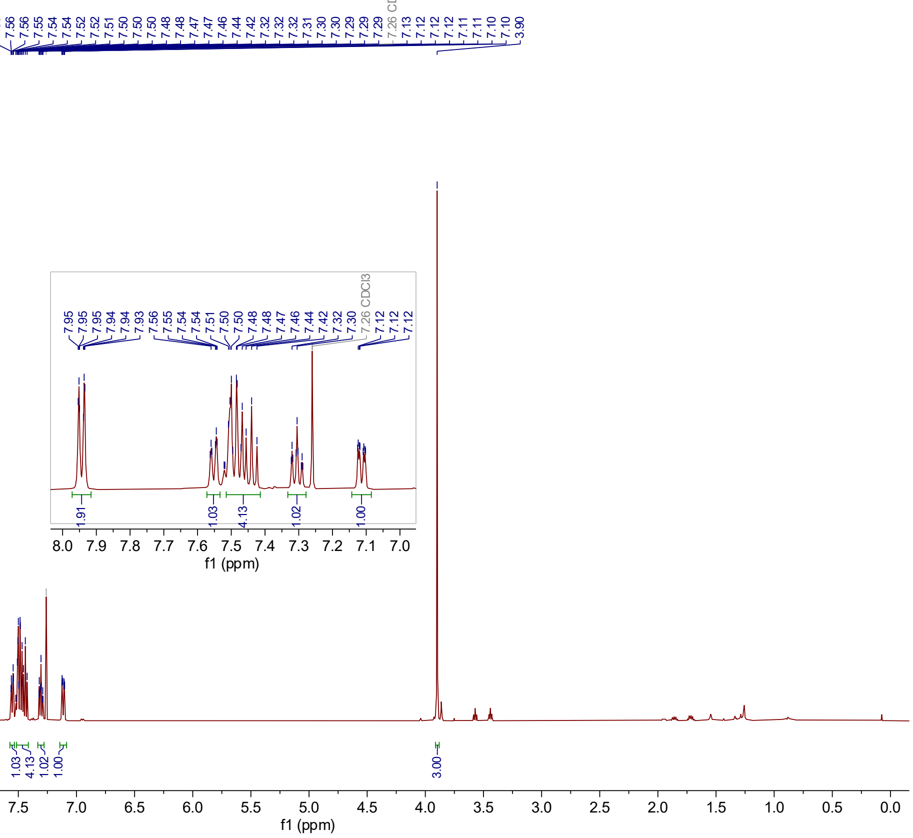

^{1}H NMR spectrum of (**5c**)

19F NMR

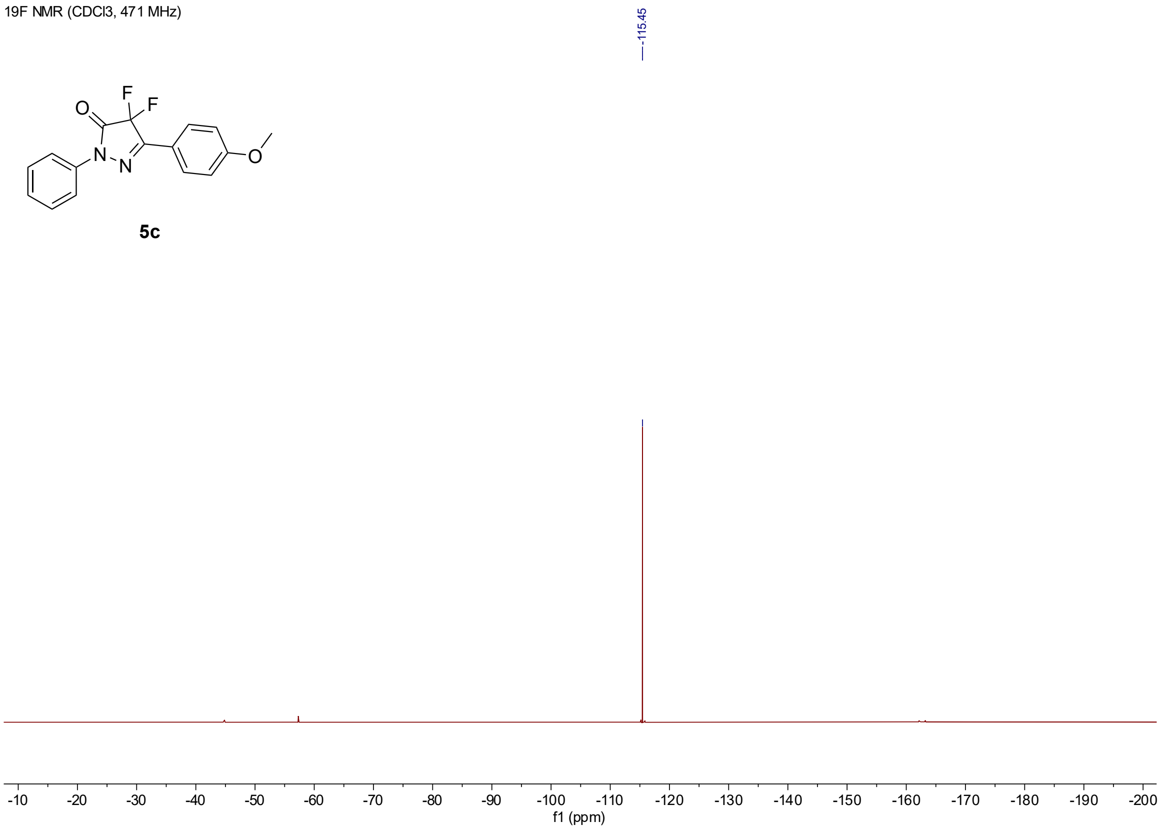

^{19}F NMR spectrum of (**5c**)

13C-NMR

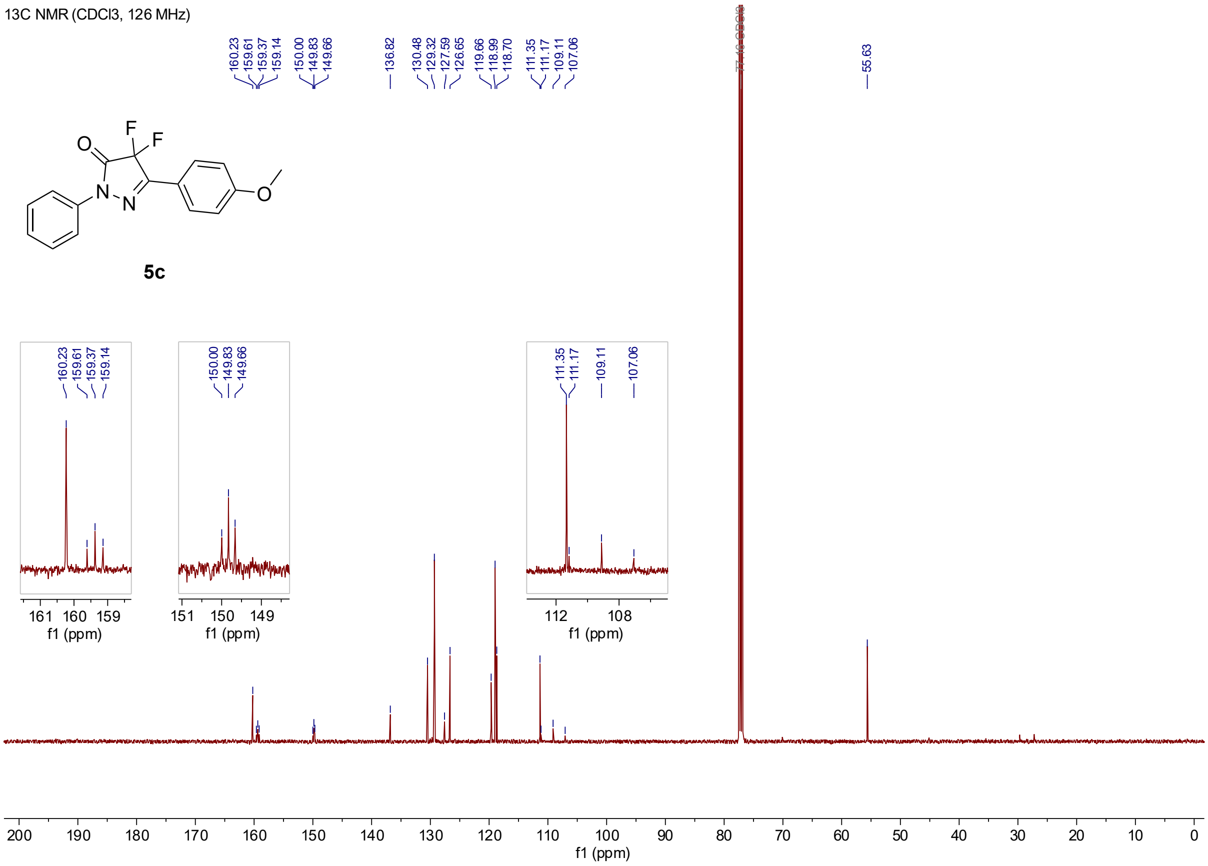

^{13}C NMR spectrum of (**5c**)

HRMS

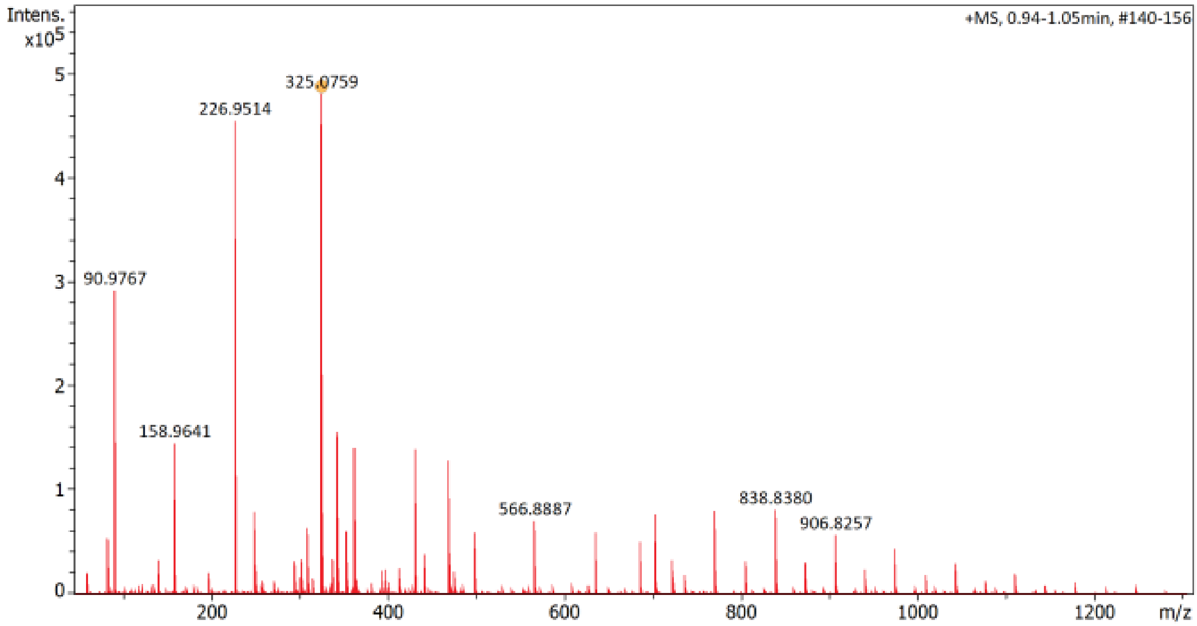

HRMS(ESI+) spectrum of (**5c**)

LC-MS

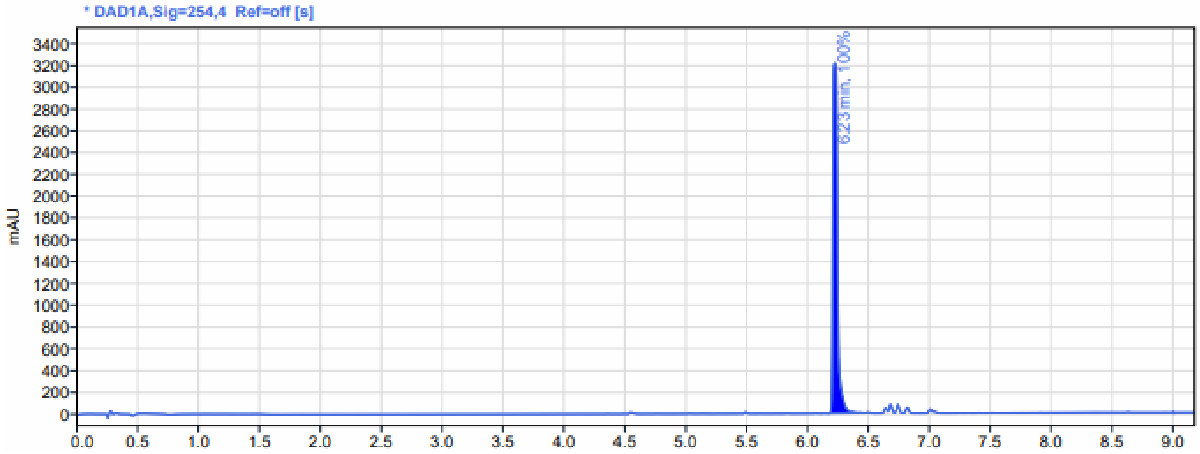

LC-MS HPLC chromatogram at 254.4 nm of (**5c**)

Characterization of (5d)

1H-NMR

1H NMR (CDCl3, 400 MHz)

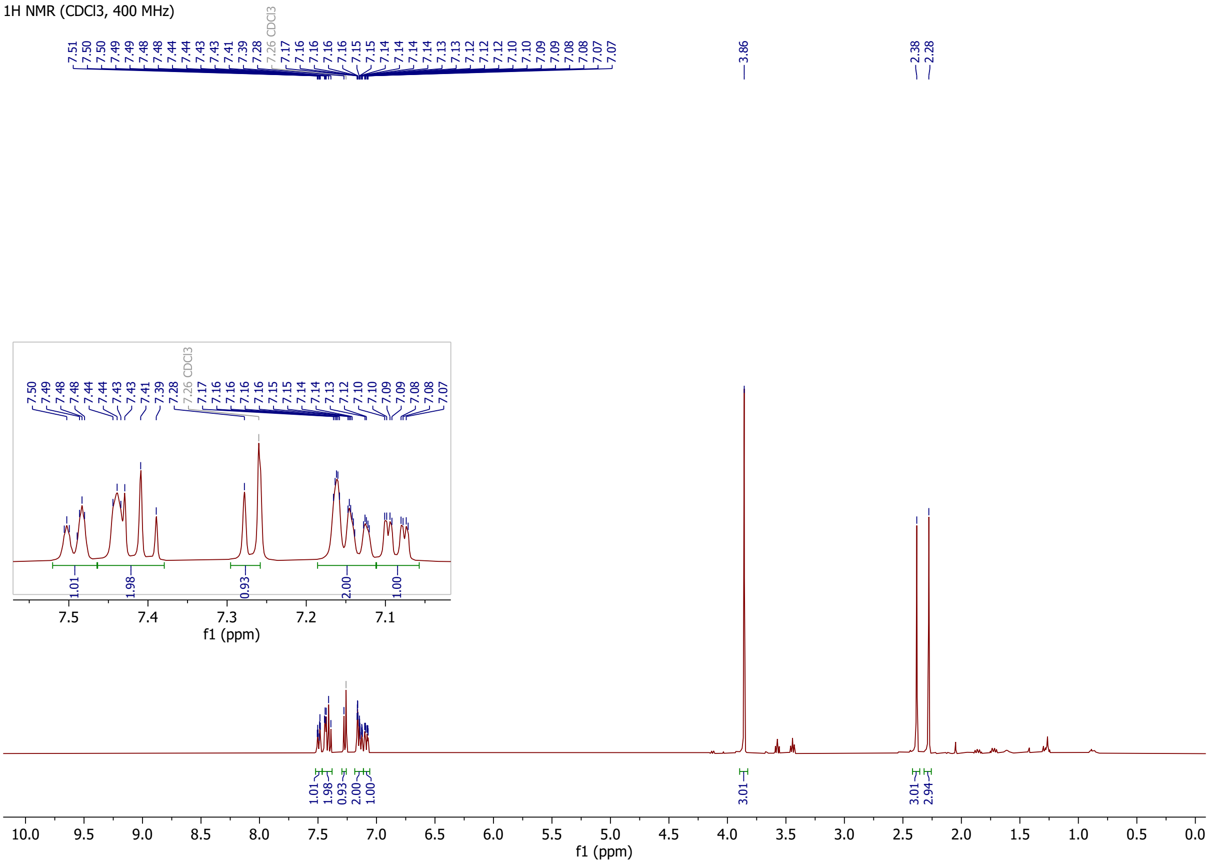

^{1}H NMR spectrum of (**5d**)

19F-NMR

19F NMR (CDCl3, 376 MHz)

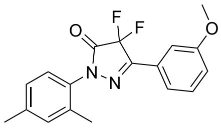

5d

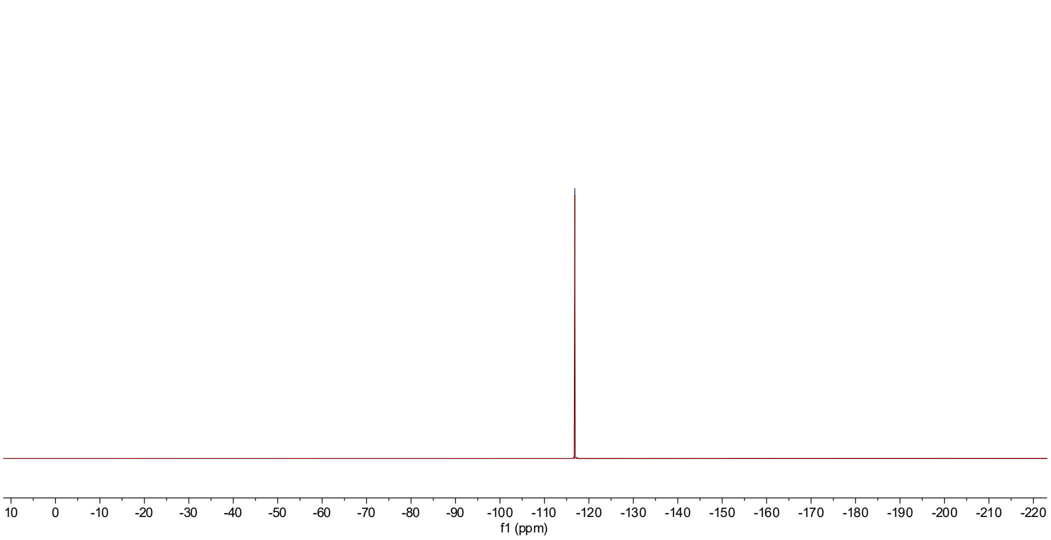

^{19}F NMR spectrum of (**5d**)

13C-NMR

13C NMR (CDCl3, 101 MHz)

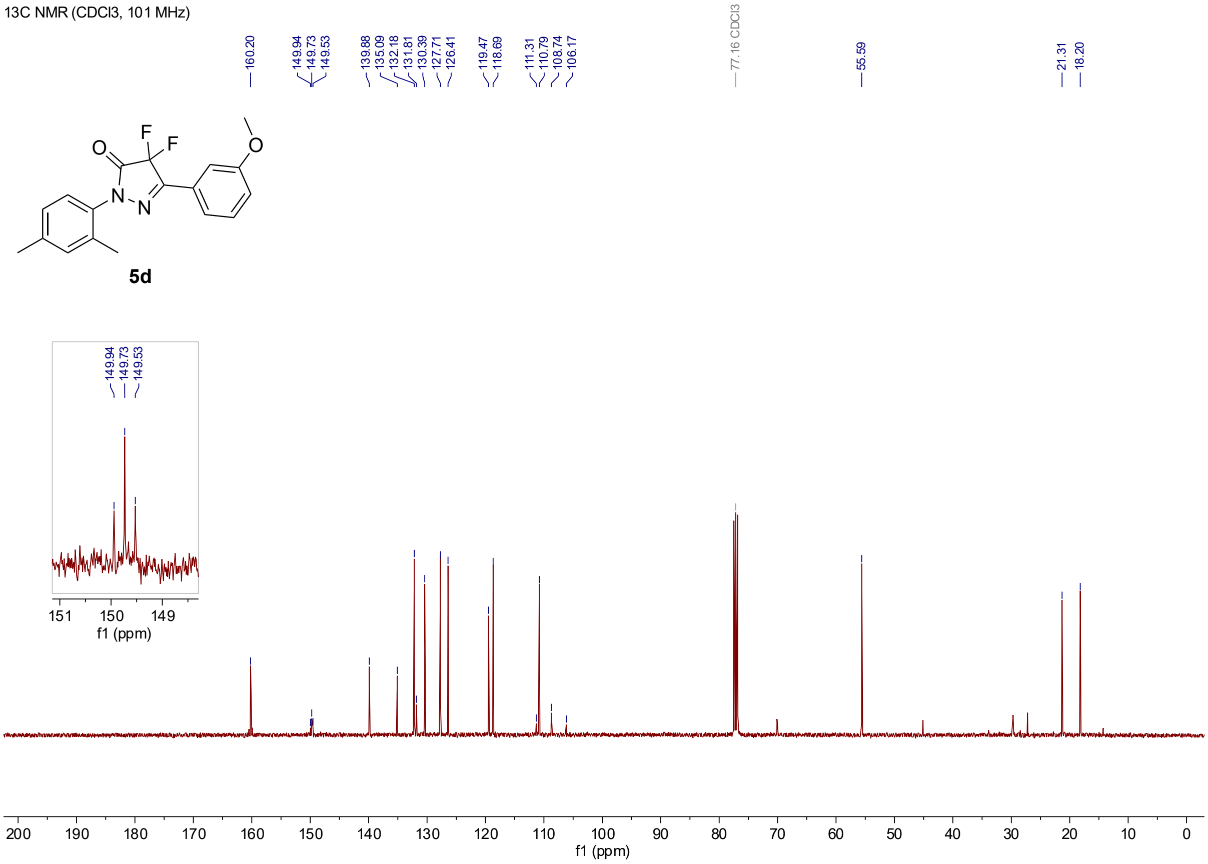

^{13}C NMR spectrum of (**5d**)

HRMS

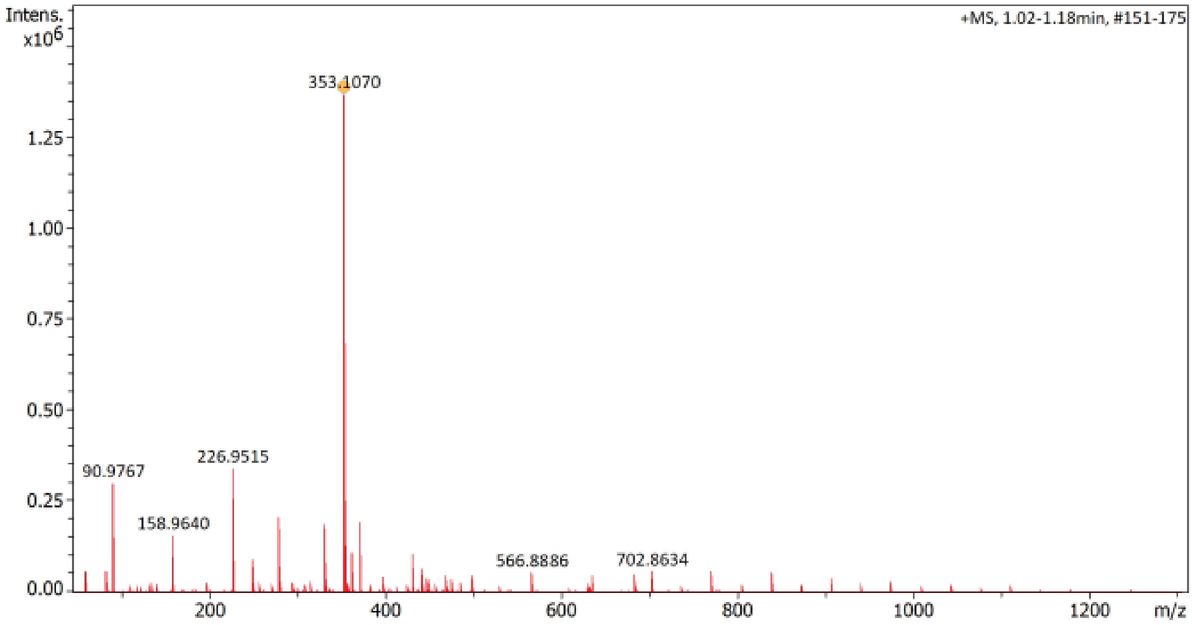

HRMS(ESI+) spectrum of (**5d**)

LC-MS

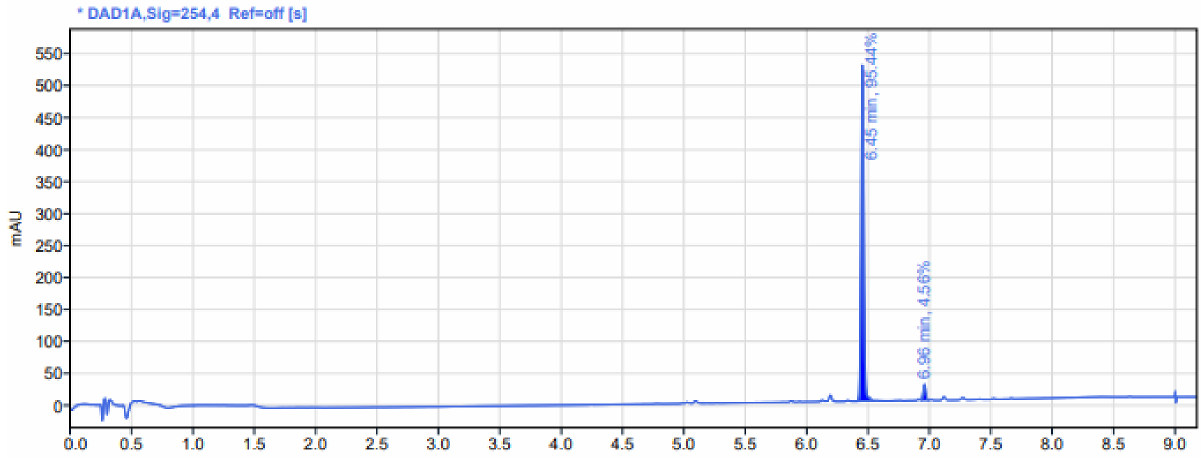

LC-MS HPLC chromatogram at 254.4 nm of (**5d**)

Characterization of (5e)

1H-NMR

1H NMR (CDCl3, 400 MHz)

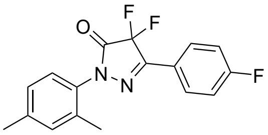

5e

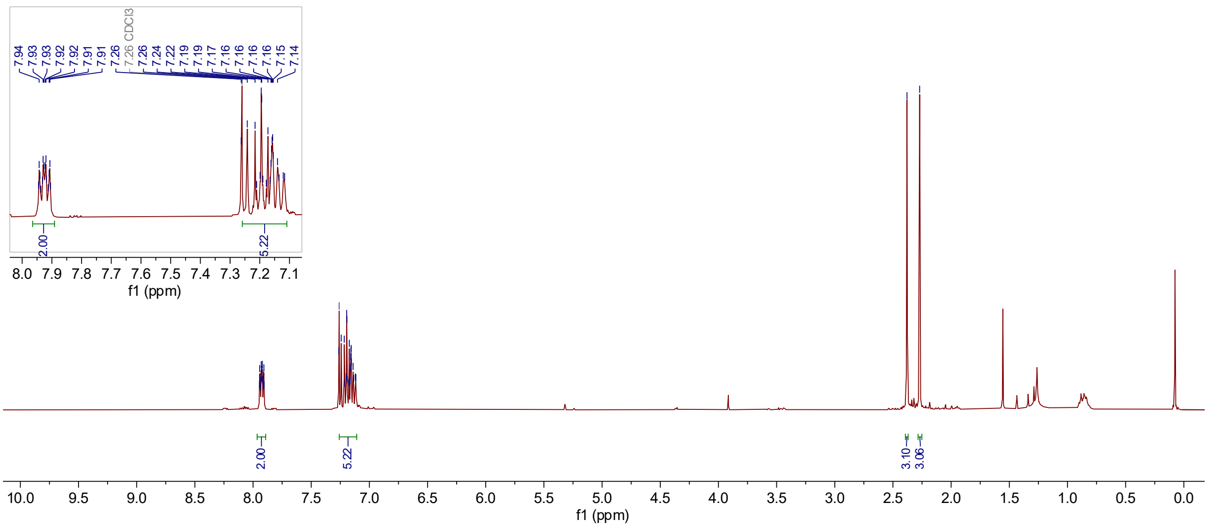

^{1}H NMR spectrum of (**5e**)

19F-NMR

19F NMR (CDCl3, 471 MHz)

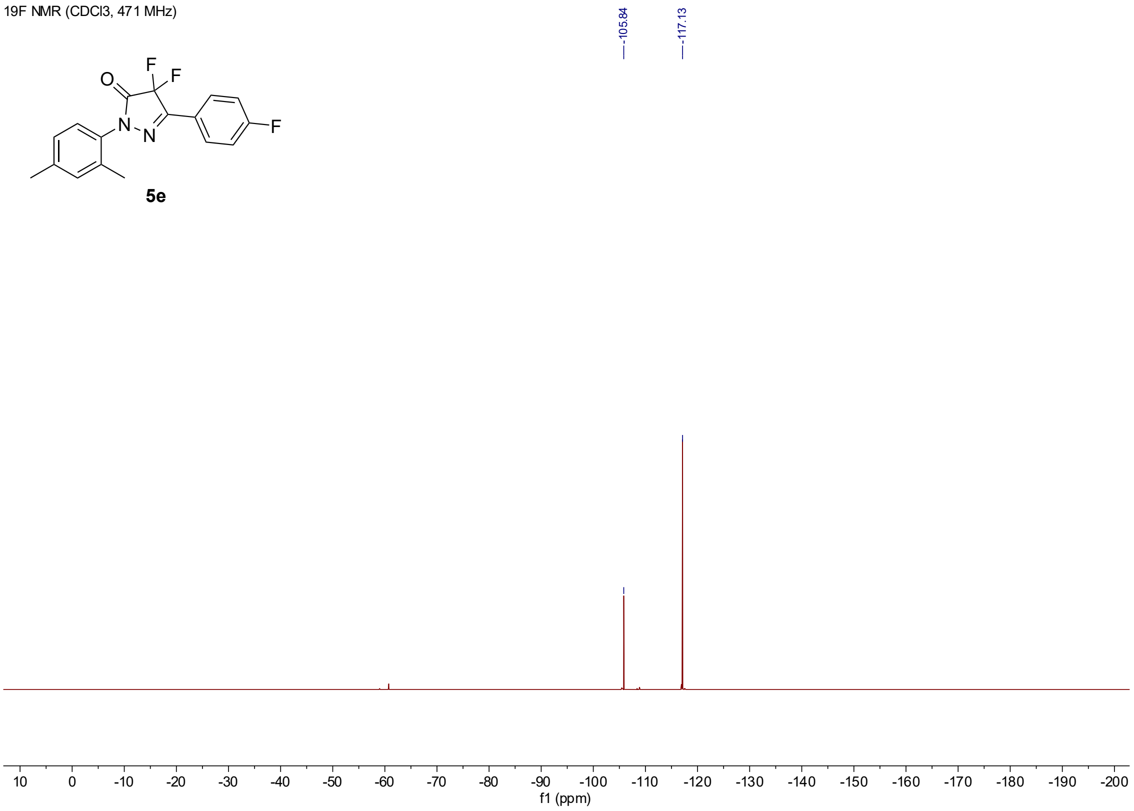

^{19}F NMR spectrum of (**5e**)

13C-NMR

13C NMR (CDCl3, 126 MHz)

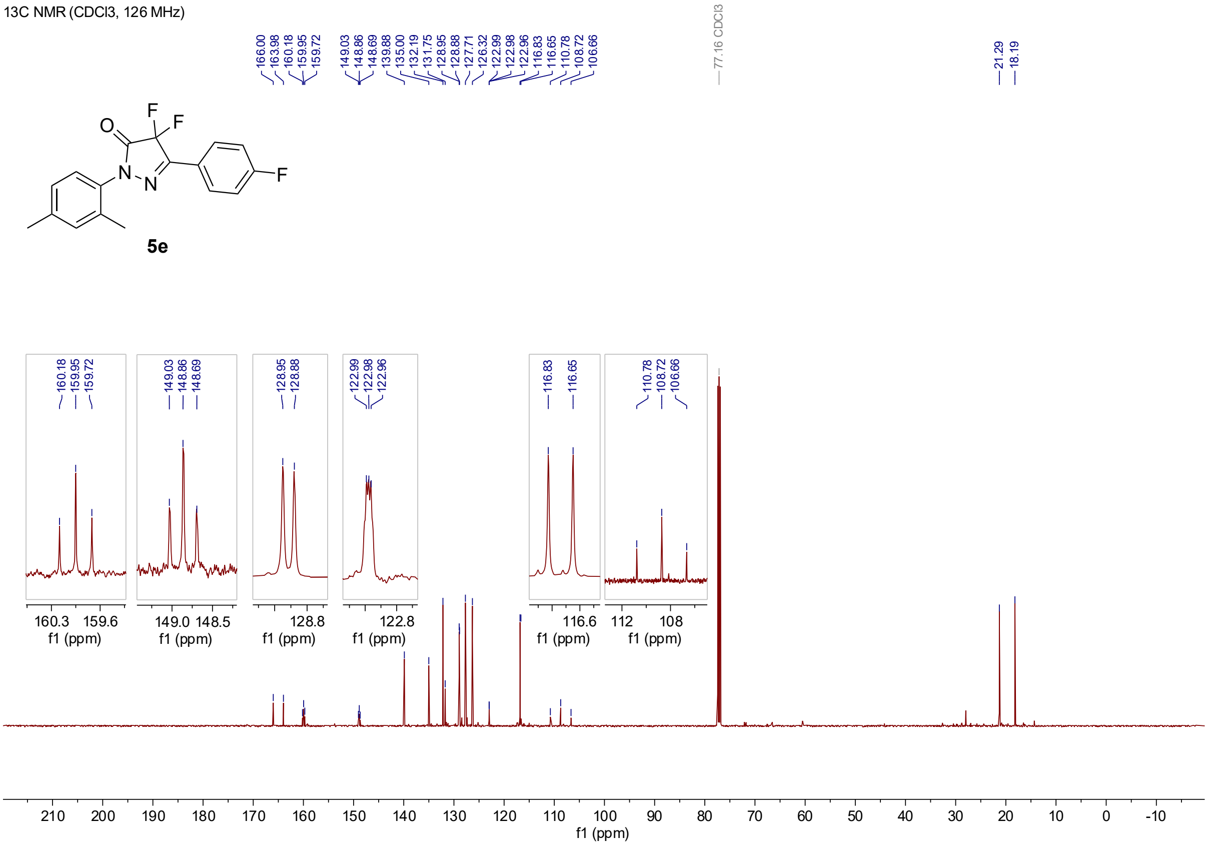

^{13}C NMR spectrum of (**5e**)

HRMS

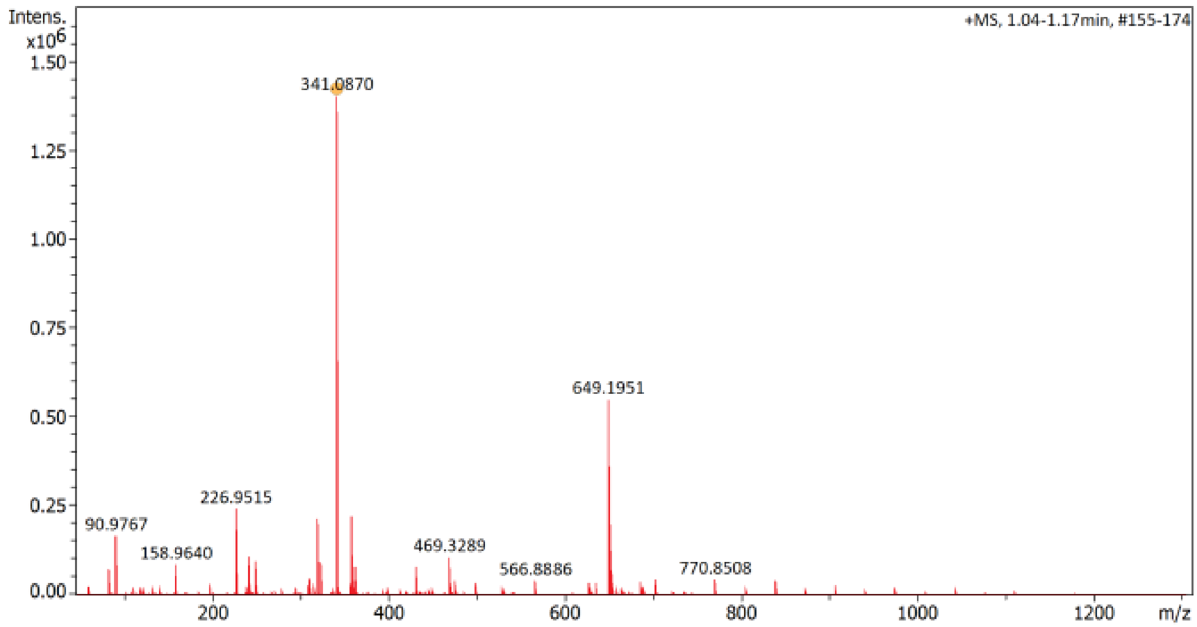

HRMS(ESI+) spectrum of (**5e**)

LC-MS

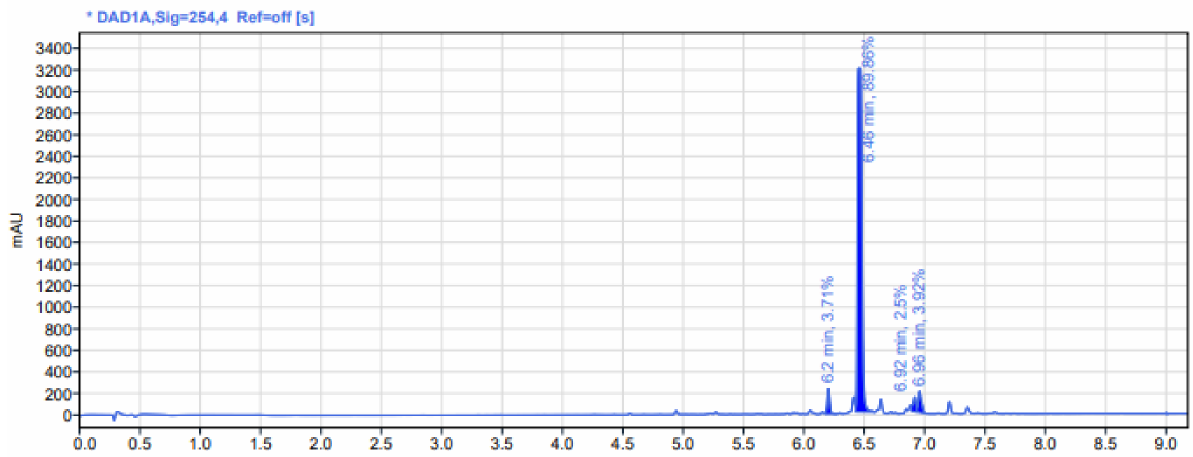

LC-MS HPLC chromatogram at 254.4 nm of (**5e**)

Characterization of tert-butyl 2-(2,4-dimethylphenyl)hydrazine-1-carboxylate

1H-NMR

1H NMR (CDCl3, 400 MHz)

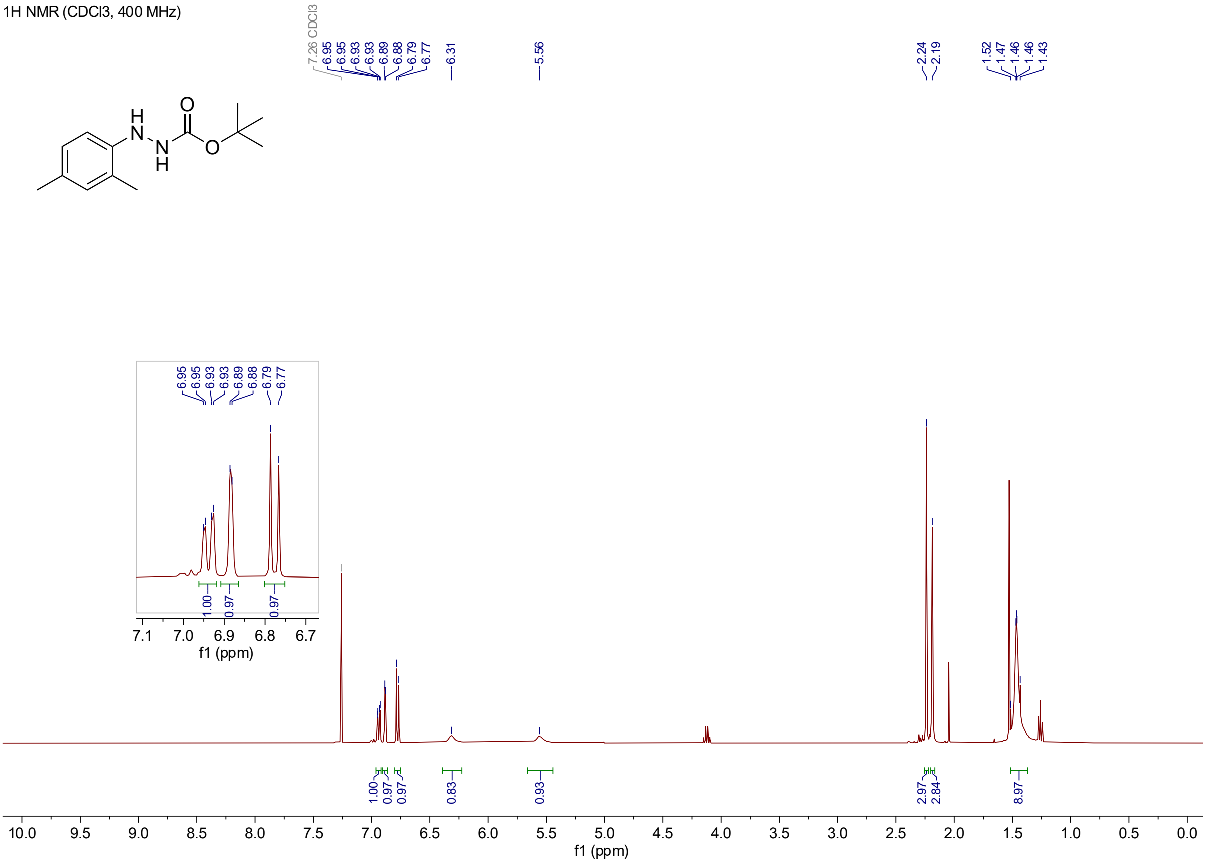

Characterization of 4-fluoro-N'-phenylbenzohydrazide

1H-NMR

1H NMR (CDCl3, 400 MHz)

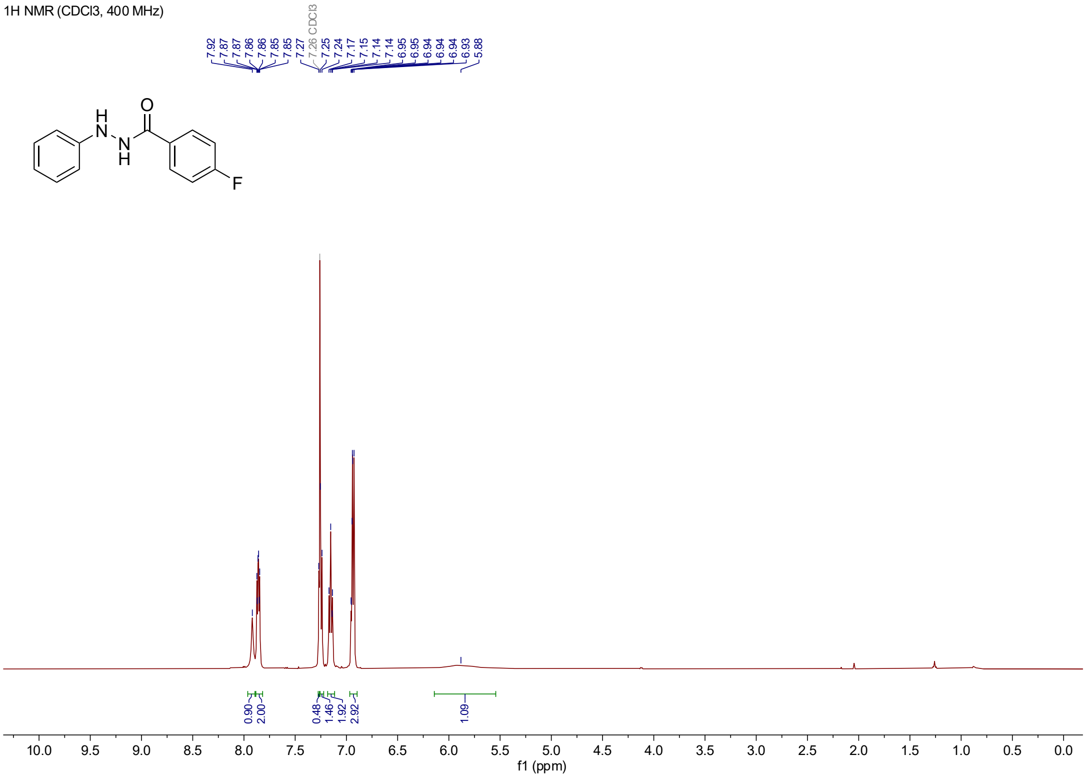

19F-NMR

19F NMR (CDCl3, 471 MHz)

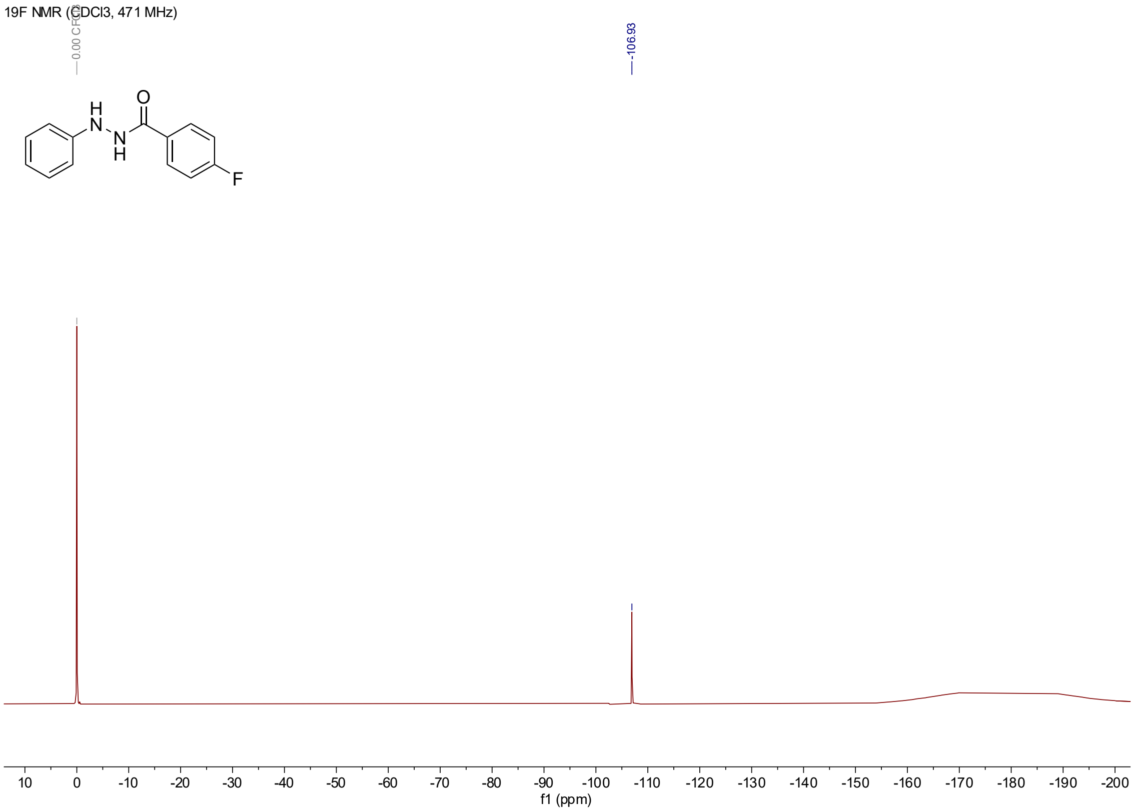

Characterization of 3-fluorobicyclo[1.1.1]pentane-1-carboxylic acid

1H-NMR

1H NMR (CDCl3, 400 MHZ)

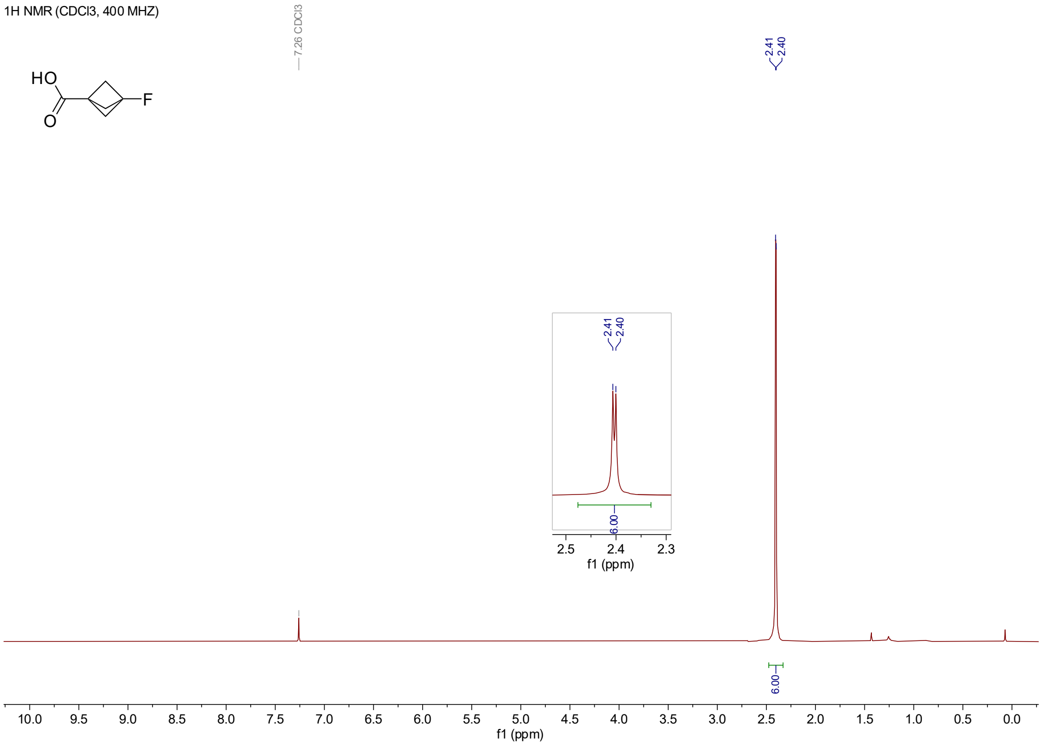

Characterization of 3-((tert-butyldimethylsilyl)oxy)benzoic acid

1H-NMR

1H NMR (CDCl3, 400 MHz)

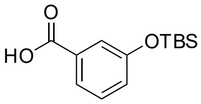

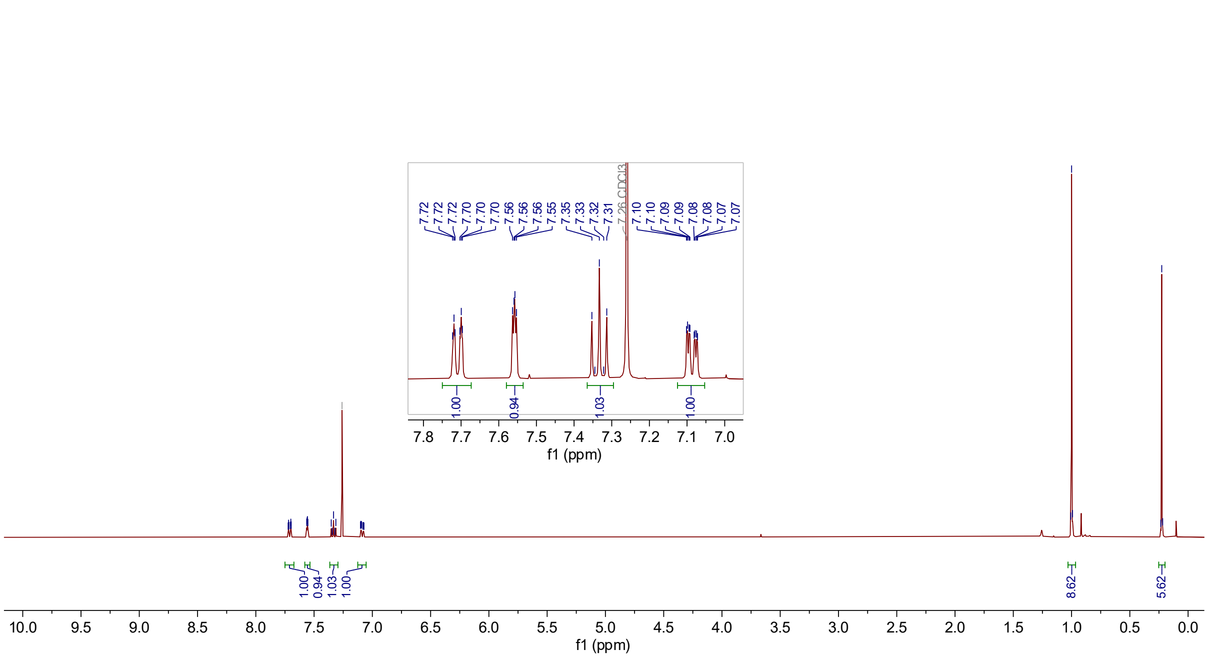

Spectra of screening experiments for synthesis of compound 5b

Screening of reaction conditions – Temperature dependency

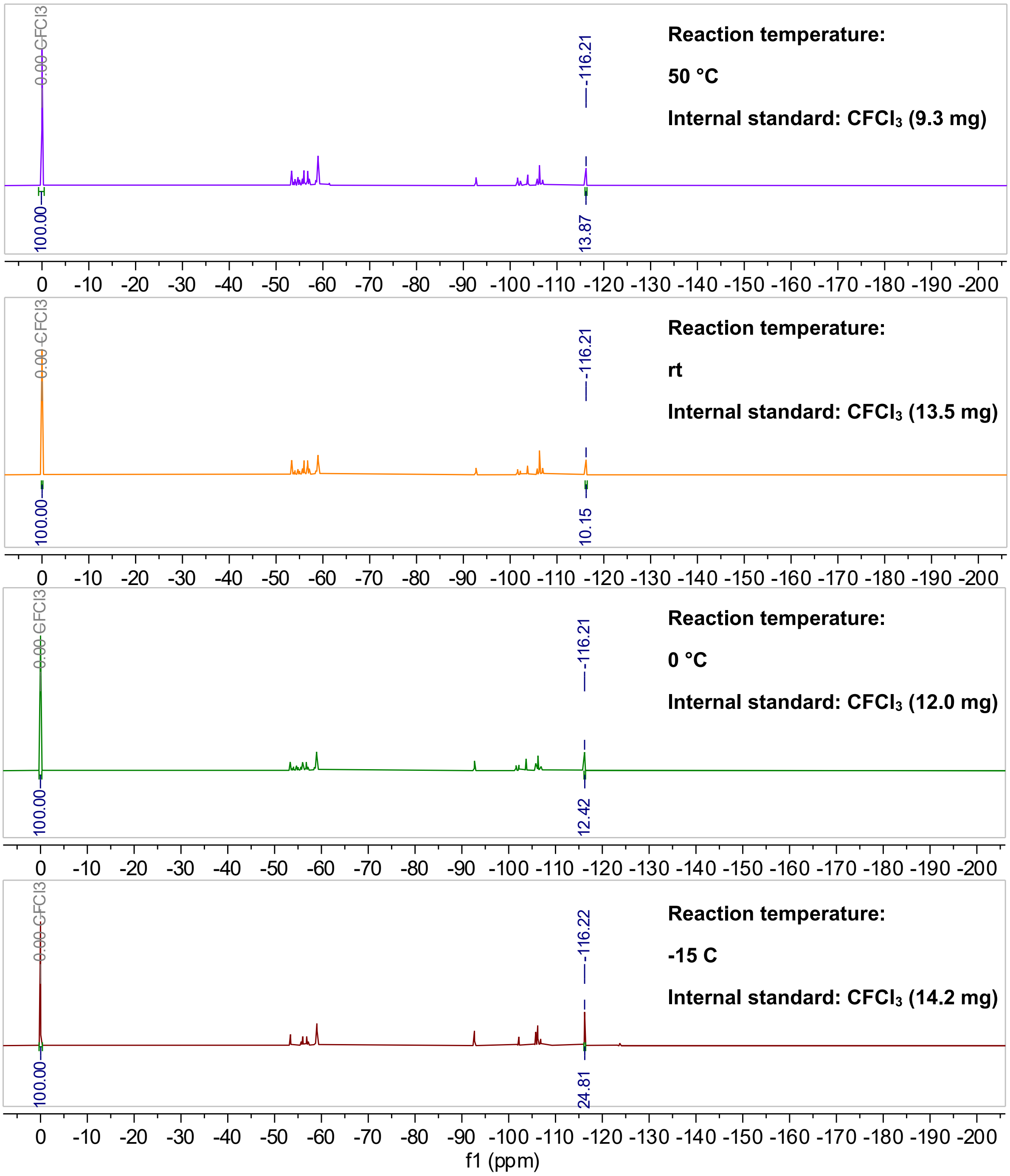

Figure S1: Optimization screening for the synthesis of **5b**. ^{19}F-NMR spectra of reactions conducted at different temperature. All reactions were conducted with following standard conditions: 0.83 mM **4b** (0.1 mmol) in THF, 1.5 eq. of AcCl (0.15 mmol), 3.0 eq. of Zn (0.75 mmol), 1 h reaction time. Integrated signal at 116.2 ppm corresponds to the 4,4-difluoro-pyrazol-3-one core of **5b**. Conversion was quantified using $CFCl_3$ as internal standard.

Screening of reaction conditions – Solvent dependency

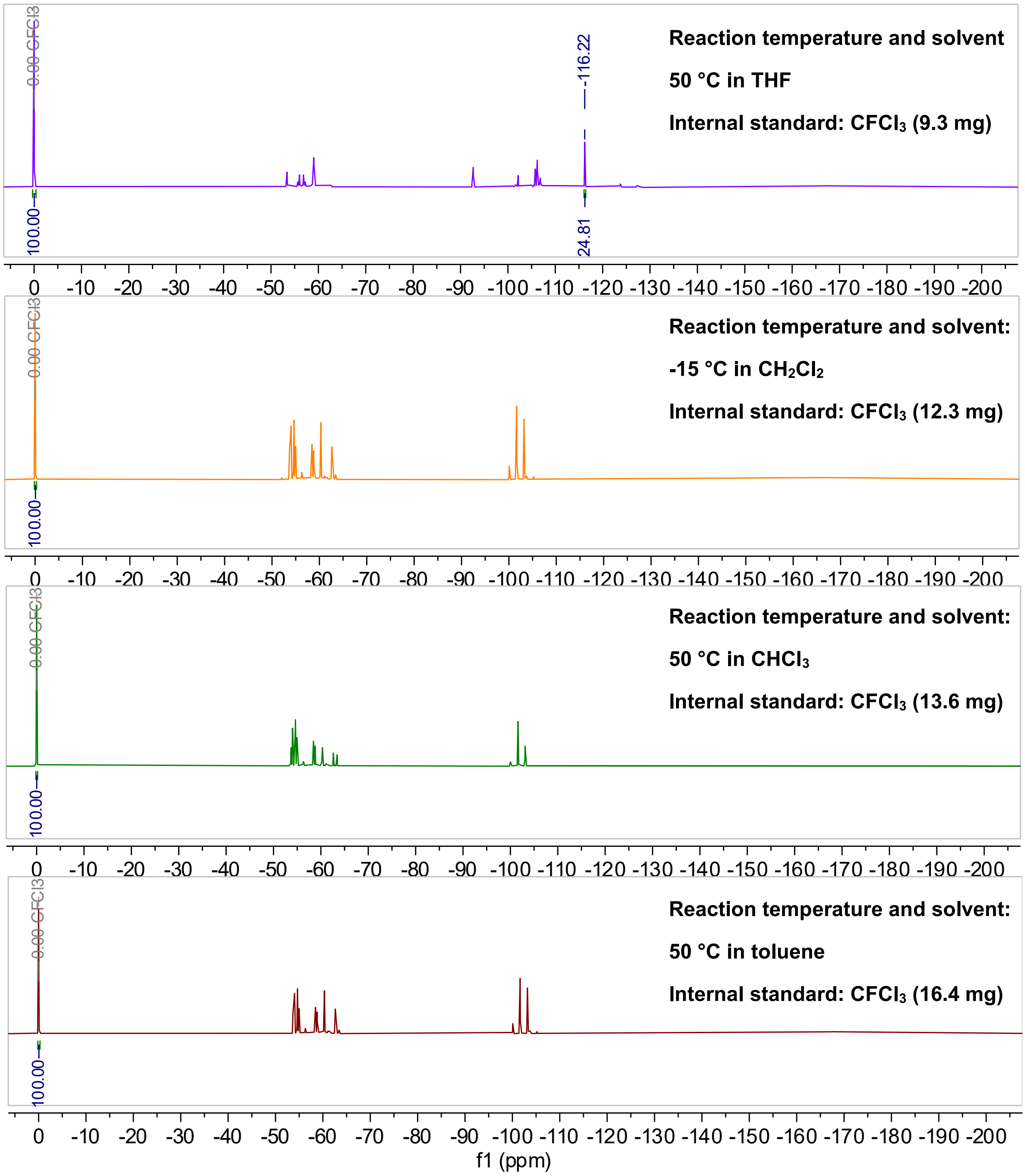

Figure S2: Optimization screening for the synthesis of **5b**. ^{19}F-NMR spectra of reactions conducted in different solvents. All reactions were conducted with following standard conditions: 0.83 mM **4b** (0.1 mmol), 1.5 eq. of AcCl (0.15 mmol), 3.0 eq. of Zn (0.75 mmol), 1 h reaction time. Integrated signal at 116.2 ppm corresponds to the 4,4-difluoro-pyrazol-3-one core of **5b**. Conversion was quantified using $CFCl_3$ as internal standard.

Screening of reaction conditions – Concentration dependency

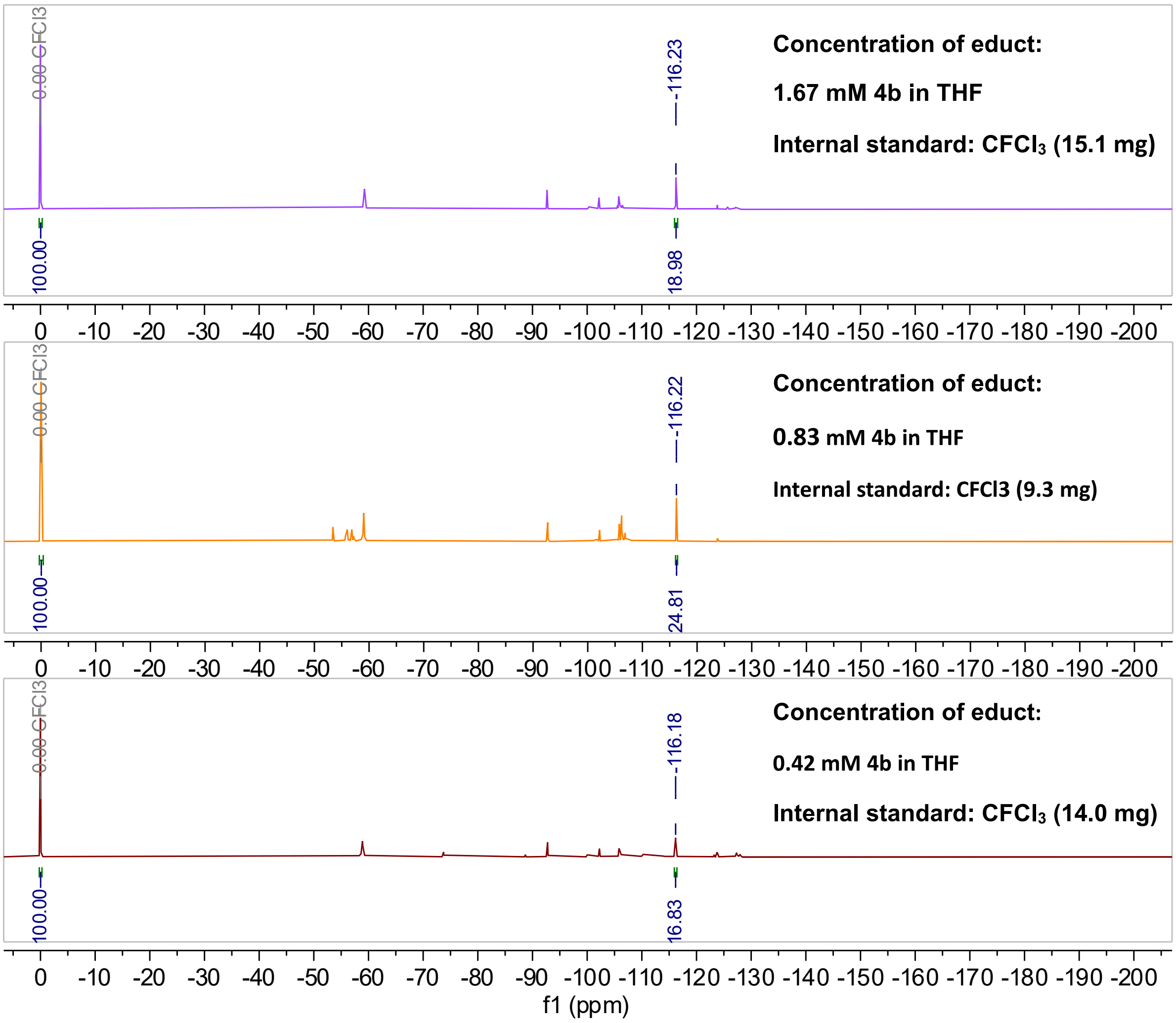

Figure S3: Optimization screening for the synthesis of **5b**. ^{19}F-NMR spectra of reactions conducted at different concentrations of educt **4b**. All reactions were conducted with following standard conditions: 1.5 eq. of AcCl, 3.0 eq. of Zn, THF, 50 °C, 1 h reaction time. Integrated signal at 116.2 ppm corresponds to the 4,4-difluoro-pyrazol-3-one core of **5b**. Conversion was quantified using $CFCl_3$ as internal standard.

Screening of reaction conditions – Reactand equivalents

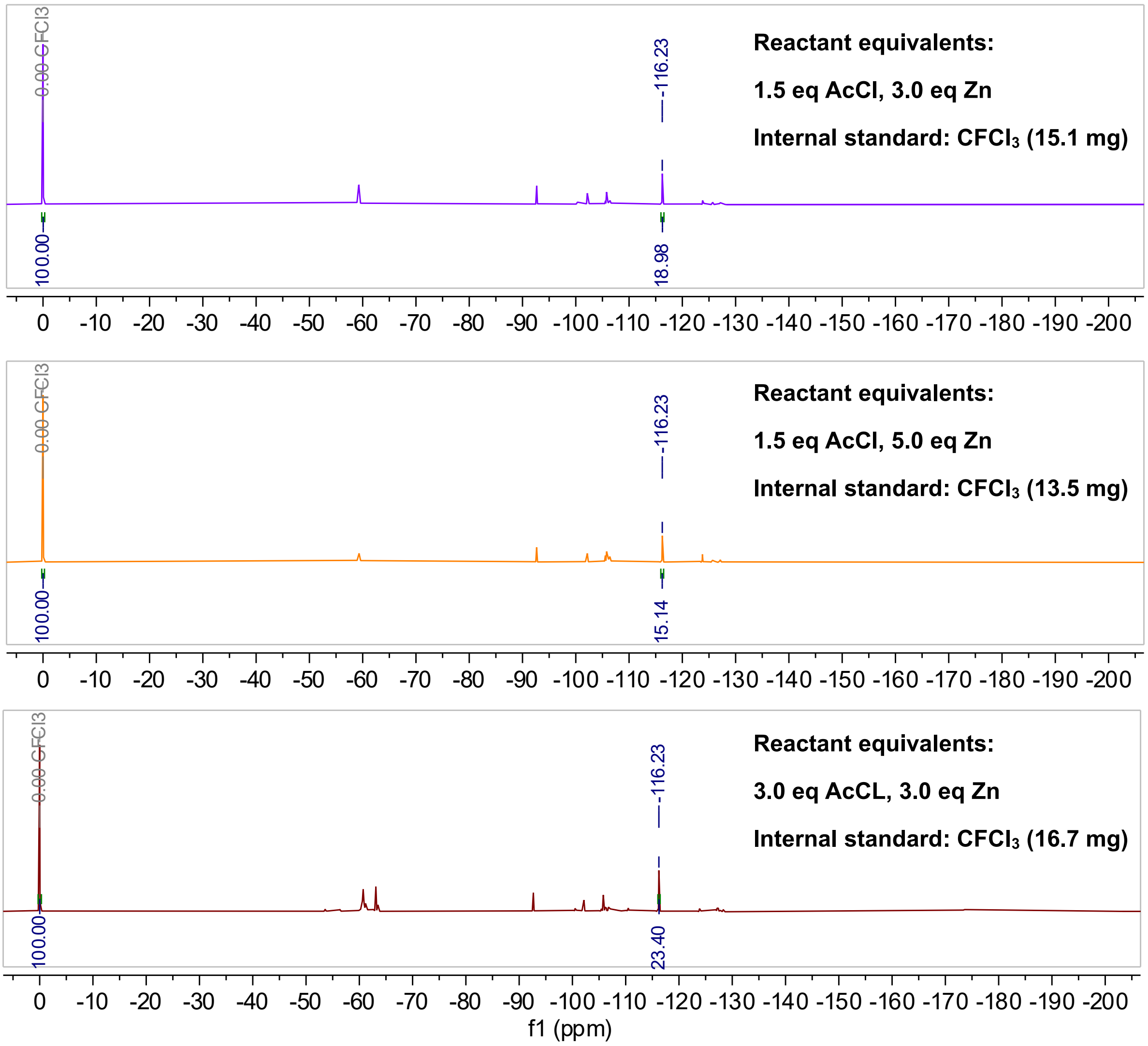

Figure S4: Optimization screening for the synthesis of **5b**. ^{19}F-NMR spectra of reactions conducted with different reagent equivalents. All reactions were conducted with following standard conditions: 1.66 mM **4b** (0.1 mmol) in THF, 50 °C, 1 h reaction time. Integrated signal at 116.2 ppm corresponds to the 4,4-difluoro-pyrazol-3-one core of **5b**. Conversion was quantified using $CFCl_3$ as internal standard.

X-ray crystallography

5b

Table S1. Crystal data and structure refinement for **5b**

**CCDC No.**	2521080
**Empirical formula**	$C_{15}H_9F_3N_2O$
**Formula weight**	290.24
**Temperature/K**	100.0(1)
**Crystal system**	triclinic
**Space group**	*P*-1 (2)
***a*/Å**	6.7585(2)

***b*/Å**	8.3151(2)
***c*/Å**	11.9615(3)
**α/°**	85.046(2)
**β/°**	79.776(2)
**γ/°**	70.592(2)
**Volume/Å^3**	623.67(3)
Z	2
**ρ_{calc} g/cm^3**	1.546
**μ/mm^{-1}**	1.122
***F*(000)**	296
**Crystal size/mm^3**	0.228×0.065×0.062
**Crystal colour**	clear yellow
**Crystal shape**	block
**Radiation**	Cu K_α (λ=1.54184)
**2Θ range/°**	7.51 to 148.52
**Index ranges**	-8 ≤ h ≤ 8, -10 ≤ k ≤ 10, -14 ≤ l ≤ 13
**Reflections collected**	17985
**Independent reflections**	2423 [R_{int} = 0.0204, R_{sigma} = 0.0110]
**Data / Restraints / Param.**	2423/0/190
**Goodness-of-fit on F^2**	1.066
**Final *R* indexes [*I*≥2σ(*I*)]**	R_1 = 0.0313, wR_2 = 0.0836
**Final *R* indexes [all data]**	R_1 = 0.0330, wR_2 = 0.0851
**Largest peak/hole /eÅ^3**	0.30/-0.26

Table S2. Fractional Atomic Coordinates (×10^4) and Equivalent Isotropic Displacement Parameters (Å^2×10^3) for **5b**. U_{eq} is defined as 1/3 of the trace of the orthogonalised U_{IJ} tensor.

**Atom**	***x***	***Y***	***z***	**U(eq)**
**F1**	1941.8(12)	3464.7(9)	9720.2(6)	27.92(19)
**F2**	2577.3(12)	1546.2(9)	4326.5(6)	26.57(19)
**F3**	5775.8(11)	1633.9(8)	4316.8(6)	25.95(18)
**O1**	4328.7(15)	2818.8(11)	2131.0(7)	29.8(2)
**N1**	2866.1(14)	5211.9(12)	3265.7(8)	17.3(2)

**N2**	2385.6(14)	5506.8(12)	4447.6(8)	17.8(2)
**C1**	1830.8(17)	5377.0(14)	6899.2(10)	19.5(2)
**C2**	1597.0(18)	5226.6(15)	8073.2(10)	22.1(3)
**C3**	2143.6(18)	3604.6(16)	8572.5(9)	20.9(2)
**C4**	2892.0(18)	2139.1(15)	7962.2(10)	21.1(2)
**C5**	3116.8(18)	2303.5(14)	6785.3(10)	20.1(2)
**C6**	2598.4(17)	3911.2(14)	6243.7(9)	17.6(2)
**C7**	2869.6(16)	4069.2(14)	5005.5(9)	16.9(2)
**C8**	3757.7(18)	2613.6(14)	4208.2(10)	19.6(2)
**C9**	3715.4(18)	3522.4(15)	3032.2(10)	20.4(2)
**C10**	2506.0(17)	6657.2(14)	2504.3(9)	17.9(2)
**C11**	2926.3(18)	6434.5(15)	1333.9(10)	21.5(2)
**C12**	2621.3(19)	7862.7(17)	609.0(10)	25.1(3)
**C13**	1889(2)	9491.0(16)	1035.3(11)	26.9(3)
**C14**	1455(2)	9690.6(15)	2200.3(11)	25.5(3)
**C15**	1755.5(18)	8284.6(15)	2943.0(10)	21.4(2)

Table S3. Anisotropic Displacement Parameters (Å^2×10^3) for **5b**. The Anisotropic displacement factor exponent takes the form: $-2\pi^2[h^2a^{*2}U_{11}+2hka^*b^*U_{12}+\ldots]$.

**Atom**	**U_{11}**	**U_{22}**	**U_{33}**	**U_{23}**	**U_{13}**	**U_{12}**
F1	34.9(4)	32.7(4)	13.9(3)	1.2(3)	-1.8(3)	-9.6(3)
F2	39.1(4)	21.9(4)	24.0(4)	-0.7(3)	-6.0(3)	-16.2(3)
F3	26.5(4)	19.8(4)	25.6(4)	-1.8(3)	-3.9(3)	0.6(3)
O1	45.5(5)	21.7(4)	19.0(4)	-6.0(3)	-0.7(4)	-7.6(4)
N1	20.4(5)	18.2(5)	13.0(4)	-2.0(3)	-2.0(3)	-5.8(4)
N2	18.9(4)	20.4(5)	13.9(5)	-2.6(4)	-1.8(4)	-6.1(4)
C1	20.3(5)	18.9(5)	18.5(5)	0.0(4)	-1.9(4)	-5.9(4)
C2	23.5(6)	23.2(6)	19.0(6)	-4.2(4)	-0.7(4)	-7.2(5)
C3	20.5(5)	28.8(6)	13.7(5)	0.9(4)	-2.1(4)	-9.2(5)
C4	21.5(5)	21.4(6)	20.2(6)	3.6(4)	-4.1(4)	-7.3(4)

C5	21.0(5)	18.9(6)	20.3(6)	-2.2(4)	-3.0(4)	-5.9(4)
C6	16.3(5)	20.0(6)	16.8(5)	-1.1(4)	-2.7(4)	-6.2(4)
C7	16.0(5)	17.6(5)	17.5(5)	-1.0(4)	-2.8(4)	-5.8(4)
C8	22.3(5)	17.4(5)	19.3(6)	-0.9(4)	-3.8(4)	-6.4(4)
C9	23.7(6)	18.7(5)	18.9(6)	-1.8(4)	-3.1(4)	-6.9(4)
C10	16.6(5)	19.4(5)	18.3(6)	1.5(4)	-4.1(4)	-6.4(4)
C11	22.0(5)	23.6(6)	18.9(6)	-1.5(4)	-3.4(4)	-7.1(5)
C12	26.9(6)	31.1(7)	17.6(6)	1.8(5)	-4.9(5)	-9.6(5)
C13	31.3(6)	25.8(6)	24.3(6)	7.3(5)	-8.7(5)	-10.1(5)
C14	31.5(6)	19.4(6)	25.6(6)	0.7(5)	-7.9(5)	-6.6(5)
C15	24.5(6)	21.6(6)	18.2(5)	-0.7(4)	-4.9(4)	-6.9(5)

Table S4. Bond Lengths for **5b**

Atom	Atom	Length/Å	Atom	Atom	Length/Å
F1	C3	1.3540(13)	C4	C5	1.3885(16)
F2	C8	1.3612(13)	C5	C6	1.3962(16)
F3	C8	1.3590(13)	C6	C7	1.4596(15)
O1	C9	1.2105(14)	C7	C8	1.4994(15)
N1	N2	1.4171(12)	C8	C9	1.5385(16)
N1	C9	1.3624(15)	C10	C11	1.3941(16)
N1	C10	1.4241(14)	C10	C15	1.3913(16)
N2	C7	1.2888(14)	C11	C12	1.3884(17)
C1	C2	1.3844(16)	C12	C13	1.3866(18)
C1	C6	1.4035(16)	C13	C14	1.3849(17)
C2	C3	1.3857(17)	C14	C15	1.3871(16)
C3	C4	1.3736(17)			

Table S5. Bond Angles for **5b**

Atom	Atom	Atom	Angle/˚	Atom	Atom	Atom	Angle/˚
N2	N1	C10	117.90(9)	F2	C8	C7	113.47(9)

C9	N1	N2	112.69(9)	F2	C8	C9	110.20(9)
C9	N1	C10	129.34(10)	F3	C8	F2	106.88(9)
C7	N2	N1	109.56(9)	F3	C8	C7	113.31(9)
C2	C1	C6	120.16(11)	F3	C8	C9	110.17(9)
C1	C2	C3	118.31(11)	C7	C8	C9	102.82(9)
F1	C3	C2	118.07(10)	O1	C9	N1	130.47(11)
F1	C3	C4	118.59(10)	O1	C9	C8	125.25(11)
C4	C3	C2	123.33(11)	N1	C9	C8	104.28(9)
C3	C4	C5	117.92(11)	C11	C10	N1	120.10(10)
C4	C5	C6	120.81(11)	C15	C10	N1	119.22(10)
C1	C6	C7	120.18(10)	C15	C10	C11	120.68(10)
C5	C6	C1	119.47(10)	C12	C11	C10	118.99(11)
C5	C6	C7	120.35(10)	C13	C12	C11	120.88(11)
N2	C7	C6	123.85(10)	C14	C13	C12	119.40(11)
N2	C7	C8	110.64(10)	C13	C14	C15	120.86(11)
C6	C7	C8	125.51(10)	C14	C15	C10	119.18(11)

Table S6. Torsion Angles for **5b**

**A**	**B**	**C**	**D**	**Angle/˚**	**A**	**B**	**C**	**D**	**Angle/˚**
F1	C3	C4	C5	-179.16(10)	C3	C4	C5	C6	0.08(17)
F2	C8	C9	O1	-59.77(15)	C4	C5	C6	C1	-0.33(17)
F2	C8	C9	N1	120.37(10)	C4	C5	C6	C7	179.12(10)
F3	C8	C9	O1	57.92(15)	C5	C6	C7	N2	178.53(10)
F3	C8	C9	N1	-121.94(10)	C5	C6	C7	C8	-2.18(17)
N1	N2	C7	C6	179.31(9)	C6	C1	C2	C3	0.18(17)
N1	N2	C7	C8	-0.07(12)	C6	C7	C8	F2	62.23(14)
N1	C10	C11	C12	-178.13(10)	C6	C7	C8	F3	-59.89(14)
N1	C10	C15	C14	178.44(10)	C6	C7	C8	C9	-178.77(10)
N2	N1	C9	O1	-178.92(12)	C7	C8	C9	O1	178.97(11)

N2	N1	C9	C8	0.92(12)	C7	C8	C9	N1	-0.89(11)
N2	N1	C10	C11	-179.13(9)	C9	N1	N2	C7	-0.59(12)
N2	N1	C10	C15	1.58(15)	C9	N1	C10	C11	4.04(18)
N2	C7	C8	F2	-118.41(10)	C9	N1	C10	C15	-175.25(11)
N2	C7	C8	F3	119.47(10)	C10	N1	N2	C7	-177.92(9)
N2	C7	C8	C9	0.60(12)	C10	N1	C9	O1	-2.0(2)
C1	C2	C3	F1	179.04(10)	C10	N1	C9	C8	177.88(10)
C1	C2	C3	C4	-0.44(18)	C10	C11	C12	C13	-0.67(18)
C1	C6	C7	N2	-2.02(17)	C11	C10	C15	C14	-0.85(17)
C1	C6	C7	C8	177.26(10)	C11	C12	C13	C14	-0.09(19)
C2	C1	C6	C5	0.20(17)	C12	C13	C14	C15	0.40(19)
C2	C1	C6	C7	-179.25(10)	C13	C14	C15	C10	0.06(18)
C2	C3	C4	C5	0.31(18)	C15	C10	C11	C12	1.15(17)

Table S7. Hydrogen Atom Coordinates (Å×10^4) and Isotropic Displacement Parameters (Å^2×10^3) for **5b.**

**Atom**	***x***	***y***	***z***	**U(eq)**
H1	1470.86	6475.01	6536.39	23
H2	1075.13	6210.35	8524.99	27
H4	3244.84	1047.32	8333.56	25
H5	3629.5	1309.8	6343.27	24
H11	3414.14	5322.47	1036.94	26
H12	2918.33	7722.34	-189.87	30
H13	1686.66	10460.9	532.81	32
H14	943.8	10805.09	2494.7	31
H15	1452.93	8431.11	3741.19	26

9a

9a was crystalized by vapor diffusion from $CHCl_3$ and hexane.

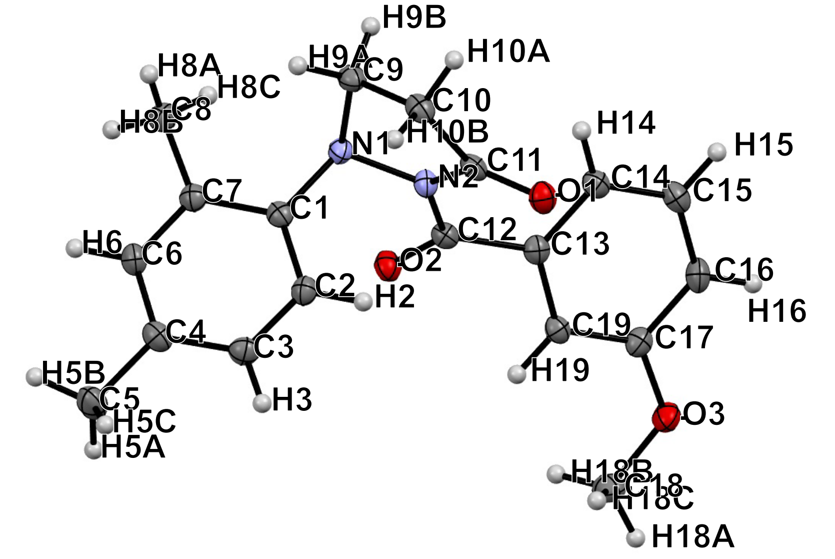

Figure S5: Determined X-ray crystal structure of **9a**

Table S8: Crystal data and structure refinement for **9a**

**CCDC No.**	**2564048**
**Empirical formula**	$C_{19}H_{20}N_2O_3$
**Formula weight**	324.37
**Temperature/K**	100.0(1)
**Crystal system**	monoclinic
**Space group**	$P2_1/c$ (14)
***a*/Å**	11.0356(1)
***b*/Å**	14.3557(2)
***c*/Å**	10.3816(1)
**α/°**	90
**β/°**	94.2410(10)
**γ/°**	90
**Volume/Å^3**	1640.19(3)
Z	4
**ρ_{calc} g/cm^3**	1.314
**μ/mm^{-1}**	0.727
***F*(000)**	688
**Crystal size/mm^3**	0.222×0.149×0.052

**Crystal colour**	clear light orange
**Crystal shape**	block
**Radiation**	Cu K_{α} (λ=1.54184)
**2Θ range/°**	8.03 to 159.42
**Index ranges**	-13 ≤ h ≤ 13, -18 ≤ k ≤ 18, -12 ≤ l ≤ 13
**Reflections collected**	36732
**Independent reflections**	3379 [R_{int} = 0.0324, R_{sigma} = 0.0152]
**Data / Restraints / Param.**	3379/0/220
**Goodness-of-fit on F^2**	1.051
**Final *R* indexes [*I*≥2σ(*I*)]**	R_1 = 0.0374, wR_2 = 0.0979
**Final *R* indexes [all data]**	R_1 = 0.0401, wR_2 = 0.0997
**Largest peak/hole /eÅ^3**	0.28/-0.20

Table S9: Fractional Atomic Coordinates (×10^4) and Equivalent Isotropic Displacement Parameters (Å^2×10^3) for **9a**. U_{eq} is defined as 1/3 of the trace of the orthogonalised U_{IJ} tensor.

**Atom**	**x**	**y**	**z**	**U(eq)**
**O1**	3984.7(8)	3855.2(6)	8652.1(8)	22.4(2)
**O2**	5475.7(8)	1811.3(6)	6444.0(8)	22.3(2)
**O3**	1012.0(8)	782.8(7)	6200.1(9)	24.3(2)
**N1**	6837.1(9)	3071.9(7)	7808.9(9)	17.8(2)
**N2**	5560.5(9)	2961.2(7)	7941.8(9)	17.4(2)
**C1**	7090.9(11)	3478.2(8)	6578.7(11)	17.8(2)
**C2**	6210.5(11)	3911.2(8)	5757.7(12)	19.8(3)
**C3**	6488.7(11)	4246.8(9)	4560.5(12)	22.0(3)
**C4**	7659.1(12)	4168.0(9)	4156.3(12)	22.4(3)
**C5**	7951.7(13)	4520.5(11)	2848.0(13)	30.4(3)
**C6**	8529.9(11)	3738.8(9)	4993.7(12)	21.5(3)
**C7**	8276.8(11)	3384.1(8)	6199.0(12)	19.6(3)
**C8**	9261.4(12)	2895.2(10)	7028.3(13)	26.0(3)
**C9**	7202.1(11)	3681.5(9)	8934.9(12)	20.7(3)
**C10**	6115.4(11)	4321.6(9)	9063.3(12)	21.2(3)

**C11**	5055.8(11)	3724.0(8)	8574.1(11)	18.2(2)
**C12**	4978.9(11)	2214.8(8)	7291.4(11)	17.5(2)
**C13**	3771.5(11)	1911.4(8)	7696.4(11)	17.5(2)
**C14**	3532.4(11)	1853.3(8)	8987.9(12)	19.8(3)
**C15**	2474.1(12)	1419.8(9)	9317.4(12)	22.2(3)
**C16**	1654.1(11)	1063.7(9)	8368.0(12)	22.4(3)
**C17**	1879.8(11)	1151.7(8)	7069.8(12)	19.4(3)
**C18**	1184.0(13)	920.1(10)	4863.1(13)	28.8(3)
**C19**	2947.0(11)	1565.2(8)	6724.2(11)	18.0(2)

Table S10: Anisotropic Displacement Parameters (Å^2×10^3) for **9a**. The Anisotropic displacement factor exponent takes the form: $-2\pi^2[h^2a^{*2}U_{11}+2hka^*b^*U_{12}+…]$.

Atom	U11	U22	U33	U23	U13	U12
**O1**	22.4(5)	20.7(4)	24.7(4)	-0.6(3)	5.1(3)	2.2(3)
**O2**	22.2(4)	23.9(4)	21.5(4)	-5.7(3)	5.7(3)	-1.6(3)
**O3**	19.6(4)	28.8(5)	24.7(5)	-4.5(4)	1.9(3)	-5.4(4)
**N1**	15.2(5)	20.7(5)	17.7(5)	-1.3(4)	2.3(4)	-3.2(4)
**N2**	16.1(5)	17.7(5)	18.9(5)	-1.3(4)	4.3(4)	-1.6(4)
**C1**	19.9(6)	16.3(5)	17.2(5)	-1.5(4)	2.1(4)	-2.6(4)
**C2**	17.9(6)	20.2(6)	21.8(6)	-1.6(5)	3.4(5)	0.0(5)
**C3**	23.5(6)	20.8(6)	21.6(6)	2.4(5)	0.5(5)	1.4(5)
**C4**	26.5(6)	20.5(6)	20.4(6)	0.5(5)	4.4(5)	-0.5(5)
**C5**	31.1(7)	35.9(7)	25.0(7)	9.1(6)	7.4(5)	4.5(6)
**C6**	19.6(6)	22.9(6)	22.6(6)	0.5(5)	5.9(5)	-0.9(5)
**C7**	19.5(6)	18.9(6)	20.3(6)	-1.2(4)	1.9(5)	-1.0(5)
**C8**	19.5(6)	34.5(7)	24.1(6)	4.0(5)	2.1(5)	3.0(5)
**C9**	22.1(6)	22.9(6)	17.1(6)	-1.9(5)	1.0(4)	-4.2(5)
**C10**	26.2(6)	18.7(6)	19.2(6)	-1.9(4)	4.4(5)	-3.8(5)
**C11**	23.6(6)	16.3(5)	14.9(5)	1.7(4)	3.6(4)	-0.1(5)
**C12**	20.1(6)	16.5(5)	16.0(5)	0.1(4)	1.7(4)	0.1(4)

**C13**	18.6(6)	14.0(5)	20.3(6)	-0.2(4)	3.3(4)	1.2(4)
**C14**	21.9(6)	18.3(6)	19.3(6)	-1.1(4)	2.4(5)	1.3(5)
**C15**	26.3(6)	21.7(6)	19.3(6)	0.3(5)	7.1(5)	0.9(5)
**C16**	19.3(6)	21.4(6)	27.5(6)	-0.6(5)	8.6(5)	-2.0(5)
**C17**	17.7(6)	16.8(5)	23.7(6)	-3.4(4)	1.5(5)	0.6(4)
**C18**	28.2(7)	34.3(7)	23.2(6)	-0.9(5)	-2.4(5)	-7.1(6)
**C19**	20.4(6)	16.0(5)	17.8(5)	-0.7(4)	3.7(4)	1.2(4)

Table S11: Bond Lengths for **9a**

**Atom**	**Atom**	**Length/Å**	**Atom**	**Atom**	**Length/Å**
O1	C11	1.2056(15)	C4	C5	1.5066(17)
O2	C12	1.2171(15)	C4	C6	1.3912(18)
O3	C17	1.3729(14)	C6	C7	1.3981(17)
O3	C18	1.4285(16)	C7	C8	1.5087(17)
N1	N2	1.4346(13)	C9	C10	1.5244(17)
N1	C1	1.4500(15)	C10	C11	1.5078(17)
N1	C9	1.4917(15)	C12	C13	1.4916(16)
N2	C11	1.4121(15)	C13	C14	1.3880(17)
N2	C12	1.3971(15)	C13	C19	1.3993(16)
C1	C2	1.3907(17)	C14	C15	1.3884(18)
C1	C7	1.4009(17)	C15	C16	1.3855(18)
C2	C3	1.3883(17)	C16	C17	1.3943(18)
C3	C4	1.3918(18)	C17	C19	1.3895(17)

Table S12: Bond Angles for **9a**

**Atom**	**Atom**	**Atom**	**Angle/˚**	**Atom**	**Atom**	**Atom**	**Angle/˚**
C17	O3	C18	116.70(10)	N1	C9	C10	104.69(9)
N2	N1	C1	112.60(9)	C11	C10	C9	102.89(10)
N2	N1	C9	101.52(9)	O1	C11	N2	125.08(11)
C1	N1	C9	113.31(9)	O1	C11	C10	128.92(11)

C11	N2	N1	112.50(9)	N2	C11	C10	105.97(10)
C12	N2	N1	117.24(9)	O2	C12	N2	119.94(11)
C12	N2	C11	129.54(10)	O2	C12	C13	121.62(11)
C2	C1	N1	123.22(11)	N2	C12	C13	118.41(10)
C2	C1	C7	119.69(11)	C14	C13	C12	121.83(11)
C7	C1	N1	117.02(10)	C14	C13	C19	121.03(11)
C3	C2	C1	120.87(11)	C19	C13	C12	116.56(10)
C2	C3	C4	120.89(11)	C13	C14	C15	119.24(11)
C3	C4	C5	120.67(11)	C16	C15	C14	120.47(11)
C6	C4	C3	117.46(11)	C15	C16	C17	120.02(11)
C6	C4	C5	121.86(12)	O3	C17	C16	115.78(11)
C4	C6	C7	123.10(12)	O3	C17	C19	123.94(11)
C1	C7	C8	122.18(11)	C19	C17	C16	120.24(11)
C6	C7	C1	118.00(11)	C17	C19	C13	118.94(11)
C6	C7	C8	119.80(11)				

Table S13: Torsion Angles for **9a**

**A**	**B**	**C**	**D**	**Angle/˚**	**A**	**B**	**C**	**D**	**Angle/˚**
O2	C12	C13	C14	136.07(12)	C4	C6	C7	C1	-0.74(19)
O2	C12	C13	C19	-35.35(17)	C4	C6	C7	C8	177.63(12)
O3	C17	C19	C13	-179.50(11)	C5	C4	C6	C7	-178.33(12)
N1	N2	C11	O1	169.75(11)	C7	C1	C2	C3	0.45(18)
N1	N2	C11	C10	-8.45(12)	C9	N1	N2	C11	27.53(12)
N1	N2	C12	O2	-15.26(16)	C9	N1	N2	C12	-161.28(10)
N1	N2	C12	C13	162.50(10)	C9	N1	C1	C2	-100.28(13)
N1	C1	C2	C3	-176.68(11)	C9	N1	C1	C7	82.52(13)
N1	C1	C7	C6	177.57(10)	C9	C10	C11	O1	167.87(12)
N1	C1	C7	C8	-0.77(17)	C9	C10	C11	N2	-14.03(12)
N1	C9	C10	C11	30.54(12)	C11	N2	C12	O2	154.17(12)

N2	N1	C1	C2	14.24(15)	C11	N2	C12	C13	-28.07(17)
N2	N1	C1	C7	-162.96(10)	C12	N2	C11	O1	-0.09(19)
N2	N1	C9	C10	-34.95(11)	C12	N2	C11	C10	-178.28(11)
N2	C12	C13	C14	-41.65(16)	C12	C13	C14	C15	-169.13(11)
N2	C12	C13	C19	146.93(11)	C12	C13	C19	C17	170.97(11)
C1	N1	N2	C11	-93.96(11)	C13	C14	C15	C16	-1.16(19)
C1	N1	N2	C12	77.23(12)	C14	C13	C19	C17	-0.52(17)
C1	N1	C9	C10	86.04(11)	C14	C15	C16	C17	-0.97(19)
C1	C2	C3	C4	-0.72(19)	C15	C16	C17	O3	-179.59(11)
C2	C1	C7	C6	0.26(17)	C15	C16	C17	C19	2.38(18)
C2	C1	C7	C8	-178.07(11)	C16	C17	C19	C13	-1.63(18)
C2	C3	C4	C5	179.08(12)	C18	O3	C17	C16	175.92(11)
C2	C3	C4	C6	0.26(19)	C18	O3	C17	C19	-6.13(17)
C3	C4	C6	C7	0.48(19)	C19	C13	C14	C15	1.91(18)

Table S14: Hydrogen Atom Coordinates (Å×10^4) and Isotropic Displacement Parameters (Å^2×10^3) for **9a**

**Atom**	**x**	**y**	**z**	**U(eq)**
H2	5408.31	3978.21	6019.54	24
H3	5872	4533.98	4009.87	26
H5A	7609.89	4094.88	2178.21	46
H5B	8835.29	4553.69	2809.55	46
H5C	7600.34	5142.22	2705.35	46
H6	9334.57	3684.15	4734.35	26
H8A	9721.67	3353.75	7565.38	39
H8B	9809.37	2574.35	6474.06	39
H8C	8892.58	2439.93	7584.01	39
H9A	7936.95	4046.49	8774.01	25
H9B	7369.7	3306.06	9727.99	25
H10A	6046.41	4506.22	9973.77	25

H10B	6175.15	4888.78	8529.26	25
H14	4086.63	2107.25	9638.85	24
H15	2310.91	1366.97	10200.21	27
H16	937.95	759.56	8601.6	27
H18A	507.79	636.32	4337.41	43
H18B	1948.99	629.21	4655.6	43
H18C	1213.18	1588.93	4679.04	43
H19	3114.14	1612.14	5841.89	22

4d

4d was crystallized by vapor diffusion from diethyl ether and hexane.

Table S15: Crystal data and structure refinement for **4d**

**CCDC No.**	**2564047**
**Empirical formula**	$C_{16}H_{16}N_2O_2$
**Formula weight**	268.31
**Temperature/K**	100.0(1)
**Crystal system**	triclinic
**Space group**	*P*-1 (2)
***a*/Å**	8.3521(2)
***b*/Å**	8.7888(2)
***c*/Å**	9.5903(3)
**α/°**	81.240(2)
**β/°**	82.865(2)
**γ/°**	85.958(2)
**Volume/Å^3**	689.42(3)
Z	2
**ρ_{calc} g/cm^3**	1.292
**μ/mm^{-1}**	0.697
***F*(000)**	284
**Crystal size/mm^3**	0.222×0.153×0.095

**Crystal colour**	clear orange
**Crystal shape**	block
**Radiation**	Cu K_α (λ=1.54184)
**2Θ range/°**	9.39 to 158.83
**Index ranges**	-10 ≤ h ≤ 10, -10 ≤ k ≤ 10, -10 ≤ l ≤ 12
**Reflections collected**	17439
**Independent reflections**	2719 [R_{int} = 0.0364, R_{sigma} = 0.0219]
**Data / Restraints / Param.**	2719/0/185
**Goodness-of-fit on F^2**	1.060
**Final R indexes [I≥2σ(I)]**	R_1 = 0.0350, wR_2 = 0.0906
**Final R indexes [all data]**	R_1 = 0.0378, wR_2 = 0.0926
**Largest peak/hole /eÅ^3**	0.16/-0.21

Table S16: Fractional Atomic Coordinates (×10^4) and Equivalent Isotropic Displacement Parameters (Å^2×10^3) for **4d**. U_{eq} is defined as 1/3 of the trace of the orthogonalised U_{IJ} tensor.

**Atom**	**x**	**y**	**z**	**U(eq)**
**O1**	5841.0(9)	2301.3(9)	966.3(9)	25.4(2)
**O2**	121.9(9)	94.4(9)	1655.1(9)	27.8(2)
**N1**	6011.1(11)	4541.8(11)	2698.0(10)	21.7(2)
**N2**	5214.8(11)	4711.5(11)	1658.5(10)	22.7(2)
**C1**	6423.8(13)	5947.4(13)	3106.0(12)	20.6(2)
**C2**	5992.2(13)	7403.1(13)	2393.5(12)	23.6(3)
**C3**	6373.6(14)	8714.5(13)	2878.7(13)	25.7(3)
**C4**	7178.0(14)	8610.2(13)	4089.0(12)	24.5(3)
**C5**	7529.8(16)	10047.5(14)	4649.7(14)	31.5(3)
**C6**	7625.0(13)	7156.4(13)	4766.4(12)	23.8(3)
**C7**	7265.0(13)	5802.1(13)	4301.7(12)	21.8(2)
**C8**	7758.5(15)	4249.5(13)	5078.3(13)	26.3(3)
**C9**	4809.7(13)	3257.4(12)	1302.6(12)	20.9(2)
**C10**	3046.8(13)	3112.6(12)	1352.9(11)	19.7(2)
**C11**	1976.3(13)	4395.5(13)	1426.1(12)	21.3(2)

**C12**	327.0(13)	4192.1(13)	1565.6(12)	23.0(2)
**C13**	-249.4(13)	2746.0(13)	1636.6(12)	23.4(2)
**C14**	828.4(13)	1463.2(13)	1552.9(12)	21.7(2)
**C15**	1152.0(15)	-1260.5(14)	1574.2(15)	30.9(3)
**C16**	2483.5(13)	1639.6(13)	1398.7(11)	20.7(2)

Table S17: Anisotropic Displacement Parameters ($Å^2 \times 10^3$) for **4d**. The Anisotropic displacement factor exponent takes the form: $-2\pi^2[h^2a^{*2}U_{11}+2hka^*b^*U_{12}+\ldots]$.

**Atom**	**x**	**y**	**z**	**U(eq)**
**O1**	5841.0(9)	2301.3(9)	966.3(9)	25.4(2)
**O2**	121.9(9)	94.4(9)	1655.1(9)	27.8(2)
**N1**	6011.1(11)	4541.8(11)	2698.0(10)	21.7(2)
**N2**	5214.8(11)	4711.5(11)	1658.5(10)	22.7(2)
**C1**	6423.8(13)	5947.4(13)	3106.0(12)	20.6(2)
**C2**	5992.2(13)	7403.1(13)	2393.5(12)	23.6(3)
**C3**	6373.6(14)	8714.5(13)	2878.7(13)	25.7(3)
**C4**	7178.0(14)	8610.2(13)	4089.0(12)	24.5(3)
**C5**	7529.8(16)	10047.5(14)	4649.7(14)	31.5(3)
**C6**	7625.0(13)	7156.4(13)	4766.4(12)	23.8(3)
**C7**	7265.0(13)	5802.1(13)	4301.7(12)	21.8(2)
**C8**	7758.5(15)	4249.5(13)	5078.3(13)	26.3(3)
**C9**	4809.7(13)	3257.4(12)	1302.6(12)	20.9(2)
**C10**	3046.8(13)	3112.6(12)	1352.9(11)	19.7(2)
**C11**	1976.3(13)	4395.5(13)	1426.1(12)	21.3(2)
**C12**	327.0(13)	4192.1(13)	1565.6(12)	23.0(2)
**C13**	-249.4(13)	2746.0(13)	1636.6(12)	23.4(2)
**C14**	828.4(13)	1463.2(13)	1552.9(12)	21.7(2)
**C15**	1152.0(15)	-1260.5(14)	1574.2(15)	30.9(3)
**C16**	2483.5(13)	1639.6(13)	1398.7(11)	20.7(2)

Table S18: Bond Lengths for **4d**

Atom	Atom	Length/Å	Atom	Atom	Length/Å
O1	C9	1.2097(13)	C4	C6	1.3907(17)
O2	C14	1.3620(14)	C6	C7	1.3968(16)
O2	C15	1.4253(14)	C7	C8	1.5080(16)
N1	N2	1.2506(13)	C9	C10	1.4809(15)
N1	C1	1.4279(14)	C10	C11	1.3936(15)
N2	C9	1.4446(14)	C10	C16	1.4010(15)
C1	C2	1.4013(16)	C11	C12	1.3889(16)
C1	C7	1.4027(16)	C12	C13	1.3797(16)
C2	C3	1.3779(16)	C13	C14	1.3980(16)
C3	C4	1.3998(17)	C14	C16	1.3889(16)
C4	C5	1.5065(16)			

Table S19: Bond Angles for **4d**

Atom	Atom	Atom	Angle/˚	Atom	Atom	Atom	Angle/˚
C14	O2	C15	117.84(9)	C6	C7	C8	120.52(10)
N2	N1	C1	114.59(9)	O1	C9	N2	121.70(10)
N1	N2	C9	112.37(9)	O1	C9	C10	125.22(10)
C2	C1	N1	122.99(10)	N2	C9	C10	113.05(9)
C2	C1	C7	120.85(10)	C11	C10	C9	120.78(10)
C7	C1	N1	116.14(10)	C11	C10	C16	121.05(10)
C3	C2	C1	119.96(11)	C16	C10	C9	118.09(10)
C2	C3	C4	120.64(11)	C12	C11	C10	118.90(10)
C3	C4	C5	120.39(11)	C13	C12	C11	120.81(10)
C6	C4	C3	118.61(11)	C12	C13	C14	120.12(10)
C6	C4	C5	121.00(11)	O2	C14	C13	114.93(10)

C4	C6	C7	122.33(11)	O2	C14	C16	124.94(10)
C1	C7	C8	121.89(10)	C16	C14	C13	120.13(10)
C6	C7	C1	117.59(10)	C14	C16	C10	118.97(10)

Table S20: Torsion Angles for **4d**

**A**	**B**	**C**	**D**	**Angle/˚**	**A**	**B**	**C**	**D**	**Angle/˚**
O1	C9	C10	C11	-164.88(11)	C2	C3	C4	C6	1.83(17)
O1	C9	C10	C16	18.35(17)	C3	C4	C6	C7	-1.58(17)
O2	C14	C16	C10	-177.93(10)	C4	C6	C7	C1	0.08(16)
N1	N2	C9	O1	-60.25(14)	C4	C6	C7	C8	-179.36(10)
N1	N2	C9	C10	121.58(10)	C5	C4	C6	C7	177.68(10)
N1	C1	C2	C3	177.40(10)	C7	C1	C2	C3	-0.96(17)
N1	C1	C7	C6	-177.26(9)	C9	C10	C11	C12	-175.81(10)
N1	C1	C7	C8	2.18(15)	C9	C10	C16	C14	175.26(10)
N2	N1	C1	C2	-0.73(15)	C10	C11	C12	C13	0.25(17)
N2	N1	C1	C7	177.69(9)	C11	C10	C16	C14	-1.51(16)
N2	C9	C10	C11	13.21(14)	C11	C12	C13	C14	-0.70(17)
N2	C9	C10	C16	-163.56(9)	C12	C13	C14	O2	179.11(10)
C1	N1	N2	C9	-179.17(8)	C12	C13	C14	C16	0.03(17)
C1	C2	C3	C4	-0.60(17)	C13	C14	C16	C10	1.05(16)
C2	C1	C7	C6	1.20(16)	C15	O2	C14	C13	179.84(10)
C2	C1	C7	C8	-179.36(10)	C15	O2	C14	C16	-1.13(17)
C2	C3	C4	C5	-177.43(11)	C16	C10	C11	C12	0.86(16)

Table S21: Hydrogen Atom Coordinates (Å×10^4) and Isotropic Displacement Parameters (Å^2×10^3) for **4d**

**Atom**	**x**	**y**	**z**	**U(eq)**
H2	5437.06	7485.66	1576.42	28
H3	6087.82	9699.05	2387.21	31
H5AA	6912.06	10078.51	5583.99	47

H5AB	7219.23	10956.76	3993.22	47
H5AC	8687.04	10042.16	4736.68	47
H5BD	6528.41	10684.86	4789.99	47
H5BE	8314.74	10630.59	3966.16	47
H5BF	7975.18	9761.97	5557.73	47
H6	8195.26	7081.81	5573.77	29
H8A	6791.17	3690.87	5472.98	39
H8B	8374.48	4392.03	5850.29	39
H8C	8430.53	3656.7	4416.49	39
H11	2368.2	5392.63	1381.37	26
H12	-413.26	5059.43	1612.71	28
H13	-1380.77	2621.66	1742.57	28
H15A	1838.04	-1154.12	662.03	46
H15B	493.47	-2156.31	1654.21	46
H15C	1832.92	-1402.79	2350.68	46

Molecular Docking

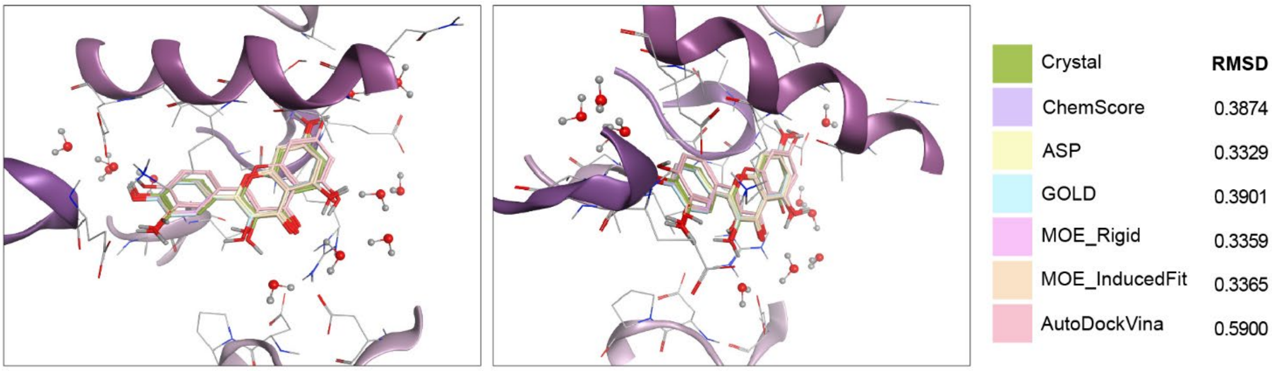

Figure S7: Overlay of quercetin crystal structure and docked quercetin from different methods (ChemScore, ASP, GOLD, MOE Rigid, MOE Induce-Fit, AutoDock Vina).

Table S22 Consensus docking rank by rank analysis compounds 9a, 10a, 14a, 11b, 12b, CJ1-34, Quercetin and Caffeine

	MOE	GOLD	ASP	Chem	Consensus Rank
Quercetin	7	7	2	2	3.74
CJ1-34	6	1	1	5	2.34
9a	1	5	4	4	2.99
10a	4	2	5	1	2.51
14a	5	6	3	3	4.05
11b	2	4	7	6	4.28
12b	3	3	6	7	4.41
Caffeine	8	8	8	8	8.00

Table S23 Consensus docking cluster analysis of compounds 9a, 10a, 14a, 11b, 12b, CJ1-34, Quercetin and Caffeine

**Entry**	**Largest cluster (Nr. Poses)**	**Scoring functions supporting main cluster**	**Centroid Pose**
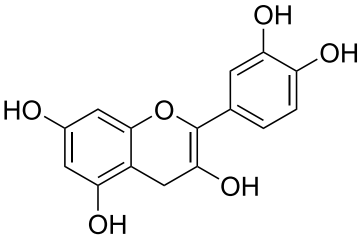 **Quercetin**	13	MOE, GOLD, ASP	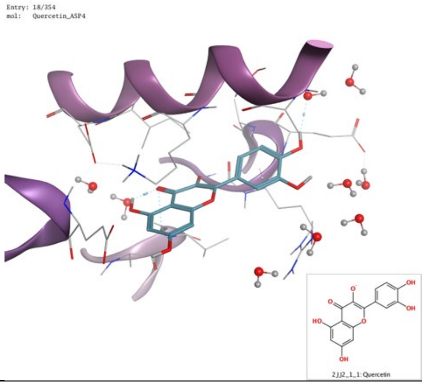
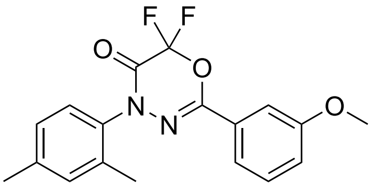 **CJ1-34**	5	MOE, GOLD, ASP	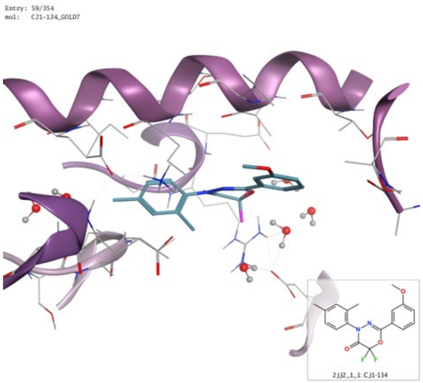

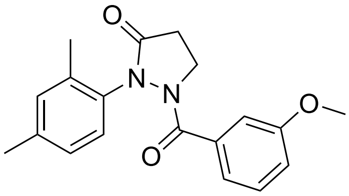 **9a**	5	CHEM, ASP	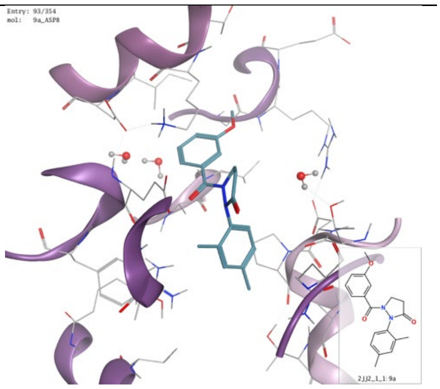
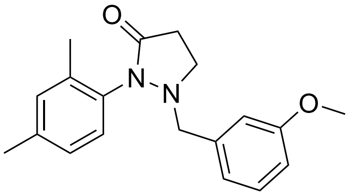 **10a**	5	MOE, GOLD, CHEM	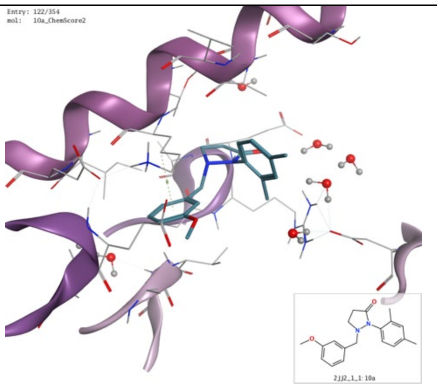
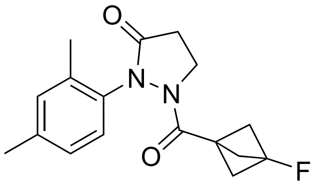 **14a**	11	MOE, GOLD, CHEM, ASP	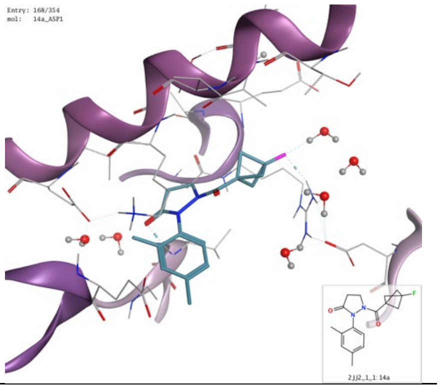
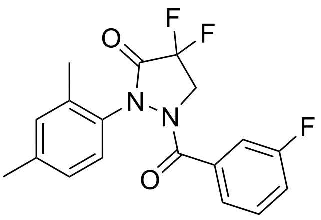 **11b**	5	MOE, GOLD, ASP	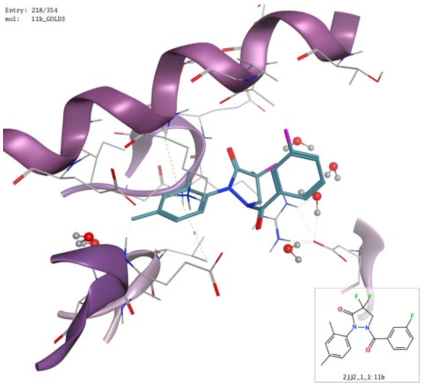

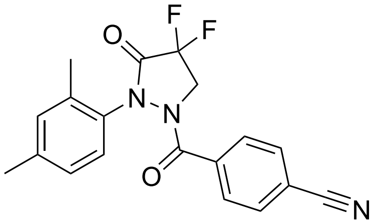 **12b**	4	CHEM, ASP	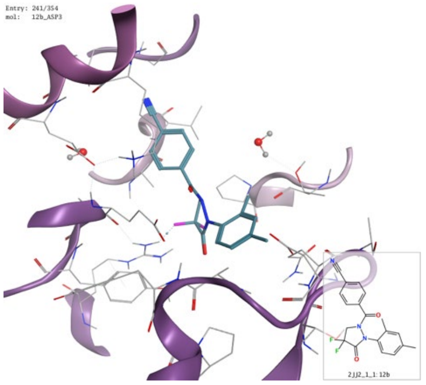

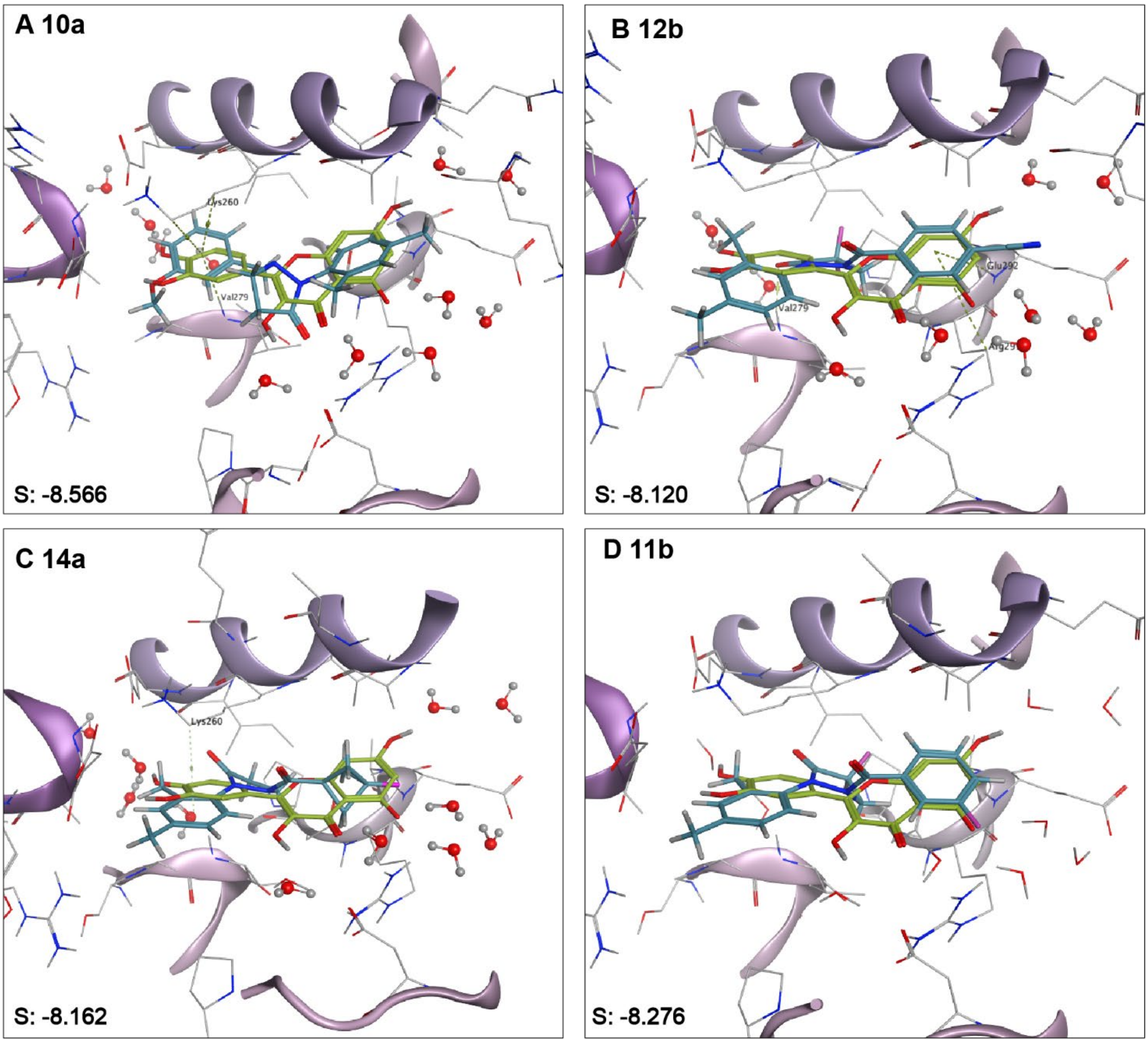

Figure S8 A) Overlay of compound **10a** with quercetin showing π–hydrogen interactions with Lys260 and Val279. **B**) Overlay of compound **12b** with quercetin showing π–hydrogen interactions with Arg291 and Glu292 as well as a hydrogen interaction between the carboxylic oxygen and Val279. **C**) Overlay of compound **14a** with quercetin showing π–hydrogen interaction with Lys260. **D**) Overlay of compound **11b** with quercetin.

Table S24: Induced Fit Docking and Scores of compounds CJ1-34, 9a-16a and 9b-12b

Ligand	Docking Score [kcal/mol]	Best Pose
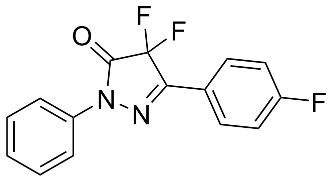 **5b**	-7.2702708	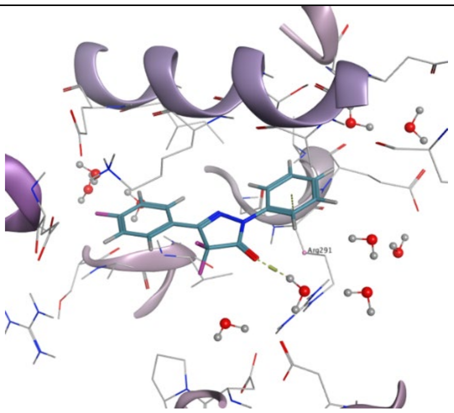
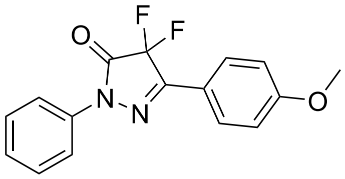 **5c**	-7.668643	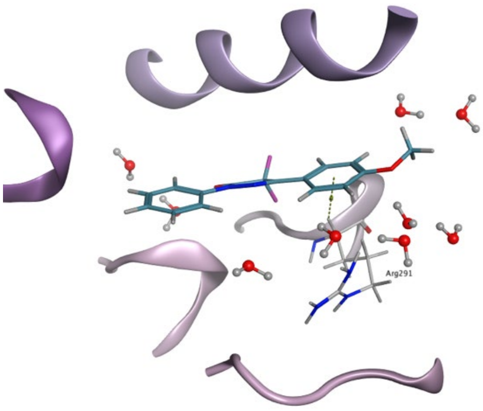
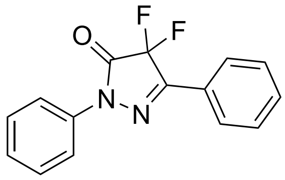 **5a**	-7.0856895	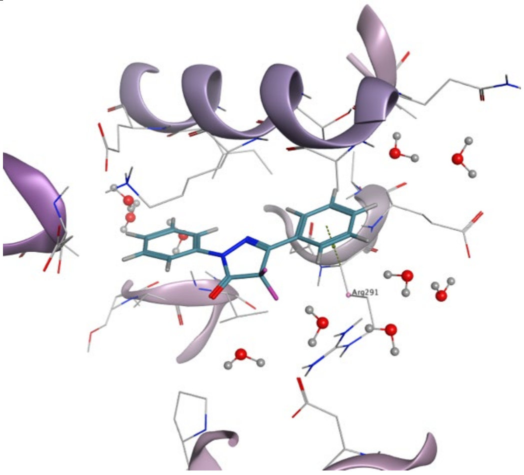

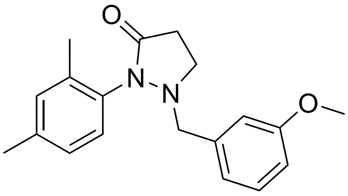<br>**10a**	-8.5660248	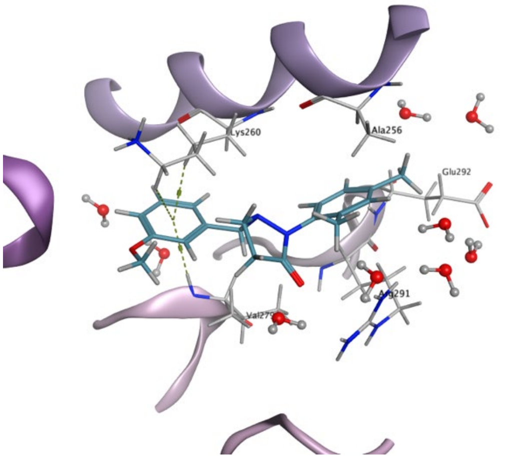
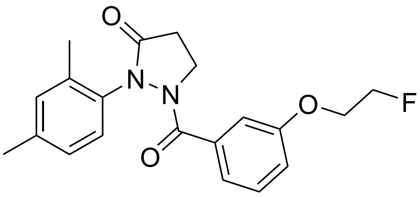<br>**16a**	-8.9375725	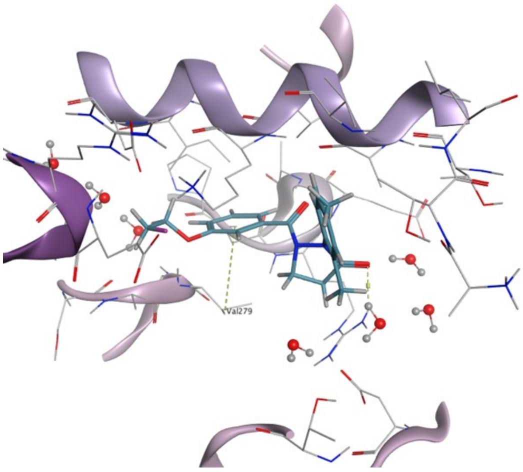
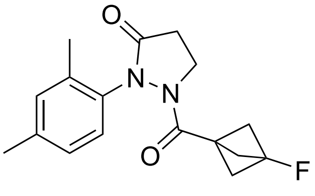<br>**14a**	-8.1626406	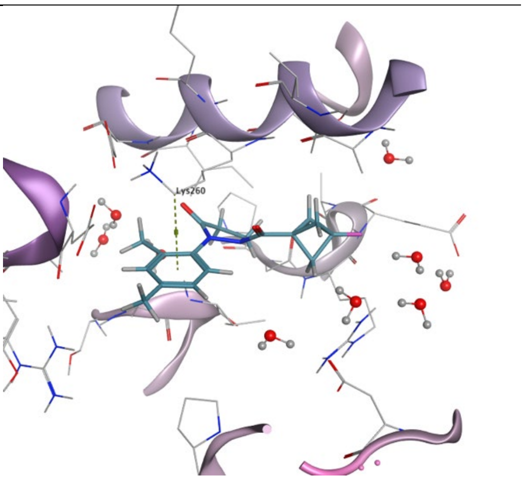

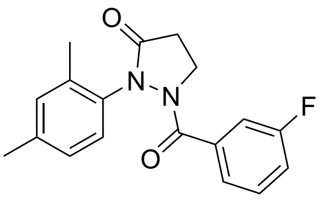 **11a**	-8.2571039	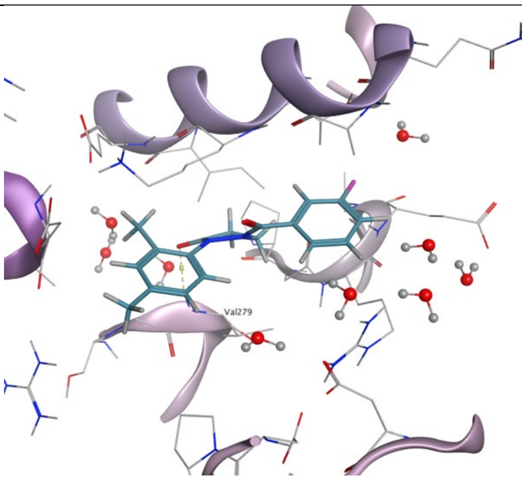
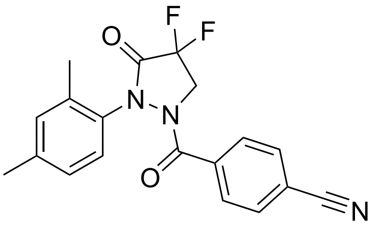 **12b**	-8.1204166	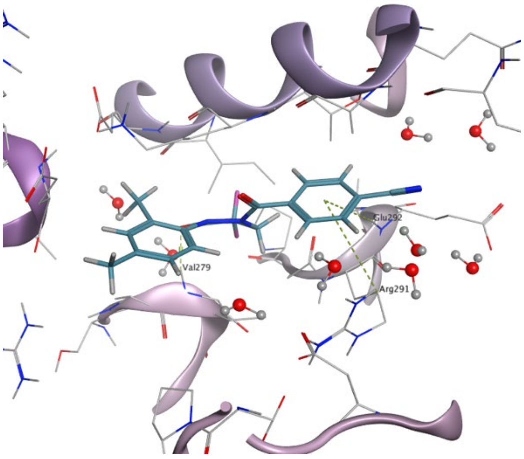
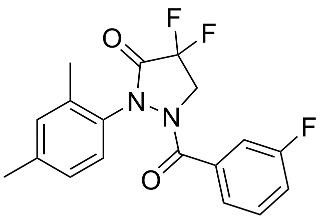 **11b**	-8.2764587	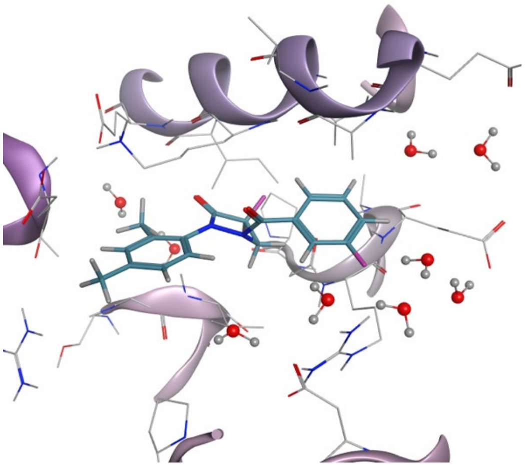

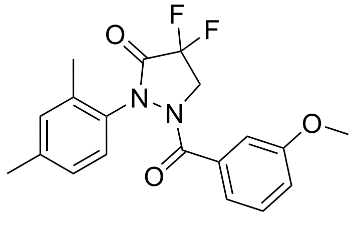 **9b**	-8.2925806	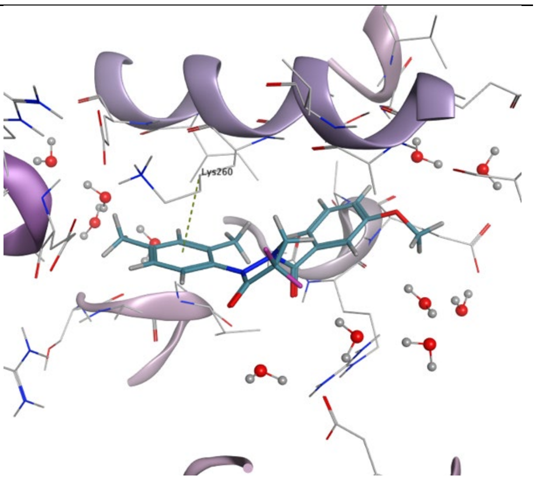
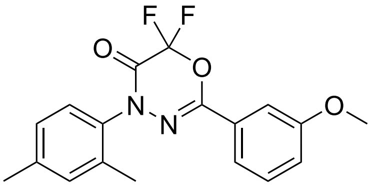 **CJ1-34**	-8.5812311	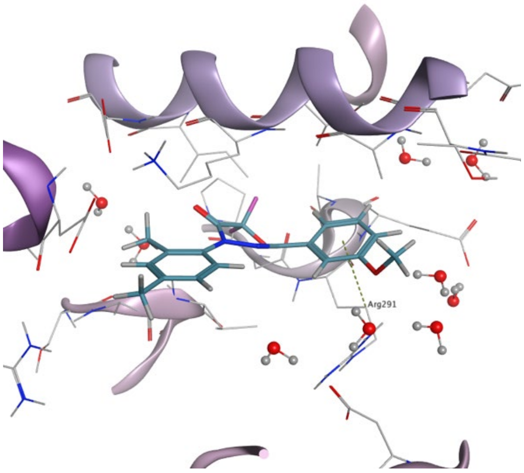
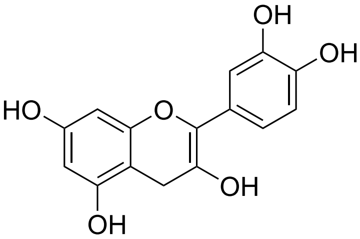 **Quercetin**	-8.3838863	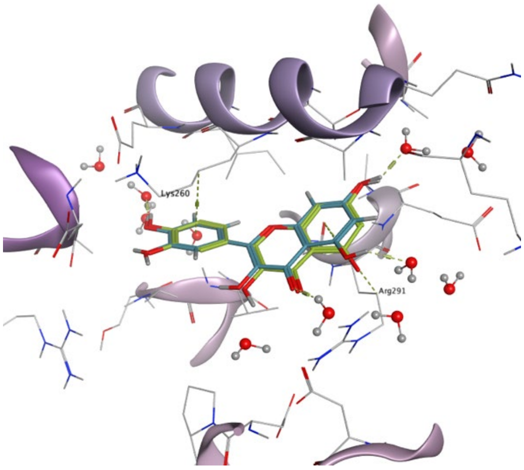

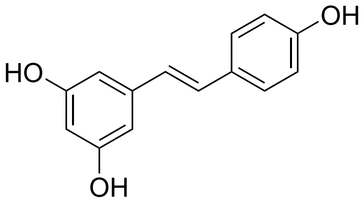 **Resveratrol**	-7.701282	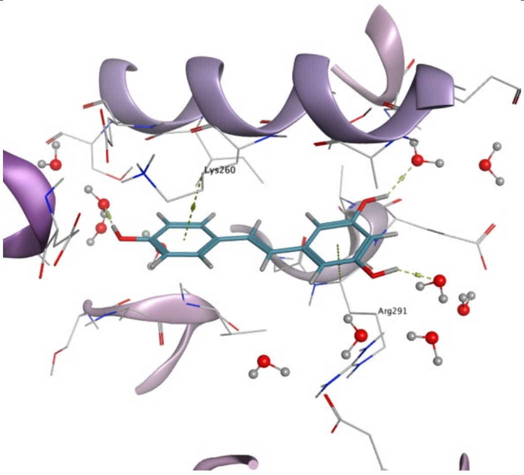
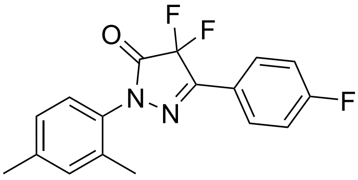 **5e**	-8.4964523	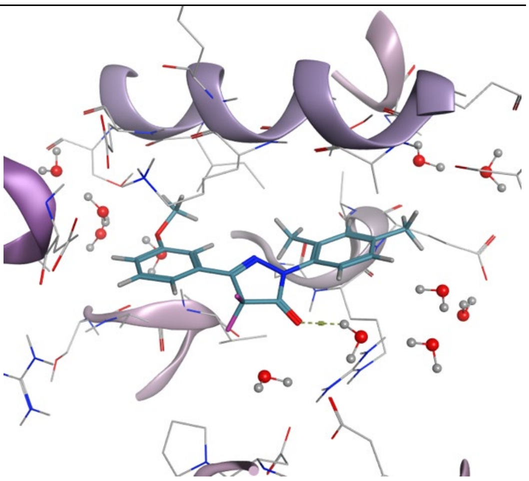
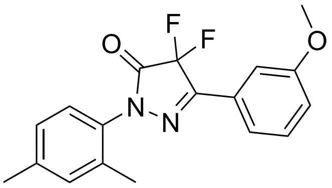 **5d**	-7.1219988	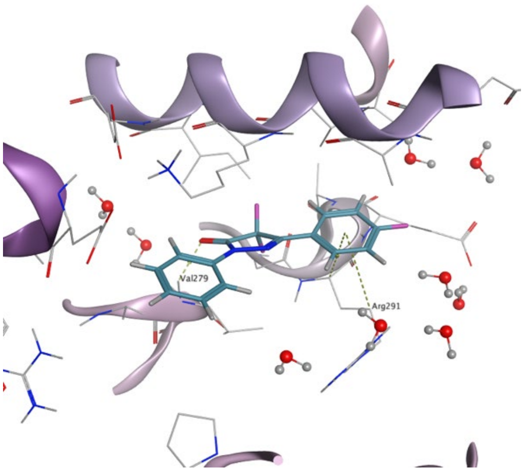

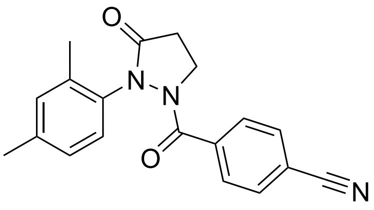 **12a**	-8.4325647	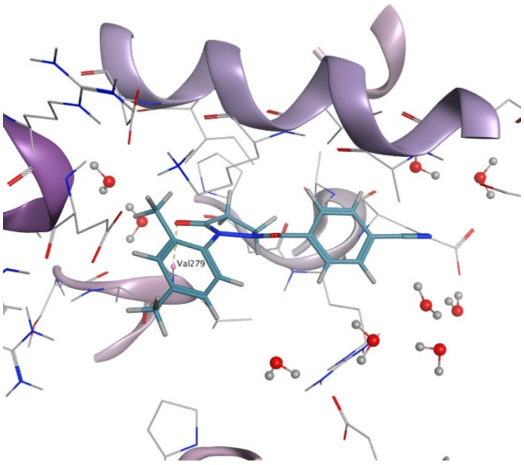
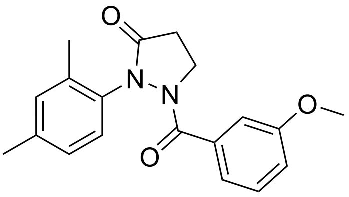 **9a**	-8.6251478	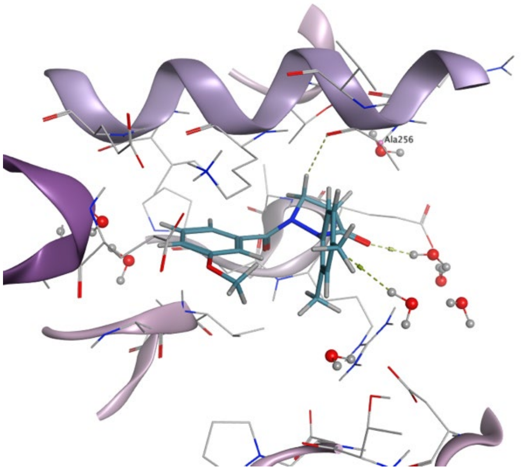
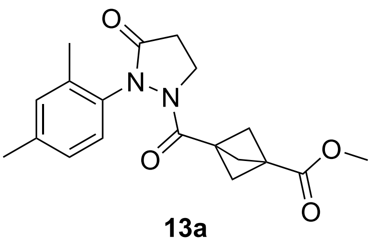 **13a**	-8.9158192	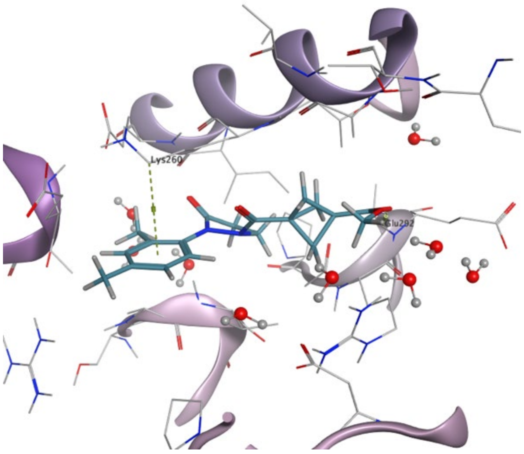

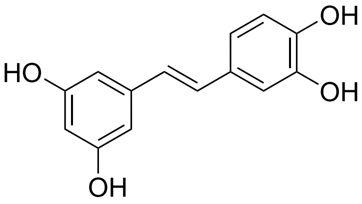 **Piceatannol**	-7.9761968	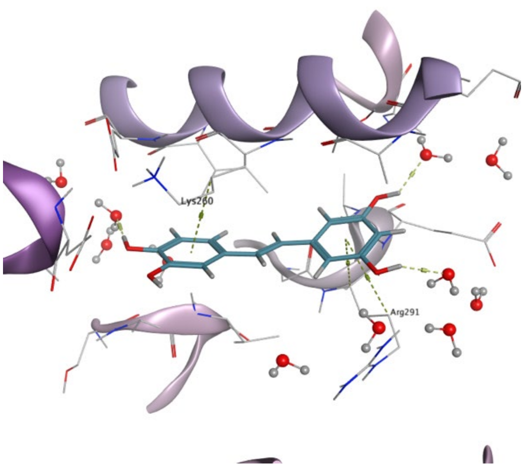
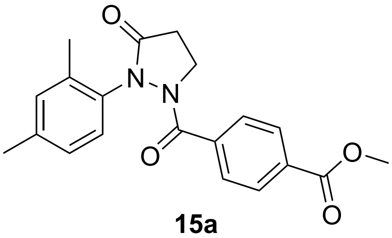 **15a**	-8.7779398	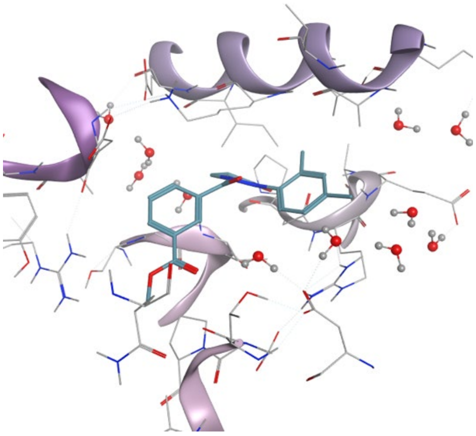

***In vitro* assays**

Ethical Statement

The animal experiments were carried out in compliance with Swiss Animal Welfare legislation according to Art.18 Tierschutzgesetz (TSchG), Art. 141 Tierschutzverordnung (TschV), Art. 30 Tierversuchsverordnung (TVV) (all Switzerland). Experiments were approved by the cantonal veterinary office of Zurich, Switzerland, under the license 34491 (National number).

ATP hydrolysis in isolated mitochondria

Isolation of functional mouse liver mitochondria

Functional mouse liver mitochondria were isolated according to an adapted procedure[7,8]. Buffer A (0.2 M Tris, pH 7.4 adjusted using 3-morpholinopropane-1-sulfonic acid (MOPS)), Buffer B (0.1 M egtazic acid, pH 7.4 adjusted using Tris) and Buffer C (1 M sucrose) were prepared in distilled water. The isolation buffer was prepared fresh on the day of the experiment by mixing 10 mL A, 1 mL B, 20 mL C and adjusting the final volume to 100 mL using distilled water. For all procedures, buffer, falcon tubes, agar plate, glass potter and centrifuge were precooled to 4 °C. An adult C57BL/6 mouse was euthanized using decapitation following the use of isofluorane anaesthesia (isofluorane/air 1:1). The liver was removed and washed four times in cooled isolation buffer. After the final wash, the liver was placed on an agar plate and minced into small pieces. Fresh isolation buffer (5 mL) was added to transfer the pieces into a 15 mL homogenizer (4 °C, Braun Biotech International, Potter S). The vessel was filled with isolation buffer to a final volume of 10 mL and homogenization was achieved by carefully moving the rod of the glass potter up and down at 1200 rpm. The homogenate was centrifuged at 600 g (4 °C, 10 min), the supernatant was transferred to a new falcon tube and centrifuged at 7000 g (4 °C, 10 min). The supernatant was discarded and the pellet was gently resuspended using a brush and 5 mL of fresh isolation buffer. The last centrifugation step was repeated (7000 g, 4 °C, 10 min), the supernatant was discarded and the pellet is resuspended in the remaining amount of isolation buffer in the tube. The protein concentration was determined with a BCA assay (PierceTM, Cat No.: 23225) and the isolated liver mitochondria were stored in aliquots at -80 °C.

Assay procedure

The assay procedure to determine the inhibition of the hydrolytic capacity of complex V was adapted from previously published literature[8–10]. The following solutions were prepared in nanopure water and stored in aliquots at -20 °C: Assay buffer (25 mM HEPES, 25 mM KCl, 2 mM $MgCl_2$ hexahydrate, pH 7.5-8.0 adjusted using KOH), Tris buffer (1 M tris-HCl, pH 8.1 adjusted using KOH), KCl solution (200 mM), $MgCl_2$ solution, (1 M), enzyme mix A (2000 U/mL pyruvate kinase (PK)) enzyme mix B (2740 U/mL LDH). Furthermore, stock solutions of oligomycin A (1.26 mM) in DMSO, FCCP (40 mM) in EtOH and antimycin A (18.2 mM) in EtOH were prepared and stored at -20 °C. On the day of the experiment, all aliquots were thawed on ice, except for the assay and Tris-buffer which were thawed at r.t.. ATP (10 mM in tris-buffer), PEP monopotassium (40 mM in Tris-buffer) and NADH (12 mM in nanopure water) were freshly prepared and kept on ice. An aliquot of isolated liver mitochondria was diluted with assay buffer to a concentration of 5 mg/mL, frozen in liquid nitrogen for 3 min and thawed in a thermomixer (Eppendorf thermomixer, Cat: 5436) at 37 °C. The freezing and thawing cycle was repeated 4 times in total. The mitochondrial suspension was further diluted with assay buffer to a concentration of 1.32 mg/mL and kept on ice. Reaction mix (2.84 mM KCl, 1.42 mM $MgCl_2$, 1.42 U/mL PK, 1.95 U/mL LDH, 0.85 µM FCCP, 0.26 µM antimycin A, 1.13 mM PEP, 638.12 µM NADH) in nanopure water was freshly prepared on ice. The experiment was conducted in a transparent 96-well tissue culture test plate (TPP®). To each well was added reaction mix (85 µL) and mitochondria suspension (25 µL). Compounds were diluted with DMSO to the desired concentration. 1 µL of diluted compound was added in triplicates achieving a final concentration of 100 nM in the well. The reaction was started by adding 2 µL ATP to each well. Immediately, the plate was placed into a preheated microplate reader at 37 °C (Agilent, BioTek Synergy HT) and the absorbance was monitored at 340 nm for 30 min.

Dose-response assessment in isolated mitochondria

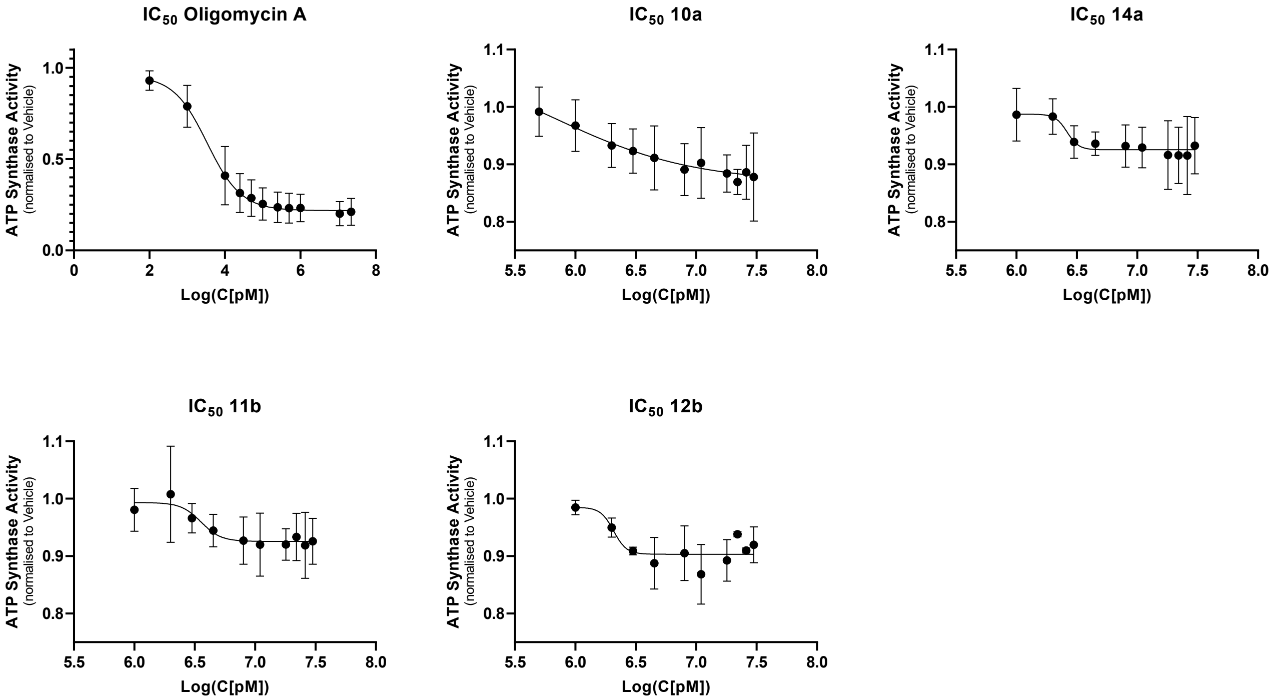

Figure S6: Dose-response curves.

ATP production in HT-22 cells

HT-22 cell line

HT-22 cells (Merck, SCC129) are an immortalized murine hippocampal neuronal cell line derived from HT-4 cells, which are primarily undifferentiated[11]. Cell aliquots were stored in a liquid nitrogen tank. For each experiment initiation, an aliquot was thawed at 37 °C and cells were cultured in pre-warmed Dulbecco's Modified Eagles's Medium (DMEM) supplemented with 10 % fetal bovine serum (FBS) and penicillin. Cells were passaged by trypsination using pre-warmed 0.25 % Trypsin-EDTA (Gibco^{TM}), cultivated in 75 cm^2 culture flasks (TPP AG, Cat No.: 90076). Flasks were maintained in a humidified incubator (Memmet GmbH) at 37 °C and 5 % CO_2. The medium was changed every two to three days to ensure optimal cell growth. Cells were split upon reaching 80-100 % confluence. Cell count was determined with an automated cell counter (Invitrogen TM, CountessTM Automated Cell Counter).

Assay procedure

This procedure was adapted from previously published work.[8] 1 day prior to conducting the assay, cells were counted and 100 µL of cell suspension in DMEM were transferred into inner wells of a white 96-well culture plate with a transparent bottom (Greiner Bio-One International GmbH, Cat No.: 655098). The plated cells were incubated for 24 h at 37 °C and 5 % CO_2. Compound solutions were prepared from stock solutions in DMSO by diluting with DMEM with a final concentration of 1 µM (<0.1 % DMSO) in the cell plate. A vehicle sample (<0.1 % DMSO in DMEM) and oligomycin A with a final concentration of 5 µM in the final cell plate were used as a positive controls. Each experiment was conducted in triplicates. Compound and vehicle-treated cells were incubated for 15 min. In a second well plate, ATP standards for a calibration curve were prepared. 50 µL per well of ATP standards in Phosphate-buffered saline (PBS) (0, 1, 2, 3, 5, 10 and 15 µM) were added to a clear 96-well plate in triplicates. 25 µL of Cell titer-Glo buffer was added to each ATP standard well and the plate was shaken on an orbital shaker (Edmund Bühler TH 30 Shaker) at 130 rpm for 15 min.
The cell plate with the incubated compounds and controls was washed with 100 µL PBS, followed by adding 50 µL of PBS for each well. Additionally, 25 µL of cell-titer Glo buffer was added into the wells and the plate was then shaken on an orbital shaker at 130 rpm for 15 min to lyse the cells. 10 µL aliquots from each well were pipetted from all ATP standard samples and all test samples into a clear 96-wellplate for protein content determination.

5 mL of Cell titer-Glo buffer were combined with Cell titer-Glo substrate in the dark and vortexed for a few seconds. 2xcell titer-Glo reagents (25 µL per well) were added to both the ATP standards and the test samples, and both were incubated in the dark for 30 min under light shaking. Subsequently, luminescence of ATP standard samples and test samples was measured at 530/25 nm using a microplate reader (Synergy HT, BioTek Instruments GmbH).
A BCA protein determination assay (Pierce™ BCA Protein Assay Kit, Thermo Scientific) was conducted with the aliquots taken from each sample. 50 parts of BCA reagent A were mixed with 1 part of BCA reagent B and 100 µL of this mixture were added into each aliquot well. The plate was incubated at 37 °C for 30 min. Subsequently, the absorbance of the samples was measured at 562 nm using a microplate reader.
The cellular ATP content was calculated using the ATP standard curve and normalized to cellular protein content/well.

Radiolabeling

Semi-preparative HPLC chromatogram

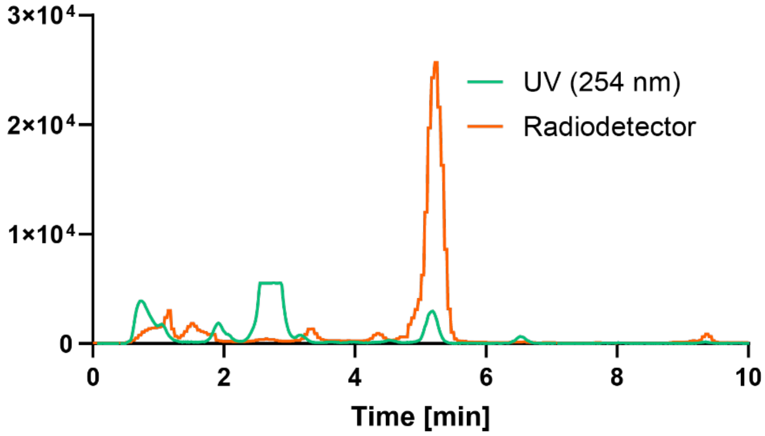

Column: ACE 3 C18, mobile phase A: 0.1 % H_3PO_4 in water, mobile phase B: 5 % water in MeCN, gradient method: 0.0-5.0 min, 35-45 % B; 5.0-8.0 min, 45-65 % B; 8.0-12.0 min, 65-75 % B; 12.0-21.0 min, 75-85 %, 21.0-25.0 min, 85-35 % B; flow=4 mL/min

Analytical HPLC chromatogram

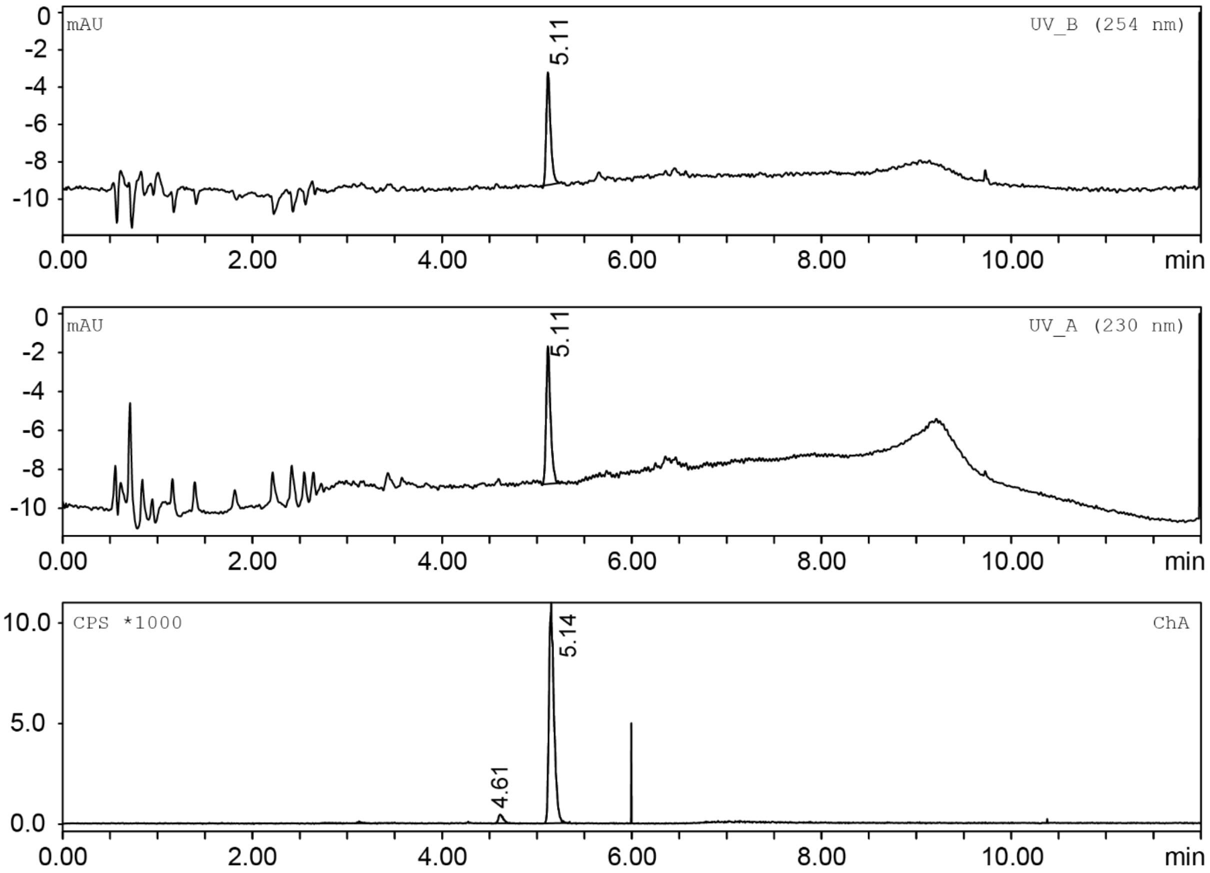

Column: Agilent Zorbax 3.5 µm XDB-C18; 75x4.6 mm; mobile phase A: 0.1 % H_3PO_4 in water, mobile phase B: 5 % water in MeCN, gradient method:0.0-5.0 min, 10 % B, 5.0-7.0 min, 10-85 % B, 7.0-10.0 min, 85-95 % B, 10.0-12.0 min, 95-10 % B; flow=1 mL/min

Calibration curve

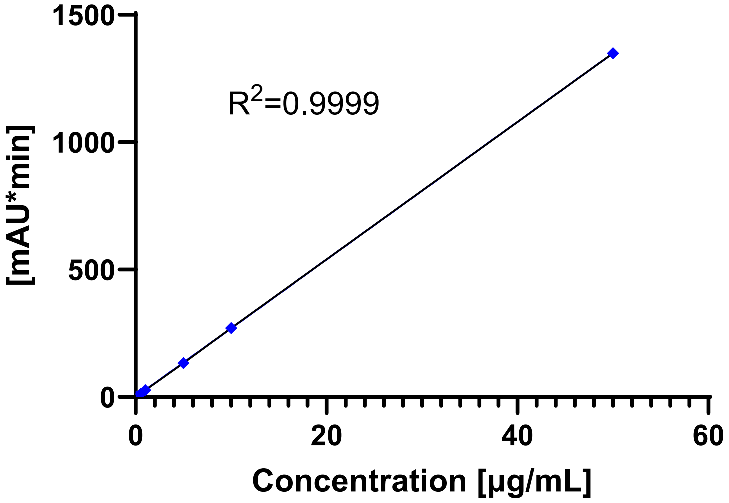

References

[1] S. Yamamura, M. Toda, Y. Hirata, “MODIFIED CLEMMENSEN REDUCTION: CHOLESTANE” *Organic Syntheses* **1973**, *53*, 86.

[2] Y. L. Zhao Shuai; Cui Jinjie; Wang Huimin; Kang Xin; Wang Yan; Tian Laijin, “Selectfluor-Mediated Oxidative Dehydrogenation of Hydrazines: A Process for the Synthesis of Azo Compounds” *Synthesis (Stuttg).* **2022**, *54*, 5245–5252.

[3] A. C. Savva, S. I. Mirallai, G. A. Zissimou, A. A. Berezin, M. Demetriades, A. Kourtellaris, C. P. Constantinides, C. Nicolaides, T. Trypiniotis, P. A. Koutentis, “Preparation of Blatter Radicals via Aza-Wittig Chemistry: The Reaction of N-Aryliminophosphoranes with 1-(Het)aroyl-2-aryldiazenes” *J. Org. Chem.* **2017**, *82*, 7564–7575.

[4] T. T. Baburaj Sivalingam, “N-Boc-O-Tosyl Hydroxylamine as a Safe and Efficient Nitrogen Source for the N-Amination of Aryl and Alkyl Amines: Electrophylic Amination” *Synlett* **2011**, *22*, 1993–1996.

[5] Y. L. Goh, V. A. Adsool, “Radical fluorination powered expedient synthesis of 3-fluorobicyclo[1.1.1]pentan-1-amine” *Org. Biomol. Chem.* **2015**, *13*, 11597–11601.

[6] K. L. Strong, M. P. Epplin, J. Bacsa, C. J. Butch, P. B. Burger, D. S. Menaldino, S. F. Traynelis, D. C. Liotta, “The Structure–Activity Relationship of a Tetrahydroisoquinoline Class of N-Methyl-d-Aspartate Receptor Modulators that Potentiates GluN2B-Containing N-Methyl-d-Aspartate Receptors” *J. Med. Chem.* **2017**, *60*, 5556–5585.

[7] C. Frezza, S. Cipolat, L. Scorrano, “Organelle isolation: Functional mitochondria from mouse liver, muscle and cultured filroblasts” *Nat. Protoc.* **2007**, *2*, 287–295.

[8] T. Serdiuk, Y. Fleischmann, D. Ghosh, A. Delparente, V. Reber, L. Frey, D. Rhyner, J. Gerez, N. Volkmar, L. Kralickova, G. Mas, C. Dörig, S. Hiller, R. Schibli, L. Mu, P. Picotti, R. Riek, “Alpha-synuclein interacts with regulators of ATP homeostasis in mitochondria” *Nat. Commun.* **2025**, *16*, 7651.

[9] F. Haraux, A. Lombès, “Kinetic analysis of ATP hydrolysis by complex V in four murine tissues: Towards an assay suitable for clinical diagnosis” *PLoS One* **2019**, *14*, e0221886-.

[10] L. Fernandez-del-Rio, C. Benincá, F. Villalobos, C. Shu, L. Stiles, M. Liesa, A. S. Divakaruni, R. Acin-Perez, O. S. Shirihai, “A novel approach to measure complex V ATP hydrolysis in frozen cell lysates and tissue homogenates” *Life Sci. Alliance* **2023**, *6*, e202201628.

[11] J. B. Davis, P. Maher, “Protein kinase C activation inhibits glutamate-induced cytotoxicity in a neuronal cell line” *Brain Res.* **1994**, *652*, 169–173.
